# Late Mortality After COVID-19 Infection Among US Veterans vs Risk-Matched Comparators: A 2-Year Cohort Analysis

**DOI:** 10.1001/jamainternmed.2023.3587

**Published:** 2023-08-21

**Authors:** Theodore J. Iwashyna, Sarah Seelye, Theodore S. Berkowitz, John Pura, Amy S. B. Bohnert, C. Barrett Bowling, Edward J. Boyko, Denise M. Hynes, George N. Ioannou, Matthew L. Maciejewski, Ann M. O’Hare, Elizabeth M. Viglianti, James Womer, Hallie C. Prescott, Valerie A. Smith

**Affiliations:** 1 Veterans Affairs (VA) Center for Clinical Management Research, Ann Arbor VA, Ann Arbor, Michigan; 2 Department of Medicine, University of Michigan Medical School, Ann Arbor; 3 School of Medicine, Johns Hopkins University, Baltimore, Maryland; 4 School of Public Health, Johns Hopkins University, Baltimore, Maryland; 5 Center of Innovation to Accelerate Discovery and Practice Transformation, Durham VA Medical Center, Durham, North Carolina; 6 Departments of Anesthesiology and Psychiatry, University of Michigan Medical School, Ann Arbor; 7 Durham VA Geriatric Research Education and Clinical Center, Durham VA Medical Center, Durham, North Carolina; 8 Department of Medicine, Duke University, Durham, North Carolina; 9 VA Puget Sound Health Care System Hospital and Specialty Medicine Service and Seattle-Denver Center of Innovation for Veteran Centered and Value Driven Care, Seattle, Washington; 10 Department of Medicine, University of Washington, Seattle; 11 VA Center to Improve Veteran Involvement in Care, Portland, Oregon; 12 College of Public Health and Human Sciences and Center for Quantitative Life Sciences, Oregon State University, Corvallis; 13 School of Nursing, Oregon Health and Science University, Portland; 14 Department of Population Health Sciences, Duke University, Durham, North Carolina

## Abstract

**Importance:**

Despite growing evidence of persistent problems after acute COVID-19, how long the excess mortality risk associated with COVID-19 persists is unknown.

**Objective:**

To measure the time course of differential mortality among Veterans who had a first-documented COVID-19 infection by separately assessing acute mortality from later mortality among matched groups with infected and uninfected individuals who survived and were uncensored at the start of each period.

**Design, Settings, and Participants:**

This retrospective cohort study used prospectively collected health record data from Veterans Affairs hospitals across the US on Veterans who had COVID-19 between March 2020 and April 2021. Each individual was matched with up to 5 comparators who had not been infected with COVID-19 at the time of matching. This match balanced, on a month-by-month basis, the risk of developing COVID-19 using 37 variables measured in the 24 months before the date of the infection or match. A primary analysis censored comparators when they developed COVID-19 with inverse probability of censoring weighting in Cox regression. A secondary analysis did not censor. Data analyses were performed from April 2021 through June 2023.

**Exposure:**

First-documented case of COVID-19 (SARS-CoV-2) infection.

**Main Outcome Measures:**

Hazard ratios for all-cause mortality at clinically meaningful intervals after infection: 0 to 90, 91 to 180, 181 to 365, and 366 to 730 days.

**Results:**

The study sample comprised 208 061 Veterans with first-documented COVID-19 infection (mean [SD] age, 60.5 (16.2) years; 21 936 (10.5) women; 47 645 [22.9] Black and 139 604 [67.1] White individuals) and 1 037 423 matched uninfected comparators with similar characteristics. Veterans with COVID-19 had an unadjusted mortality rate of 8.7% during the 2-year period after the initial infection compared with 4.1% among uninfected comparators, with censoring if the comparator later developed COVID-19—an adjusted hazard ratio (aHR) of 2.01 (95% CI, 1.98-2.04). The risk of excess death varied, being highest during days 0 to 90 after infection (aHR, 6.36; 95% CI, 6.20-6.51) and still elevated during days 91 to 180 (aHR, 1.18; 95% CI, 1.12-1.23). Those who survived COVID-19 had decreased mortality on days 181 to 365 (aHR, 0.92; 95% CI, 0.89-0.95) and 366 to 730 (aHR, 0.89; 95% CI, 0.85-0.92). These patterns were consistent across sensitivity analyses.

**Conclusion and Relevance:**

The findings of this retrospective cohort study indicate that although overall 2-year mortality risk was worse among those infected with COVID-19, by day 180 after infection they had no excess mortality during the next 1.5 years.

## Key Points

**Question:**

Do survivors of acute COVID-19 have ongoing excess mortality in the months and years after acute illness?

**Findings:**

In this retrospective cohort study of 208 061 Veterans infected with COVID-19 and 1 037 423 matched uninfected comparators, COVID-19 survivors had no clinically significant excess hazard of death greater than comparators among those who survived at least 6 months after infection.

**Meaning:**

For ongoing clinical care after COVID-19, efforts that focus on improving survival outcomes may be less relevant than approaches that address the needs of individual survivors.

## Teaser

This retrospective cohort study of COVID-19 survivors and matched uninfected comparators assesses the time course of excess mortality after COVID-19.

## Introduction

The lives lost to acute COVID-19 are well documented.^1,2,3,4,5,6,7,8^ Less clear is whether survivors of COVID-19 experience excess mortality after acute infection, and if so, how long it persists. The potential for excess mortality after acute infection may be hypothesized on the basis of (1) the incident and exacerbated diagnoses after acute COVID-19,^9^ (2) by analogy to sepsis (where immune dysregulation, functional impairment, and other excess mortality can persist long after the initial illness^10,11^), and (3) ongoing disability, cognitive impairment, and rehospitalization after pneumonia.^12,13^ Understanding the time course of excess mortality after COVID-19, if present, would have prognostic implications for the care of COVID-19 survivors (some of whom develop post−COVID-19 condition), and affect health system planning.

We compared the time course of differential mortality among Veterans who had a first-documented COVID-19 infection between March 2020 and April 2021, followed up through April 2022, by separately evaluating acute mortality (eg, on days 0-90 after infection) and later mortality (ie, on days 181-365 and 366-730) among matched groups with infected and uninfected individuals who survived and were uncensored at the start of each period. Using target trial emulation methods (comparison of a target trial vs our observational emulation is available in eTable 1 in Supplement 1), we compared the death rate of these individuals to matched Veterans with an equivalent month-specific risk of COVID-19, who did not have COVID-19 in or before that month, using the electronic health records of the US Department of Veterans Affairs (VA). We also performed a post hoc analysis of mortality risk by subgroups to understand whether COVID-19 disproportionately increased mortality for those who were already most likely to die.

## Methods

This retrospective cohort study was reviewed and approved by the institutional review boards of the Ann Arbor, Durham, Portland, and Puget Sound VA Medical Centers. Informed consent was waived because we used existing data; however, the data were not deidentified. We followed the Strengthening the Reporting of Observational Studies in Epidemiology (STROBE) reporting guideline.

### Study Design and Population

As described in detail elsewhere,^14^ we assembled 14 separate monthly patient cohorts—1 for each month of the initial infection observation period (March 2020-April 2021)—for the purpose of defining index dates and matching covariates. Each month, VA enrollees were included if assigned to a VA primary care team (eg, Patient Aligned Care Team) or had at least 1 VA primary care clinic visit during the 2 years prior. We used the VA COVID-19 Shared Data Resource database to identify Veterans with a documented positive COVID-19 result in a given month, and uninfected potential comparators without this documented result before or in the same month; individuals in both groups met the same inclusion criteria. To avoid misclassification of the first infection date, we excluded Veterans whose records showed a COVID-19 diagnostic code (*International Statistical Classification of Diseases and Related Health Problems, Tenth Revision* codes B97.29, U07.1, U09.9, J12.82, or Z86.16), in a fee-for-service Medicare claim that was dated 15 or more days before the VA test result.

We convened multidisciplinary experts to select appropriate control variables, and we published a peer-reviewed consensus-directed acyclic graph for the relationship between COVID-19 and long-term mortality.^15^ In a 2-step process, infected patients were exact-matched with replacement to uninfected controls based on index month, sex, immunosuppressive medication use (binary), state of residence, and COVID-19 vaccination status (January-April 2021 cohorts only). Then, 37 covariates were included in the month-specific propensity-score model (eTable 2 in Supplement 1).^16^ A caliper of 0.2 times the pooled estimate of the SD of the logit of the propensity score was used to set limits on which uninfected patients could be matched to each infected patient, and up to 5 matched uninfected patients closest in propensity score were retained for each infected patient.^17^ For Veterans with COVID-19, their index date was defined as the date of the earliest positive documentation of COVID-19; and for Veterans who were uninfected, it was the first day of the relevant month.^18,19^ The study flow diagrams with sample construction details are available as eFigure 1 in Supplement 1.

### Statistical Analysis

Covariate balance between the infected and uninfected comparison group was assessed using standardized mean differences (SMDs), where differences of less than 0.1 indicated excellent covariate balance.^20^ The association between COVID-19 infection and all-cause mortality was assessed using Kaplan-Meier estimators. Then, the association was evaluated using a Cox model stratified by matched group coupled with empirical sandwich standard errors to account for matching with replacement. We estimated an average hazard ratio (HR) over the full 2 years. Acknowledging that we expected relative mortality rates to vary over the follow-up period, and hence that the HRs would not remain proportional, we also estimated HRs at 4 clinically meaningful intervals after infection: days 0 to 90, 91 to 180, 181 to 365, and 366 to 730.

For all analyses, individuals were censored at either the end of the study period (April 1, 2022) or at 730 days of follow-up, whichever came first. For all analyses, to maintain comparability between cohorts over time, for a matched group to be included for analysis in that interval, the infected individual and at least 1 uninfected matched comparator must have remained alive and uncensored in that interval.

The primary analysis sought to avoid bias from crossover from the comparators to the infected group, where the acute mortality of later infections among comparators would induce a falsely elevated apparent late mortality in comparators. For the primary analysis, individuals in the comparator group were each censored at first observed infection date if they developed a COVID-19 infection—identified based on either a VA-identified infection or Medicare diagnosis of COVID-19 before the other censoring dates. To reduce potential bias in estimates from censoring, we applied inverse probability of censoring weights^21^ on a monthly basis. We used the same covariates that we used in the matching process, updated monthly, with weights estimated using pooled logistic regression for comparators and all individuals in the COVID-19 infection group having a weight of 1 (*with censoring, weighted* approach).

We also conducted a secondary analysis (*without censoring* approach), in which we did *not* censor comparators at their first-documented infection. In contrast with a *with censoring, weighted* approach, the *without censoring* approach allowed for crossover from the comparator group to the infected group, and for deaths that accrued from such crossover to be included in the mortality of the uninfected comparator group. As a further secondary analysis, we considered an alternative formulation of the primary approach, in which we censored all surviving individuals in a given matched group at the time that the first comparator in that group was observed to develop COVID-19 (*with censoring, unweighted* approach), rather than use inverse probability of censoring weights in the adjusted analyses.

Statistical tests were 2-tailed and *P* values < .05 were considered statistically significant. Data analyses were performed from April 2021 through June 2023 using Stata, release 17.0 (StataCorp LLC) and replicated in SAS, version 9.4 (SAS Institute Inc). All Stata analytic code and the log file are available in Supplement 2. The .do file is available in Supplement 3 and on GitHub (https://github.com/ccmrcodes/CORC24monthMortality).

### Post Hoc Subgroup Analyses

To assess heterogeneity in mortality risk, we conducted post hoc subgroup analyses. We explored whether the unexpected and apparent reduction in later mortality from COVID-19 may have been associated with acceleration of mortality among those who were more likely to die regardless (ie, even in the absence of COVID-19). We divided age into 3 groups on an a priori bases—less than age 65, age 66 to 85, and age greater than 85 years. We divided the pre-COVID-19 Gagne index score, a measure of comorbidity included in the match and measured for all individuals exclusively on pre-COVID-19 information, into approximate tertiles. Lastly, we used all of the variables in eTable 1 in Supplement 1 to perform a prediction analysis for the risk of death in the comparator group—identifying those at greatest risk of death without COVID-19 in the first 90 days after the index date and separately for days 181 to 730. Then, we applied these risk scores from the comparator group to the COVID-19 group, and conducted analyses stratified by tertile of risk. Covariates imbalanced in any subgroup (based on SMD >0.1) were adjusted for all subgroup analyses.

We conducted several additional sensitivity analyses, including (1) subanalyses by calendar time of infection, dividing these into approximately the first 3 waves: March to June 2020, July to November 2020, and December 2020 to April 2021; (2) subanalyses by whether or not a Veteran with COVID-19 was hospitalized in a VA or Medicare facility within 7 days before or after their first-documented evidence of COVID-19; and (3) estimation of a combined HR for days 91 to 730.

In addition, we explored the potential effects that unidentified COVID-19 infections among comparators may have had on our primary analyses. The actual mortality rate of these cases—missed either because a diagnosis was not made, or it was not recorded in the VA or Medicare data—is unknown. In eTable 4 in Supplement 1, we present estimates for how a range of acute mortality rates among comparators with undetected COVID-19 infections in days 366 to 730 would change the number of these undetected cases and how many would be required to meaningfully change the study inferences.

## Results

### Aggregate Rates of Death

We identified 208 061 Veterans (Mean [SD] age, 60.5 [16.2] years; 183 226 [88.1] men and 21 936 [10.5%] women; 1961 [0.9%] American Indian/Alaska Native, 2081 [1.0%] Asian, 47 645 [22.9%] Black, 1944 [0.9%] Native Hawaiian/other Pacific Islander, White 139 604 [67.1%], and 1975 [0.9%] individuals of multiple races) with first-documented positive COVID-19 test results in March 2020 through April 2021 and matched them 1:5 to 1 037 423 comparator Veterans on a monthly basis who did not have a first-documented positive COVID-19 test result during each given month. The demographic characteristics and summary statistics are nearly identical for the 2 groups, as shown in Table 1. The study participant flow diagram is available as eFigure 1 in Supplement 1. The SMDs on matching variables were all less than 0.1 after matching. Among 1 037 423 comparators, 116 181 (11.2%) were observed to develop COVID-19 infections prior to death or the end of follow-up and were censored in the primary analyses. Of the Veterans with COVID-19, 46 902 (22.5%) were hospitalized within 7 days of their index dates; 12 626 (1.2%) comparators were also hospitalized.

**Table 1.** Characteristics and Descriptive Statistics With SMDs for Veterans With COVID-19 and Matched Comparators^a^

Variable	COVID-19 group, No. (%)	Comparator group, No. (%)	SMD
Total participants, No.	208 061	1 037 423	NA
Age, mean (SD), y	60.5 (16.2)	60.5 (16.5)	0.003
BMI, mean (SD)	31.3 (6.3)	31.3 (6.6)	0.010
**Sex**			
Female	21 936 (10.5)	109 085 (10.5)	0.004
Male	183 226 (88.1)	914 361 (88.1)	
Unknown/missing data	2899 (1.4)	13 977 (1.3)	
**Race**			
American Indian/Alaska Native	1961 (0.9)	9578 (0.9)	0.003
Asian	2081 (1.0)	10 536 (1.0)	
Black/African American	47 645 (22.9)	237 896 (22.9)	
Native Hawaiian/other Pacific Islander	1944 (0.9)	9704 (0.9)	
White	139 604 (67.1)	696 309 (67.1)	
Multiple races	1975 (0.9)	9869 (1.0)	
Missing data	12 851 (6.2)	63 531 (6.1)	
**Hispanic ethnicity**			
Yes	20 280 (9.7)	99 093 (9.6)	0.007
No	180 675 (86.8)	903 000 (87.0)	
Missing data	7106 (3.4)	35 330 (3.4)	
**Residence**			
Urban	148 997 (71.6)	742 434 (71.6)	0.001
Not urban (includes missing data)	59 064 (28.4)	294 989 (28.4)	
Distance to nearest VAMC, mile	35.6 (36.5)	35.8 (35.1)	0.004
**Smoking status**			
Current	26 144 (12.6)	131 249 (12.7)	0.012
Former	88 025 (42.3)	439 908 (42.4)	
Never	81 777 (39.3)	408 648 (39.4)	
Missing data	12 115 (5.8)	57 618 (5.6)	
Gagne index score, mean (SD)	1.4 (2.3)	1.4 (2.2)	0.017
**Health care utilization or status during previous 24 mo**			
VA inpatient admissions, mean (SD)	0.4 (1.2)	0.4 (1.3)	0.002
VA primary care visits, mean (SD)	8.6 (9.7)	8.3 (10.4)	0.027
VA specialty care visits, mean (SD)	13.9 (14.1)	13.4 (15.3)	0.030
Mental health care utilizations, mean (SD)	7.9 (22.6)	7.8 (21.9)	0.004
Immunosuppression	20 313 (9.8)	101 256 (9.8)	0.0001
VA nursing home, at index date	2162 (1.0)	9544 (0.9)	0.012
**Nosos risk adjustment category (score)**			
1 (0-0.417)	5694 (2.7)	26 035 (2.5)	0.031
2 (0.417-0.471)	9582 (4.6)	45 530 (4.4)	
3 (0.471-0.534)	12 431 (6.0)	60 994 (5.9)	
4 (0.534-0.611)	15 159 (7.3)	75 933 (7.3)	
5 (0.611-0.707)	17 707 (8.5)	90 104 (8.7)	
6 (0.707-0.829)	20 526 (9.9)	105 586 (10.2)	
7 (0.829-0.998)	23 431 (11.3)	119 913 (11.6)	
8 (0.998-1.259)	26 893 (12.9)	136 532 (13.2)	
9 (1.259-1.805)	31 294 (15.0)	157 063 (15.1)	
10 (≥1.805)	40 555 (19.5)	194 278 (18.7)	
Missing data	4789 (2.3)	25 455 (2.5)	
**CAN category (score)**			
1 (0-20)	34 417 (16.5)	170 471 (16.4)	0.025
2 (25-40)	31 900 (15.3)	160 008 (15.4)	
3 (45-60)	38 219 (18.4)	194 475 (18.7)	
4 (65-80)	46 759 (22.5)	235 676 (22.7)	
5 (85-90)	30 638 (14.7)	153 135 (14.8)	
6 (95-99)	22 072 (10.6)	102 842 (9.9)	
Missing data	4056 (1.9)	20 816 (2.0)	

Abbreviations: BMI, body mass index calculated as weight in kilograms divided by height in meters squared; CAN, Care Assessment Need; NA, not available; SMD, standardized mean difference (smaller numbers indicate better match); VA, US Department of Veterans Affairs; VAMC, Veterans Affairs Medical Center.

^a^ All matching variables are presented in eTable 2 in Supplement 1.

Altogether, the mortality rate by day 730 for the Veterans with documented COVID-19 in our study cohort was 8.7% compared with 4.1% among the matched uninfected comparators (*P* < .001; Figure). This implies an excess of 9625 deaths in the COVID-19 infected group, censoring those comparators who were later observed to develop COVID-19. Consistent patterns for an uncensored sensitivity analysis are shown in eFigure 2 in Supplement 1.

**Figure.**
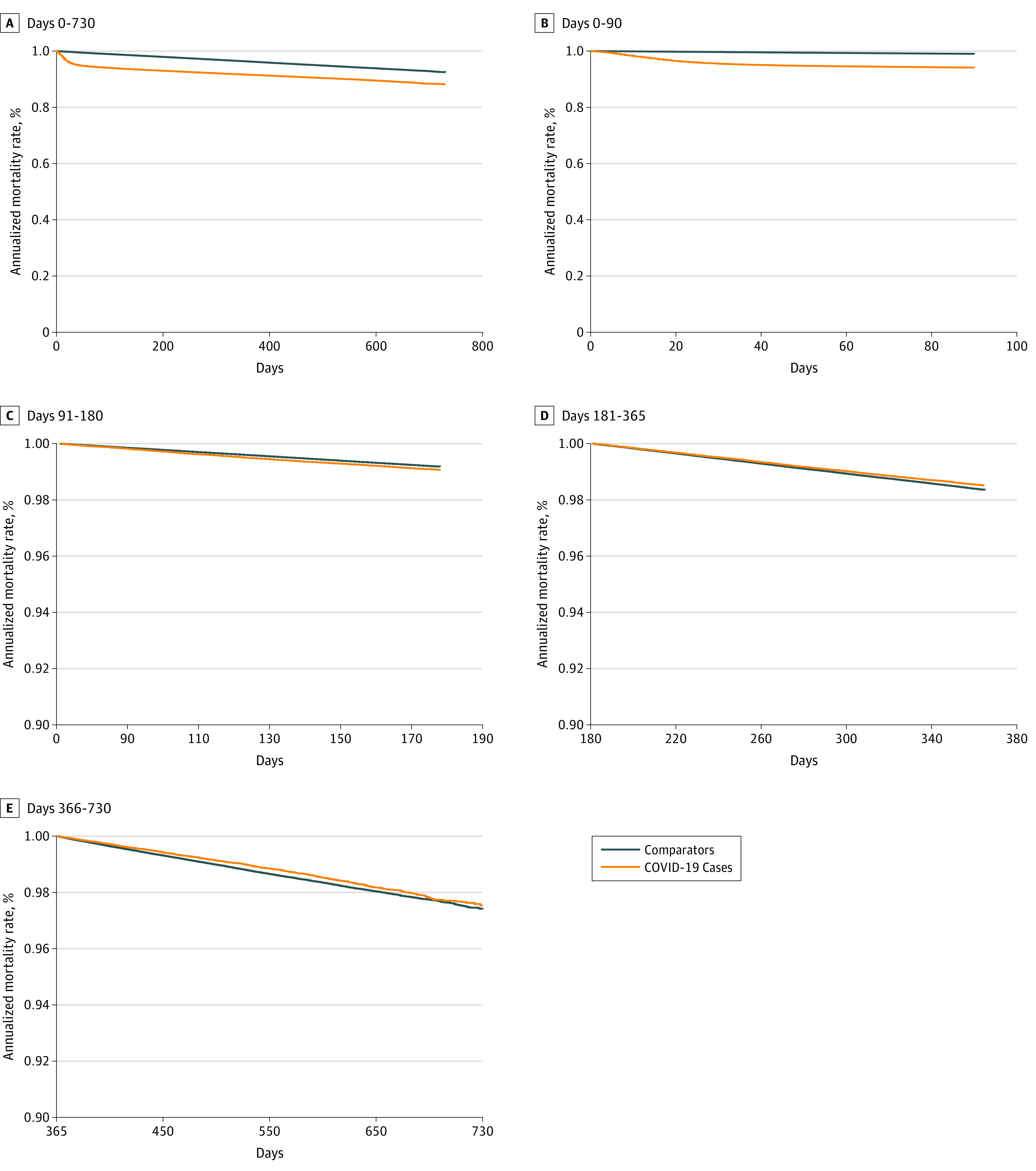
Overall and Time Since Infection-Specific Mortality, From *With Censoring, Unweighted* Approach Figure panels C, D, and E have different y-axis scaling to allow for clearer interpretation.

As shown in the Figure, panels D and E, among matched groups with at least 1 infected and 1 uninfected individual who survived at least 180 days from the index date, there were fewer deaths among the infected than their matched comparators during all subsequent time intervals. The annualized mortality rate by day 365 was 1.37% among Veterans who survived COVID-19 through day 180 in contrast with 1.51% among their comparators in the same interval in primary analyses. Likewise, the mortality rate by day 730 was 0.81% among Veterans who survived COVID-19 through day 365 in contrast with 0.94% among their comparators in the same interval.

### Model-Estimated Results

In all analyses, a greater hazard of death (adjusted HR [aHR], 2.01; 95% CI, 1.98-2.04) was present among those in the documented COVID-19 infection cohort across the entire 2-year analytic period (Table 2). The risk of excess death varied, highest on days 0 to 90 after infection (aHR, 6.36; 95% CI, 6.20-6.51) and declining on days 91 to 180 (aHR, 1.18; 95% CI, 1.12-1.23). Those who survived COVID-19 had lower mortality (greater survival rates) on days 181 to 365 (aHR, 0.92; 95% CI, 0.89-0.95), and 366 to 730 (aHR, 0.89; 95% CI, 0.85- 0.92) than their matched comparators. Regardless of analytic approach taken, there was evidence of lower hazard of death among the originally infected cohort compared with their comparators during all time periods after day 181.

**Table 2.** Cox Regression Results Under Different Approaches to Analyzing Observed COVID-19 Infections Among Comparators

Approach	With censoring, weighted	With censoring, unweighted	Without censoring
Target trial being emulated	As if randomizing to infection or never getting infected	As if randomizing to infection or never getting infected	As if randomizing to infection *or* not at that month
Censoring of comparators	End of administrative follow-up *or* at earliest individual date of infection for comparators	End of administrative follow-up *or* at earliest date of infection among any of the specific set of 5:1 matched comparators for everyone in the set	End of administrative follow-up
Weighting	Inverse probability of censoring	Unweighted	Unweighted
**Hazard ratio of infected Veterans to comparators (95% CI)**			
Overall	2.01 (1.98-2.04)	2.27 (2.23-2.31)	1.75 (1.72-1.77)
Days 0-90	6.36 (6.20-6.51)	6.47 (6.32-6.63)	5.75 (5.62-5.88)
Days 91-180	1.18 (1.12-1.23)	1.16 (1.11-1.22)	1.05 (1.01-1.10)
Days 181-365	0.92 (0.89-0.95)	0.92 (0.88-0.96)	0.81 (0.78-0.83)
Days 366-730	0.89 (0.85-0.92)	0.88 (0.84-0.93)	0.76 (0.73-0.79)

### Post Hoc Subgroup Analyses

A lower risk of death in Veterans who had been infected with COVID-19 compared with their matched comparators, was observed during later time points (after day 180) among low-risk subgroups but not high-risk subgroups (Table 3). This finding opposes what the accelerated death hypothesis would imply. Considering the cases with the highest predicted risk of death on days 1 to 90 or days 181 to 730—the in-cohort c-statistics for these risk scores were 0.85 and 0.83, respectively—in neither case were the reduced later deaths concentrated among the Veterans at greatest risk of death regardless of whether they contracted COVID-19. Consistent patterns, with no substantial risk of excess mortality after day 180, were also seen in results stratified by calendar time of infection (eTable 3 in Supplement 1). Similar results were seen when combining days 91 to 730 to estimate a single HR for the later period: aHR, 0.96 (95% CI, 0.94-0.99). There was no evidence of ongoing excess mortality in the post hoc subgroup of patients who were not hospitalized at the time of their first-documented positive COVID-19 result. In contrast, patients with COVID-19 who had been initially hospitalized died more often during all time periods after infection through day 730 (Table 4). Post hoc sensitivity analyses of how many missed COVID-19 cases among comparators would have been required for equalizing the apparent mortality rates in the Kaplan-Meier curves are presented in eTable 4 in Supplement 1.

**Table 3.** Subgroup Analyses for Time Since Infection With Adjusted Hazard Ratio (aHR) of Patients With COVID-19 to Comparators^a^

Risk subgroup	aHR (95% CI)			
	Age, y	Gagne index score	Predicted risk of death (95% CI)	
			Days 0-90	Days 181-730
Lowest risk	<65 y	−2-0	<0.18%	<1.0%
Days 0-90	4.93 (4.61-5.27)	8.31 (7.81-8.84)	6.39 (5.64-7.24)	5.42 (4.73-6.22)
Days 91-180	1.06 (0.95-1.19)	1.04 (0.92-1.18)	0.96 (0.70-1.30)	0.89 (0.65-1.23)
Days 181-365	0.83 (0.76-0.91)	0.75 (0.67-0.83)	0.71 (0.57-0.89)	0.73 (0.58-0.91)
Days 366-730	0.79 (0.71-0.89)	0.80 (0.71-0.91)	0.81 (0.64-1.04)	0.74 (0.57-0.96)
Middle risk	65-85 y	1-2	0.18%-0.82%	1.0%-4.5%
Days 0-90	7.57 (7.28-7.88)	7.62 (7.23-8.03)	7.75 (7.30-8.22)	7.77 (7.33-8.24)
Days 91-180	1.21 (1.13-1.28)	1.23 (1.13-1.35)	0.94 (0.83-1.07)	0.95 (0.84-1.08)
Days 181-365	0.92 (0.87-0.97)	0.86 (0.80-0.93)	0.72 (0.65-0.79)	0.71 (0.64-0.78)
Days 366-730	0.92 (0.87-0.97)	0.90 (0.83-0.98)	0.69 (0.61-0.77)	0.67 (0.59-0.76)
Highest risk	>85 y	>3	>0.82%	>4.5%
Days 0-90	7.25 (6.53-8.04)	5.94 (5.68-6.21)	6.58 (6.35-6.83)	6.66 (6.42-6.91)
Days 91-180	1.16 (1.02-1.33)	1.24 (1.16-1.33)	1.27 (1.20-1.35)	1.27 (1.20-1.35)
Days 181-365	0.98 (0.88-1.09)	1.06 (1.01-1.12)	1.00 (0.96-1.05)	1.00 (0.96-1.05)
Days 366-730	1.03 (0.91-1.17)	1.00 (0.94-1.07)	1.00 (0.95-1.06)	1.02 (0.96-1.07)

Abbreviations: BMI, body mass index calculated as weight in kilograms divided by height in meters squared; CAN, Care Assessment Need; SMD, standardized mean difference (smaller numbers indicate better match); VAMC, Veterans Affairs Medical Center.

^a^ Each column represents a with-censoring, weighted, set of 3 models, with subgroups defined by characteristics of the patients with COVID-19 within each matched group, adjusted for all imbalanced covariates (SMD >0.1) within any subgroups (age; BMI; ethnicity; smoking status; Gagne index score; number of inpatient admissions in prior 24 mo; number of primary care visits in prior 24 mo; number of specialty care visits in prior 24 mo; NOSOS (categories as shown in Table 1); CAN score (categorical); and distance to nearest VAMC.

**Table 4.** Association of COVID-19-Infection With Subsequent Death, Subanalyzed by Whether the Veteran With COVID-19 Was Acutely Hospitalized^a^

Time period	With censoring, weighted (95% CI)	
	COVID-19, hospitalized	COVID-19, not hospitalized
Overall	4.19 (4.08-4.31)	1.11 (1.08-1.15)
Days 0-90	16.02 (15.24-16.84)	2.60 (2.48-2.72)
Days 91-180	1.84 (1.71-1.97)	0.85 (0.79-0.91)
Days 181-365	1.29 (1.22-1.37)	0.74 (0.70-0.78)
Days 366-730	1.22 (1.14-1.31)	0.76 (0.71-0.81)

^a^ This analysis parallels the first results column of Table 2, separated by whether the Veteran with COVID-19 was hospitalized within 7 days of the documented positive COVID-19 test result or not. Matched groups were preserved within these analyses, which adjust for all imbalanced covariates as in Table 3. These should be considered hypothesis-generating in a target trial emulation framework, as whether COVID-19 leading to acute hospitalization may be part of the causal pathway by which COVID-19 causes adverse mortality. In the language of clinical trials that target trial emulation seeks to follow, hospitalization is a postrandomization variable.

## Discussion

In this national cohorts study of patients with COVID-19, the overall 2-year mortality rate was greater among those who had survived COVID-19 than among the uninfected matched comparators. However, there was no evidence of excess late mortality relative to risk-matched uninfected comparators. In fact, there was evidence of reduced mortality among Veterans who survived COVID-19 by at least 180 days, which was robust to multiple sensitivity analyses and subgroup analyses. This finding did not appear to be explained by observed later infections among comparators or elevated mortality not attributable to COVID-19 among high-risk groups.

The large number of deaths attributable to COVID-19 have been noted on death certificate tabulations and comparisons with historical rates of death.^1,3,4,5,6,7,8,22,23,24^ Prior studies have generally adjusted for demographic information,^10,11,17^ and some have adjusted for a range of more nuanced measures such as socioeconomic status, insurance coverage, comorbid conditions, and long-term care.^9,10,18,19,20,21,22,23,24^ Notably, Al-Aly and colleagues^9^ compared vaccinated VA patients who had breakthrough COVID-19 infections with both unvaccinated and vaccinated controls without infections and found increased mortality from 30 days through 6 months after infection. Their study findings are consistent with our results but did not report later outcomes and used a different matching strategy. However, nearly all of these studies have focused on acute, early, or aggregate mortality, and few have separated the temporal course of mortality differences between patients with COVID-19 and patients at equal risk who might have developed COVID-19; however, consistent findings were noted for up to 1 year in older Italian patients, and in younger but not older Estonians up to 1 year after infection.^7^ These results are similarly compatible with a recent meta-analysis.^25^

We did not see evidence of consistent excess mortality at the population-level or in those not initially hospitalized for COVID-19, as might have been expected given the concerns regarding incident highly morbid new diagnoses among COVID-19 survivors. Our findings do not appear to be entirely consistent with either the so-called “accelerated death” or the “depletion of susceptibles”^26^ hypotheses, although we cannot definitively rule them out. Although our results narrow the range of plausible explanations, there are multiple credible mechanisms still to be evaluated. For example, it is possible that the substantial attention to COVID-19 and its sequelae produced relatively greater access to and/or greater willingness to seek care for COVID-19 survivors, and that this greater attention to their medical needs has been beneficial. Our data cannot distinguish benefits of better access to care among patients with COVID-19 from adverse effects of care disrupted or missed by comparators. Also, it is possible that these COVID-19 infections conveyed resistance to future severe infections, or there were immunologic or other priming mechanisms from the wide-ranging physiologic changes described after COVID-19. These data, only 2 years into the pandemic, are not the final word—ongoing follow-up will be essential.

These results may suggest that for ongoing care after COVID-19, especially among those not initially hospitalized for COVID-19, efforts that focus on increasing survival may be less relevant than approaches addressing the needs of individual survivors. These results also have implications for studies of the sequelae of COVID-19, in which differences in mortality, and thus, differential time at risk must be considered. Our study’s results among those hospitalized for COVID-19 should be considered strictly hypothesis-generating given that hospitalization is analogous to a postrandomization variable in this analytic framework^27^; however, they are consistent with the findings of other published studies of excess mortality among patients hospitalized with sepsis or pneumonia.^10,11^

### Limitations

These results have limitations that must be considered. Only up to 24 months of follow-up had accrued for these participants who became infected in March 2020 through April 2021. Therefore, these findings should not be generalized more recent variants or the more widely available home testing and vaccination options. The potential cumulative effects of COVID-19 will not be fully seen for years or decades to come—these data suggest ongoing differences for which follow-up is needed. These results were obtained within the Veterans Health Administration, which provides comparatively broad access to care and follow-up; results among patients without regular access to care may be different. We analyzed the sensitivity of our results to COVID-19 infections known to VA or Medicare. Yet asymptomatic, untested, or unreported cases occur with increasing frequency; therefore, misclassification, even if it is nondifferential, may have biased these results.^28^ Analyses of potential mediators, such as the role of post−COVID-19 condition, are beyond our scope but are needed. Our presentation of results by hospitalization status should be considered only hypothesis-generating and a first step.^16,27^ Other potential mediators include the development of symptoms of post−COVID-19 condition and subsequent care and illness among both the COVID-19-infected and their comparators. Finally, the so-called hazards of hazard rates for interpreting causal effects have been described,^28^ and although we focus on within-match comparisons and use inverse probability of censoring weighting, unobserved confounding cannot be ruled out.

## Conclusions

The findings of this retrospective cohort study indicate that COVID-19 was not associated with any clinically significant excess mortality among those who survived at least 180 days compared with closely risk-matched comparators, despite having worse 2-year total mortality. This finding has individual level and health system planning implications and should be reassuring to persons who have survived COVID-19 for at least 180 days. To the unknown extent that the target trial emulation strategy and extensive controls successfully balanced confounders, our findings may support causal interpretations that warrant biologic and health system evaluation to optimize care for patients in the aftermath of several years of widespread COVID-19 infection.

## Notes

Supplement 1. **eTable 1.** Target Trial Emulation Comparison Table **eTable 2.** Variables Included in Propensity Score **eTable 3.** Replication of Primary Analysis (paralleling first column of Table 2, separately analyzed by dates of COVID-19 infection) **eTable 4. **Number of Unmeasured COVID-19 Cases and Their Case Fatality Rate **eFigure 1.** Study Flow Diagram for Overall Cohort **eFigure 2. **Without Censoring Kaplan-Meier Curves

Supplement 2.
Stata code .log file

Supplement 3.
Stata code .do file

Supplement 4. **Nonauthor Collaborators.** The VA HSD&R Collaboratory nonauthor collaborators

Supplement 5.
Data Sharing Statement
