# Supplementary material for: Late Mortality After COVID-19 Infection Among US Veterans vs Risk-Matched Comparators: A 2-Year Cohort Analysis
Source: JAMA Intern Med. 2023 Aug 21;183(10):1111–9. doi: 10.1001/jamainternmed.2023.3587 (PMC10442778; doi:10.1001/jamainternmed.2023.3587)
Supplement: Supplement 1. — eTable 1. Target Trial Emulation Comparison Table eTable 2. Variables Included in Propensity Score eTable 3. Replication of Primary Analysis (paralleling first column of Table 2, separately analyzed by dates of COVID-19 infection) eTable 4. Number of Unmeasured COVID-19 Cases and Their Case Fatality Rate eFigure 1. Study Flow Diagram for Overall Cohort eFigure 2. Without Censoring Kaplan-Meier Curves [file jamainternmed-e233587-s001.pdf]

Supplemental Online Content

Iwashyna , Seelye S, Berkowitz TS, et al; for the VA HSR&D COVID-19 Observational Research Collaboratory. Late mortality after COVID-19 infection among US Veterans vs risk-matched comparators: a 2-year cohort analysis. *JAMA Intern Med.* Published online August 21, 2023. doi:10.1001/jamainternmed.2023.3587

eTable 1. Target Trial Emulation Comparison Table

eTable 2. Variables Included in Propensity Score

eTable 3. Replication of Primary Analysis (paralleling first column of Table 2, separately analyzed by dates of COVID-19 infection)

eTable 4. Number of Unmeasured COVID-19 Cases and Their Case Fatality Rate

eFigure 1. Study Flow Diagram for Overall Cohort

eFigure 2. Without Censoring Kaplan-Meier Curves

This supplemental material has been provided by the authors to give readers additional information about their work.

© 2023 Iwashyna TJ et al. *JAMA Intern Med.*

eTable 1: Target Trial Emulation Comparison Table.

	Unethical Target Trial	Emulation
**Goal**	To test the effect of individual infection with SARS-CoV-2 on subsequent death up to 2 years	Same
**Setting**	VA nationwide system	Same
**Inclusion Criteria**	Veterans aged 18 and above in care in the VHA with an assigned primary care team for at least two years on randomization date, or who had at least one VHA primary care clinic visit in that period	Same
**Exclusion Criteria**	Previous COVID-19 Infection<br><br>Address outside of DC or 50 States	Previous documented SARS-CoV-2 Infection in National Surveillance tool or Medicare-documented COVID-19 diagnosis or related diagnostic codes (ICD-10: B97.29, U07.1, U09.9, J12.82, 179 Z86.16) listed in fee-for-service Medicare claims<br><br>Address outside of DC or 50 States<br><br>Missing or invalid key matching variables: age, height, weight, ZIP code<br><br>No suitable matches between infected patients and comparator
**Enrollment Period**	March, 2020—April, 2021	Same
**“Treatment” Strategies**	Inoculum of SARS-CoV-2 sufficient to guarantee COVID-19 infection	SARS-CoV-2 Infection with a confirmatory PCR test for SARS-CoV-2 in VA National Surveillance Tool
**Comparator**	Double-blinded inoculum of placebo	Best matched Veteran with neither documented SARS-CoV-2 Infection in National Surveillance tool nor Medicare-documented COVID-19 diagnosis through the month at which matched as a comparator

© 2023 Iwashyna TJ et al. *JAMA Intern Med.*

**Approach to balancing confounders**	1:1 Randomization	Up to 5:1 (Comparator:Infected) matching on 5 exact criteria and 39 propensity score criteria from VA data
**Primary Outcome and Follow-up**	Mortality up to 2 years, analyzed overall and by period days 1-90, 91-180, 181-365, and 266-730	same
**Follow-up Period**	2 years from inoculation	2 years from the earliest date of a documented positive test for those with COVID-19 infection; comparators began surveillance for outcomes from the same date (“index date”, the emulated equivalent of “randomization and inoculation date”) as that of their individually matched infected patient and were followed for 2 years
**Causal Contrast**	Primary Analysis: Per-Protocol censoring if comparator develops COVID-19<br><br>Sensitivity analysis: Intention-to-Treat (ITT) without censoring for cross-over from developing later COVID-19 in comparators	Primary Analysis: With censoring and weighting, as the observational analog of the per-protocol effect, given the 5:1 match rather than 1:1 in the Target Trial, using inverse probability of censoring weighting.<br><br>Sensitivity Analyses: With censoring, unweighted analysis which excludes the matched strata that included the comparator with an infection at the moment of first infection in any matched comparator; and without censoring for later COVID-19 in comparators
**Statistical analysis**	Cox proportional hazard model to estimate the time to event. Separate analyses for subgroups.	Same

Footnotes: Abbreviations: VHA Veterans Health Administration; SARS-CoV-2 Severe acute respiratory syndrome coronavirus version 2; PCR polymerase chain reaction; COVID-19 Coronavirus disease 2019; ICD-10 International classification of disease tenth edition clinical modification; CAN Care Assessment Need Score; ITT Intent to treat; PP Per protocol

© 2023 Iwashyna TJ et al. *JAMA Intern Med.*

eTable 2: Variables included in propensity score.

Construction of all variables and matches have been previously described at https://bmcmedresmethodol.biomedcentral.com/articles/10.1186/s12874-023-01882-z

Categorical variables included in propensity score:

- immunosuppressive medication use
- nursing home residence any time
- sex
- race/ethnicity
- rurality
- state of residence
- smoking status
- categorization of two comorbidity scores (CAN[12], Nosos[13])
- Indicators for diagnosed CDC high-risk conditions based on ICD-19 codes: coronary heart disease, cancer (excluding non-metastatic skin cancers), chronic kidney disease, congestive heart failure, pulmonary-associated conditions (including asthma, COPD, interstitial lung disease, and cystic fibrosis), dementia, diabetes, hypertension, liver disease, sickle cell/thalassemia, solid organ or blood stem cell transplant, stroke/cerebrovascular disorders, substance use disorder, anxiety disorder, bipolar disorder, major depression, PTSD, and schizophrenia
- Vaccination status (January-April 2021)

Continuous covariates included:

- age
- body mass index (BMI)
- Gagne comorbidity score
- distance from a Veteran's home to nearest VA hospital
- four VA utilization measures (inpatient admissions, primary care visits, specialty care visits, mental health visits in the prior 2 years).

© 2023 Iwashyna TJ et al. *JAMA Intern Med.*

eTable 3: Replication of Primary Analysis, paralleling first column of **Table 2**, separately analyzed by dates of COVID-19 infection. Note that the matched groups are preserved within these analyses.

	With Censoring, Weighted		
Time Period of Infection:	March-June 2020	July-Nov 2020	Dec 2020-April 2021
Hazard Ratio of Infected to Comparators is…			
Overall	2.24 (2.16, 2.32)	1.89 (1.84, 1.94)	2.02 (1.98, 2.07)
Days 0-90	8.16 (7.66, 8.68)	6.18 (5.94, 6.44)	6.02 (5.83, 6.22)
Days 91-180	1.40 (1.25, 1.56)	1.14 (1.05, 1.22)	1.15 (1.08, 1.22)
Days 181-365	1.05 (0.96, 1.15)	0.92 (0.87, 0.98)	0.89 (0.85, 0.93)
Days 366-730	1.01 (0.93, 1.08)	0.84 (0.79, 0.90)	0.86 (0.80, 0.92)

© 2023 Iwashyna TJ et al. *JAMA Intern Med.*

eTable 4a: Number of Unmeasured COVID-19 Cases and Their Case Fatality Rate needed among comparators to account for excess day 366-730 deaths relative to COVID-19 cases under primary analysis assumptions. There were 2,380 deaths by day 730 observed among 133,904 COVID-19+ patients alive at day 366, and 12,678 deaths among 642,663 comparator patients alive at day 366 in the with censoring, unweighted analyses, as represented in Figure 1. Note that overall acute mortality (within first 90 days) was 5.86% for the infected cohort under the with censoring, unweighted analysis.

If acute mortality due to unobserved COVID among comparators is…	Then the number of COVID cases unobserved among comparators necessary to equalize mortality between COVID-19 and comparators is…	The observed number of COVID-19 cases in the comparators during days 366 to 730 was...	Which would imply that the fraction of all COVID-19 cases that were NOT observed was…
0.50%	251,071	57,226	81%
1%	125,535	57,226	69%
5%	25,107	57,226	30%
10%	12,554	57,226	18%

eTable 4b: Number of Unmeasured COVID-19 Cases and Their Without Censoring Case Fatality Rate needed among comparators to account for excess day 366-730 deaths relative to COVID-19 cases under intention-to-treat assumptions. There were 2,517 deaths by day 730 observed among 181,647 COVID-19+ patients alive at day 366, and 15,481 deaths among 867,836 comparator patients alive at day 366 in the per-protocol unweighted analyses, as represented in Figure 1. Note that overall acute mortality (within first 90 days) was 5.97% for the cohort under without censoring analysis.

If acute mortality due to unobserved COVID among comparators is…	Then the number of COVID cases unobserved among comparators necessary to equalize mortality between COVID-19 and comparators is…	The observed number of COVID-19 cases in the comparators during days 366 to 740 was...	Which would imply that the fraction of all COVID-19 cases that were NOT observed was…
0.50%	691,158	57,226	92%
1%	345,579	57,226	86%
5%	69,116	57,226	55%
10%	34,558	57,226	38%

© 2023 Iwashyna TJ et al. *JAMA Intern Med.*

eFigure 1: Study Flow Diagram for Overall Cohort, as previously described at https://bmcmedresmethodol.biomedcentral.com/articles/10.1186/s12874-023-01882-z. The final cohort for the present paper excludes an additional 475 COVID-19 positive Veterans with invalid death dates, for a "final case sample" of 208,061 Veterans. Taking each of these Veterans up to 5 best matched comparators yields a final Comparator sample of 1,037,423.

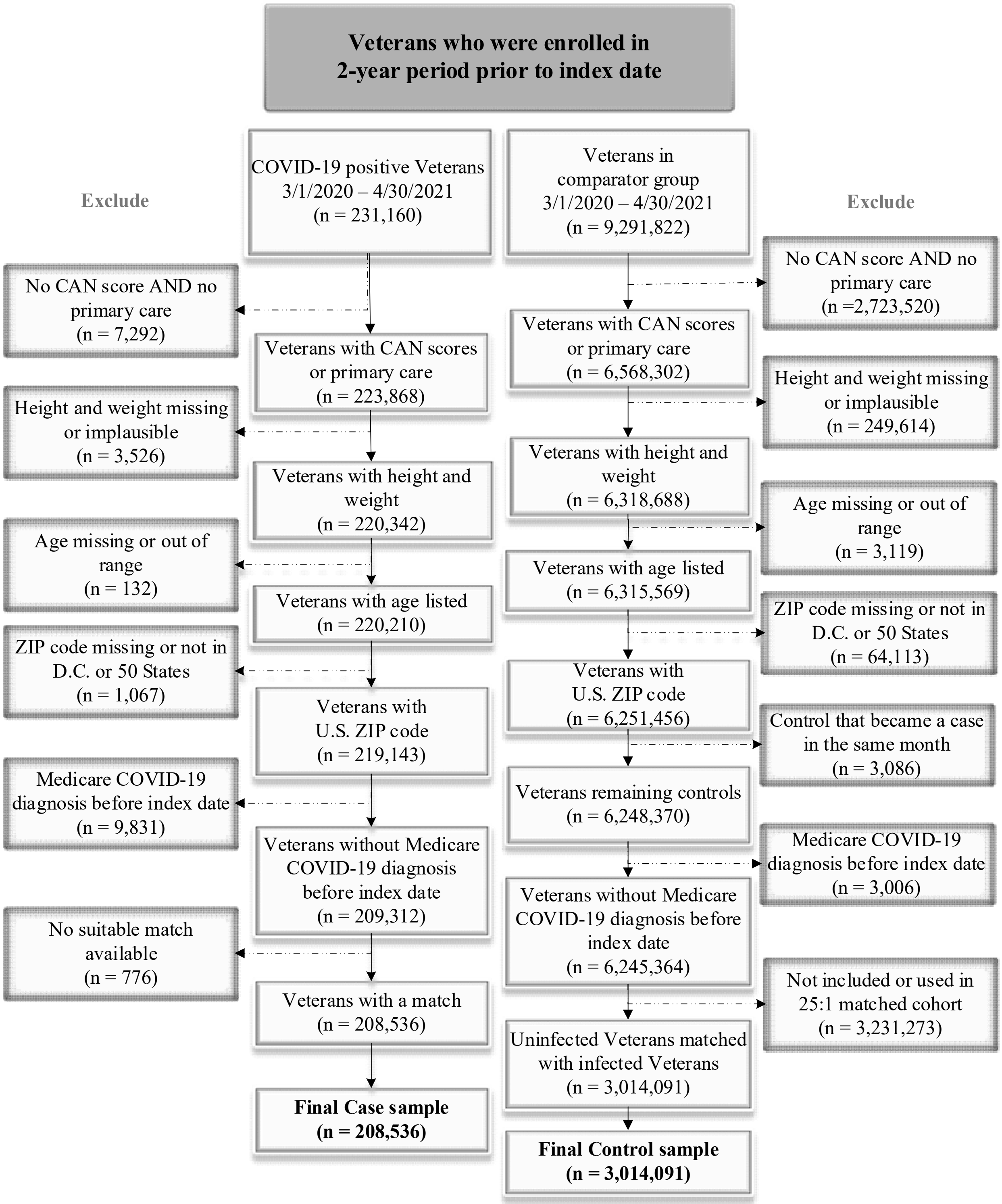

© 2023 Iwashyna TJ et al. *JAMA Intern Med.*

eFigure 2: Without Censoring Kaplan-Meier Curves: From unweighted analyses without censoring of comparators who develop later evidence of COVID-19 infection. Note that Figures A2c, A2d, and A2e have a different axis scaling to allow easier viewing.

eFigure 2a: Days 0-730, without censoring

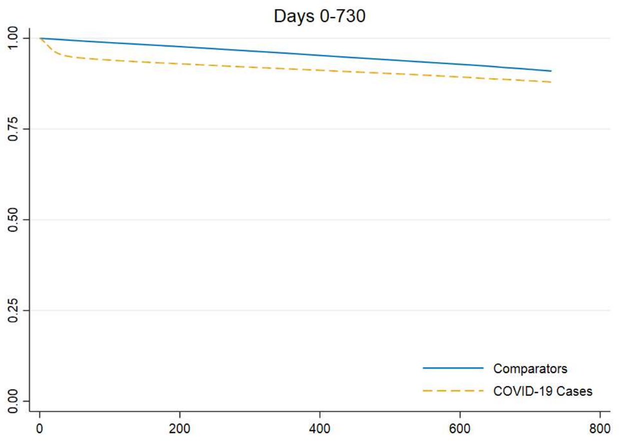

eFigure 2b: Days 0-90, without censoring

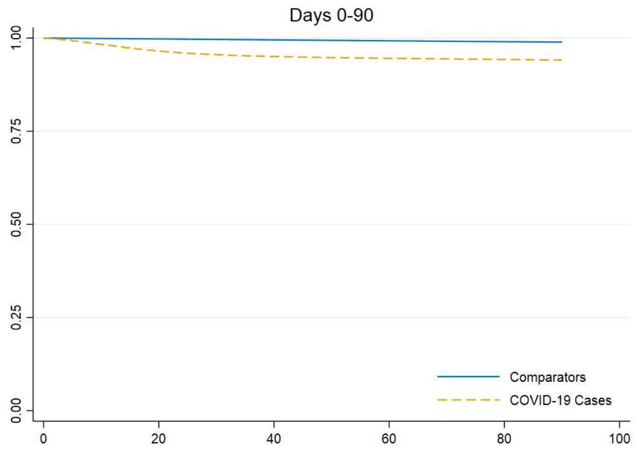

eFigure 2c: Days 91-180, without censoring (Note axis)

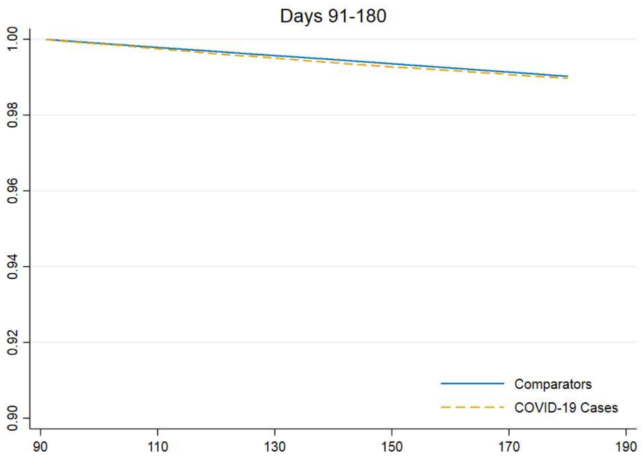

eFigure 2d: Days 181-365, without censoring (Note axis)

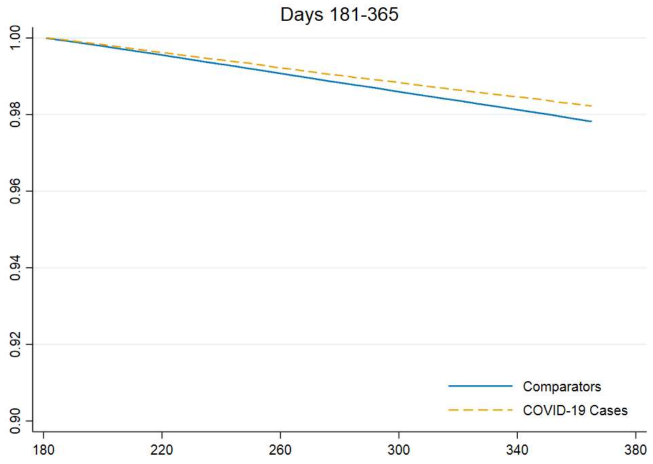

Figure 2e: Days 366-730, without censoring (Note axis)

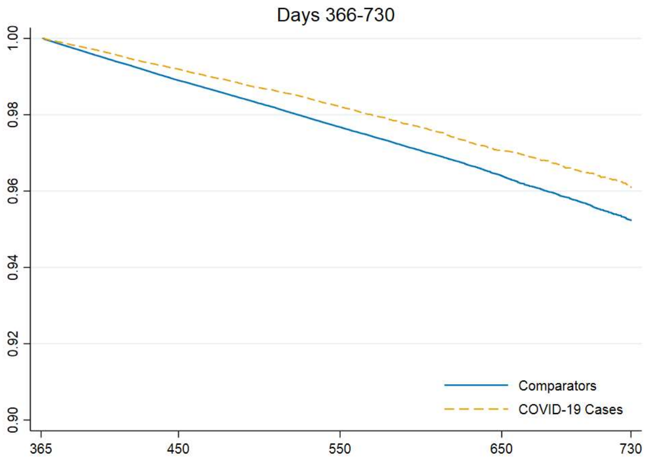

© Iwashyna TJ et al. *JAMA Intern Med.*
