# Supplementary material for: Late Mortality After COVID-19 Infection Among US Veterans vs Risk-Matched Comparators: A 2-Year Cohort Analysis
Source: JAMA Intern Med. 2023 Aug 21;183(10):1111–9. doi: 10.1001/jamainternmed.2023.3587 (PMC10442778; doi:10.1001/jamainternmed.2023.3587)
Supplement: Supplement 2. — Stata code .log file [file jamainternmed-e233587-s002.pdf]

```
------------------------------------------------------------------------
------------------------------------------------------------------------
-----------------------------------------------
      name:  
       log:  ...\LTO Mortality\Logs\analysis_lto_mortality_20230605.log
  log type:  text
 opened on:   5 Jun 2023, 15:46:46

. 
. 
*************************************************************************
*
. * Project:      LTO Mortality in COVID+ patients
. * 
. * Author:       Sarah Seelye
. *
. * Date Created: 2022 May 24
. * Date Updated: 2023 June 5
. 
*************************************************************************
*
. 
. * open death dataset using Portland mortality definitions
. use Data\cohort_dod_allsrc, clear

. 
. * address duplicates
. duplicates report patienticn

Duplicates in terms of patienticn

--------------------------------------
   Copies | Observations       Surplus
----------+---------------------------
        1 |      3147853             0
        2 |          266           133
--------------------------------------

. duplicates report patienticn birthdate

Duplicates in terms of patienticn birthdate

--------------------------------------
   Copies | Observations       Surplus
----------+---------------------------
        1 |      3147853             0
        2 |          266           133
--------------------------------------

.         //all of the duplicates have the same birthdate and 
patienticn.
. duplicates report patienticn scrssn_numeric
```

```
Duplicates in terms of patienticn scrssn_numeric

--------------------------------------
   Copies | Observations       Surplus
----------+---------------------------
        1 |      3148119             0
--------------------------------------

.         //patienticn and SCRSSN uniquely identify patients, but SCRSSN
is not
.         //included in the matchedcohort dataset
. duplicates tag patienticn, gen(dup)

Duplicates in terms of patienticn

. tab dup //266 duplicates; 133 unique Vets

        dup |      Freq.     Percent        Cum.
------------+-----------------------------------
          0 |  3,147,853       99.99       99.99
          1 |        266        0.01      100.00
------------+-----------------------------------
      Total |  3,148,119      100.00

. bysort patienticn (scrssn): gen patientn = _n

. bysort patienticn: gen dod_notsame = 1 if
best_death_date!=best_death_date[_n-1] & patientn==2
(3,148,105 missing values generated)

. bysort patienticn: egen dod_notsame_pat = max(dod_notsame)
(3,148,091 missing values generated)

. tab dod_notsame_pat patientn

dod_notsam |       patientn
     e_pat |         1          2 |     Total
-----------+----------------------+----------
         1 |        14         14 |        28
-----------+----------------------+----------
     Total |        14         14 |        28


.
. * drop duplicates that have different birth dates
. drop if dup==1 & dod_notsame_pat==1
(28 observations deleted)

.
. * drop duplicate patienticns for second SCRSSN on record
. drop if patientn==2
(119 observations deleted)

.
. * keep variables for merge
```

```
. keep patienticn best_death_date

. destring patienticn, replace
patienticn: all characters numeric; replaced as long

.
. format best_death_date %td

.
. tempfile dod

. save `dod'
file D:\Temp\STATA\VHAANNSeelyS\ST_9a30_000001.tmp saved as .dta format

.
. * merge dod dataset with updated _datalong dataset(version 8/22/2022)
. use Data\matchedcohort_25to1_datalong, clear
(Created in file
'P:\ORD_Ioannou_202104007D\MethodsGroup\Programs\CORC_DataExplor)

. destring patienticn, replace
patienticn: all characters numeric; replaced as long

. format patienticn %12.0g

.
. merge m:1 patienticn using `dod'

    Result                      Number of obs
    -----------------------------------------
    Not matched                            83
        from master                        83  (_merge==1)
        from using                          0  (_merge==2)

    Matched                         5,382,629  (_merge==3)
    -----------------------------------------

. drop _merge

.
. *----------------------------
. * Build 5:1 Matched Cohort
. *----------------------------
.
. * drop comparators who aren't matched to cases
. tab ismatched_case

 case has a |
     match? |      Freq.     Percent        Cum.
------------+-----------------------------------
          0 |        776        0.01        0.01
          1 |  5,381,936       99.99      100.00
------------+-----------------------------------
      Total |  5,382,712      100.00
```

```
. drop if ismatched_case==0
(776 observations deleted)

. count //n=5,381,936
  5,381,936

. tab case //case=208,536

 indicator: |
         is |
 `index_dt` |
the date of |
  the first |
   positive |
   covid-19 |
test result |
        for |      Freq.     Percent        Cum.
------------+-----------------------------------
          0 |  5,173,400       96.13       96.13
          1 |    208,536        3.87      100.00
------------+-----------------------------------
      Total |  5,381,936      100.00

.
. * create a new index_dt variable to use the case's index date for all
controls
. gen index_dt_case = index_dt if case==1
(5,173,400 missing values generated)

. format index_dt_case %td

. gsort matchgroupnumber -case

. by matchgroupnumber: replace index_dt_case = index_dt_case[_n-1] if
index_dt_case[_n-1]!=.
(5173400 real changes made)

.
. * create an indextodeath variable to count number of days between
index date
. * and death
. gen indextodeath_caseindex = best_death_date-index_dt_case
(4,979,036 missing values generated)

. sum indextodeath_caseindex

    Variable |        Obs        Mean    Std. dev.       Min        Max
-------------+---------------------------------------------------------
indextodea~x |    402,900    287.2704    304.4486     -28306        881

. sum indextodeath_caseindex if indextodeath_caseindex<0

    Variable |        Obs        Mean    Std. dev.       Min        Max
```

```
-------------+---------------------------------------------------------
indextodea~x |     11,653   -284.7707    1266.695     -28306         -1

.
. * drop indextodeath_caseindex<0 to exclude observations that die
before the
. * index date
. drop if indextodeath_caseindex<0
(11,653 observations deleted)

. tab case //case=208,063

 indicator: |
         is |
 `index_dt` |
the date of |
  the first |
   positive |
   covid-19 |
test result |
        for |      Freq.     Percent        Cum.
------------+-----------------------------------
          0 |  5,162,220       96.13       96.13
          1 |    208,063        3.87      100.00
------------+-----------------------------------
      Total |  5,370,283      100.00

.
. * drop those with an infection date prior to index date
. * create time-to-infection variable
. gen ttinfection = futureinfectedcontrolindexdate - index_dt_case
(4,682,272 missing values generated)

.
. sum ttinfection

    Variable |        Obs        Mean    Std. dev.       Min        Max
-------------+---------------------------------------------------------
 ttinfection |    688,011    338.3773    180.3509       -417        867

. sum ttinfection if ttinfection <0

    Variable |        Obs        Mean    Std. dev.       Min        Max
-------------+---------------------------------------------------------
 ttinfection |      1,411   -61.74557    63.32246       -417         -1

. drop if ttinfection<0 //n=1411
(1,411 observations deleted)

. tab case //case=208,063

 indicator: |
         is |
 `index_dt` |
```

```
the date of |
  the first |
   positive |
   covid-19 |
test result |
        for |      Freq.     Percent        Cum.
------------+-----------------------------------
          0 |  5,160,809       96.12       96.12
          1 |    208,063        3.88      100.00
------------+-----------------------------------
      Total |  5,368,872      100.00

.
. drop ttinfection

.
. * keep the best 5 matches
. gsort matchgroupnumber propensityscoreabsdiffrank -case

. by matchgroupnumber: gen n = _n

. tab n

          n |      Freq.     Percent        Cum.
------------+-----------------------------------
          1 |    208,536        3.88        3.88
          2 |    208,533        3.88        7.77
          3 |    208,198        3.88       11.65
          4 |    207,853        3.87       15.52
          5 |    207,664        3.87       19.39
          6 |    207,532        3.87       23.25
          7 |    207,375        3.86       27.11
          8 |    207,263        3.86       30.97
          9 |    207,173        3.86       34.83
         10 |    207,096        3.86       38.69
         11 |    207,040        3.86       42.55
         12 |    206,971        3.86       46.40
         13 |    206,888        3.85       50.25
         14 |    206,801        3.85       54.11
         15 |    206,736        3.85       57.96
         16 |    206,696        3.85       61.81
         17 |    206,645        3.85       65.66
         18 |    206,575        3.85       69.50
         19 |    206,491        3.85       73.35
         20 |    206,423        3.84       77.19
         21 |    206,341        3.84       81.04
         22 |    206,304        3.84       84.88
         23 |    206,258        3.84       88.72
         24 |    206,154        3.84       92.56
         25 |    205,551        3.83       96.39
         26 |    193,775        3.61      100.00
------------+-----------------------------------
      Total |  5,368,872      100.00
```

```
.
. keep if n<=6
(4,120,556 observations deleted)

.
. tab n

          n |      Freq.     Percent        Cum.
------------+-----------------------------------
          1 |    208,536       16.71       16.71
          2 |    208,533       16.71       33.41
          3 |    208,198       16.68       50.09
          4 |    207,853       16.65       66.74
          5 |    207,664       16.64       83.38
          6 |    207,532       16.62      100.00
------------+-----------------------------------
      Total |  1,248,316      100.00

. tab n case

           |     indicator: is
           |  `index_dt` the date
           | of the first positive
           | covid-19 test result
           |          for
         n |         0          1 |     Total
-----------+----------------------+----------
         1 |       473    208,063 |   208,536
         2 |   208,533          0 |   208,533
         3 |   208,198          0 |   208,198
         4 |   207,853          0 |   207,853
         5 |   207,664          0 |   207,664
         6 |   207,532          0 |   207,532
-----------+----------------------+----------
     Total | 1,040,253    208,063 | 1,248,316

.
. bysort matchgroupnumber (n): egen totalpairs = max(n)

. tab totalpairs

 totalpairs |      Freq.     Percent        Cum.
------------+-----------------------------------
          1 |          3        0.00        0.00
          2 |        670        0.05        0.05
          3 |      1,035        0.08        0.14
          4 |        756        0.06        0.20
          5 |        660        0.05        0.25
          6 |  1,245,192       99.75      100.00
------------+-----------------------------------
      Total |  1,248,316      100.00

. tab totalpairs case // 3 patients - 2 cases - have no matched pair
```

```
            |     indicator: is
            |  `index_dt` the date
            | of the first positive
            | covid-19 test result
            |          for
totalpairs |         0          1 |     Total
-----------+----------------------+----------
         1 |         1          2 |         3
         2 |       335        335 |       670
         3 |       691        344 |     1,035
         4 |       567        189 |       756
         5 |       528        132 |       660
         6 | 1,038,131    207,061 | 1,245,192
-----------+----------------------+----------
     Total | 1,040,253    208,063 | 1,248,316

.
. * drop the 3 patients with no matched pair
. drop if totalpairs==1
(3 observations deleted)

. tab case //case=208,061

 indicator: |
         is |
 `index_dt` |
the date of |
  the first |
   positive |
   covid-19 |
test result |
        for |      Freq.     Percent        Cum.
------------+-----------------------------------
          0 |  1,040,252       83.33       83.33
          1 |    208,061       16.67      100.00
------------+-----------------------------------
      Total |  1,248,313      100.00

.
. * confirm that all matched groups have a case
. bysort matchgroupnumber: egen groupwithacase = max(case)

. tab groupwithacase //2,829 comparators don't have a matched case

groupwithac |
        ase |      Freq.     Percent        Cum.
------------+-----------------------------------
          0 |      2,829        0.23        0.23
          1 |  1,245,484       99.77      100.00
------------+-----------------------------------
      Total |  1,248,313      100.00

.
. * drop comparator observations who don't have a matched case
```

```
. drop if groupwithacase==0 //2,829 comparators dropped
(2,829 observations deleted)

.
. * confirm that each case does not have more than 5 controls
. gen control = case==0

. by matchgroupnumber: egen totalcontrols = sum(control)

. tab totalcontrols if case==1

totalcontro |
         ls |      Freq.     Percent        Cum.
------------+-----------------------------------
          1 |        335        0.16        0.16
          2 |        344        0.17        0.33
          3 |        189        0.09        0.42
          4 |        132        0.06        0.48
          5 |    207,061       99.52      100.00
------------+-----------------------------------
      Total |    208,061      100.00

.
. * count
. tab case //n=1,245,484, of which 208,061 are cases

 indicator: |
         is |
 `index_dt` |
the date of |
  the first |
   positive |
   covid-19 |
test result |
        for |      Freq.     Percent        Cum.
------------+-----------------------------------
          0 |  1,037,423       83.29       83.29
          1 |    208,061       16.71      100.00
------------+-----------------------------------
      Total |  1,245,484      100.00

.
. * drop variables no longer needed
. drop n totalpairs groupwithacase control totalcontrols

.
. * merge with covid+ hospitalization dataset
. merge 1:1 matchgroupnumber patienticn using
Data\Hospitalizations\lto_mortality_hospitalizations_20230516

    Result                           Number of obs
    -----------------------------------------
    Not matched                         1,237,206
        from master                     1,185,956  (_merge==1)
```

```
        from using                        51,250  (_merge==2)

    Matched                               59,528  (_merge==3)
    -----------------------------------------

. drop if _merge==2
(51,250 observations deleted)

. drop _merge

. count //1,245,484
  1,245,484

.
. recode index_hospitalization (.=0)
(1,185,956 changes made to index_hospitalization)

.
. * identify matchgroups in which the covid+ case was hospitalized/not
hospitalized
. gen case_hospitalized=index_hospitalization==1 if case==1
(1,037,423 missing values generated)

. bysort matchgroupnumber: egen case_hospitalized_matchgrp =
max(case_hospitalized)

.
. drop case_hospitalized

.
. *-------------------------
. * Construct Covariates
. *-------------------------
.
. * create a new unique patient id variable that combines patienticn &
matchgroupnumber
. tostring patienticn, gen(patienticn_str)
patienticn_str generated as str10

. tostring matchgroupnumber, gen(matchgroup_str)
matchgroup_str generated as str6

.
. gen uniq_patid_str = patienticn_str + matchgroup_str

. destring uniq_patid_str, gen(uniq_patid)
uniq_patid_str: all characters numeric; uniq_patid generated as double

. format uniq_patid %14.0g

.
. drop patienticn_str matchgroup_str uniq_patid_str

. duplicates report uniq_patid
```

```
Duplicates in terms of uniq_patid

--------------------------------------
   Copies | Observations       Surplus
----------+---------------------------
        1 |      1245484             0
--------------------------------------

.
. * wave of infection
.         * March-June 2020
.         * July-Nov 2020
.         * Dec 2020-Apr 2021
.
. tab index_month

  number of |
     months |
    *after* |
 march 2020 |
   when the |
 index date |
    occurs, |
      i.e., |
 0=march 20 |      Freq.     Percent        Cum.
------------+-----------------------------------
          0 |     13,998        1.12        1.12
          1 |     40,162        3.22        4.35
          2 |     26,037        2.09        6.44
          3 |     41,683        3.35        9.79
          4 |     89,471        7.18       16.97
          5 |     50,101        4.02       20.99
          6 |     40,590        3.26       24.25
          7 |     72,930        5.86       30.11
          8 |    185,021       14.86       44.96
          9 |    261,384       20.99       65.95
         10 |    216,893       17.41       83.36
         11 |     93,827        7.53       90.90
         12 |     59,322        4.76       95.66
         13 |     54,065        4.34      100.00
------------+-----------------------------------
      Total |  1,245,484      100.00

.
. gen wave = .
(1,245,484 missing values generated)

. replace wave = 1 if inrange(index_month, 0, 3)
(121,880 real changes made)

. replace wave = 2 if inrange(index_month, 4, 8)
(438,113 real changes made)
```

```
. replace wave = 3 if inrange(index_month, 9, 13)
(685,491 real changes made)

. tab index_month wave

 number of |
    months |
   *after* |
march 2020 |
  when the |
index date |
   occurs, |
     i.e., |               wave
0=march 20 |         1          2          3 |     Total
-----------+---------------------------------+----------
         0 |    13,998          0          0 |    13,998 
         1 |    40,162          0          0 |    40,162 
         2 |    26,037          0          0 |    26,037 
         3 |    41,683          0          0 |    41,683 
         4 |         0     89,471          0 |    89,471 
         5 |         0     50,101          0 |    50,101 
         6 |         0     40,590          0 |    40,590 
         7 |         0     72,930          0 |    72,930 
         8 |         0    185,021          0 |   185,021 
         9 |         0          0    261,384 |   261,384 
        10 |         0          0    216,893 |   216,893 
        11 |         0          0     93,827 |    93,827 
        12 |         0          0     59,322 |    59,322 
        13 |         0          0     54,065 |    54,065 
-----------+---------------------------------+----------
     Total |   121,880    438,113    685,491 | 1,245,484 

. 
. * age categories
. sum ageatindexdate

    Variable |        Obs        Mean    Std. dev.       Min        Max
-------------+---------------------------------------------------------
ageatinde~te |  1,245,484    60.47358    16.41887   18.88493   99.99726

. 
. gen agecat = .
(1,245,484 missing values generated)

. replace agecat=1 if ageatindexdate<65
(670,258 real changes made)

. replace agecat=2 if inrange(ageatindexdate, 65, 85)
(513,602 real changes made)

. replace agecat=3 if ageatindexdate>85
(61,624 real changes made)

. 
```

```
. * create indicator for COVID+ cases
. gen infected = .
(1,245,484 missing values generated)

. replace infected = 1 if case==1
(208,061 real changes made)

. replace infected = 0 if case==0
(1,037,423 real changes made)

.
. * label values of categorical variables
. tab sex3cat infected

    sex of |
   veteran |       infected
 (3-level) |         0          1 |     Total
-----------+----------------------+----------
         0 |    13,977      2,899 |    16,876
         1 |   109,085     21,936 |   131,021
         2 |   914,361    183,226 | 1,097,587
-----------+----------------------+----------
     Total | 1,037,423    208,061 | 1,245,484

. recode sex3cat 0=99
(16,876 changes made to sex3cat)

. lab def sex3cat 99 "Unknown" 1 "Female" 2 "Male", replace

. lab val sex3cat sex3cat

. tab sex3cat infected

    sex of |
   veteran |       infected
 (3-level) |         0          1 |     Total
-----------+----------------------+----------
    Female |   109,085     21,936 |   131,021
      Male |   914,361    183,226 | 1,097,587
   Unknown |    13,977      2,899 |    16,876
-----------+----------------------+----------
     Total | 1,037,423    208,061 | 1,245,484

.
. tab race7cat infected

   race of |
   veteran |       infected
 (7-level) |         0          1 |     Total
-----------+----------------------+----------
         0 |    63,531     12,851 |    76,382
         1 |     9,578      1,961 |    11,539
         2 |    10,536      2,081 |    12,617
         3 |   237,896     47,645 |   285,541
```

```
         4 |     9,704      1,944 |    11,648 
         5 |   696,309    139,604 |   835,913 
         6 |     9,869      1,975 |    11,844 
-----------+----------------------+----------
     Total | 1,037,423    208,061 | 1,245,484 

. recode race7cat 0=99
(76,382 changes made to race7cat)

. lab def race7cat 1 "AmericanIndian/AlaskaNative" 2 "Asian" 3 
"Black/AfricanAmer" ///
>                                  4 "NativeHawaiian/PacificIsland" 5 
"White" 6 "MultipleRace"     ///
>                                  99 "Missing" , replace

. lab val race7cat race7cat

. tab race7cat infected

      race of veteran |       infected
            (7-level) |         0          1 |     Total
----------------------+----------------------+----------
AmericanIndian/Alaska |     9,578      1,961 |    11,539 
                Asian |    10,536      2,081 |    12,617 
    Black/AfricanAmer |   237,896     47,645 |   285,541 
NativeHawaiian/Pacifi |     9,704      1,944 |    11,648 
                White |   696,309    139,604 |   835,913 
         MultipleRace |     9,869      1,975 |    11,844 
              Missing |    63,531     12,851 |    76,382 
----------------------+----------------------+----------
                Total | 1,037,423    208,061 | 1,245,484 

. 
. tab ethnicity3cat infected

 ethnicity |
of veteran |       infected
 (3-level) |         0          1 |     Total
-----------+----------------------+----------
         0 |    35,330      7,106 |    42,436 
         1 |    99,093     20,280 |   119,373 
         2 |   903,000    180,675 | 1,083,675 
-----------+----------------------+----------
     Total | 1,037,423    208,061 | 1,245,484 

. recode ethnicity3cat 0=99
(42,436 changes made to ethnicity3cat)

. lab def ethnicity3cat 1 "Hispanic" 2 "NotHispanic" 99 "Missing", 
replace

. lab val ethnicity3cat ethnicity3cat

. tab ethnicity3cat infected, nol
```

```
  ethnicity |
 of veteran |       infected
  (3-level) |         0          1 |     Total
------------+----------------------+----------
          1 |    99,093     20,280 |   119,373 
          2 |   903,000    180,675 | 1,083,675 
         99 |    35,330      7,106 |    42,436 
------------+----------------------+----------
      Total | 1,037,423    208,061 | 1,245,484 

. 
. tab rurality2cat infected

   rurality |
         of |
 veteran's |
   home zip |
       code |       infected
  (2-level) |         0          1 |     Total
------------+----------------------+----------
          0 |   294,989     59,064 |   354,053 
          1 |   742,434    148,997 |   891,431 
------------+----------------------+----------
      Total | 1,037,423    208,061 | 1,245,484 

. lab def rurality2cat 0 "NotUrban" 1 "Urban", replace

. lab val rurality2cat rurality2cat

. tab rurality2cat infected

   rurality |
         of |
 veteran's |
   home zip |
       code |       infected
  (2-level) |         0          1 |     Total
------------+----------------------+----------
   NotUrban |   294,989     59,064 |   354,053 
      Urban |   742,434    148,997 |   891,431 
------------+----------------------+----------
      Total | 1,037,423    208,061 | 1,245,484 

. 
. tab smoking4cat infected

    smoking |
  status of |
    veteran |       infected
  (4-level) |         0          1 |     Total
------------+----------------------+----------
          0 |    57,618     12,115 |    69,733 
          1 |   131,249     26,144 |   157,393 
```

```
         2 |   439,908     88,025 |   527,933 
         3 |   408,648     81,777 |   490,425 
-----------+----------------------+----------
     Total | 1,037,423    208,061 | 1,245,484 

. recode smoking4cat 0=99
(69,733 changes made to smoking4cat)

. lab def smoking4cat 1 "Current" 2 "Former" 3 "Never" 99 "Missing", 
replace

. lab val smoking4cat smoking4cat

. tab smoking4cat infected

   smoking |
 status of |
   veteran |       infected
 (4-level) |         0          1 |     Total
-----------+----------------------+----------
   Current |   131,249     26,144 |   157,393 
    Former |   439,908     88,025 |   527,933 
     Never |   408,648     81,777 |   490,425 
   Missing |    57,618     12,115 |    69,733 
-----------+----------------------+----------
     Total | 1,037,423    208,061 | 1,245,484 

. 
. tab immuno infected

immunosupp |       infected
   ressed? |         0          1 |     Total
-----------+----------------------+----------
         0 |   936,167    187,748 | 1,123,915 
         1 |   101,256     20,313 |   121,569 
-----------+----------------------+----------
     Total | 1,037,423    208,061 | 1,245,484 

. tab clcatindexdate infected

  clc care |
  received |
 before or |
  on index |       infected
      date |         0          1 |     Total
-----------+----------------------+----------
         0 | 1,027,879    205,899 | 1,233,778 
         1 |     9,544      2,162 |    11,706 
-----------+----------------------+----------
     Total | 1,037,423    208,061 | 1,245,484 

. 
. tab nosos11cat infected
```

```
      nosos |       infected
(11-level) |         0          1 |     Total
-----------+----------------------+----------
         0 |    25,455      4,789 |    30,244
         1 |    26,035      5,694 |    31,729
         2 |    45,530      9,582 |    55,112
         3 |    60,994     12,431 |    73,425
         4 |    75,933     15,159 |    91,092
         5 |    90,104     17,707 |   107,811
         6 |   105,586     20,526 |   126,112
         7 |   119,913     23,431 |   143,344
         8 |   136,532     26,893 |   163,425
         9 |   157,063     31,294 |   188,357
        10 |   194,278     40,555 |   234,833
-----------+----------------------+----------
     Total | 1,037,423    208,061 | 1,245,484

. recode nosos11cat 0=99
(30,244 changes made to nosos11cat)

. tab nosos11cat infected

     nosos |       infected
(11-level) |         0          1 |     Total
-----------+----------------------+----------
         1 |    26,035      5,694 |    31,729
         2 |    45,530      9,582 |    55,112
         3 |    60,994     12,431 |    73,425
         4 |    75,933     15,159 |    91,092
         5 |    90,104     17,707 |   107,811
         6 |   105,586     20,526 |   126,112
         7 |   119,913     23,431 |   143,344
         8 |   136,532     26,893 |   163,425
         9 |   157,063     31,294 |   188,357
        10 |   194,278     40,555 |   234,833
        99 |    25,455      4,789 |    30,244
-----------+----------------------+----------
     Total | 1,037,423    208,061 | 1,245,484

.
. tab canscore7cat infected

 can score |       infected
 (7-level) |         0          1 |     Total
-----------+----------------------+----------
         0 |    20,816      4,056 |    24,872
         1 |   170,471     34,417 |   204,888
         2 |   160,008     31,900 |   191,908
         3 |   194,475     38,219 |   232,694
         4 |   235,676     46,759 |   282,435
         5 |   153,135     30,638 |   183,773
         6 |   102,842     22,072 |   124,914
-----------+----------------------+----------
     Total | 1,037,423    208,061 | 1,245,484
```

```
. recode canscore7cat 0=99
(24,872 changes made to canscore7cat)

. tab canscore7cat infected

 can score |       infected
 (7-level) |         0          1 |     Total
-----------+----------------------+----------
         1 |   170,471     34,417 |   204,888
         2 |   160,008     31,900 |   191,908
         3 |   194,475     38,219 |   232,694
         4 |   235,676     46,759 |   282,435
         5 |   153,135     30,638 |   183,773
         6 |   102,842     22,072 |   124,914
        99 |    20,816      4,056 |    24,872
-----------+----------------------+----------
     Total | 1,037,423    208,061 | 1,245,484

.
. *-------------------------
. * Time to Study End
. *-------------------------
.
. * identify study end as April 1, 2022
. gen studyend = mdy(4, 1, 2022)

. format studyend %td

.
. * count days from index to studyend
. gen ttstudyend = studyend-index_dt_case

.
. *----------------------
. * Mortality Variables
. *----------------------
.
. * Alive on day 91
. gen alive_day91 = .
(1,245,484 missing values generated)

. replace alive_day91 = 1 if indextodeath_caseindex>=91
(1,221,670 real changes made)

. replace alive_day91 = 0 if inrange(indextodeath_caseindex, 0, 90)
(23,814 real changes made)

. replace alive_day91 = 0 if ttstudyend<91
(0 real changes made)

. bysort alive_day91: sum indextodeath_caseindex

------------------------------------------------------------------------------
```

```
--------------------------------------------------------------------------
------------------------------------------------
-> alive_day91 = 0

    Variable |        Obs        Mean    Std. dev.       Min        Max
-------------+---------------------------------------------------------
indextodea~x |     23,814    32.98224     25.4598          0         90

--------------------------------------------------------------------------
--------------------------------------------------------------------------
------------------------------------------------
-> alive_day91 = 1

    Variable |        Obs        Mean    Std. dev.       Min        Max
-------------+---------------------------------------------------------
indextodea~x |     72,781    360.3306    166.2994         91        881


.
. * Alive on day 181
. gen alive_day181 = .
(1,245,484 missing values generated)

. replace alive_day181 = 1 if indextodeath_caseindex>=181
(1,209,311 real changes made)

. replace alive_day181 = 0 if inrange(indextodeath_caseindex, 0, 180)
(36,173 real changes made)

. replace alive_day181 = 0 if ttstudyend<181
(0 real changes made)

. bysort alive_day181: sum indextodeath_caseindex

--------------------------------------------------------------------------
--------------------------------------------------------------------------
------------------------------------------------
-> alive_day181 = 0

    Variable |        Obs        Mean    Std. dev.       Min        Max
-------------+---------------------------------------------------------
indextodea~x |     36,173    67.94139    54.91375          0        180

--------------------------------------------------------------------------
--------------------------------------------------------------------------
------------------------------------------------
-> alive_day181 = 1

    Variable |        Obs        Mean    Std. dev.       Min        Max
-------------+---------------------------------------------------------
indextodea~x |     60,422    406.3589    143.8607        181        881


.
```

```
. * Alive on day 366
. gen alive_day366 = .
(1,245,484 missing values generated)

. replace alive_day366 = 1 if indextodeath_caseindex>=366
(1,183,181 real changes made)

. replace alive_day366 = 0 if inrange(indextodeath_caseindex, 0, 365)
(62,303 real changes made)

. replace alive_day366 = 0 if ttstudyend<366
(52,071 real changes made)

. bysort alive_day366: sum indextodeath_caseindex

------------------------------------------------------------------------
------------------------------------------------------------------------
-----------------------------------------------
-> alive_day366 = 0

    Variable |        Obs        Mean    Std. dev.       Min        Max
-------------+---------------------------------------------------------
indextodea~x |     62,734    156.0614    116.8082          0        498

------------------------------------------------------------------------
------------------------------------------------------------------------
-----------------------------------------------
-> alive_day366 = 1

    Variable |        Obs        Mean    Std. dev.       Min        Max
-------------+---------------------------------------------------------
indextodea~x |     33,861    508.5585    103.4674        366        881


.
. * Died Index Day-Day 90
. gen died_0_90 = .
(1,245,484 missing values generated)

. replace died_0_90 = 1 if inrange(indextodeath_caseindex, 0, 90)
(23,814 real changes made)

. replace died_0_90 = 0 if indextodeath_caseindex>90
(1,221,670 real changes made)

. tab died_0_90, m

  died_0_90 |      Freq.     Percent        Cum.
------------+-----------------------------------
          0 |  1,221,670       98.09       98.09
          1 |     23,814        1.91      100.00
------------+-----------------------------------
      Total |  1,245,484      100.00
```

```
.
. * Died Day 91-Day 180
. gen died_91_180 = .
(1,245,484 missing values generated)

. replace died_91_180 = 1 if inrange(indextodeath_caseindex, 91, 180)
(12,359 real changes made)

. replace died_91_180 = 0 if indextodeath_caseindex>180
(1,209,311 real changes made)

. replace died_91_180 = 0 if (indextodeath_caseindex>ttstudyend) &
(inrange(indextodeath_caseindex, 91, 180)) ///
>
& (inrange(ttstudyend, 91, 180))
(0 real changes made)

. tab died_91_180, m

died_91_180 |      Freq.     Percent        Cum.
------------+-----------------------------------
          0 |  1,209,311       97.10       97.10
          1 |     12,359        0.99       98.09
          . |     23,814        1.91      100.00
------------+-----------------------------------
      Total |  1,245,484      100.00

. sum indextodeath_caseindex if indextodeath_caseindex<91

    Variable |        Obs        Mean    Std. dev.       Min        Max
-------------+---------------------------------------------------------
indextodea~x |     23,814    32.98224     25.4598          0         90

.
. * Died Day 181-Day 365
. gen died_181_365 = .
(1,245,484 missing values generated)

. replace died_181_365 = 1 if inrange(indextodeath_caseindex, 181, 365)
(26,130 real changes made)

. replace died_181_365 = 0 if indextodeath_caseindex>365
(1,183,181 real changes made)

. replace died_181_365 = 0 if (indextodeath_caseindex>ttstudyend) &
(inrange(indextodeath_caseindex, 181, 365)) ///
>
& (inrange(ttstudyend, 181, 365))
(63 real changes made)

. tab died_181_365, m

died_181_36 |
          5 |      Freq.     Percent        Cum.
```

```
------------+-----------------------------------
          0 |  1,183,244       95.00       95.00
          1 |     26,067        2.09       97.10
          . |     36,173        2.90      100.00
------------+-----------------------------------
      Total |  1,245,484      100.00

. sum indextodeath_caseindex if indextodeath_caseindex<181

    Variable |        Obs        Mean    Std. dev.       Min        Max
-------------+---------------------------------------------------------
indextodea~x |     36,173    67.94139    54.91375          0        180

.
. * Died Day 366-730
. gen died_366_730 = .
(1,245,484 missing values generated)

. replace died_366_730 = 1 if inrange(indextodeath_caseindex, 366, 730)
(32,904 real changes made)

. replace died_366_730 = 0 if indextodeath_caseindex>730
(1,150,277 real changes made)

. replace died_366_730 = 0 if (indextodeath_caseindex>ttstudyend) &
(inrange(indextodeath_caseindex, 366, 730)) ///
>
& (inrange(ttstudyend, 366, 730))
(12,316 real changes made)

. tab died_366_730, m

died_366_73 |
          0 |      Freq.     Percent        Cum.
------------+-----------------------------------
          0 |  1,162,593       93.34       93.34
          1 |     20,588        1.65       95.00
          . |     62,303        5.00      100.00
------------+-----------------------------------
      Total |  1,245,484      100.00

. sum indextodeath_caseindex if indextodeath_caseindex<366

    Variable |        Obs        Mean    Std. dev.       Min        Max
-------------+---------------------------------------------------------
indextodea~x |     62,303    154.2197    115.0548          0        365

.
. * Died Day 0-365
. gen died_0_365 = .
(1,245,484 missing values generated)

. replace died_0_365 = 1 if inrange(indextodeath_caseindex, 0, 365)
(62,303 real changes made)
```

```
. replace died_0_365 = 0 if indextodeath_caseindex>365
(1,183,181 real changes made)

. replace died_0_365 = 0 if (indextodeath_caseindex>ttstudyend) &
(inrange(indextodeath_caseindex, 0, 365)) ///
>
& (inrange(ttstudyend, 0, 365))
(63 real changes made)

.
. * Died Day 0-730
. gen died_0_730 = .
(1,245,484 missing values generated)

. replace died_0_730 = 1 if inrange(indextodeath_caseindex, 0, 730)
(95,207 real changes made)

. replace died_0_730 = 0 if indextodeath_caseindex>730
(1,150,277 real changes made)

. replace died_0_730 = 0 if (indextodeath_caseindex>ttstudyend) &
(inrange(indextodeath_caseindex, 0, 730)) ///
>
& (inrange(ttstudyend, 0, 730))
(12,810 real changes made)

.
. * Died Day 91-730
. gen died_91_730 = .
(1,245,484 missing values generated)

. replace died_91_730 = 1 if inrange(indextodeath_caseindex, 91, 730)
(71,393 real changes made)

. replace died_91_730 = 0 if indextodeath_caseindex>730
(1,150,277 real changes made)

. replace died_91_730 = 0 if (indextodeath_caseindex>ttstudyend) &
(inrange(indextodeath_caseindex, 91, 730)) ///
>
& (inrange(ttstudyend, 91, 730))
>
(12,810 real changes made)

.
. *---------------------------
. * Time to Event Variables
. *---------------------------
.
. * alive on index day, censored at 90 days
. gen tte_0_90 = ttstudyend

. replace tte_0_90 = 90 if ttstudyend>90
```

```
(1,245,484 real changes made)

. replace tte_0_90 = indextodeath_caseindex  ///
>                                         if indextodeath_caseindex
indextodeath_caseindex<90
(23,659 real changes made)

.
. * alive on Day 91, censored at 180 days
. gen tte_91_180 = ttstudyend

. replace tte_91_180 = 180 if ttstudyend>180
(1,245,484 real changes made)

. replace tte_91_180 = indextodeath_caseindex if  ///
>                                                 indextodeath_caseindex
inrange(indextodeath_caseindex, 91, 180)
(12,195 real changes made)

. replace tte_91_180 = . if alive_day91==0
(23,814 real changes made, 23,814 to missing)

.
. * alive on Day 181, censored at 365 days
. gen tte_181_365 = ttstudyend

. replace tte_181_365 = 365 if ttstudyend>365
(1,191,419 real changes made)

. replace tte_181_365 = indextodeath_caseindex if         ///
>                                                 indextodeath_caseindex
inrange(indextodeath_caseindex, 181, 365)
(25,921 real changes made)

. replace tte_181_365 = . if alive_day181==0
(36,173 real changes made, 36,173 to missing)

.
. * alive on Day 366, censored at 730 days
. gen tte_366_730 = ttstudyend

. replace tte_366_730 = 730 if ttstudyend>730
(13,998 real changes made)

. replace tte_366_730 = indextodeath_caseindex if         ///
>                                                 indextodeath_caseindex
inrange(indextodeath_caseindex, 366, 730)
(20,049 real changes made)

. replace tte_366_730 = . if alive_day366==0
(114,374 real changes made, 114,374 to missing)

.
. * alive on index day, censored at 365 days
```

```
. gen tte_0_365 = ttstudyend

. replace tte_0_365 = 365 if ttstudyend>365
(1,191,419 real changes made)

. replace tte_0_365 = indextodeath_caseindex if ///
>                                                   indextodeath_caseindex
indextodeath_caseindex<365
(62,094 real changes made)


.
. * alive on index day, censored at 730 days
. gen tte_0_730 = ttstudyend

. replace tte_0_730 = 730 if ttstudyend>730
(13,998 real changes made)

. replace tte_0_730 = indextodeath_caseindex if ///
>                                                   indextodeath_caseindex
indextodeath_caseindex<730
(82,285 real changes made)


.
. * alive on Day 91, censored at 730 days
. gen tte_91_730 = ttstudyend

. replace tte_91_730 = 730 if ttstudyend>730
(13,998 real changes made)

. replace tte_91_730 = indextodeath_caseindex if  ///
>                                                   indextodeath_caseindex
inrange(indextodeath_caseindex, 91, 730)
(58,471 real changes made)

. replace tte_91_730 = . if alive_day91==0
(23,814 real changes made, 23,814 to missing)


.
. *--------------------------------------------------------------------------
----------
. * for each period, identify whether the case and at least one
comparator
. * in a matched group are alive on the first day of the period
. *--------------------------------------------------------------------------
----------
.
. ** Index Day, Day 0 **
.          * Each match group in dataset has at least one case and one
comparator alive
.          * on index day
.
. ** Alive on Day 91, 181, 366 **
.
. foreach i of numlist 91 181 366 {
```

```
  2. 
.         //cases
.         gen alive_day`i'_case = 0
  3.         replace alive_day`i'_case = 1 if case==1 & alive_day`i'==1
  4.         replace alive_day`i'_case = 0 if case==1 & ttstudyend<`i'
  5.         tab alive_day`i'_case alive_day`i' if case==1
  6.         sum indextodeath if indextodeath<`i' & case==1
  7. 
.         bysort matchgroupnumber: egen alive_day`i'_case_grp = 
max(alive_day`i'_case)
  8. 
. 
.         //comparators
.         gen alive_day`i'_control = 0
  9.         replace alive_day`i'_control = 1 if case==0 & 
alive_day`i'==1
 10.         replace alive_day`i'_control = 0 if case==0 & 
ttstudyend<`i'
 11. 
.         tab alive_day`i'_control alive_day`i' if case==0
 12.         sum indextodeath if indextodeath<`i' & case==0
 13. 
.         bysort matchgroupnumber: egen alive_day`i'_control_num = 
sum(alive_day`i'_control)
 14. 
.         //case and at least one comparator alive on day`i'
.         gen alive_day`i'_casecontrol = 0
 15.         replace alive_day`i'_casecontrol = 1 if 
alive_day`i'_case_grp==1 & alive_day`i'_control_num>=1
 16. 
.         tab alive_day`i'_casecontrol case
 17. 
.         //drop variables we no longer need
.         drop alive_day`i'_case alive_day`i'_case_grp 
alive_day`i'_control alive_day`i'_control_num
 18. 
. }
(195,646 real changes made)
(0 real changes made)

alive_day9 |      alive_day91
    1_case |         0          1 |     Total
-----------+----------------------+----------
         0 |    12,415          0 |    12,415 
         1 |         0    195,646 |   195,646 
-----------+----------------------+----------
     Total |    12,415    195,646 |   208,061 

    Variable |        Obs        Mean    Std. dev.       Min        Max
-------------+---------------------------------------------------------
indextodea~x |     12,415    22.35747    19.79229          0         90
(1,026,024 real changes made)
(0 real changes made)
```

```
alive_day9 |      alive_day91
 1_control |         0          1 |     Total
-----------+----------------------+----------
         0 |    11,399          0 |    11,399 
         1 |         0  1,026,024 | 1,026,024 
-----------+----------------------+----------
     Total |    11,399  1,026,024 | 1,037,423 

    Variable |        Obs        Mean    Std. dev.       Min        Max
-------------+---------------------------------------------------------
indextodea~x |     11,399      44.554    25.89809          0         90
(1,171,141 real changes made)

           |     indicator: is
           |  `index_dt` the date
           | of the first positive
alive_day9 | covid-19 test result
1_casecont |          for
       rol |         0          1 |     Total
-----------+----------------------+----------
         0 |    61,924     12,419 |    74,343 
         1 |   975,499    195,642 | 1,171,141 
-----------+----------------------+----------
     Total | 1,037,423    208,061 | 1,245,484 
(193,602 real changes made)
(0 real changes made)

alive_day1 |     alive_day181
   81_case |         0          1 |     Total
-----------+----------------------+----------
         0 |    14,459          0 |    14,459 
         1 |         0    193,602 |   193,602 
-----------+----------------------+----------
     Total |    14,459    193,602 |   208,061 

    Variable |        Obs        Mean    Std. dev.       Min        Max
-------------+---------------------------------------------------------
indextodea~x |     14,459    38.05222    43.91964          0        180
(1,015,709 real changes made)
(0 real changes made)

alive_day1 |     alive_day181
81_control |         0          1 |     Total
-----------+----------------------+----------
         0 |    21,714          0 |    21,714 
         1 |         0  1,015,709 | 1,015,709 
-----------+----------------------+----------
     Total |    21,714  1,015,709 | 1,037,423 

    Variable |        Obs        Mean    Std. dev.       Min        Max
-------------+---------------------------------------------------------
indextodea~x |     21,714    87.84411    52.42273          0        180
(1,158,905 real changes made)
```

```
           |     indicator: is
           |  `index_dt` the date
           | of the first positive
alive_day1 | covid-19 test result
81_casecon |          for
      trol |         0          1 |     Total
-----------+----------------------+----------
         0 |    72,113     14,466 |    86,579
         1 |   965,310    193,595 | 1,158,905
-----------+----------------------+----------
     Total | 1,037,423    208,061 | 1,245,484
(181,657 real changes made)
(0 real changes made)

alive_day3 |     alive_day366
   66_case |         0          1 |     Total
-----------+----------------------+----------
         0 |    26,404          0 |    26,404
         1 |         0    181,657 |   181,657
-----------+----------------------+----------
     Total |    26,404    181,657 |   208,061

    Variable |        Obs        Mean    Std. dev.       Min        Max
-------------+---------------------------------------------------------
indextodea~x |     17,900    83.07486    103.0125          0        365
(949,453 real changes made)
(0 real changes made)

alive_day3 |     alive_day366
66_control |         0          1 |     Total
-----------+----------------------+----------
         0 |    87,970          0 |    87,970
         1 |         0    949,453 |   949,453
-----------+----------------------+----------
     Total |    87,970    949,453 | 1,037,423

    Variable |        Obs        Mean    Std. dev.       Min        Max
-------------+---------------------------------------------------------
indextodea~x |     44,403       182.9    106.9272          0        365
(1,087,503 real changes made)

           |     indicator: is
           |  `index_dt` the date
           | of the first positive
alive_day3 | covid-19 test result
66_casecon |          for
      trol |         0          1 |     Total
-----------+----------------------+----------
         0 |   131,567     26,414 |   157,981
         1 |   905,856    181,647 | 1,087,503
-----------+----------------------+----------
     Total | 1,037,423    208,061 | 1,245,484

.
```

```
. * Save dataset for use in tables and Cox models
. *save Data\mortality_varforcox_20230516, replace
.
. *-----------------
. * ITT Results
. *-----------------
.
. use Data\mortality_varforcox_20230516, clear
(Created in file
'P:\ORD_Ioannou_202104007D\MethodsGroup\Programs\CORC_DataExplor)

.
. * excess deaths (Results)
. tab died_0_730 infected, co

+-------------------+
| Key               |
|-------------------|
|     frequency     |
| column percentage |
+-------------------+

           |       infected
died_0_730 |         0          1 |     Total
-----------+----------------------+----------
         0 |   975,435    187,652 | 1,163,087
           |     94.02      90.19 |     93.38
-----------+----------------------+----------
         1 |    61,988     20,409 |    82,397
           |      5.98       9.81 |      6.62
-----------+----------------------+----------
     Total | 1,037,423    208,061 | 1,245,484
           |    100.00     100.00 |    100.00

.
. di 20409/208061 // 0.09809143 - mortality rate of cases
.09809143

. di 61988/1037423 // 0.0597519 - moratlity rate of comparators
.0597519

. di 208061*0.0597519 //12432.04 - number case deaths if comparator rate
12432.04

. di 20409-12432.04  //7976.96 excess deaths
7976.96

.
. * Overall *
.
. * 90-day mortality for cases and comparators who are alive on index
day
. tab died_0_90 infected  , co
```

```
+-------------------+
| Key               |
|-------------------|
|     frequency     |
| column percentage |
+-------------------+

           |       infected
 died_0_90 |         0          1 |     Total
-----------+----------------------+----------
         0 | 1,026,024    195,646 | 1,221,670
           |     98.90      94.03 |     98.09
-----------+----------------------+----------
         1 |    11,399     12,415 |    23,814
           |      1.10       5.97 |      1.91
-----------+----------------------+----------
     Total | 1,037,423    208,061 | 1,245,484
           |    100.00     100.00 |    100.00

. cs died_0_90 infected

                 |        infected        |
                 |   Exposed   Unexposed  |      Total
-----------------+------------------------+-----------
           Cases |     12415       11399  |      23814
        Noncases |    195646     1026024  |    1221670
-----------------+------------------------+-----------
           Total |    208061     1037423  |    1245484
                 |                        |
            Risk |    .05967    .0109878  |   .0191203
                 |                        |
                 |      Point estimate    |    [95% conf. interval]
                 |------------------------+------------------------
 Risk difference |         .0486822       |    .0476448    .0497196
      Risk ratio |         5.430567       |    5.296565    5.567959
 Attr. frac. ex. |         .8158572       |    .8111984     .820401
 Attr. frac. pop |         .4253324       |
                 +-------------------------------------------------
                               chi2(1) = 21899.73  Pr>chi2 = 0.0000

.
. * 180-day mortality for cases and comparators who are alive on day 91
&
. * with case and at least one comparator from matchgroup alive
. tab died_91_180 infected if alive_day91_casecontrol==1 , co

+-------------------+
| Key               |
|-------------------|
|     frequency     |
| column percentage |
+-------------------+

died_91_18 |       infected
```

```
          0 |         0          1 |     Total
-----------+----------------------+----------
          0 |   955,483    193,598 | 1,149,081
            |     99.01      98.96 |     99.00
-----------+----------------------+----------
          1 |     9,514      2,044 |    11,558
            |      0.99       1.04 |      1.00
-----------+----------------------+----------
      Total |   964,997    195,642 | 1,160,639
            |    100.00     100.00 |    100.00

. cs died_91_180 infected if alive_day91_casecontrol==1

                 |        infected        |
                 |   Exposed   Unexposed  |      Total
-----------------+------------------------+-----------
           Cases |      2044        9514  |      11558
        Noncases |    193598      955483  |    1149081
-----------------+------------------------+-----------
           Total |    195642      964997  |    1160639
                 |                        |
            Risk |  .0104477    .0098591  |   .0099583
                 |                        |
                 |      Point estimate    |    [95% conf. interval]
                 |------------------------+-----------------------
 Risk difference |         .0005886       |    .0000968    .0010803
      Risk ratio |         1.059697       |    1.010503    1.111285
 Attr. frac. ex. |         .0563338       |    .0103938    .1001412
 Attr. frac. pop |         .0099625       |
                 +------------------------------------------------
                               chi2(1) =     5.72  Pr>chi2 = 0.0168

.
. * 365-day mortality for cases and comparators who are alive on day 181
& not censored
. tab died_181_365 infected if alive_day181_casecontrol==1 , co

+-------------------+
| Key               |
|-------------------|
|     frequency     |
| column percentage |
+-------------------+

died_181_3 |       infected
        65 |         0          1 |     Total
-----------+----------------------+----------
         0 |   925,006    190,163 | 1,115,169
           |     97.82      98.23 |     97.89
-----------+----------------------+----------
         1 |    20,594      3,432 |    24,026
           |      2.18       1.77 |      2.11
-----------+----------------------+----------
     Total |   945,600    193,595 | 1,139,195
```

```
           |    100.00     100.00 |    100.00

. cs died_181_365 infected if alive_day181_casecontrol==1

                 |        infected         |
                 |   Exposed   Unexposed   |      Total
-----------------+-------------------------+------------
           Cases |      3432       20594   |      24026
        Noncases |    190163      925006   |    1115169
-----------------+-------------------------+------------
           Total |    193595      945600   |    1139195
                 |                         |
            Risk |  .0177277    .0217788   |   .0210903
                 |                         |
                 |      Point estimate     |    [95% conf. interval]
                 |-------------------------+------------------------
 Risk difference |         -.004051        |   -.0047084   -.0033937
      Risk ratio |         .8139916        |    .7853629    .8436638
 Prev. frac. ex. |         .1860084        |    .1563362    .2146371
 Prev. frac. pop |         .0316103        |
                 +--------------------------------------------------
                               chi2(1) =   127.73  Pr>chi2 = 0.0000

.
. * 730-day mortality for cases and comparators who are alive on day 366
& not censored
. tab died_366_730 infected if alive_day366_casecontrol==1 , co

+-------------------+
| Key               |
|-------------------|
|     frequency     |
| column percentage |
+-------------------+

died_366_7 |       infected
        30 |         0          1 |     Total
-----------+----------------------+----------
         0 |   852,355    179,130 | 1,031,485
           |     98.22      98.61 |     98.29
-----------+----------------------+----------
         1 |    15,481      2,517 |    17,998
           |      1.78       1.39 |      1.71
-----------+----------------------+----------
     Total |   867,836    181,647 | 1,049,483
           |    100.00     100.00 |    100.00

. cs died_366_730 infected if alive_day366_casecontrol==1

                 |        infected         |
                 |   Exposed   Unexposed   |      Total
-----------------+-------------------------+------------
           Cases |      2517       15481   |      17998
        Noncases |    179130      852355   |    1031485
```

```
-----------------+------------------------+------------
           Total |    181647      867836  |    1049483
                 |                        |
            Risk |  .0138565    .0178386  |   .0171494
                 |                        |
                 |      Point estimate    |    [95% conf. interval]
                 |------------------------+------------------------
 Risk difference |        -.0039821       |   -.0045875   -.0033767
      Risk ratio |         .7767721       |    .7449587    .8099442
 Prev. frac. ex. |         .2232279       |    .1900558    .2550413
 Prev. frac. pop |         .0386368       |
                 +-------------------------------------------------
                               chi2(1) =   141.31  Pr>chi2 = 0.0000

.
. *------------
. * Age <65
. *------------
.
. * 90-day mortality for cases and comparators who are alive on day 1
. tab died_0_90 infected if agecat==1 & ttstudyend>=0, co

+-------------------+
| Key               |
|-------------------|
|     frequency     |
| column percentage |
+-------------------+

           |       infected
 died_0_90 |         0          1 |     Total
-----------+----------------------+----------
         0 |   553,541    113,650 |   667,191
           |     99.70      98.79 |     99.54
-----------+----------------------+----------
         1 |     1,676      1,391 |     3,067
           |      0.30       1.21 |      0.46
-----------+----------------------+----------
     Total |   555,217    115,041 |   670,258
           |    100.00     100.00 |    100.00

. cs died_0_90 infected if agecat==1 & ttstudyend>=0

                 |        infected        |
                 |   Exposed   Unexposed  |      Total
-----------------+------------------------+------------
           Cases |      1391        1676  |       3067
        Noncases |    113650      553541  |     667191
-----------------+------------------------+------------
           Total |    115041      555217  |     670258
                 |                        |
            Risk |  .0120913    .0030186  |   .0045758
                 |                        |
                 |      Point estimate    |    [95% conf. interval]
```

```
                 |-------------------------+------------------------
 Risk difference |         .0090727        |    .0084249    .0097205
      Risk ratio |          4.00556        |    3.731753    4.299456
 Attr. frac. ex. |          .750347        |    .7320294    .7674125
 Attr. frac. pop |         .3403106        |
                 +-------------------------------------------------
                               chi2(1) =  1722.13  Pr>chi2 = 0.0000

.
. * 180-day mortality for cases and comparators who are alive on day 91
. tab died_91_180 infected if alive_day91_casecontrol==1 & agecat==1 &
ttstudyend>=91, co

+-------------------+
| Key               |
|-------------------|
|     frequency     |
| column percentage |
+-------------------+

died_91_18 |       infected
         0 |         0          1 |     Total
-----------+----------------------+----------
         0 |   521,865    113,344 |   635,209
           |     99.73      99.73 |     99.73
-----------+----------------------+----------
         1 |     1,400        303 |     1,703
           |      0.27       0.27 |      0.27
-----------+----------------------+----------
     Total |   523,265    113,647 |   636,912
           |    100.00     100.00 |    100.00

. cs died_91_180 infected if alive_day91_casecontrol==1 & agecat==1 &
ttstudyend>=91

                 |        infected         |
                 |   Exposed   Unexposed   |      Total
-----------------+-------------------------+-----------
           Cases |       303        1400   |       1703
        Noncases |    113344      521865   |     635209
-----------------+-------------------------+-----------
           Total |    113647      523265   |     636912
                 |                         |
            Risk |  .0026662    .0026755   |   .0026738
                 |                         |
                 |      Point estimate     |    [95% conf. interval]
                 |-------------------------+------------------------
 Risk difference |        -9.36e-06        |   -.0003402    .0003215
      Risk ratio |         .9965023        |    .8802729    1.128078
 Prev. frac. ex. |         .0034977        |   -.1280784    .1197271
 Prev. frac. pop |         .0006241        |
                 +-------------------------------------------------
                               chi2(1) =     0.00  Pr>chi2 = 0.9558
```

```
.
. * 365-day mortality for cases and comparators who are alive on day 181
& not censored
. tab died_181_365 infected if alive_day181_casecontrol==1 & agecat==1 &
ttstudyend>=181, co

+-------------------+
| Key               |
|-------------------|
|     frequency     |
| column percentage |
+-------------------+

died_181_3 |       infected
        65 |         0          1 |     Total
-----------+----------------------+----------
         0 |   513,794    112,816 |   626,610
           |     99.39      99.53 |     99.41
-----------+----------------------+----------
         1 |     3,178        528 |     3,706
           |      0.61       0.47 |      0.59
-----------+----------------------+----------
     Total |   516,972    113,344 |   630,316
           |    100.00     100.00 |    100.00

. cs died_181_365 infected if alive_day181_casecontrol==1 & agecat==1 &
ttstudyend>=181

                 |        infected        |
                 |   Exposed   Unexposed  |      Total
-----------------+------------------------+-----------
           Cases |       528        3178  |       3706
        Noncases |    112816      513794  |     626610
-----------------+------------------------+-----------
           Total |    113344      516972  |     630316
                 |                        |
            Risk |  .0046584    .0061473  |   .0058796
                 |                        |
                 |      Point estimate    |    [95% conf. interval]
                 |------------------------+------------------------
 Risk difference |       -.0014889        |    -.001939   -.0010389
      Risk ratio |        .7577894        |    .6912628    .8307184
 Prev. frac. ex. |        .2422106        |    .1692816    .3087372
 Prev. frac. pop |        .0435545        |
                 +-------------------------------------------------
                               chi2(1) =    35.26  Pr>chi2 = 0.0000

.
. * 730-day mortality for cases and comparators who are alive on day 366
& not censored
. tab died_366_730 infected if alive_day366_casecontrol==1 & agecat==1 &
ttstudyend>=366, co

+-------------------+
```

```
| Key               |
|-------------------|
|     frequency     |
| column percentage |
+-------------------+

died_366_7 |       infected
        30 |         0          1 |     Total
-----------+----------------------+----------
         0 |   476,993    106,579 |   583,572 
           |     99.51      99.64 |     99.53 
-----------+----------------------+----------
         1 |     2,364        390 |     2,754 
           |      0.49       0.36 |      0.47 
-----------+----------------------+----------
     Total |   479,357    106,969 |   586,326 
           |    100.00     100.00 |    100.00 

. cs died_366_730 infected if alive_day366_casecontrol==1 & agecat==1 &
ttstudyend>=366

                 |        infected        |
                 |   Exposed   Unexposed  |      Total
-----------------+------------------------+------------
           Cases |       390        2364  |       2754
        Noncases |    106579      476993  |     583572
-----------------+------------------------+------------
           Total |    106969      479357  |     586326
                 |                        |
            Risk |  .0036459    .0049316  |    .004697
                 |                        |
                 |      Point estimate    |    [95% conf. interval]
                 |------------------------+------------------------
 Risk difference |        -.0012857       |   -.0016977   -.0008736 
      Risk ratio |         .7392959       |    .6643324    .8227183 
 Prev. frac. ex. |         .2607041       |    .1772817    .3356676 
 Prev. frac. pop |         .0475627       |
                 +-------------------------------------------------
                               chi2(1) =    30.92  Pr>chi2 = 0.0000

. 
. *------------
. * Age 65-85
. *------------
. 
. * 90-day mortality for cases and comparators who are alive on day 1
. tab died_0_90 infected if agecat==2 & ttstudyend>=0, co

+-------------------+
| Key               |
|-------------------|
|     frequency     |
| column percentage |
+-------------------+
```

```
           |       infected
 died_0_90 |         0          1 |     Total
-----------+----------------------+----------
         0 |   424,483     74,472 |   498,955 
           |     98.42      90.49 |     97.15 
-----------+----------------------+----------
         1 |     6,818      7,829 |    14,647 
           |      1.58       9.51 |      2.85 
-----------+----------------------+----------
     Total |   431,301     82,301 |   513,602 
           |    100.00     100.00 |    100.00 

. cs died_0_90 infected if agecat==2 & ttstudyend>=0

                 | infected               |
                 |   Exposed   Unexposed  |      Total
-----------------+------------------------+-----------
           Cases |      7829        6818  |      14647
        Noncases |     74472      424483  |     498955
-----------------+------------------------+-----------
           Total |     82301      431301  |     513602
                 |                        |
            Risk |  .0951264     .015808  |   .0285182
                 |                        |
                 |      Point estimate    |    [95% conf. interval]
                 |------------------------+------------------------
 Risk difference |         .0793184       |    .0772797    .0813571 
      Risk ratio |         6.017618       |    5.830439    6.210807 
 Attr. frac. ex. |         .8338213       |    .8284863    .8389903 
 Attr. frac. pop |         .4456876       |
                 +-------------------------------------------------
                               chi2(1) = 15694.61  Pr>chi2 = 0.0000

. 
. * 180-day mortality for cases and comparators who are alive on day 91
. tab died_91_180 infected if alive_day91_casecontrol==1 & agecat==2 & 
ttstudyend>=91, co

+-------------------+
| Key               |
|-------------------|
|     frequency     |
| column percentage |
+-------------------+

died_91_18 |       infected
         0 |         0          1 |     Total
-----------+----------------------+----------
         0 |   391,280     73,211 |   464,491 
           |     98.55      98.31 |     98.51 
-----------+----------------------+----------
         1 |     5,753      1,260 |     7,013 
           |      1.45       1.69 |      1.49 
```

```
-----------+----------------------+----------
     Total |   397,033     74,471 |   471,504 
           |    100.00     100.00 |    100.00 

. cs died_91_180 infected if alive_day91_casecontrol==1 & agecat==2 &
ttstudyend>=91

                 |        infected        |
                 |   Exposed   Unexposed  |      Total
-----------------+------------------------+-----------
           Cases |      1260        5753  |       7013
        Noncases |     73211      391280  |     464491
-----------------+------------------------+-----------
           Total |     74471      397033  |     471504
                 |                        |
            Risk |  .0169193      .01449  |   .0148737
                 |                        |
                 |      Point estimate    |    [95% conf. interval]
                 |------------------------+------------------------
 Risk difference |         .0024294       |    .0014313    .0034274 
      Risk ratio |         1.167658       |    1.099154    1.240431 
 Attr. frac. ex. |         .1435847       |    .0902098    .1938283 
 Attr. frac. pop |         .0257973       |
                 +-------------------------------------------------
                               chi2(1) =    25.26  Pr>chi2 = 0.0000

. 
. * 365-day mortality for cases and comparators who are alive on day 181
& not censored
. tab died_181_365 infected if alive_day181_casecontrol==1 & agecat==2 &
ttstudyend>=181, co

+-------------------+
| Key               |
|-------------------|
|     frequency     |
| column percentage |
+-------------------+

died_181_3 |       infected
        65 |         0          1 |     Total
-----------+----------------------+----------
         0 |   374,071     71,093 |   445,164 
           |     96.70      97.11 |     96.77 
-----------+----------------------+----------
         1 |    12,754      2,116 |    14,870 
           |      3.30       2.89 |      3.23 
-----------+----------------------+----------
     Total |   386,825     73,209 |   460,034 
           |    100.00     100.00 |    100.00 

. cs died_181_365 infected if alive_day181_casecontrol==1 & agecat==2 &
ttstudyend>=181
```

```
                 |        infected        |
                 |   Exposed   Unexposed  |      Total
-----------------+------------------------+------------
           Cases |      2116       12754  |      14870
        Noncases |     71093      374071  |     445164
-----------------+------------------------+------------
           Total |     73209      386825  |     460034
                 |                        |
            Risk |  .0289036     .032971  |   .0323237
                 |                        |
                 |      Point estimate    |    [95% conf. interval]
                 |------------------------+------------------------
 Risk difference |       -.0040674        |   -.0054051   -.0027297
      Risk ratio |         .876636        |    .8377907    .9172825
 Prev. frac. ex. |         .123364        |    .0827175    .1622093
 Prev. frac. pop |        .0196319        |
                 +-------------------------------------------------
                               chi2(1) =    32.56  Pr>chi2 = 0.0000

.
. * 730-day mortality for cases and comparators who are alive on day 366
& not censored
. tab died_366_730 infected if alive_day366_casecontrol==1 & agecat==2 &
ttstudyend>=366, co

+-------------------+
| Key               |
|-------------------|
|     frequency     |
| column percentage |
+-------------------+

died_366_7 |       infected
        30 |         0          1 |     Total
-----------+----------------------+----------
         0 |   343,610     67,016 |   410,626
           |     97.29      97.69 |     97.36
-----------+----------------------+----------
         1 |     9,562      1,586 |    11,148
           |      2.71       2.31 |      2.64
-----------+----------------------+----------
     Total |   353,172     68,602 |   421,774
           |    100.00     100.00 |    100.00

. cs died_366_730 infected if alive_day366_casecontrol==1 & agecat==2 &
ttstudyend>=366

                 |        infected        |
                 |   Exposed   Unexposed  |      Total
-----------------+------------------------+------------
           Cases |      1586        9562  |      11148
        Noncases |     67016      343610  |     410626
-----------------+------------------------+------------
           Total |     68602      353172  |     421774
```

```
                 |                        |
            Risk |  .0231189    .0270746  |   .0264312
                 |                        |
                 |      Point estimate    |    [95% conf. interval]
                 |------------------------+------------------------
 Risk difference |       -.0039558        |   -.0052012   -.0027103
      Risk ratio |        .8538939        |    .8102154    .8999272
 Prev. frac. ex. |        .1461061        |    .1000728    .1897846
 Prev. frac. pop |        .0237643        |
                 +-------------------------------------------------
                               chi2(1) =    34.93  Pr>chi2 = 0.0000

.
. *------------
. * Age 85+
. *------------
.
. * 90-day mortality for cases and comparators who are alive on day 1
. tab died_0_90 infected if agecat==3 & ttstudyend>=0, co

+-------------------+
| Key               |
|-------------------|
|     frequency     |
| column percentage |
+-------------------+

           |       infected
 died_0_90 |         0          1 |     Total
-----------+----------------------+----------
         0 |    48,000      7,524 |    55,524 
           |     94.29      70.19 |     90.10 
-----------+----------------------+----------
         1 |     2,905      3,195 |     6,100 
           |      5.71      29.81 |      9.90 
-----------+----------------------+----------
     Total |    50,905     10,719 |    61,624 
           |    100.00     100.00 |    100.00 

. cs died_0_90 infected if agecat==3 & ttstudyend>=0

                 |        infected        |
                 |   Exposed   Unexposed  |      Total
-----------------+------------------------+-----------
           Cases |      3195        2905  |       6100
        Noncases |      7524       48000  |      55524
-----------------+------------------------+-----------
           Total |     10719       50905  |      61624
                 |                        |
            Risk |  .2980688    .0570671  |   .0989874
                 |                        |
                 |      Point estimate    |    [95% conf. interval]
                 |------------------------+------------------------
 Risk difference |        .2410018        |    .2321112    .2498923
```

```
      Risk ratio |         5.223131       |    4.989677    5.467507
 Attr. frac. ex. |         .8085439       |    .7995862    .8171013
 Attr. frac. pop |         .4234915       |
                 +-------------------------------------------------
                               chi2(1) =  5766.26  Pr>chi2 = 0.0000

.
. * 180-day mortality for cases and comparators who are alive on day 91
. tab died_91_180 infected if alive_day91_casecontrol==1 & agecat==3 &
ttstudyend>=91, co

+-------------------+
| Key               |
|-------------------|
|     frequency     |
| column percentage |
+-------------------+

died_91_18 |       infected
         0 |         0          1 |     Total
-----------+----------------------+----------
         0 |    42,338      7,043 |    49,381 
           |     94.72      93.61 |     94.56 
-----------+----------------------+----------
         1 |     2,361        481 |     2,842 
           |      5.28       6.39 |      5.44 
-----------+----------------------+----------
     Total |    44,699      7,524 |    52,223 
           |    100.00     100.00 |    100.00 

. cs died_91_180 infected if alive_day91_casecontrol==1 & agecat==3 &
ttstudyend>=91

                 |        infected        |
                 |   Exposed   Unexposed  |      Total
-----------------+------------------------+-----------
           Cases |       481        2361  |       2842
        Noncases |      7043       42338  |      49381
-----------------+------------------------+-----------
           Total |      7524       44699  |      52223
                 |                        |
            Risk |  .0639288      .05282  |   .0544205
                 |                        |
                 |      Point estimate    |    [95% conf. interval]
                 |------------------------+------------------------
 Risk difference |         .0111088       |    .0052052    .0170124
      Risk ratio |         1.210314       |    1.100674    1.330876
 Attr. frac. ex. |         .1737682       |    .0914654    .2486153
 Attr. frac. pop |         .0294098       |
                 +-------------------------------------------------
                               chi2(1) =    15.44  Pr>chi2 = 0.0001

.
. * 365-day mortality for cases and comparators who are alive on day 181
```

```
& not censored
. tab died_181_365 infected if alive_day181_casecontrol==1 & agecat==3 &
ttstudyend>=181, co

+-------------------+
| Key               |
|-------------------|
|     frequency     |
| column percentage |
+-------------------+

died_181_3 |       infected
        65 |         0          1 |     Total
-----------+----------------------+----------
         0 |    37,141      6,254 |    43,395
           |     88.85      88.81 |     88.84
-----------+----------------------+----------
         1 |     4,662        788 |     5,450
           |     11.15      11.19 |     11.16
-----------+----------------------+----------
     Total |    41,803      7,042 |    48,845
           |    100.00     100.00 |    100.00

. cs died_181_365 infected if alive_day181_casecontrol==1 & agecat==3 &
ttstudyend>=181

                 |        infected        |
                 |   Exposed   Unexposed  |      Total
-----------------+------------------------+-----------
           Cases |       788        4662  |       5450
        Noncases |      6254       37141  |      43395
-----------------+------------------------+-----------
           Total |      7042       41803  |      48845
                 |                        |
            Risk |     .1119    .1115231  |   .1115774
                 |                        |
                 |      Point estimate    |    [95% conf. interval]
                 |------------------------+------------------------
 Risk difference |         .0003769       |   -.0075803    .0083341
      Risk ratio |          1.00338       |    .9344751    1.077365
 Attr. frac. ex. |         .0033685       |   -.0701195    .0718098
 Attr. frac. pop |          .000487       |
                 +-------------------------------------------------
                               chi2(1) =     0.01  Pr>chi2 = 0.9260

.
. * 730-day mortality for cases and comparators who are alive on day 366
& not censored
. tab died_366_730 infected if alive_day366_casecontrol==1 & agecat==3 &
ttstudyend>=366, co

+-------------------+
| Key               |
|-------------------|
```

```
|     frequency     |
| column percentage |
+-------------------+

died_366_7 |       infected
        30 |         0          1 |     Total
-----------+----------------------+----------
         0 |    31,752      5,535 |    37,287
           |     89.93      91.10 |     90.10
-----------+----------------------+----------
         1 |     3,555        541 |     4,096
           |     10.07       8.90 |      9.90
-----------+----------------------+----------
     Total |    35,307      6,076 |    41,383
           |    100.00     100.00 |    100.00

. cs died_366_730 infected if alive_day366_casecontrol==1 & agecat==3 &
ttstudyend>=366

                 |        infected        |
                 |   Exposed   Unexposed  |      Total
-----------------+------------------------+-----------
           Cases |       541        3555  |       4096
        Noncases |      5535       31752  |      37287
-----------------+------------------------+-----------
           Total |      6076       35307  |      41383
                 |                        |
            Risk |  .0890388    .1006882  |   .0989778
                 |                        |
                 |      Point estimate    |    [95% conf. interval]
                 |------------------------+------------------------
 Risk difference |        -.0116494       |   -.0194682   -.0038306
      Risk ratio |         .8843022       |    .8112224    .9639655
 Prev. frac. ex. |         .1156978       |    .0360345    .1887776
 Prev. frac. pop |         .0169872       |
                 +-------------------------------------------------
                               chi2(1) =     7.89  Pr>chi2 = 0.0050

.
. *-----------
. * Wave 1
. *-----------
.
. * 90-day mortality for cases and comparators who are alive on day 1
. tab died_0_90 infected if wave==1 & ttstudyend>=0, co

+-------------------+
| Key               |
|-------------------|
|     frequency     |
| column percentage |
+-------------------+

           |       infected
```

```
 died_0_90 |         0          1 |     Total
-----------+----------------------+----------
         0 |    99,999     18,259 |   118,258 
           |     98.48      89.80 |     97.03 
-----------+----------------------+----------
         1 |     1,547      2,075 |     3,622 
           |      1.52      10.20 |      2.97 
-----------+----------------------+----------
     Total |   101,546     20,334 |   121,880 
           |    100.00     100.00 |    100.00 

. cs died_0_90 infected if wave==1 & ttstudyend>=0

                 |        infected        |
                 |   Exposed   Unexposed  |      Total
-----------------+------------------------+-----------
           Cases |      2075        1547  |       3622
        Noncases |     18259       99999  |     118258
-----------------+------------------------+-----------
           Total |     20334      101546  |     121880
                 |                        |
            Risk |  .1020458    .0152345  |   .0297178
                 |                        |
                 |      Point estimate    |    [95% conf. interval]
                 |------------------------+------------------------
 Risk difference |         .0868114       |    .0825831    .0910397
      Risk ratio |         6.698349       |     6.28251    7.141713
 Attr. frac. ex. |         .8507095       |     .840828    .8599776
 Attr. frac. pop |         .4873612       |
                 +-------------------------------------------------
                               chi2(1) =  4427.84  Pr>chi2 = 0.0000

. 
. * 180-day mortality for cases and comparators who are alive on day 91
. tab died_91_180 infected if alive_day91_casecontrol==1 & wave==1 &
ttstudyend>=91, co

+-------------------+
| Key               |
|-------------------|
|     frequency     |
| column percentage |
+-------------------+

died_91_18 |       infected
         0 |         0          1 |     Total
-----------+----------------------+----------
         0 |    88,661     17,947 |   106,608 
           |     98.65      98.29 |     98.59 
-----------+----------------------+----------
         1 |     1,217        312 |     1,529 
           |      1.35       1.71 |      1.41 
-----------+----------------------+----------
     Total |    89,878     18,259 |   108,137 
```

```
           |    100.00     100.00 |    100.00

. cs died_91_180 infected if alive_day91_casecontrol==1 & wave==1 &
ttstudyend>=91

                 |        infected         |
                 |   Exposed   Unexposed   |      Total
-----------------+-------------------------+-----------
           Cases |       312        1217   |       1529
        Noncases |     17947       88661   |     106608
-----------------+-------------------------+-----------
           Total |     18259       89878   |     108137
                 |                         |
            Risk |  .0170875    .0135406   |   .0141395
                 |                         |
                 |      Point estimate     |    [95% conf. interval]
                 |-------------------------+-------------------------
 Risk difference |         .0035469        |    .0015209    .0055728
      Risk ratio |         1.261945        |    1.115499    1.427617
 Attr. frac. ex. |         .2075724        |    .1035404    .2995318
 Attr. frac. pop |         .0423562        |
                 +---------------------------------------------------
                               chi2(1) =    13.70  Pr>chi2 = 0.0002

.
. * 365-day mortality for cases and comparators who are alive on day 181
& not censored
. tab died_181_365 infected if alive_day181_casecontrol==1 & wave==1 &
ttstudyend>=181, co

+-------------------+
| Key               |
|-------------------|
|     frequency     |
| column percentage |
+-------------------+

died_181_3 |       infected
       65 |         0          1 |     Total
-----------+----------------------+----------
         0 |    84,668     17,491 |   102,159
           |     97.13      97.47 |     97.19
-----------+----------------------+----------
         1 |     2,501        454 |     2,955
           |      2.87       2.53 |      2.81
-----------+----------------------+----------
     Total |    87,169     17,945 |   105,114
           |    100.00     100.00 |    100.00

. cs died_181_365 infected if alive_day181_casecontrol==1 & wave==1 &
ttstudyend>=181

                 |        infected         |
                 |   Exposed   Unexposed   |      Total
```

```
-----------------+------------------------+------------
           Cases |       454        2501  |       2955
        Noncases |     17491       84668  |     102159
-----------------+------------------------+------------
           Total |     17945       87169  |     105114
                 |                        |
            Risk |  .0252995    .0286914  |   .0281123
                 |                        |
                 |      Point estimate    |    [95% conf. interval]
                 |------------------------+------------------------
 Risk difference |        -.0033919       |   -.0059427    -.000841
      Risk ratio |         .8817811       |    .7989165    .9732404
 Prev. frac. ex. |         .1182189       |    .0267596    .2010835
 Prev. frac. pop |         .0201823       |
                 +-------------------------------------------------
                               chi2(1) =     6.27  Pr>chi2 = 0.0123

.
. * 730-day mortality for cases and comparators who are alive on day 366
& not censored
. tab died_366_730 infected if alive_day366_casecontrol==1 & wave==1 &
ttstudyend>=181, co

+-------------------+
| Key               |
|-------------------|
|     frequency     |
| column percentage |
+-------------------+

died_366_7 |       infected
        30 |         0          1 |     Total
-----------+----------------------+----------
         0 |    78,914     16,785 |    95,699 
           |     95.53      95.96 |     95.60 
-----------+----------------------+----------
         1 |     3,694        706 |     4,400 
           |      4.47       4.04 |      4.40 
-----------+----------------------+----------
     Total |    82,608     17,491 |   100,099 
           |    100.00     100.00 |    100.00 

. cs died_366_730 infected if alive_day366_casecontrol==1 & wave==1 &
ttstudyend>=181

                 |        infected        |
                 |   Exposed   Unexposed  |      Total
-----------------+------------------------+------------
           Cases |       706        3694  |       4400
        Noncases |     16785       78914  |      95699
-----------------+------------------------+------------
           Total |     17491       82608  |     100099
                 |                        |
            Risk |  .0403636    .0447172  |   .0439565
```

```
                 |                        |
                 |      Point estimate    |    [95% conf. interval]
                 |------------------------+------------------------
 Risk difference |        -.0043536       |    -.007593   -.0011142
      Risk ratio |         .9026415       |    .8342144    .9766813
 Prev. frac. ex. |         .0973585       |    .0233187    .1657856
 Prev. frac. pop |         .0170121       |
                 +-------------------------------------------------
                               chi2(1) =     6.51  Pr>chi2 = 0.0107

.
. *-----------
. * Wave 2
. *-----------
.
. * 90-day mortality for cases and comparators who are alive on day 1
. tab died_0_90 infected if wave==2 & ttstudyend>=0, co

+-------------------+
| Key               |
|-------------------|
|     frequency     |
| column percentage |
+-------------------+

           |       infected
 died_0_90 |         0          1 |     Total
-----------+----------------------+----------
         0 |   360,765     68,942 |   429,707
           |     98.86      94.21 |     98.08
-----------+----------------------+----------
         1 |     4,167      4,239 |     8,406
           |      1.14       5.79 |      1.92
-----------+----------------------+----------
     Total |   364,932     73,181 |   438,113
           |    100.00     100.00 |    100.00

. cs died_0_90 infected if wave==2 & ttstudyend>=0

                 |        infected        |
                 |   Exposed   Unexposed  |      Total
-----------------+------------------------+-----------
           Cases |      4239        4167  |       8406
        Noncases |     68942      360765  |     429707
-----------------+------------------------+-----------
           Total |     73181      364932  |     438113
                 |                        |
            Risk |  .0579249    .0114186  |   .0191868
                 |                        |
                 |      Point estimate    |    [95% conf. interval]
                 |------------------------+------------------------
 Risk difference |         .0465063       |    .0447791    .0482335
      Risk ratio |         5.072868       |    4.864157    5.290533
 Attr. frac. ex. |         .8028728       |    .7944145    .8109831
```

```
 Attr. frac. pop |         .4048748       |
                 +-----------------------------------------------
                               chi2(1) =  7005.81  Pr>chi2 = 0.0000

.
. * 180-day mortality for cases and comparators who are alive on day 91
. tab died_91_180 infected if alive_day91_casecontrol==1 & wave==2 &
ttstudyend>=91, co

+-------------------+
| Key               |
|-------------------|
|     frequency     |
| column percentage |
+-------------------+

died_91_18 |       infected
         0 |         0          1 |     Total
-----------+----------------------+----------
         0 |   336,264     68,230 |   404,494
           |     98.93      98.97 |     98.94
-----------+----------------------+----------
         1 |     3,633        712 |     4,345
           |      1.07       1.03 |      1.06
-----------+----------------------+----------
     Total |   339,897     68,942 |   408,839
           |    100.00     100.00 |    100.00

. cs died_91_180 infected if alive_day91_casecontrol==1 & wave==2 &
ttstudyend>=91

                 |        infected        |
                 |   Exposed   Unexposed  |      Total
-----------------+------------------------+-----------
           Cases |       712        3633  |       4345
        Noncases |     68230      336264  |     404494
-----------------+------------------------+-----------
           Total |     68942      339897  |     408839
                 |                        |
            Risk |  .0103275    .0106885  |   .0106277
                 |                        |
                 |      Point estimate    |    [95% conf. interval]
                 |------------------------+------------------------
 Risk difference |        -.000361        |   -.0011911    .0004691
      Risk ratio |        .9662245        |    .8920176    1.046605
 Prev. frac. ex. |        .0337755        |   -.0466047    .1079824
 Prev. frac. pop |        .0056955        |
                 +-----------------------------------------------
                               chi2(1) =     0.71  Pr>chi2 = 0.3993

.
. * 365-day mortality for cases and comparators who are alive on day 181
& not censored
. tab died_181_365 infected if alive_day181_casecontrol==1 & wave==2 &
```

```
ttstudyend>=181, co

+-------------------+
| Key               |
|-------------------|
|     frequency     |
| column percentage |
+-------------------+

died_181_3 |       infected
        65 |         0          1 |     Total
-----------+----------------------+----------
         0 |   326,114     67,092 |   393,206
           |     97.99      98.33 |     98.05
-----------+----------------------+----------
         1 |     6,695      1,137 |     7,832
           |      2.01       1.67 |      1.95
-----------+----------------------+----------
     Total |   332,809     68,229 |   401,038
           |    100.00     100.00 |    100.00

. cs died_181_365 infected if alive_day181_casecontrol==1 & wave==2 &
ttstudyend>=181

                 |        infected        |
                 |   Exposed   Unexposed  |      Total
-----------------+------------------------+-----------
           Cases |      1137        6695  |       7832
        Noncases |     67092      326114  |     393206
-----------------+------------------------+-----------
           Total |     68229      332809  |     401038
                 |                        |
            Risk |  .0166645    .0201166  |   .0195293
                 |                        |
                 |      Point estimate    |    [95% conf. interval]
                 |------------------------+------------------------
 Risk difference |        -.0034522       |   -.0045246   -.0023797
      Risk ratio |         .8283921       |    .7783378    .8816653
 Prev. frac. ex. |         .1716079       |    .1183347    .2216622
 Prev. frac. pop |         .0291958       |
                 +-------------------------------------------------
                               chi2(1) =    35.24  Pr>chi2 = 0.0000

.
. * 730-day mortality for cases and comparators who are alive on day 366
& not censored
. tab died_366_730 infected if alive_day366_casecontrol==1 & wave==2 &
ttstudyend>=366, co

+-------------------+
| Key               |
|-------------------|
|     frequency     |
| column percentage |
```

```
+-------------------+

died_366_7 |       infected
        30 |         0          1 |     Total
-----------+----------------------+----------
         0 |   313,659     66,001 |   379,660 
           |     97.79      98.37 |     97.89 
-----------+----------------------+----------
         1 |     7,098      1,091 |     8,189 
           |      2.21       1.63 |      2.11 
-----------+----------------------+----------
     Total |   320,757     67,092 |   387,849 
           |    100.00     100.00 |    100.00 

. cs died_366_730 infected if alive_day366_casecontrol==1 & wave==2 &
ttstudyend>=366

                 |        infected         |
                 |   Exposed   Unexposed  |      Total
-----------------+------------------------+-----------
           Cases |      1091        7098  |       8189
        Noncases |     66001      313659  |     379660
-----------------+------------------------+-----------
           Total |     67092      320757  |     387849
                 |                        |
            Risk |  .0162613    .0221289  |   .0211139
                 |                        |
                 |      Point estimate    |    [95% conf. interval]
                 |------------------------+-----------------------
 Risk difference |        -.0058676       |   -.0069517   -.0047836 
      Risk ratio |         .7348423       |    .6898441    .7827757 
 Prev. frac. ex. |         .2651577       |    .2172243    .3101559 
 Prev. frac. pop |         .0458683       |
                 +-----------------------------------------------
                               chi2(1) =    92.43  Pr>chi2 = 0.0000


.
. *-----------
. * Wave 3
. *-----------
.
. * 90-day mortality for cases and comparators who are alive on day 1
. tab died_0_90 infected if wave==3 & ttstudyend>=0, co

+-------------------+
| Key               |
|-------------------|
|     frequency     |
| column percentage |
+-------------------+

           |       infected
 died_0_90 |         0          1 |     Total
-----------+----------------------+----------
```

```
          0 |   565,260    108,445 |   673,705 
            |     99.00      94.67 |     98.28 
-----------+----------------------+----------
          1 |     5,685      6,101 |    11,786 
            |      1.00       5.33 |      1.72 
-----------+----------------------+----------
      Total |   570,945    114,546 |   685,491 
            |    100.00     100.00 |    100.00 

. cs died_0_90 infected if wave==3 & ttstudyend>=0

                 |        infected        |
                 |   Exposed   Unexposed  |      Total
-----------------+------------------------+-----------
           Cases |      6101        5685  |      11786
        Noncases |    108445      565260  |     673705
-----------------+------------------------+-----------
           Total |    114546      570945  |     685491
                 |                        |
            Risk |  .0532624    .0099572  |   .0171935
                 |                        |
                 |      Point estimate    |    [95% conf. interval]
                 |------------------------+------------------------
 Risk difference |         .0433053       |    .0419796    .0446309
      Risk ratio |         5.349152       |    5.162236    5.542835
 Attr. frac. ex. |         .8130545       |    .8062855    .8195869
 Attr. frac. pop |         .4208761       |
                 +-------------------------------------------------
                               chi2(1) = 10588.18  Pr>chi2 = 0.0000

. 
. * 180-day mortality for cases and comparators who are alive on day 91
. tab died_91_180 infected if alive_day91_casecontrol==1 & wave==3 &
ttstudyend>=91, co

+-------------------+
| Key               |
|-------------------|
|     frequency     |
| column percentage |
+-------------------+

died_91_18 |       infected
         0 |         0          1 |     Total
-----------+----------------------+----------
         0 |   530,558    107,421 |   637,979 
           |     99.13      99.06 |     99.12 
-----------+----------------------+----------
         1 |     4,664      1,020 |     5,684 
           |      0.87       0.94 |      0.88 
-----------+----------------------+----------
     Total |   535,222    108,441 |   643,663 
           |    100.00     100.00 |    100.00 
```

```
. cs died_91_180 infected if alive_day91_casecontrol==1 & wave==3 &
ttstudyend>=91

                 |        infected         |
                 |   Exposed   Unexposed   |      Total
-----------------+-------------------------+-----------
           Cases |      1020        4664   |       5684
        Noncases |    107421      530558   |     637979
-----------------+-------------------------+-----------
           Total |    108441      535222   |     643663
                 |                         |
            Risk |   .009406    .0087141   |   .0088307
                 |                         |
                 |      Point estimate     |    [95% conf. interval]
                 |-------------------------+-----------------------
 Risk difference |         .0006919        |    .0000657     .001318
      Risk ratio |         1.079399        |    1.009012    1.154696
 Attr. frac. ex. |         .0735587        |    .0089317    .1339714
 Attr. frac. pop |         .0132002        |
                 +-------------------------------------------------
                               chi2(1) =     4.93  Pr>chi2 = 0.0264

.
. * 365-day mortality for cases and comparators who are alive on day 181
& not censored
. tab died_181_365 infected if alive_day181_casecontrol==1 & wave==3 &
ttstudyend>=181, co

+-------------------+
| Key               |
|-------------------|
|     frequency     |
| column percentage |
+-------------------+

died_181_3 |       infected
        65 |         0          1 |     Total
-----------+----------------------+----------
         0 |   514,224    105,580 |   619,804
           |     97.83      98.29 |     97.91
-----------+----------------------+----------
         1 |    11,398      1,841 |    13,239
           |      2.17       1.71 |      2.09
-----------+----------------------+----------
     Total |   525,622    107,421 |   633,043
           |    100.00     100.00 |    100.00

. cs died_181_365 infected if alive_day181_casecontrol==1 & wave==3 &
ttstudyend>=181

                 |        infected         |
                 |   Exposed   Unexposed   |      Total
-----------------+-------------------------+-----------
           Cases |      1841       11398   |      13239
```

```
        Noncases |    105580      514224  |     619804
-----------------+------------------------+-----------
           Total |    107421      525622  |     633043
                 |                        |
            Risk |  .0171382    .0216848  |   .0209133
                 |                        |
                 |      Point estimate    |    [95% conf. interval]
                 |------------------------+------------------------
 Risk difference |       -.0045466        |   -.0054169   -.0036763
      Risk ratio |        .7903318        |    .7526963    .8298492
 Prev. frac. ex. |        .2096682        |    .1701508    .2473037
 Prev. frac. pop |        .0355786        |
                 +-------------------------------------------------
                               chi2(1) =    90.05  Pr>chi2 = 0.0000

.
. * 730-day mortality for cases and comparators who are alive on day 366
& not censored
. tab died_366_730 infected if alive_day366_casecontrol==1 & wave==3 &
ttstudyend>=366, co

+-------------------+
| Key               |
|-------------------|
|     frequency     |
| column percentage |
+-------------------+

died_366_7 |       infected
        30 |         0          1 |     Total
-----------+----------------------+----------
         0 |   459,782     96,344 |   556,126
           |     98.99      99.26 |     99.04
-----------+----------------------+----------
         1 |     4,689        720 |     5,409
           |      1.01       0.74 |      0.96
-----------+----------------------+----------
     Total |   464,471     97,064 |   561,535
           |    100.00     100.00 |    100.00

. cs died_366_730 infected if alive_day366_casecontrol==1 & wave==3 &
ttstudyend>=366

                 |         infected       |
                 |   Exposed   Unexposed  |      Total
-----------------+------------------------+-----------
           Cases |       720        4689  |       5409
        Noncases |     96344      459782  |     556126
-----------------+------------------------+-----------
           Total |     97064      464471  |     561535
                 |                        |
            Risk |  .0074178    .0100954  |   .0096325
                 |                        |
                 |      Point estimate    |    [95% conf. interval]
```

```
                 |------------------------+------------------------
 Risk difference |        -.0026776       |   -.0032892    -.002066
      Risk ratio |         .7347721       |     .679539    .7944946
 Prev. frac. ex. |         .2652279       |    .2055054     .320461
 Prev. frac. pop |         .0458459       |
                 +-------------------------------------------------
                               chi2(1) =    60.34  Pr>chi2 = 0.0000

.
.
. *---------------------------------------------------------
. * Overall for comparators by later infection status
. *---------------------------------------------------------
.
. tab isfutureinfectedcontrol case , co

+-------------------+
| Key               |
|-------------------|
|     frequency     |
| column percentage |
+-------------------+

indicator: |
    is the |
patienticn |
        an |     indicator: is
uninfected |  `index_dt` the date
control in | of the first positive
  the 25:1 | covid-19 test result
   matched |          for
 cohort wh |         0          1 |     Total
-----------+----------------------+----------
         0 |   900,097    208,061 | 1,108,158
           |     86.76     100.00 |     88.97
-----------+----------------------+----------
         1 |   137,326          0 |   137,326
           |     13.24       0.00 |     11.03
-----------+----------------------+----------
     Total | 1,037,423    208,061 | 1,245,484
           |    100.00     100.00 |    100.00

. tab isfutureinfectedcontrol case if alive_day91, co

+-------------------+
| Key               |
|-------------------|
|     frequency     |
| column percentage |
+-------------------+

indicator: |
    is the |
patienticn |
```

```
        an |     indicator: is
uninfected |  `index_dt` the date
control in | of the first positive
  the 25:1 | covid-19 test result
   matched |          for
 cohort wh |         0          1 |     Total
-----------+----------------------+----------
         0 |   889,988    195,646 | 1,085,634 
           |     86.74     100.00 |     88.86 
-----------+----------------------+----------
         1 |   136,036          0 |   136,036 
           |     13.26       0.00 |     11.14 
-----------+----------------------+----------
     Total | 1,026,024    195,646 | 1,221,670 
           |    100.00     100.00 |    100.00 

. tab isfutureinfectedcontrol case if alive_day181, co

+-------------------+
| Key               |
|-------------------|
|     frequency     |
| column percentage |
+-------------------+

indicator: |
    is the |
patienticn |
        an |     indicator: is
uninfected |  `index_dt` the date
control in | of the first positive
  the 25:1 | covid-19 test result
   matched |          for
 cohort wh |         0          1 |     Total
-----------+----------------------+----------
         0 |   881,032    193,602 | 1,074,634 
           |     86.74     100.00 |     88.86 
-----------+----------------------+----------
         1 |   134,677          0 |   134,677 
           |     13.26       0.00 |     11.14 
-----------+----------------------+----------
     Total | 1,015,709    193,602 | 1,209,311 
           |    100.00     100.00 |    100.00 

. tab isfutureinfectedcontrol case if alive_day366, co

+-------------------+
| Key               |
|-------------------|
|     frequency     |
| column percentage |
+-------------------+

indicator: |
```

```
    is the |
patienticn |
        an |     indicator: is
uninfected |  `index_dt` the date
control in | of the first positive
  the 25:1 | covid-19 test result
   matched |          for
 cohort wh |         0          1 |     Total
-----------+----------------------+----------
         0 |   823,199    181,657 | 1,004,856 
           |     86.70     100.00 |     88.84 
-----------+----------------------+----------
         1 |   126,254          0 |   126,254 
           |     13.30       0.00 |     11.16 
-----------+----------------------+----------
     Total |   949,453    181,657 | 1,131,110 
           |    100.00     100.00 |    100.00 

.
. * 90-day mortality for comparators by later infection status who are
alive on index date
. tab died_0_90 isfutureinfectedcontrol if infected==0 & ttstudyend>=0,
co

+-------------------+
| Key               |
|-------------------|
|     frequency     |
| column percentage |
+-------------------+

           |   indicator: is the
           |     patienticn an
           | uninfected control in
           |   the 25:1 matched
           |       cohort wh
 died_0_90 |         0          1 |     Total
-----------+----------------------+----------
         0 |   889,988    136,036 | 1,026,024 
           |     98.88      99.06 |     98.90 
-----------+----------------------+----------
         1 |    10,109      1,290 |    11,399 
           |      1.12       0.94 |      1.10 
-----------+----------------------+----------
     Total |   900,097    137,326 | 1,037,423 
           |    100.00     100.00 |    100.00 

. cs died_0_90 isfutureinfectedcontrol if infected==0 & ttstudyend>=0

                 | indicator: is the      |
                 | patienticn an          |
                 | uninfected control in  |
                 | the 25:1 matched       |
                 | cohort wh              |
```

```
                 |   Exposed   Unexposed  |      Total
-----------------+------------------------+-----------
           Cases |      1290       10109  |      11399
        Noncases |    136036      889988  |    1026024
-----------------+------------------------+-----------
           Total |    137326      900097  |    1037423
                 |                        |
            Risk |  .0093937     .011231  |   .0109878
                 |                        |
                 |      Point estimate    |    [95% conf. interval]
                 |------------------------+------------------------
 Risk difference |        -.0018373       |    -.002392   -.0012826
      Risk ratio |         .8364078       |     .789538    .8860599
 Prev. frac. ex. |         .1635922       |    .1139401     .210462
 Prev. frac. pop |         .0216551       |
                 +-------------------------------------------------
                               chi2(1) =    37.01  Pr>chi2 = 0.0000

.
. * 180-day mortality for comparators by later infection status who are
alive on day 91
. tab died_91_180 isfutureinfectedcontrol if infected==0 &
alive_day91==1 & ttstudyend>=91, co

+-------------------+
| Key               |
|-------------------|
|     frequency     |
| column percentage |
+-------------------+

           |   indicator: is the
           |     patienticn an
           | uninfected control in
           |   the 25:1 matched
died_91_18 |       cohort wh
         0 |         0          1 |     Total
-----------+----------------------+----------
         0 |   881,032    134,677 | 1,015,709
           |     98.99      99.00 |     98.99
-----------+----------------------+----------
         1 |     8,956      1,359 |    10,315
           |      1.01       1.00 |      1.01
-----------+----------------------+----------
     Total |   889,988    136,036 | 1,026,024
           |    100.00     100.00 |    100.00

. cs died_91_180 isfutureinfectedcontrol if infected==0 & alive_day91==1
& ttstudyend>=91

                 | indicator: is the      |
                 | patienticn an          |
                 | uninfected control in  |
                 | the 25:1 matched       |
```

```
                 | cohort wh              |
                 |   Exposed   Unexposed  |      Total
-----------------+------------------------+-----------
           Cases |      1359        8956  |      10315
        Noncases |    134677      881032  |    1015709
-----------------+------------------------+-----------
           Total |    136036      889988  |    1026024
                 |                        |
            Risk |    .00999    .0100631  |   .0100534
                 |                        |
                 |      Point estimate    |    [95% conf. interval]
                 |------------------------+-----------------------
 Risk difference |       -.0000731        |   -.0006408    .0004946
      Risk ratio |        .9927403        |    .9379505    1.050731
 Prev. frac. ex. |        .0072597        |   -.0507307    .0620495
 Prev. frac. pop |        .0009625        |
                 +-----------------------------------------------
                              chi2(1) =     0.06  Pr>chi2 = 0.8014

.
. * 365-day mortality for comparators by later infection status who are
alive on day 181 & not censored
. tab died_181_365 isfutureinfectedcontrol if infected==0 &
alive_day181==1 & ttstudyend>=181, co

+-------------------+
| Key               |
|-------------------|
|     frequency     |
| column percentage |
+-------------------+

           |   indicator: is the
           |     patienticn an
           | uninfected control in
           |   the 25:1 matched
died_181_3 |       cohort wh
        65 |         0          1 |     Total
-----------+----------------------+----------
         0 |   862,422    130,652 |   993,074
           |     97.89      97.01 |     97.77
-----------+----------------------+----------
         1 |    18,610      4,025 |    22,635
           |      2.11       2.99 |      2.23
-----------+----------------------+----------
     Total |   881,032    134,677 | 1,015,709
           |    100.00     100.00 |    100.00

. cs died_181_365 isfutureinfectedcontrol if infected==0 &
alive_day181==1 & ttstudyend>=181

                 | indicator: is the      |
                 | patienticn an          |
                 | uninfected control in  |
```

```
                 | the 25:1 matched       |
                 | cohort wh              |
                 |   Exposed   Unexposed  |      Total
-----------------+------------------------+------------
           Cases |      4025       18610  |      22635
        Noncases |    130652      862422  |     993074
-----------------+------------------------+------------
           Total |    134677      881032  |    1015709
                 |                        |
            Risk |  .0298863     .021123  |   .0222849
                 |                        |
                 |      Point estimate    |    [95% conf. interval]
                 |------------------------+------------------------
 Risk difference |         .0087634       |    .0078057     .009721
      Risk ratio |         1.414874       |    1.368145    1.463199
 Attr. frac. ex. |         .2932233       |    .2690833     .316566
 Attr. frac. pop |         .0521415       |
                 +-------------------------------------------------
                               chi2(1) =   411.75  Pr>chi2 = 0.0000

.
. * 730-day mortality for comparators by later infection status who are
alive on day 366 & not censored
. tab died_366_730 isfutureinfectedcontrol if infected==0 &
alive_day366==1 & ttstudyend>=366, co

+-------------------+
| Key               |
|-------------------|
|     frequency     |
| column percentage |
+-------------------+

           |   indicator: is the
           |     patienticn an
           | uninfected control in
           |   the 25:1 matched
died_366_7 |       cohort wh
        30 |         0          1 |     Total
-----------+----------------------+----------
         0 |   809,909    121,905 |   931,814
           |     98.39      96.56 |     98.14
-----------+----------------------+----------
         1 |    13,290      4,349 |    17,639
           |      1.61       3.44 |      1.86
-----------+----------------------+----------
     Total |   823,199    126,254 |   949,453
           |    100.00     100.00 |    100.00

. cs died_366_730 isfutureinfectedcontrol if infected==0 &
alive_day366==1 & ttstudyend>=366

                 | indicator: is the      |
                 | patienticn an          |
```

```
                 | uninfected control in  |
                 | the 25:1 matched       |
                 | cohort wh              |
                 |   Exposed   Unexposed  |      Total
-----------------+------------------------+------------
           Cases |      4349       13290  |      17639
        Noncases |    121905      809909  |     931814
-----------------+------------------------+------------
           Total |    126254      823199  |     949453
                 |                        |
            Risk |  .0344464    .0161443  |   .0185781
                 |                        |
                 |      Point estimate    |    [95% conf. interval]
                 |------------------------+------------------------
 Risk difference |         .0183021       |    .0172599    .0193443
      Risk ratio |         2.133655       |    2.062901    2.206835
 Attr. frac. ex. |         .5313206       |    .5152457    .5468624
 Attr. frac. pop |         .1310002       |
                 +-------------------------------------------------
                               chi2(1) =  2011.05  Pr>chi2 = 0.0000

.
.
. *---------------------------------------------
. * Appendix Figure 2 - Kaplan Meier Curves
. *---------------------------------------------
.
. * 0-90 Days
. preserve

.         stset tte_0_90 , failure(died_0_90==1)

Survival-time data settings

         Failure event: died_0_90==1
Observed time interval: (0, tte_0_90]
     Exit on or before: failure

-------------------------------------------------------------------------
--
  1,245,484  total observations
        341  observations end on or before enter()
-------------------------------------------------------------------------
--
  1,245,143  observations remaining, representing
     23,473  failures in single-record/single-failure data
  110735739  total analysis time at risk and under observation
                                                At risk from t =
0
                                     Earliest observed entry t =
0
                                          Last observed exit t =
90
```

```
.         sts test infected, logrank

        Failure _d: died_0_90==1
  Analysis time _t: tte_0_90

Equality of survivor functions
Log-rank test

         |  Observed       Expected
infected |    events         events
---------+-------------------------
       0 |     11258       19660.53
       1 |     12215        3812.47
---------+-------------------------
   Total |     23473       23473.00

                 chi2(1) = 22116.70
                 Pr>chi2 =   0.0000

.         sts graph, by(infected) name(survival090itt, replace) ///
>                 legend(pos(5) ring(0) rows(2) region(lcolor(none)) ///
>                                size(small) label(1 "Comparators") 
label(2 "COVID-19 Cases"))    ///
>                 graphregion(color(white)) bgcolor(white) title("Days 
0-90", color(black) size(medium)) ///
>                 xtitle("") ///
>                 plot1opts(lpattern(solid) lcolor("0 114 178")) ///
>                 plot2opts(lpattern(dash) lcolor("230 159 0"))  ///
>                 tlabel(, labsize(small)) ylabel(, labsize(small))

        Failure _d: died_0_90==1
  Analysis time _t: tte_0_90

.         *graph save "survival090itt" "Figures\survival090itt.gph", 
replace
.         stset , clear

. restore


.
. * 91-180 Days
. preserve

.         keep if alive_day91_casecontrol==1
(74,343 observations deleted)

.         stset tte_91_180 , failure(died_91_180==1)

Survival-time data settings

         Failure event: died_91_180==1
Observed time interval: (0, tte_91_180]
     Exit on or before: failure
```

```
------------------------------------------------------------------------
--
  1,171,141  total observations
     10,502  event time missing (tte_91_180>=.)            PROBABLE
ERROR
------------------------------------------------------------------------
--
  1,160,639  observations remaining, representing
     11,558  failures in single-record/single-failure data
  208398252  total analysis time at risk and under observation
                                                At risk from t =
0
                                     Earliest observed entry t =
0
                                          Last observed exit t =
180

.         sts test infected, logrank

        Failure _d: died_91_180==1
  Analysis time _t: tte_91_180

Equality of survivor functions
Log-rank test

         |  Observed       Expected
infected |    events         events
---------+-------------------------
       0 |      9514        9610.64
       1 |      2044        1947.36
---------+-------------------------
   Total |     11558       11558.00

                   chi2(1) =   5.77
                   Pr>chi2 = 0.0163

.         sts graph, by(infected) name(survival91180itt, replace) ///
>                 legend(pos(5) ring(0) rows(2) region(lcolor(none)) ///
>                                 size(small) label(1 "Comparators")
label(2 "COVID-19 Cases"))    ///
>                 graphregion(color(white)) bgcolor(white) title("Days
91-180", color(black) size(medium)) ///
>                 xtitle("") ///
>                 plot1opts(lpattern(solid) lcolor("0 114 178")) ///
>                 plot2opts(lpattern(dash) lcolor("230 159 0"))   ///
>                 ylabel(0.9 (0.02) 1.0, labsize(small)) ///
>                 tmin(91) tlabel(90 (20) 190, labsize(small))

        Failure _d: died_91_180==1
  Analysis time _t: tte_91_180

.         *graph save "survival91180itt" "Figures\survival91180itt.gph",
replace
.         stset , clear
```

```
. restore

.
. * 181-365 Days
. preserve

.         keep if alive_day181_casecontrol==1
(86,579 observations deleted)

.         stset tte_181_365 , failure(died_181_365==1)

Survival-time data settings

         Failure event: died_181_365==1
Observed time interval: (0, tte_181_365]
     Exit on or before: failure

------------------------------------------------------------------------
--
  1,158,905  total observations
     19,710  event time missing (tte_181_365>=.)            PROBABLE
ERROR
------------------------------------------------------------------------
--
  1,139,195  observations remaining, representing
     24,026  failures in single-record/single-failure data
  412887955  total analysis time at risk and under observation
                                                 At risk from t =
0
                                      Earliest observed entry t =
0
                                           Last observed exit t =
365

.         sts test infected, logrank

        Failure _d: died_181_365==1
  Analysis time _t: tte_181_365

Equality of survivor functions
Log-rank test

         |  Observed       Expected
infected |    events         events
---------+-------------------------
       0 |     20594       19936.63
       1 |      3432        4089.37
---------+-------------------------
   Total |     24026       24026.00

                   chi2(1) = 127.36
                   Pr>chi2 = 0.0000
```

```
.         sts graph, by(infected) name(survival181365itt, replace) ///
>                 legend(pos(5) ring(0) rows(2) region(lcolor(none)) ///
>                               size(small) label(1 "Comparators")
label(2 "COVID-19 Cases"))    ///
>                 graphregion(color(white)) bgcolor(white) title("Days
181-365", color(black) size(medium)) ///
>                 xtitle("") ///
>                 plot1opts(lpattern(solid) lcolor("0 114 178")) ///
>                 plot2opts(lpattern(dash) lcolor("230 159 0"))   ///
>                 ylabel(0.9 (0.02) 1.0, labsize(small)) ///
>                 tmin(181) tlabel(180 (40) 380, labsize(small))

        Failure _d: died_181_365==1
  Analysis time _t: tte_181_365

.         *graph save "survival181365itt"
"Figures\survival181365itt.gph", replace
.         stset , clear

. restore


.
. * 366-730 Days
. preserve

.         keep if alive_day366_casecontrol==1
(157,981 observations deleted)

.         stset tte_366_730 , failure(died_366_730==1)

Survival-time data settings

         Failure event: died_366_730==1
Observed time interval: (0, tte_366_730]
     Exit on or before: failure

--------------------------------------------------------------------------
--
  1,087,503  total observations
     38,020  event time missing (tte_366_730>=.)            PROBABLE
ERROR
--------------------------------------------------------------------------
--
  1,049,483  observations remaining, representing
     17,998  failures in single-record/single-failure data
  528362986  total analysis time at risk and under observation
                                                At risk from t =
0
                                     Earliest observed entry t =
0
                                          Last observed exit t =
730

.         sts test infected, logrank
```

```
        Failure _d: died_366_730==1
  Analysis time _t: tte_366_730

Equality of survivor functions
Log-rank test

         |  Observed       Expected
infected |    events         events
---------+-------------------------
       0 |     15481       14871.89
       1 |      2517        3126.11
---------+-------------------------
   Total |     17998       17998.00

                   chi2(1) = 143.65
                   Pr>chi2 = 0.0000

.         sts graph, by(infected) name(survival366730itt, replace)  ///
>                 legend(pos(5) ring(0) rows(2) region(lcolor(none)) ///
>                                 size(small) label(1 "Comparators")
label(2 "COVID-19 Cases"))    ///
>                 graphregion(color(white)) bgcolor(white) title("Days
366-730", color(black) size(medium)) ///
>                 xtitle("") ///
>                 plot1opts(lpattern(solid) lcolor("0 114 178")) ///
>                 plot2opts(lpattern(dash) lcolor("230 159 0"))   ///
>                 ylabel(0.9 (0.02) 1.0, labsize(small)) ///
>                 tmin(366) tlabel(365 450 550 650 730, labsize(small))

        Failure _d: died_366_730==1
  Analysis time _t: tte_366_730

.         *graph save "survival366730itt"
"Figures\survival366730itt.gph", replace
.         stset , clear

. restore


.
. * 0-365 Days
. preserve

.         stset tte_0_365 , failure(died_0_365)

Survival-time data settings

         Failure event: died_0_365!=0 & died_0_365<.
Observed time interval: (0, tte_0_365]
     Exit on or before: failure

------------------------------------------------------------------------
--
  1,245,484  total observations
```

```
        341  observations end on or before enter()
--------------------------------------------------------------------------
--
  1,245,143  observations remaining, representing
     61,899  failures in single-record/single-failure data
  440719447  total analysis time at risk and under observation
                                                     At risk from t =
0
                                          Earliest observed entry t =
0
                                               Last observed exit t =
365

.         *sts test infected, logrank
.         sts graph, by(infected) name(survival0365, replace)

        Failure _d: died_0_365
  Analysis time _t: tte_0_365

.         stset , clear

. restore

.
. * 0-730 Days
. preserve

.         stset tte_0_730 , failure(died_0_730)

Survival-time data settings

         Failure event: died_0_730!=0 & died_0_730<.
Observed time interval: (0, tte_0_730]
     Exit on or before: failure

--------------------------------------------------------------------------
--
  1,245,484  total observations
        341  observations end on or before enter()
--------------------------------------------------------------------------
--
  1,245,143  observations remaining, representing
     82,056  failures in single-record/single-failure data
  598623178  total analysis time at risk and under observation
                                                     At risk from t =
0
                                          Earliest observed entry t =
0
                                               Last observed exit t =
730

.         stdescribe if infected==1

        Failure _d: died_0_730
```

```
  Analysis time _t: tte_0_730

                                   |-------------- Per subject ---------
-----|
Category                   Total        Mean         Min     Median
Max
------------------------------------------------------------------------
------
Number of subjects        207861
Number of records         207861           1           1          1
1

Entry time (first)                         0           0          0
0
Exit time (final)                   461.8972           1        471
730

Subjects with gap              0
Time on gap                    0
Time at risk            96010404    461.8972           1        471
730

Failures                   20209    .0972236           0          0
1
------------------------------------------------------------------------
------

.         stdescribe if infected==0

        Failure _d: died_0_730
  Analysis time _t: tte_0_730

                                   |-------------- Per subject ---------
-----|
Category                   Total        Mean         Min     Median
Max
------------------------------------------------------------------------
------
Number of subjects       1037282
Number of records        1037282           1           1          1
1

Entry time (first)                         0           0          0
0
Exit time (final)                   484.5479           1        476
730

Subjects with gap              0
Time on gap                    0
Time at risk           5.026e+08    484.5479           1        476
730

Failures                   61847    .0596241           0          0
1
```

```
--------------------------------------------------------------------------
------

.         *sts test infected, logrank
.         sts graph, by(infected) name(survival0730itt, replace) ///
>                 legend(pos(5) ring(0) rows(2) region(lcolor(none)) ///
>                                size(small) label(1 "Comparators")
label(2 "COVID-19 Cases"))    ///
>                 graphregion(color(white)) bgcolor(white) title("Days
0-730", color(black) size(medium)) ///
>                 xtitle("") ///
>                 plot1opts(lpattern(solid) lcolor("0 114 178")) ///
>                 plot2opts(lpattern(dash) lcolor("230 159 0"))   ///
>                 tlabel(, labsize(small)) ylabel(, labsize(small))

        Failure _d: died_0_730
  Analysis time _t: tte_0_730

.         *graph save "survival0730itt" "Figures\survival0730itt.gph",
replace
.         stset , clear

. restore

.
. *------------------------
. * Table 1: SMDs
. *------------------------
.
. * Baseline covariate balance for 5-to-1 match cohort
.
. tab infected

   infected |      Freq.     Percent        Cum.
------------+-----------------------------------
          0 |  1,037,423       83.29       83.29
          1 |    208,061       16.71      100.00
------------+-----------------------------------
      Total |  1,245,484      100.00

.
. stddiff ageatindexdate, by(infected)

--------------------------------------------------------------------------
------
             |       infected=0         |       infected=1         |
             | Mean or N     SD or (%) | Mean or N     SD or (%) |  Std
Diff
-------------+--------------------------+--------------------------+-----
------
ageatinde~te |     60.47        16.464 |     60.51         16.19 |   -
0.00286
--------------------------------------------------------------------------
------
```

```
. stddiff bmi, by(infected)

--------------------------------------------------------------------------
------
              |        infected=0          |        infected=1          |
              | Mean or N      SD or (%)  | Mean or N      SD or (%)  |  Std
Diff
--------------+---------------------------+---------------------------+-----
------
          bmi |     31.28         6.6078 |     31.34          6.3499 |   -
0.01003
--------------------------------------------------------------------------
------

. stddiff i.sex3cat, by(infected)

--------------------------------------------------------------------------
------
              |        infected=0          |        infected=1          |
              | Mean or N      SD or (%)  | Mean or N      SD or (%)  |  Std
Diff
--------------+---------------------------+---------------------------+-----
------
      sex3cat |                            |                            |
       Female |    109085         (10.5) |     21936          (10.5) |
0.00411
         Male |    914361         (88.1) |    183226          (88.1) |
      Unknown |     13977         ( 1.3) |      2899          ( 1.4) |
--------------------------------------------------------------------------
------

. stddiff i.race7cat, by(infected)

--------------------------------------------------------------------------
------
              |        infected=0          |        infected=1          |
              | Mean or N      SD or (%)  | Mean or N      SD or (%)  |  Std
Diff
--------------+---------------------------+---------------------------+-----
------
     race7cat |                            |                            |
AmericanIn~e |       9578         ( 0.9) |      1961          ( 0.9) |
0.00338
        Asian |     10536         ( 1.0) |      2081          ( 1.0) |
Black/Afri~r |    237896         (22.9) |     47645          (22.9) |
NativeHawa~d |       9704         ( 0.9) |      1944          ( 0.9) |
        White |    696309         (67.1) |    139604          (67.1) |
MultipleRace |       9869         ( 1.0) |      1975          ( 0.9) |
      Missing |     63531         ( 6.1) |     12851          ( 6.2) |
--------------------------------------------------------------------------
------

. stddiff i.ethnicity3cat, by(infected)
```

```
------------------------------------------------------------------------
------
             |       infected=0         |       infected=1         |
             | Mean or N     SD or (%)  | Mean or N     SD or (%)  |  Std
Diff
-------------+--------------------------+--------------------------+------
------
ethnicity3~t |                          |                          |
    Hispanic |     99093         ( 9.6) |     20280         ( 9.7) |
0.00668
 NotHispanic |    903000         (87.0) |    180675         (86.8) |
     Missing |     35330         ( 3.4) |      7106         ( 3.4) |
------------------------------------------------------------------------
------

. stddiff i.rurality2cat, by(infected)

------------------------------------------------------------------------
------
             |       infected=0         |       infected=1         |
             | Mean or N     SD or (%)  | Mean or N     SD or (%)  |  Std
Diff
-------------+--------------------------+--------------------------+------
------
rurality2cat |                          |                          |
    NotUrban |    294989         (28.4) |     59064         (28.4) |
0.00104
       Urban |    742434         (71.6) |    148997         (71.6) |
------------------------------------------------------------------------
------

. stddiff i.smoking4cat, by(infected)

------------------------------------------------------------------------
------
             |       infected=0         |       infected=1         |
             | Mean or N     SD or (%)  | Mean or N     SD or (%)  |  Std
Diff
-------------+--------------------------+--------------------------+------
------
 smoking4cat |                          |                          |
     Current |    131249         (12.7) |     26144         (12.6) |
0.01171
      Former |    439908         (42.4) |     88025         (42.3) |
       Never |    408648         (39.4) |     81777         (39.3) |
     Missing |     57618         ( 5.6) |     12115         ( 5.8) |
------------------------------------------------------------------------
------

. stddiff gagne, by(infected)

------------------------------------------------------------------------
------
```

```
             |       infected=0         |       infected=1         |
             | Mean or N     SD or (%) | Mean or N     SD or (%) |  Std
Diff
-------------+-------------------------+-------------------------+------
------
       gagne |     1.403        2.2383 |     1.441        2.3416 |   -
0.01655
--------------------------------------------------------------------------
------

. stddiff numipadmits, by(infected)

--------------------------------------------------------------------------
------
             |       infected=0         |       infected=1         |
             | Mean or N     SD or (%) | Mean or N     SD or (%) |  Std
Diff
-------------+-------------------------+-------------------------+------
------
 numipadmits |     .3567        1.2623 |     .3585        1.2094 |   -
0.00152
--------------------------------------------------------------------------
------

. stddiff util_numpcstops, by(infected)

--------------------------------------------------------------------------
------
             |       infected=0         |       infected=1         |
             | Mean or N     SD or (%) | Mean or N     SD or (%) |  Std
Diff
-------------+-------------------------+-------------------------+------
------
util_numpc~s |     8.278        10.421 |     8.551        9.6582 |   -
0.02719
--------------------------------------------------------------------------
------

. stddiff util_numscstops, by(infected)

--------------------------------------------------------------------------
------
             |       infected=0         |       infected=1         |
             | Mean or N     SD or (%) | Mean or N     SD or (%) |  Std
Diff
-------------+-------------------------+-------------------------+------
------
util_numsc~s |     13.44         15.34 |     13.88        14.141 |   -
0.02959
--------------------------------------------------------------------------
------

. stddiff util_nummhstops, by(infected)
```

```
------------------------------------------------------------------------
------
             |      infected=0         |      infected=1         |
             | Mean or N     SD or (%) | Mean or N     SD or (%) |  Std
Diff
-------------+-------------------------+-------------------------+------
------
util_nummh~s |      7.78        21.931 |     7.868        22.644 |   -
0.00392
------------------------------------------------------------------------
------

. stddiff i.immuno, by(infected)

------------------------------------------------------------------------
------
             |      infected=0         |      infected=1         |
             | Mean or N     SD or (%) | Mean or N     SD or (%) |  Std
Diff
-------------+-------------------------+-------------------------+------
------
      immuno |                         |                         |
           0 |    936167        (90.2) |    187748        (90.2) |
0.00009
           1 |    101256        ( 9.8) |     20313        ( 9.8) |
------------------------------------------------------------------------
------

. stddiff i.clcatindexdate, by(infected)

------------------------------------------------------------------------
------
             |      infected=0         |      infected=1         |
             | Mean or N     SD or (%) | Mean or N     SD or (%) |  Std
Diff
-------------+-------------------------+-------------------------+------
------
clcatindex~e |                         |                         |
           0 |   1027879        (99.1) |    205899        (99.0) |
0.01210
           1 |      9544        ( 0.9) |      2162        ( 1.0) |
------------------------------------------------------------------------
------

. stddiff i.nosos11cat, by(infected)

------------------------------------------------------------------------
------
             |      infected=0         |      infected=1         |
             | Mean or N     SD or (%) | Mean or N     SD or (%) |  Std
Diff
-------------+-------------------------+-------------------------+------
------
  nosos11cat |                         |                         |
```

```
          1 |     26035         ( 2.5) |      5694         ( 2.7) |
0.03131
          2 |     45530         ( 4.4) |      9582         ( 4.6) |
          3 |     60994         ( 5.9) |     12431         ( 6.0) |
          4 |     75933         ( 7.3) |     15159         ( 7.3) |
          5 |     90104         ( 8.7) |     17707         ( 8.5) |
          6 |    105586         (10.2) |     20526         ( 9.9) |
          7 |    119913         (11.6) |     23431         (11.3) |
          8 |    136532         (13.2) |     26893         (12.9) |
          9 |    157063         (15.1) |     31294         (15.0) |
         10 |    194278         (18.7) |     40555         (19.5) |
         99 |     25455         ( 2.5) |      4789         ( 2.3) |
-------------------------------------------------------------------------
------

. stddiff i.canscore7cat, by(infected)

-------------------------------------------------------------------------
------
             |       infected=0          |       infected=1          |
             | Mean or N     SD or (%) | Mean or N     SD or (%) |  Std
Diff
-------------+-------------------------+-------------------------+-----
------
canscore7cat |                         |                         |
          1 |    170471         (16.4) |     34417         (16.5) |
0.02458
          2 |    160008         (15.4) |     31900         (15.3) |
          3 |    194475         (18.7) |     38219         (18.4) |
          4 |    235676         (22.7) |     46759         (22.5) |
          5 |    153135         (14.8) |     30638         (14.7) |
          6 |    102842         ( 9.9) |     22072         (10.6) |
         99 |     20816         ( 2.0) |      4056         ( 1.9) |
-------------------------------------------------------------------------
------

. stddiff neareststa3ndistance, by(infected)

-------------------------------------------------------------------------
------
             |       infected=0          |       infected=1          |
             | Mean or N     SD or (%) | Mean or N     SD or (%) |  Std
Diff
-------------+-------------------------+-------------------------+-----
------
nearestst~ce |     35.75        35.103 |     35.59        36.521 |
0.00443
-------------------------------------------------------------------------
------

.
.
. *******
. * ITT *
```

```
. *******
.
. use Data\mortality_varforcox_20230516, clear
(Created in file
'P:\ORD_Ioannou_202104007D\MethodsGroup\Programs\CORC_DataExplor)

.
. *-------------------------------------
. * Table 2: ITT Unadjusted
. *-------------------------------------
.
. * Overall
. preserve

.         sort uniq_patid

.         stset tte_0_730, failure(died_0_730) id(uniq_patid)

Survival-time data settings

           ID variable: uniq_patid
         Failure event: died_0_730!=0 & died_0_730<.
Observed time interval: (tte_0_730[_n-1], tte_0_730]
     Exit on or before: failure

------------------------------------------------------------------------
--
  1,245,484  total observations
        341  observations end on or before enter()
------------------------------------------------------------------------
--
  1,245,143  observations remaining, representing
  1,245,143  subjects
     82,056  failures in single-failure-per-subject data
  598623178  total analysis time at risk and under observation
                                                At risk from t =
0
                                     Earliest observed entry t =
0
                                          Last observed exit t =
730

.         stcox infected, efron vce(cluster patienticn)
strata(matchgroupnumber)

        Failure _d: died_0_730
  Analysis time _t: tte_0_730
       ID variable: uniq_patid

Iteration 0:   log pseudolikelihood = -143313.92
Iteration 1:   log pseudolikelihood = -141294.91
Iteration 2:   log pseudolikelihood =  -141214.1
Iteration 3:   log pseudolikelihood = -141214.06
Refining estimates:
```

```
Iteration 0:   log pseudolikelihood = -141214.06

Stratified Cox regression with Efron method for ties
Strata variable: matchgroupnumber

No. of subjects = 1,245,143                          Number of obs =
1,245,143
No. of failures =    82,056
Time at risk    = 598623178
                                                     Wald chi2(1)  =
5361.40
Log pseudolikelihood = -141214.06                    Prob > chi2   =
0.0000

                     (Std. err. adjusted for 1,091,939 clusters in
patienticn)
------------------------------------------------------------------------
------
             |               Robust
          _t | Haz. ratio   std. err.      z    P>|z|     [95% conf.
interval]
-------------+----------------------------------------------------------
------
    infected |   1.748196   .0133364    73.22   0.000     1.722252
1.774532
------------------------------------------------------------------------
------

. restore

.
. * By Period
.
. * stset data
. sort uniq_patid

. recode tte_0_730 (0=0.5)
(341 changes made to tte_0_730)

. stset tte_0_730, failure(died_0_730) id(uniq_patid)

Survival-time data settings

           ID variable: uniq_patid
         Failure event: died_0_730!=0 & died_0_730<.
Observed time interval: (tte_0_730[_n-1], tte_0_730]
     Exit on or before: failure

------------------------------------------------------------------------
--
  1,245,484  total observations
          0  exclusions
------------------------------------------------------------------------
--
```

```
  1,245,484  observations remaining, representing
  1,245,484  subjects
     82,397  failures in single-failure-per-subject data
  598623349  total analysis time at risk and under observation
                                                At risk from t =
0
                                     Earliest observed entry t =
0
                                          Last observed exit t =
730

. stsplit period, at(0 90 180 365 730)
(3,562,091 observations (episodes) created)

.
. stdescribe if infected==1

        Failure _d: died_0_730
  Analysis time _t: tte_0_730
       ID variable: uniq_patid

                                   |-------------- Per subject ---------
-----|
Category                   Total        Mean         Min     Median
Max
----------------------------------------------------------------------
------
Number of subjects        208061
Number of records         778966    3.743931           1          4
4

Entry time (first)                         0           0          0
0
Exit time (final)                   461.4536          .5        471
730

Subjects with gap              0
Time on gap                    0           .           .          .
.
Time at risk            96010504    461.4536          .5        471
730

Failures                   20409    .0980914           0          0
1
----------------------------------------------------------------------
------

. stdescribe if infected==0

        Failure _d: died_0_730
  Analysis time _t: tte_0_730
       ID variable: uniq_patid

                                   |-------------- Per subject ---------
```

```
-----|
Category                      Total        Mean         Min     Median
Max
---------------------------------------------------------------------------
------
Number of subjects        1037423
Number of records         4028609    3.883285           1          4
4

Entry time (first)                          0           0          0
0
Exit time (final)                    484.4821          .5        476
730

Subjects with gap               0
Time on gap                     0           .           .          .
.
Time at risk            5.026e+08    484.4821          .5        476
730

Failures                    61988    .0597519           0          0
1
---------------------------------------------------------------------------
------

.
. * create new interaction variables after splitting data
. gen timeinfected_0_90 = infected * (tte_0_730<=90)

. gen timeinfected_91_180 = infected * (tte_0_730>90 & tte_0_730<=180)

. gen timeinfected_181_365 = infected * (tte_0_730>180 & tte_0_730<=365)

. gen timeinfected_366_730 = infected * (tte_0_730>365 & tte_0_730<=730)

.
. * Cox Model
. stcox   timeinfected_0_90 timeinfected_91_180 timeinfected_181_365
///
>                  timeinfected_366_730
///
>                  , efron vce(cluster patienticn)
strata(matchgroupnumber)

        Failure _d: died_0_730
  Analysis time _t: tte_0_730
       ID variable: uniq_patid

Iteration 0:   log pseudolikelihood =  -143924.5
Iteration 1:   log pseudolikelihood = -137775.88
Iteration 2:   log pseudolikelihood = -135674.34
Iteration 3:   log pseudolikelihood = -135605.93
Iteration 4:   log pseudolikelihood = -135605.93
Refining estimates:
```

```
Iteration 0:   log pseudolikelihood = -135605.93

Stratified Cox regression with Efron method for ties
Strata variable: matchgroupnumber

No. of subjects =   1,245,484                       Number of obs =
4,807,575
No. of failures =      82,397
Time at risk    = 598623348.5
                                                    Wald chi2(4)  =
23276.74
Log pseudolikelihood = -135605.93                   Prob > chi2   =
0.0000

                             (Std. err. adjusted for 1,092,248 clusters
in patienticn)
-----------------------------------------------------------------------
--------------
                     |               Robust
                  _t | Haz. ratio   std. err.      z    P>|z|     [95%
conf. interval]
---------------------+-------------------------------------------------
--------------
   timeinfected_0_90 |   5.752757   .0664537   151.47   0.000
5.623973    5.884489
 timeinfected_91_180 |   1.054258   .0234888     2.37   0.018
1.009212    1.101315
timeinfected_181_365 |   .8058384   .0141301   -12.31   0.000
.7786145    .8340142
timeinfected_366_730 |   .7610328   .0152282   -13.65   0.000
.7317639    .7914724
-----------------------------------------------------------------------
--------------


.
. *-----------------------------------------------
. * Subgroup Analyses - Hospitalization, Adj.
. *-----------------------------------------------
.
. * Subgroup analysis for hospitalized/not hospitalized & adjust for
. * imbalanced hospitalization covariates (SMDs>0.1 for hospitalization
subgroups)
.
.
. * Hospitalized Patients
. local imbalanced ageatindexdate bmi i.ethnicity3cat i.smoking4cat ///
>                                 gagne numipadmits util_numpcstops
util_numscstops ///
>                                 i.nosos11cat i.canscore7cat
neareststa3ndistance

.
. stcox   timeinfected_0_90 timeinfected_91_180 timeinfected_181_365
///
```

```
>                  timeinfected_366_730 `imbalanced'  ///
>                  if index_hospitalization==1 ///
>                  , efron vce(cluster patienticn)
strata(matchgroupnumber)

        Failure _d: died_0_730
  Analysis time _t: tte_0_730
       ID variable: uniq_patid

Iteration 0:   log pseudolikelihood = -1440.6393
Iteration 1:   log pseudolikelihood = -1172.9772
Iteration 2:   log pseudolikelihood = -1161.3109
Iteration 3:   log pseudolikelihood = -1161.1456
Iteration 4:   log pseudolikelihood = -1161.1456
Refining estimates:
Iteration 0:   log pseudolikelihood = -1161.1456

Stratified Cox regression with Efron method for ties
Strata variable: matchgroupnumber

No. of subjects =     59,528                          Number of obs =
196,626
No. of failures =     17,336
Time at risk    = 23,463,828
                                                      Wald chi2(32) =
592.53
Log pseudolikelihood = -1161.1456                     Prob > chi2   =
0.0000

                                (Std. err. adjusted for 58,791 clusters
in patienticn)
------------------------------------------------------------------------
--------------
                     |               Robust
                  _t | Haz. ratio   std. err.      z    P>|z|     [95%
conf. interval]
---------------------+--------------------------------------------------
--------------
   timeinfected_0_90 |   1.766286   .1029108     9.76   0.000
1.575675    1.979956
 timeinfected_91_180 |   .4206626    .048798    -7.46   0.000
.3351138    .5280506
timeinfected_181_365 |   .6015507    .060755    -5.03   0.000
.4935181    .7332319
timeinfected_366_730 |   .4963705   .0635028    -5.47   0.000
.3862852    .6378283
      ageatindexdate |   1.041538    .003405    12.45   0.000
1.034886    1.048233
                 bmi |   .9774227   .0044998    -4.96   0.000
.9686428    .9862821
                     |
       ethnicity3cat |
         NotHispanic  |   .8922326   .1242112    -0.82   0.413
.6791707    1.172134
```

Missing	.768472	.183414	-1.10	0.270	.4813586	1.226838
smoking4cat						
Former	1.089274	.1145585	0.81	0.416	.8863732	1.338621
Never	1.222564	.1366328	1.80	0.072	.9820691	1.521953
Missing	1.467125	.2287238	2.46	0.014	1.08085	1.991448
gagne	1.105934	.0137585	8.09	0.000	1.079294	1.133232
numipadmits	.9977639	.0169808	-0.13	0.895	.9650312	1.031607
util_numpcstops	.9978743	.0024727	-0.86	0.390	.9930396	1.002733
util_numscstops	.996118	.0020255	-1.91	0.056	.9921559	1.000096
nosos11cat						
2	1.890463	1.139095	1.06	0.291	.580334	6.158263
3	1.235832	.6985895	0.37	0.708	.4081253	3.742187
4	.8904004	.523422	-0.20	0.843	.2813238	2.818151
5	1.623904	.9557067	0.82	0.410	.512398	5.146513
6	.8261061	.480529	-0.33	0.743	.2641857	2.583225
7	.7919346	.4583485	-0.40	0.687	.2547058	2.462293
8	.9265324	.5320575	-0.13	0.894	.3006503	2.855352
9	.7797282	.4487269	-0.43	0.665	.2523969	2.40881
10	.7730383	.4460245	-0.45	0.655	.2495044	2.395101
99	1.460799	.9961124	0.56	0.578	.3838485	5.559312
canscore7cat						
2	1.794562	.6174142	1.70	0.089	.914334	3.522184
3	2.669336	.8389288	3.12	0.002	1.441732	4.942219
4	2.444791	.7847459	2.79	0.005	1.303219	4.586341
5	2.855091	.9563174	3.13	0.002	1.480841	5.50467
6	3.447004	1.201155	3.55	0.000	1.741122	6.824244
99	1.896987	.9236675	1.31	0.189		

```
.7304718    4.926351
                     |
neareststa3ndistance |   1.006099   .0010286     5.95   0.000
1.004085    1.008117
------------------------------------------------------------------------
--------------


.
. * Non-Hospitalized
. stcox   timeinfected_0_90 timeinfected_91_180 timeinfected_181_365
///
>                  timeinfected_366_730 `imbalanced'  ///
>                  if index_hospitalization==0 ///
>                  , efron vce(cluster patienticn)
strata(matchgroupnumber)

        Failure _d: died_0_730
  Analysis time _t: tte_0_730
       ID variable: uniq_patid

Iteration 0:   log pseudolikelihood = -110382.69
Iteration 1:   log pseudolikelihood = -78084.666
Iteration 2:   log pseudolikelihood = -75957.277
Iteration 3:   log pseudolikelihood = -75826.973
Iteration 4:   log pseudolikelihood = -75825.526
Iteration 5:   log pseudolikelihood = -75825.526
Refining estimates:
Iteration 0:   log pseudolikelihood = -75825.526

Stratified Cox regression with Efron method for ties
Strata variable: matchgroupnumber

No. of subjects = 1,185,956                          Number of obs =
4,610,949
No. of failures =    65,061
Time at risk    = 575159521
                                                     Wald chi2(32) =
41033.75
Log pseudolikelihood = -75825.526                    Prob > chi2   =
0.0000

                             (Std. err. adjusted for 1,040,959 clusters
in patienticn)
------------------------------------------------------------------------
--------------
                     |               Robust
                  _t | Haz. ratio   std. err.      z    P>|z|     [95%
conf. interval]
---------------------+--------------------------------------------------
--------------
   timeinfected_0_90 |   2.685776   .0610014    43.50   0.000
2.568838    2.808038
 timeinfected_91_180 |   .7958783   .0281021    -6.47   0.000
.7426619     .852908
```

timeinfected_181_365	.6655412	.0183059	-14.80	0.000	.6306121	.7024049
timeinfected_366_730	.6678794	.0214694	-12.56	0.000	.6270984	.7113124
ageatindexdate	1.06346	.0008091	80.87	0.000	1.061876	1.065047
bmi	.983417	.0011663	-14.10	0.000	.9811337	.9857057
ethnicity3cat						
NotHispanic	1.245418	.0294182	9.29	0.000	1.189074	1.304432
Missing	1.318301	.0462191	7.88	0.000	1.230755	1.412073
smoking4cat						
Former	.7509477	.0153342	-14.03	0.000	.7214866	.7816118
Never	.6592874	.0140648	-19.53	0.000	.6322893	.6874384
Missing	1.416026	.0524786	9.39	0.000	1.316817	1.52271
gagne	1.146393	.0042877	36.53	0.000	1.13802	1.154828
numipadmits	1.020387	.0069369	2.97	0.003	1.006881	1.034074
util_numpcstops	1.002465	.0029099	0.85	0.396	.9967781	1.008185
util_numscstops	.9907894	.0038424	-2.39	0.017	.9832869	.9983492
nosos11cat						
2	.9459805	.0451239	-1.16	0.244	.8615477	1.038688
3	.9692975	.0467629	-0.65	0.518	.8818437	1.065424
4	.8547473	.0429125	-3.13	0.002	.774646	.9431313
5	.7876494	.0402236	-4.67	0.000	.7126296	.8705667
6	.7874746	.0410336	-4.59	0.000	.7110207	.8721493
7	.7431751	.0384667	-5.73	0.000	.6714799	.8225255
8	.726324	.03568	-6.51	0.000	.6596535	.7997328
9	.6948543	.0381959	-6.62	0.000	.6238835	.7738985
10	.8022314	.0464572	-3.81	0.000	.7161543	.8986543
99	.8713138	.0900282	-1.33	0.182	.7115812	1.066902

```
        canscore7cat |
                  2  |   1.771898   .0653161    15.52   0.000
1.648396    1.904653
                  3  |   2.506954   .0888844    25.92   0.000
2.338659     2.68736
                  4  |   3.557269   .1414773    31.91   0.000
3.290511    3.845653
                  5  |    4.72518   .2235596    32.82   0.000
4.306713    5.184307
                  6  |   5.524527   .2973654    31.75   0.000
4.971392    6.139206
                 99  |   1.808708     .25705     4.17   0.000
1.368981    2.389679
                     |
neareststa3ndistance |   1.003734   .0001643    22.78   0.000
1.003412    1.004056
--------------------------------------------------------------------------
--------------


.
. *---------------------------------
. * Subgroup Analysis - Age, Adj.
. *---------------------------------
.
. local ageimbal   bmi i.ethnicity3cat i.smoking4cat ///
>                                 gagne numipadmits util_numpcstops
util_numscstops ///
>                                 i.nosos11cat i.canscore7cat
neareststa3ndistance

.
. stcox   timeinfected_0_90 timeinfected_91_180 timeinfected_181_365
///
>                  timeinfected_366_730 `ageimbal'  ///
>                  if agecat==1 ///
>                  , efron vce(cluster patienticn)
strata(matchgroupnumber)

        Failure _d: died_0_730
  Analysis time _t: tte_0_730
       ID variable: uniq_patid

Iteration 0:   log pseudolikelihood = -14558.298
Iteration 1:   log pseudolikelihood = -10426.226
Iteration 2:   log pseudolikelihood = -10288.915
Iteration 3:   log pseudolikelihood = -10285.867
Iteration 4:   log pseudolikelihood = -10285.864
Refining estimates:
Iteration 0:   log pseudolikelihood = -10285.864

Stratified Cox regression with no ties
Strata variable: matchgroupnumber

No. of subjects =     670,258                        Number of obs =
```

```
2,630,678
No. of failures =      12,010
Time at risk    = 331392467.5
                                                     Wald chi2(31)  =
6018.82
Log pseudolikelihood = -10285.864                    Prob > chi2    =
0.0000

                               (Std. err. adjusted for 584,782 clusters
in patienticn)
------------------------------------------------------------------------
--------------
                     |               Robust
                  _t | Haz. ratio   std. err.      z    P>|z|     [95%
conf. interval]
---------------------+--------------------------------------------------
--------------
   timeinfected_0_90 |   5.284862   .2392858    36.77   0.000
4.836078    5.775292
 timeinfected_91_180 |   .8990531   .0605317    -1.58   0.114
.7879079    1.025877
timeinfected_181_365 |    .671949   .0338182    -7.90   0.000
.6088308    .7416108
timeinfected_366_730 |   .7167203   .0439245    -5.43   0.000
.6355993    .8081946
                 bmi |    1.01642   .0020623     8.03   0.000
1.012386     1.02047
                     |
       ethnicity3cat |
        NotHispanic  |   1.042822   .0492994     0.89   0.375
.9505384    1.144065
            Missing  |   .9905843   .0794553    -0.12   0.906
.8464788    1.159223
                     |
          smoking4cat |
             Former  |   1.264539   .0520099     5.71   0.000
1.166602    1.370698
              Never  |   1.106283   .0494139     2.26   0.024
1.013552    1.207498
            Missing  |   1.494596   .0891653     6.74   0.000
1.329666    1.679985
                     |
               gagne |   1.281692   .0096121    33.09   0.000
1.26299     1.30067
         numipadmits |   .9424521   .0089856    -6.22   0.000
.9250041    .9602291
     util_numpcstops |    1.00814   .0018797     4.35   0.000
1.004463    1.011831
     util_numscstops |   .9934476   .0014898    -4.38   0.000
.9905319    .9963719
                     |
          nosos11cat |
                   2 |   1.860846    .261989     4.41   0.000
1.412113    2.452174
```

```
                  3  |   2.038102   .2812301     5.16   0.000
1.555148    2.671038
                  4  |   2.339634   .3223176     6.17   0.000
1.786006    3.064877
                  5  |   2.496255    .347292     6.58   0.000
1.900489    3.278782
                  6  |   2.911601   .4024285     7.73   0.000
2.220662    3.817518
                  7  |    3.16451   .4390644     8.30   0.000
2.411045    4.153437
                  8  |   3.142462    .436919     8.24   0.000
2.392883     4.12685
                  9  |   3.320349   .4617294     8.63   0.000
2.528222    4.360661
                 10  |   3.534511   .4953993     9.01   0.000
2.685495    4.651942
                 99  |    4.58878   .7759396     9.01   0.000
3.294301    6.391917
                     |
        canscore7cat |
                  2  |   2.080118   .1240203    12.28   0.000
1.850708    2.337966
                  3  |   3.126317   .1945111    18.32   0.000
2.76741    3.531771
                  4  |   5.145746   .3333184    25.29   0.000
4.532223     5.84232
                  5  |   10.20501   .7459336    31.78   0.000
8.842903    11.77692
                  6  |   17.16867   1.452132    33.61   0.000
14.54594     20.2643
                 99  |   6.622869   1.049954    11.93   0.000
4.854016    9.036311
                     |
neareststa3ndistance |    1.00161   .0004359     3.70   0.000
1.000756    1.002465
-------------------------------------------------------------------------
--------------

.
. stcox   timeinfected_0_90 timeinfected_91_180 timeinfected_181_365
///
>                 timeinfected_366_730 `ageimbal'  ///
>                 if agecat==2 ///
>                 , efron vce(cluster patienticn)
strata(matchgroupnumber)

        Failure _d: died_0_730
  Analysis time _t: tte_0_730
       ID variable: uniq_patid

Iteration 0:   log pseudolikelihood = -53913.685
Iteration 1:   log pseudolikelihood = -44548.506
Iteration 2:   log pseudolikelihood = -44390.217
Iteration 3:   log pseudolikelihood = -44389.356
```

```
Iteration 4:   log pseudolikelihood = -44389.356
Refining estimates:
Iteration 0:   log pseudolikelihood = -44389.356

Stratified Cox regression with Efron method for ties
Strata variable: matchgroupnumber

No. of subjects =     513,602                        Number of obs =
1,962,091
No. of failures =      50,712
Time at risk    = 241928487.5
                                                     Wald chi2(31) =
16103.99
Log pseudolikelihood = -44389.356                    Prob > chi2   =
0.0000

                               (Std. err. adjusted for 453,183 clusters
in patienticn)
------------------------------------------------------------------------
--------------
                     |               Robust
                  _t | Haz. ratio   std. err.      z    P>|z|     [95%
conf. interval]
---------------------+--------------------------------------------------
--------------
   timeinfected_0_90 |   6.789824   .1469138    88.52   0.000
6.507899    7.083963
 timeinfected_91_180 |   1.100616   .0351329     3.00   0.003
1.033867    1.171675
timeinfected_181_365 |   .7972723   .0213794    -8.45   0.000
.7564515    .8402959
timeinfected_366_730 |    .784958   .0226386    -8.40   0.000
.741818    .8306068
                 bmi |   .9806657   .0012335   -15.52   0.000
.9782511    .9830863
                     |
       ethnicity3cat |
        NotHispanic  |   1.168981   .0334091     5.46   0.000
1.105301    1.236331
            Missing  |   1.225305   .0521831     4.77   0.000
1.127181    1.331972
                     |
         smoking4cat |
             Former  |   .9551981   .0220499    -1.99   0.047
.9129443    .9994076
              Never  |   .8393279   .0205431    -7.16   0.000
.8000146     .880573
            Missing  |   1.723717   .0961577     9.76   0.000
1.545189    1.922872
                     |
               gagne |   1.162702   .0046521    37.68   0.000
1.153619    1.171855
         numipadmits |   .9902413   .0072735    -1.34   0.182
.9760877      1.0046
```

```
     util_numpcstops |   1.003995   .0032129     1.25   0.213
.9977178    1.010312
     util_numscstops |   .9929268   .0042029    -1.68   0.094
.9847234    1.001199
                     |
          nosos11cat |
                  2  |   .9713428   .0787541    -0.36   0.720
.8286271    1.138639
                  3  |   1.060664   .0859827     0.73   0.468
.9048472    1.243313
                  4  |   .9181926   .0751208    -1.04   0.297
.7821568    1.077888
                  5  |   .8083899   .0668536    -2.57   0.010
.6874273    .9506376
                  6  |   .7646974   .0634564    -3.23   0.001
.6499124    .8997552
                  7  |   .7171678   .0593465    -4.02   0.000
.6097936    .8434488
                  8  |   .6960344   .0562486    -4.48   0.000
.5940767    .8154904
                  9  |   .6528993   .0552624    -5.04   0.000
.5530942     .770714
                 10  |    .715163   .0624061    -3.84   0.000
.6027373    .8485591
                 99  |   .9311091   .1480032    -0.45   0.653
.6818659    1.271458
                     |
        canscore7cat |
                  2  |   2.000095   .0888593    15.60   0.000
1.833301    2.182064
                  3  |   3.064247   .1343136    25.55   0.000
2.811988    3.339136
                  4  |   4.265834   .2116398    29.24   0.000
3.870557    4.701478
                  5  |   5.710978   .3308729    30.07   0.000
5.097943     6.39773
                  6  |    7.54406   .5008294    30.44   0.000
6.623632    8.592392
                 99  |   3.041893   .5061014     6.69   0.000
2.195449    4.214678
                     |
neareststa3ndistance |    1.00334   .0001943    17.21   0.000
1.002959    1.003721
----------------------------------------------------------------------------
--------------

.
. stcox   timeinfected_0_90 timeinfected_91_180 timeinfected_181_365
///
>                 timeinfected_366_730 `ageimbal'  ///
>                 if agecat==3 ///
>                 , efron vce(cluster patienticn)
strata(matchgroupnumber)
```

```
        Failure _d: died_0_730
  Analysis time _t: tte_0_730
       ID variable: uniq_patid

Iteration 0:   log pseudolikelihood = -3847.2796
Iteration 1:   log pseudolikelihood =  -3282.884
Iteration 2:   log pseudolikelihood = -3261.7561
Iteration 3:   log pseudolikelihood =  -3261.483
Iteration 4:   log pseudolikelihood = -3261.4829
Refining estimates:
Iteration 0:   log pseudolikelihood = -3261.4829

Stratified Cox regression with Efron method for ties
Strata variable: matchgroupnumber

No. of subjects =     61,624                           Number of obs =
214,806
No. of failures =     19,675
Time at risk    = 25,302,394
                                                       Wald chi2(31) =
1063.16
Log pseudolikelihood = -3261.4829                      Prob > chi2   =
0.0000

                                (Std. err. adjusted for 55,357 clusters
in patienticn)
------------------------------------------------------------------------
--------------
                     |               Robust
                  _t | Haz. ratio   std. err.      z    P>|z|     [95%
conf. interval]
---------------------+--------------------------------------------------
--------------
   timeinfected_0_90 |   6.671193   .5149214    24.59   0.000
5.734596    7.760759
 timeinfected_91_180 |   1.012016   .1035795     0.12   0.907
.8280701    1.236823
timeinfected_181_365 |    .995476   .0778448    -0.06   0.954
.85402    1.160362
timeinfected_366_730 |    .961431   .0914894    -0.41   0.679
.7978443    1.158559
                 bmi |   .9571774   .0037111   -11.29   0.000
.9499313    .9644787
                     |
       ethnicity3cat |
        NotHispanic  |   1.395965   .1376561     3.38   0.001
1.150635    1.693602
            Missing  |   1.489865   .1918538     3.10   0.002
1.157539    1.917602
                     |
         smoking4cat |
             Former  |   .9711018   .1047362    -0.27   0.786
.7860681    1.199691
              Never  |   .9014219   .0993692    -0.94   0.346
```

```
.7262648    1.118822
            Missing  |   1.675092   .2065422     4.18   0.000
1.315479    2.133013
                     |
               gagne |    1.08911   .0097226     9.56   0.000
1.07022    1.108334
         numipadmits |   .9908893   .0140306    -0.65   0.518
.9637679    1.018774
     util_numpcstops |    1.01271   .0024446     5.23   0.000
1.00793    1.017513
     util_numscstops |   .9784877   .0021997    -9.67   0.000
.9741858    .9828086
                     |
          nosos11cat |
                  2  |   .9429193   .0977917    -0.57   0.571
.769476    1.155458
                  3  |   .9031725   .0983665    -0.94   0.350
.7295658     1.11809
                  4  |   .8953397   .1002418    -0.99   0.323
.7189318    1.115034
                  5  |   .8658082   .1022117    -1.22   0.222
.6869646    1.091212
                  6  |   1.096416   .1298875     0.78   0.437
.8692355    1.382972
                  7  |   .9816744   .1175476    -0.15   0.877
.7763239    1.241344
                  8  |   .9285591   .1156599    -0.60   0.552
.72742    1.185315
                  9  |   .9388058    .115931    -0.51   0.609
.7369921    1.195883
                 10  |   1.197808   .1488669     1.45   0.146
.9388527    1.528188
                 99  |   1.116855   .2182779     0.57   0.572
.7614444    1.638157
                     |
        canscore7cat |
                  2  |   .1010701   .0870501    -2.66   0.008
.0186851    .5466999
                  3  |   .2257802    .188703    -1.78   0.075
.0438801    1.161728
                  4  |   .3572221   .2985071    -1.23   0.218
.0694457    1.837518
                  5  |   .4832342   .4045207    -0.87   0.385
.0936715    2.492918
                  6  |   .5566018   .4673908    -0.70   0.485
.1073426    2.886139
                 99  |   .5242315   .4522857    -0.75   0.454
.0966365    2.843841
                     |
neareststa3ndistance |   1.002624   .0006049     4.34   0.000
1.001439     1.00381
----------------------------------------------------------------------------
--------------
```

```
.
.
. *---------------------------------------------------
. * Subgroup Analyses - Comorbidities, Adj.
. *---------------------------------------------------
.
. * Subgroup analysis by tertile of comorbidity & adjust for
. * covariates
.
. * create tertiles of Gagne scores
. sum gagne, de

                gagne score (min=-2, max=24)
-------------------------------------------------------------
      Percentiles      Smallest
 1%           -1             -2
 5%           -1             -2
10%           -1             -2       Obs           4,807,575
25%            0             -2       Sum of wgt.   4,807,575

50%            1                      Mean           1.352648
                        Largest       Std. dev.      2.190437
75%            2             20
90%            4             20       Variance       4.798016
95%            6             20       Skewness       1.904482
99%            9             22       Kurtosis       7.836349

. histogram gagne
(bin=66, start=-2, width=.36363636)

. xtile gagne_tertile = gagne, nq(3)

. bysort gagne_tertile: sum gagne, de

------------------------------------------------------------------------
------------------------------------------------------------------------
-----------------------------------------------
-> gagne_tertile = 1

                gagne score (min=-2, max=24)
-------------------------------------------------------------
      Percentiles      Smallest
 1%           -1             -2
 5%           -1             -2
10%           -1             -2       Obs           2,188,313
25%            0             -2       Sum of wgt.   2,188,313

50%            0                      Mean          -.2387684
                        Largest       Std. dev.      .4293505
75%            0              0
90%            0              0       Variance       .1843419
95%            0              0       Skewness      -1.274364
99%            0              0       Kurtosis       2.772565
```

```
--------------------------------------------------------------------------
--------------------------------------------------------------------------
-----------------------------------------------
-> gagne_tertile = 2

                gagne score (min=-2, max=24)
-------------------------------------------------------------
      Percentiles      Smallest
 1%            1              1
 5%            1              1
10%            1              1       Obs           1,039,996
25%            1              1       Sum of wgt.   1,039,996

50%            1                      Mean                  1
                        Largest       Std. dev.             0
75%            1              1
90%            1              1       Variance              0
95%            1              1       Skewness              .
99%            1              1       Kurtosis              .

--------------------------------------------------------------------------
--------------------------------------------------------------------------
-----------------------------------------------
-> gagne_tertile = 3

                gagne score (min=-2, max=24)
-------------------------------------------------------------
      Percentiles      Smallest
 1%            2              2
 5%            2              2
10%            2              2       Obs           1,579,266
25%            2              2       Sum of wgt.   1,579,266

50%            3                      Mean           3.790027
                        Largest       Std. dev.      2.195128
75%            5             20
90%            7             20       Variance       4.818587
95%            8             20       Skewness       1.719706
99%           11             22       Kurtosis       6.502357


.
. * Tertiles of Gagne scores
. local imbalanced ageatindexdate bmi i.ethnicity3cat i.smoking4cat ///
>                                   numipadmits util_numpcstops 
util_numscstops ///
>                                   i.nosos11cat i.canscore7cat 
neareststa3ndistance

.
. forval i=1/3 {
  2.
.         stcox   timeinfected_0_90 timeinfected_91_180 
timeinfected_181_365  ///
```

```
>                         timeinfected_366_730 `imbalanced'  ///
>                         if gagne_tertile==`i'  ///
>                         , efron vce(cluster patienticn)
strata(matchgroupnumber)
  3. }

        Failure _d: died_0_730
  Analysis time _t: tte_0_730
       ID variable: uniq_patid

Iteration 0:   log pseudolikelihood = -15700.491
Iteration 1:   log pseudolikelihood = -9803.7776
Iteration 2:   log pseudolikelihood = -9309.1306
Iteration 3:   log pseudolikelihood = -9270.3879
Iteration 4:   log pseudolikelihood = -9269.9866
Iteration 5:   log pseudolikelihood = -9269.9865
Refining estimates:
Iteration 0:   log pseudolikelihood = -9269.9865

Stratified Cox regression with no ties
Strata variable: matchgroupnumber

No. of subjects =   558,603                          Number of obs =
2,188,313
No. of failures =    12,952
Time at risk    = 273188034
                                                     Wald chi2(31) =
6841.31
Log pseudolikelihood = -9269.9865                    Prob > chi2   =
0.0000

                               (Std. err. adjusted for 502,965 clusters
in patienticn)
-----------------------------------------------------------------------
--------------
                     |               Robust
                  _t | Haz. ratio   std. err.      z    P>|z|     [95%
conf. interval]
---------------------+-------------------------------------------------
--------------
   timeinfected_0_90 |   7.500308   .3367596    44.88   0.000
6.86848    8.190257
 timeinfected_91_180 |   1.092474   .0832297     1.16   0.246
.940942     1.26841
timeinfected_181_365 |   .6642259   .0379718    -7.16   0.000
.5938205    .7429788
timeinfected_366_730 |   .6921604   .0440451    -5.78   0.000
.6110001    .7841015
      ageatindexdate |    1.07893   .0018691    43.85   0.000
1.075273      1.0826
                 bmi |   .9983562   .0026552    -0.62   0.536
.9931656    1.003574
                     |
       ethnicity3cat |
```

NotHispanic	1.280785	.080613	3.93	0.000	1.132143	1.448942
Missing	1.234298	.1081758	2.40	0.016	1.039488	1.465618
smoking4cat						
Former	.6624782	.0367862	-7.42	0.000	.5941635	.7386474
Never	.5459423	.0330027	-10.01	0.000	.4849431	.6146144
Missing	1.706001	.0959742	9.49	0.000	1.527894	1.904869
numipadmits	.9505709	.0367023	-1.31	0.189	.8812901	1.025298
util_numpcstops	.9832362	.0033014	-5.04	0.000	.9767869	.989728
util_numscstops	.9968608	.0029489	-1.06	0.288	.9910977	1.002657
nosos11cat						
2	.8761236	.0616149	-1.88	0.060	.7633139	1.005605
3	.8618274	.0636981	-2.01	0.044	.7456029	.996169
4	.7932618	.0627446	-2.93	0.003	.679343	.9262837
5	.7636355	.0625533	-3.29	0.001	.650369	.8966281
6	.8066059	.0683144	-2.54	0.011	.6832348	.9522539
7	.7688624	.0683523	-2.96	0.003	.6459165	.9152103
8	.7526862	.0685552	-3.12	0.002	.6296308	.8997916
9	.7029997	.06663	-3.72	0.000	.5838196	.8465092
10	.8173364	.0838966	-1.97	0.049	.6683871	.9994789
99	.698701	.0981341	-2.55	0.011	.5305645	.9201199
canscore7cat						
2	1.566203	.0695732	10.10	0.000	1.43561	1.708676
3	2.140287	.1034365	15.75	0.000	1.946861	2.352931
4	3.256214	.1831993	20.98	0.000	2.916239	3.635824
5	3.971556	.3151847	17.38	0.000	3.399452	4.639941
6	4.446388	.6905831	9.61	0.000	3.279476	6.028514
99	.4094631	.0807369	-4.53	0.000		

```
.2782128    .6026324
                     |
nearststa3ndistance |   1.003543   .0004132     8.59   0.000
1.002733    1.004353
--------------------------------------------------------------------------
--------------

        Failure _d: died_0_730
  Analysis time _t: tte_0_730
       ID variable: uniq_patid

Iteration 0:   log pseudolikelihood = -6722.4221
Iteration 1:   log pseudolikelihood =  -4490.065
Iteration 2:   log pseudolikelihood = -4255.7469
Iteration 3:   log pseudolikelihood = -4235.9202
Iteration 4:   log pseudolikelihood = -4235.6764
Iteration 5:   log pseudolikelihood = -4235.6764
Refining estimates:
Iteration 0:   log pseudolikelihood = -4235.6764

Stratified Cox regression with Efron method for ties
Strata variable: matchgroupnumber

No. of subjects =   266,962                          Number of obs =
1,039,996
No. of failures =    11,065
Time at risk    = 129794950
                                                     Wald chi2(31) =
2722.68
Log pseudolikelihood = -4235.6764                    Prob > chi2   =
0.0000

                               (Std. err. adjusted for 238,877 clusters
in patienticn)
--------------------------------------------------------------------------
--------------
                     |               Robust
                  _t | Haz. ratio   std. err.      z    P>|z|     [95%
conf. interval]
---------------------+----------------------------------------------------
--------------
   timeinfected_0_90 |   6.690617   .4389967    28.97   0.000
5.883227     7.60881
 timeinfected_91_180 |   .9872681   .0927747    -0.14   0.892
.8211959    1.186925
timeinfected_181_365 |   .6966577   .0617185    -4.08   0.000
.5856115    .8287609
timeinfected_366_730 |    .648577   .0603411    -4.65   0.000
.5404669    .7783126
      ageatindexdate |   1.071258   .0024072    30.63   0.000
1.06655    1.075986
                 bmi |   .9884357   .0031168    -3.69   0.000
.9823458    .9945634
                     |
```

ethnicity3cat						
NotHispanic	1.475896	.126844	4.53	0.000	1.247097	1.746671
Missing	1.666383	.2088367	4.07	0.000	1.303465	2.130347
smoking4cat						
Former	.6098506	.0417252	-7.23	0.000	.5333169	.6973673
Never	.5384721	.0426363	-7.82	0.000	.461068	.6288708
Missing	1.253396	.1405786	2.01	0.044	1.006049	1.561556
numipadmits	.9522055	.0394156	-1.18	0.237	.878003	1.032679
util_numpcstops	1.004287	.0078943	0.54	0.586	.9889334	1.01988
util_numscstops	.9797727	.0113424	-1.77	0.078	.9577923	1.002258
nosos11cat						
2	1.127268	.173236	0.78	0.436	.8340965	1.523484
3	.9379436	.1455454	-0.41	0.680	.6919769	1.271341
4	.6826789	.1067492	-2.44	0.015	.5024765	.9275072
5	.7095511	.1112083	-2.19	0.029	.5218845	.9647016
6	.713858	.1126987	-2.14	0.033	.5238781	.9727323
7	.674498	.1073065	-2.48	0.013	.4938126	.9212958
8	.6487984	.103657	-2.71	0.007	.474368	.8873689
9	.6125635	.0991114	-3.03	0.002	.4460962	.8411503
10	.6310939	.1047568	-2.77	0.006	.455828	.8737496
99	.6612896	.2011372	-1.36	0.174	.3643268	1.200307
canscore7cat						
2	1.61206	.1678453	4.59	0.000	1.314485	1.977002
3	2.125999	.2187151	7.33	0.000	1.737779	2.600948
4	2.90234	.3070768	10.07	0.000	2.358786	3.571151
5	5.057096	.6054842	13.54	0.000	3.999329	6.394627
6	6.567526	1.026871	12.04	0.000	4.834054	8.922615

```
                 99  |   .7513228   .3286854    -0.65   0.513
.3187474    1.770951
                     |
neareststa3ndistance |   1.003733   .0006102     6.13   0.000
1.002537    1.004929
------------------------------------------------------------------------
--------------

        Failure _d: died_0_730
  Analysis time _t: tte_0_730
       ID variable: uniq_patid

Iteration 0:   log pseudolikelihood = -61611.495
Iteration 1:   log pseudolikelihood = -48752.581
Iteration 2:   log pseudolikelihood =  -48126.01
Iteration 3:   log pseudolikelihood = -48110.657
Iteration 4:   log pseudolikelihood =  -48110.59
Iteration 5:   log pseudolikelihood =  -48110.59
Refining estimates:
Iteration 0:   log pseudolikelihood =  -48110.59

Stratified Cox regression with Efron method for ties
Strata variable: matchgroupnumber

No. of subjects =     419,919                        Number of obs =
1,579,266
No. of failures =      58,380
Time at risk    = 195640364.5
                                                     Wald chi2(31) =
20250.19
Log pseudolikelihood = -48110.59                     Prob > chi2   =
0.0000

                               (Std. err. adjusted for 358,321 clusters
in patienticn)
------------------------------------------------------------------------
--------------
                     |               Robust
                  _t | Haz. ratio   std. err.      z    P>|z|     [95%
conf. interval]
---------------------+--------------------------------------------------
--------------
   timeinfected_0_90 |   5.216509   .1041893    82.70   0.000
5.016247    5.424766
 timeinfected_91_180 |    1.11421   .0342786     3.52   0.000
1.049011    1.183462
timeinfected_181_365 |    .896399   .0208447    -4.70   0.000
.8564612    .9381991
timeinfected_366_730 |   .8391086   .0232476    -6.33   0.000
.7947592    .8859329
      ageatindexdate |   1.056754   .0007212    80.88   0.000
1.055342    1.058169
                 bmi |   .9723692   .0009973   -27.32   0.000
.9704164    .9743259
```

```
                     |
     ethnicity3cat |
       NotHispanic  |   1.298186   .0348883     9.71   0.000
1.231576    1.368398
           Missing  |   1.330528   .0561322     6.77   0.000
1.224936    1.445221
                     |
        smoking4cat |
            Former  |   .7561238   .0158406   -13.34   0.000
.7257055    .7878171
             Never  |   .6661088   .0154207   -17.55   0.000
.6365601     .697029
           Missing  |   1.249002   .0438704     6.33   0.000
1.16591    1.338015
                     |
        numipadmits |   1.089049   .0039806    23.34   0.000
1.081275    1.096878
    util_numpcstops |   1.003157   .0005878     5.38   0.000
1.002006     1.00431
    util_numscstops |   .9921074    .000543   -14.48   0.000
.9910437    .9931722
                     |
         nosos11cat |
                 2  |   1.228826   .1708616     1.48   0.138
.9356971    1.613784
                 3  |   1.188955    .147572     1.39   0.163
.9322123    1.516407
                 4  |   1.032778   .1247076     0.27   0.789
.8151257    1.308547
                 5  |    .746758   .0885363    -2.46   0.014
.5919168    .9421046
                 6  |   .7019939   .0821868    -3.02   0.003
.5580561    .8830571
                 7  |   .6491978   .0755549    -3.71   0.000
.5167881     .815533
                 8  |   .6314767   .0732206    -3.96   0.000
.5031058    .7926022
                 9  |   .6028599    .069684    -4.38   0.000
.4806477    .7561465
                10  |   .7274012   .0839814    -2.76   0.006
.5800953    .9121129
                99  |   .8319036   .1152345    -1.33   0.184
.6341112    1.091392
                     |
       canscore7cat |
                 2  |   1.704064   .2050474     4.43   0.000
1.346053    2.157296
                 3  |   2.154765   .2443652     6.77   0.000
1.725313    2.691114
                 4  |   2.903397   .3263227     9.48   0.000
2.329362    3.618894
                 5  |   3.927339   .4440077    12.10   0.000
3.146772    4.901528
                 6  |   5.082562   .5824803    14.19   0.000
```

```
4.060054    6.362583
                   99  |   2.764754   .3918539     7.18   0.000
2.09418    3.650051
                       |
neareststa3ndistance |   1.004875   .0002013    24.28   0.000
1.004481     1.00527
------------------------------------------------------------------------
--------------

.
.
.
. ******************************************************************
. * PP1: Per Protocol for IPCWs - Censoring at Crossover Infections *
. ******************************************************************
.
. *----------------------------------------
. * Prepping Data for IPCWs and Table 1
. *----------------------------------------
.
. use Data\mortality_varforcox_20230516, clear
(Created in file
'P:\ORD_Ioannou_202104007D\MethodsGroup\Programs\CORC_DataExplor)

.
. * create ttinfection variable
. gen ttinfection = futureinfectedcontrolindexdate - index_dt_case
(1,108,158 missing values generated)

. sum ttinfection

    Variable |        Obs        Mean    Std. dev.       Min        Max
-------------+---------------------------------------------------------
 ttinfection |    137,326    338.8583    179.3397          0        867

. count if ttinfection==0
  6


.
. *-----------------------------------------------------------------------
----------
. * identify those who die during each period; those who die after
infection
. * crossover are coded as not dead at the time of crossover
. *-----------------------------------------------------------------------
----------
.
. * Died Index Day-Day 90;
. gen died_0_90_crossover = .
(1,245,484 missing values generated)

. replace died_0_90_crossover = 1 if inrange(indextodeath_caseindex, 0,
90)
(23,814 real changes made)
```

```
. replace died_0_90_crossover = 0 if indextodeath_caseindex>90
(1,221,670 real changes made)

. replace died_0_90_crossover = 0 if indextodeath_caseindex>ttinfection
(1,157 real changes made)

.
. tab died_0_90 died_0_90_crossover, m

           |  died_0_90_crossover
 died_0_90 |         0          1 |     Total
-----------+----------------------+----------
         0 | 1,221,670          0 | 1,221,670
         1 |     1,157     22,657 |    23,814
-----------+----------------------+----------
     Total | 1,222,827     22,657 | 1,245,484

. tab died_0_90_crossover case, m

           |     indicator: is
           |  `index_dt` the date
           | of the first positive
           | covid-19 test result
died_0_90_ |          for
 crossover |         0          1 |     Total
-----------+----------------------+----------
         0 | 1,027,181    195,646 | 1,222,827
         1 |    10,242     12,415 |    22,657
-----------+----------------------+----------
     Total | 1,037,423    208,061 | 1,245,484

.
. * Died Day 91-180;
. gen died_91_180_crossover = .
(1,245,484 missing values generated)

. replace died_91_180_crossover = 1 if inrange(indextodeath_caseindex,
91, 180)
(12,359 real changes made)

. replace died_91_180_crossover = 0 if indextodeath_caseindex>180
(1,209,311 real changes made)

. replace died_91_180_crossover = 0 if
indextodeath_caseindex>ttinfection
(2,424 real changes made)

. replace died_91_180_crossover = 0 if
(indextodeath_caseindex>ttstudyend) & (inrange(indextodeath_caseindex,
91, 180)) ///
>
& (inrange(ttstudyend, 91, 180))
(0 real changes made)
```

```
. tab died_91_180 died_91_180_crossover, m //missing represents those
who were censored in period 1

died_91_18 |      died_91_180_crossover
         0 |         0          1          . |     Total
-----------+---------------------------------+----------
         0 | 1,209,311          0          0 | 1,209,311
         1 |     1,267     11,092          0 |    12,359
         . |     1,157          0     22,657 |    23,814
-----------+---------------------------------+----------
     Total | 1,211,735     11,092     22,657 | 1,245,484

. tab died_91_180_crossover case, m

           |     indicator: is
           |  `index_dt` the date
           | of the first positive
died_91_18 | covid-19 test result
0_crossove |          for
         r |         0          1 |     Total
-----------+----------------------+----------
         0 | 1,018,133    193,602 | 1,211,735
         1 |     9,048      2,044 |    11,092
         . |    10,242     12,415 |    22,657
-----------+----------------------+----------
     Total | 1,037,423    208,061 | 1,245,484

.
. * Died Day 181-365;
. gen died_181_365_crossover = .
(1,245,484 missing values generated)

. replace died_181_365_crossover = 1 if inrange(indextodeath_caseindex,
181, 365)
(26,130 real changes made)

. replace died_181_365_crossover = 0 if indextodeath_caseindex>365
(1,183,181 real changes made)

. replace died_181_365_crossover = 0 if
indextodeath_caseindex>ttinfection
(6,211 real changes made)

. replace died_181_365_crossover = 0 if
(indextodeath_caseindex>ttstudyend) & (inrange(indextodeath_caseindex,
181, 365)) ///
>
& (inrange(ttstudyend, 181, 365))
(59 real changes made)

.
. tab died_181_365 died_181_365_crossover, m //missing represents those
who were censored in period 1&2
```

```
died_181_3 |      died_181_365_crossover
        65 |         0          1          . |     Total
-----------+---------------------------------+----------
         0 | 1,183,244          0          0 | 1,183,244
         1 |     3,783     22,284          0 |    26,067
         . |     2,424          0     33,749 |    36,173
-----------+---------------------------------+----------
     Total | 1,189,451     22,284     33,749 | 1,245,484

. tab died_181_365_crossover case, m

           |     indicator: is
           |  `index_dt` the date
           | of the first positive
died_181_3 | covid-19 test result
65_crossov |          for
        er |         0          1 |     Total
-----------+----------------------+----------
         0 |   999,281    190,170 | 1,189,451
         1 |    18,852      3,432 |    22,284
         . |    19,290     14,459 |    33,749
-----------+----------------------+----------
     Total | 1,037,423    208,061 | 1,245,484

.
. * Died Day 366-730;
. gen died_366_730_crossover = .
(1,245,484 missing values generated)

. replace died_366_730_crossover = 1 if inrange(indextodeath_caseindex,
366, 730)
(32,904 real changes made)

. replace died_366_730_crossover = 0 if indextodeath_caseindex>730
(1,150,277 real changes made)

. replace died_366_730_crossover = 0 if
indextodeath_caseindex>ttinfection
(12,311 real changes made)

. replace died_366_730_crossover = 0 if
(indextodeath_caseindex>ttstudyend) & (inrange(indextodeath_caseindex,
366, 730)) ///
>
& (inrange(ttstudyend, 366, 730))
(10,385 real changes made)

.
. tab died_366_730 died_366_730_crossover, m //missing represents those
who were censored in period 1-3

died_366_7 |      died_366_730_crossover
        30 |         0          1          . |     Total
```

```
-----------+-----------------------------------+----------
         0 | 1,162,593          0          0 | 1,162,593 
         1 |     4,169     16,419          0 |    20,588 
         . |     6,211          0     56,092 |    62,303 
-----------+-----------------------------------+----------
     Total | 1,172,973     16,419     56,092 | 1,245,484 

. tab died_366_730_crossover case, m

           |     indicator: is
           |  `index_dt` the date
           | of the first positive
died_366_7 | covid-19 test result
30_crossov |          for
        er |         0          1 |     Total
-----------+----------------------+----------
         0 |   985,394    187,579 | 1,172,973 
         1 |    13,837      2,582 |    16,419 
         . |    38,192     17,900 |    56,092 
-----------+----------------------+----------
     Total | 1,037,423    208,061 | 1,245,484 

. 
. tab died_366_730 died_366_730_crossover if case==1

           | died_366_730_crossove
died_366_7 |           r
        30 |         0          1 |     Total
-----------+----------------------+----------
         0 |   187,579          0 |   187,579 
         1 |         0      2,582 |     2,582 
-----------+----------------------+----------
     Total |   187,579      2,582 |   190,161 

. tab died_366_730 died_366_730_crossover if case==0

           | died_366_730_crossove
died_366_7 |           r
        30 |         0          1 |     Total
-----------+----------------------+----------
         0 |   975,014          0 |   975,014 
         1 |     4,169     13,837 |    18,006 
-----------+----------------------+----------
     Total |   979,183     13,837 |   993,020 

. 
. *-----------------------------------------------------------------------
----------
. * for each period, identify whether patient is alive on the first day
of the
. * period AND was not an infection crossover in the prior period(s)
. *-----------------------------------------------------------------------
----------
. 
```

```
. * Alive on day 91 & no crossover in prior period
. gen alive_day91_crossover = .
(1,245,484 missing values generated)

. replace alive_day91_crossover = 1 if indextodeath_caseindex>=91
(1,221,670 real changes made)

. replace alive_day91_crossover = 0 if inrange(indextodeath_caseindex,
0, 90)
(23,814 real changes made)

. replace alive_day91_crossover = 0 if inrange(ttinfection, 0, 90)
(15,756 real changes made)

. replace alive_day91_crossover = 0 if ttstudyend<91
(0 real changes made)

.
. sum ttinfection if inrange(ttinfection, 0, 90)

    Variable |        Obs        Mean    Std. dev.       Min        Max
-------------+---------------------------------------------------------
 ttinfection |     17,044    46.16592    22.62754          0         90

. tab alive_day91 alive_day91_crossover, m

alive_day9 | alive_day91_crossover
         1 |         0          1 |     Total
-----------+----------------------+----------
         0 |    23,814          0 |    23,814
         1 |    15,756  1,205,914 | 1,221,670
-----------+----------------------+----------
     Total |    39,570  1,205,914 | 1,245,484

. tab alive_day91_crossover died_91_180_crossover

alive_day9 |
1_crossove | died_91_180_crossover
         r |         0          1 |     Total
-----------+----------------------+----------
         0 |    16,913          0 |    16,913
         1 | 1,194,822     11,092 | 1,205,914
-----------+----------------------+----------
     Total | 1,211,735     11,092 | 1,222,827

. tab alive_day91 alive_day91_crossover if case==1

alive_day9 | alive_day91_crossover
         1 |         0          1 |     Total
-----------+----------------------+----------
         0 |    12,415          0 |    12,415
         1 |         0    195,646 |   195,646
-----------+----------------------+----------
     Total |    12,415    195,646 |   208,061
```

```
. tab alive_day91 alive_day91_crossover if case==0

alive_day9 | alive_day91_crossover
         1 |         0          1 |     Total
-----------+----------------------+----------
         0 |    11,399          0 |    11,399 
         1 |    15,756  1,010,268 | 1,026,024 
-----------+----------------------+----------
     Total |    27,155  1,010,268 | 1,037,423 

. tab alive_day91_crossover died_0_90_crossover, m

alive_day9 |
1_crossove |  died_0_90_crossover
         r |         0          1 |     Total
-----------+----------------------+----------
         0 |    16,913     22,657 |    39,570 
         1 | 1,205,914          0 | 1,205,914 
-----------+----------------------+----------
     Total | 1,222,827     22,657 | 1,245,484 

. sum ttinfection if ttinfection<91 //17044 crossover infections prior
to day 91

    Variable |        Obs        Mean    Std. dev.       Min        Max
-------------+---------------------------------------------------------
 ttinfection |     17,044    46.16592    22.62754          0         90

.
. * Alive on day 181 & no crossover in prior periods
. gen alive_day181_crossover = .
(1,245,484 missing values generated)

. replace alive_day181_crossover = 1 if indextodeath_caseindex>=181
(1,209,311 real changes made)

. replace alive_day181_crossover = 0 if inrange(indextodeath_caseindex,
0, 180)
(36,173 real changes made)

. replace alive_day181_crossover = 0 if inrange(ttinfection, 0, 180)
(27,222 real changes made)

. replace alive_day181_crossover = 0 if ttstudyend<181
(0 real changes made)

.
. sum ttinfection if inrange(ttinfection, 0, 180)

    Variable |        Obs        Mean    Std. dev.       Min        Max
-------------+---------------------------------------------------------
 ttinfection |     29,870    84.65018    50.53237          0        180
```

```
. tab alive_day181 alive_day181_crossover, m

           | alive_day181_crossove
alive_day1 |           r
        81 |         0          1 |     Total
-----------+----------------------+----------
         0 |    36,173          0 |    36,173
         1 |    27,222  1,182,089 | 1,209,311
-----------+----------------------+----------
     Total |    63,395  1,182,089 | 1,245,484

. tab alive_day181 alive_day181_crossover if case==1

           | alive_day181_crossove
alive_day1 |           r
        81 |         0          1 |     Total
-----------+----------------------+----------
         0 |    14,459          0 |    14,459
         1 |         0    193,602 |   193,602
-----------+----------------------+----------
     Total |    14,459    193,602 |   208,061

. tab alive_day181 alive_day181_crossover if case==0

           | alive_day181_crossove
alive_day1 |           r
        81 |         0          1 |     Total
-----------+----------------------+----------
         0 |    21,714          0 |    21,714
         1 |    27,222    988,487 | 1,015,709
-----------+----------------------+----------
     Total |    48,936    988,487 | 1,037,423

. tab alive_day181_crossover died_91_180_crossover

alive_day1 |
81_crossov | died_91_180_crossover
        er |         0          1 |     Total
-----------+----------------------+----------
         0 |    29,646     11,092 |    40,738
         1 | 1,182,089          0 | 1,182,089
-----------+----------------------+----------
     Total | 1,211,735     11,092 | 1,222,827

. sum ttinfection if ttinfection<181 //29,870 crossover infections prior
to day 181

    Variable |        Obs        Mean    Std. dev.       Min        Max
-------------+---------------------------------------------------------
 ttinfection |     29,870    84.65018    50.53237          0        180

. tab died_181_365_crossover case if alive_day181_crossover

           |     indicator: is
```

```
           |  `index_dt` the date
           | of the first positive
died_181_3 | covid-19 test result
65_crossov |          for
        er |         0          1 |     Total
-----------+----------------------+----------
         0 |   969,635    190,170 | 1,159,805
         1 |    18,852      3,432 |    22,284
-----------+----------------------+----------
     Total |   988,487    193,602 | 1,182,089

.
. * Alive on day 366 & no crossover in prior periods
. gen alive_day366_crossover = .
(1,245,484 missing values generated)

. replace alive_day366_crossover = 1 if indextodeath_caseindex>=366
(1,183,181 real changes made)

. replace alive_day366_crossover = 0 if inrange(indextodeath_caseindex,
0, 365)
(62,303 real changes made)

. replace alive_day366_crossover = 0 if inrange(ttinfection, 0, 365)
(65,576 real changes made)

. replace alive_day366_crossover = 0 if ttstudyend<366
(48,497 real changes made)

.
. sum ttinfection if inrange(ttinfection, 0, 365)

    Variable |        Obs        Mean    Std. dev.       Min        Max
-------------+---------------------------------------------------------
 ttinfection |     72,253    201.5884    111.1569          0        365

. tab alive_day366 alive_day366_crossover

           | alive_day366_crossove
alive_day3 |           r
        66 |         0          1 |     Total
-----------+----------------------+----------
         0 |   114,374          0 |   114,374
         1 |    62,002  1,069,108 | 1,131,110
-----------+----------------------+----------
     Total |   176,376  1,069,108 | 1,245,484

. tab alive_day366 alive_day366_crossover if case==1

           | alive_day366_crossove
alive_day3 |           r
        66 |         0          1 |     Total
-----------+----------------------+----------
         0 |    26,404          0 |    26,404
```

```
         1 |         0    181,657 |   181,657 
-----------+----------------------+----------
     Total |    26,404    181,657 |   208,061 

. tab alive_day366 alive_day366_crossover if case==0

           | alive_day366_crossove
alive_day3 |           r
        66 |         0          1 |     Total
-----------+----------------------+----------
         0 |    87,970          0 |    87,970 
         1 |    62,002    887,451 |   949,453 
-----------+----------------------+----------
     Total |   149,972    887,451 | 1,037,423 

. tab alive_day366_crossover died_181_365_crossover

alive_day3 | died_181_365_crossove
66_crossov |           r
        er |         0          1 |     Total
-----------+----------------------+----------
         0 |   120,343     22,284 |   142,627 
         1 | 1,069,108          0 | 1,069,108 
-----------+----------------------+----------
     Total | 1,189,451     22,284 | 1,211,735 

. sum ttinfection if ttinfection<366  //72253 crossover infections prior
to day 181

    Variable |        Obs        Mean    Std. dev.       Min        Max
-------------+---------------------------------------------------------
 ttinfection |     72,253    201.5884    111.1569          0        365

. tab died_366_730_crossover case if alive_day366_crossover

           |     indicator: is
           |  `index_dt` the date
           | of the first positive
died_366_7 | covid-19 test result
30_crossov |          for
        er |         0          1 |     Total
-----------+----------------------+----------
         0 |   873,923    179,139 | 1,053,062 
         1 |    13,528      2,518 |    16,046 
-----------+----------------------+----------
     Total |   887,451    181,657 | 1,069,108 


. 
. * check mortality by period prior to applying below criteria
. tab died_0_90_crossover case, co

+-------------------+
| Key               |
|-------------------|
```

```
|     frequency     |
| column percentage |
+-------------------+

           |     indicator: is
           |  `index_dt` the date
           | of the first positive
           | covid-19 test result
died_0_90_ |          for
 crossover |         0          1 |     Total
-----------+----------------------+----------
         0 | 1,027,181    195,646 | 1,222,827 
           |     99.01      94.03 |     98.18 
-----------+----------------------+----------
         1 |    10,242     12,415 |    22,657 
           |      0.99       5.97 |      1.82 
-----------+----------------------+----------
     Total | 1,037,423    208,061 | 1,245,484 
           |    100.00     100.00 |    100.00 

. tab died_91_180_crossover case, co

+-------------------+
| Key               |
|-------------------|
|     frequency     |
| column percentage |
+-------------------+

           |     indicator: is
           |  `index_dt` the date
           | of the first positive
died_91_18 | covid-19 test result
0_crossove |          for
         r |         0          1 |     Total
-----------+----------------------+----------
         0 | 1,018,133    193,602 | 1,211,735 
           |     99.12      98.96 |     99.09 
-----------+----------------------+----------
         1 |     9,048      2,044 |    11,092 
           |      0.88       1.04 |      0.91 
-----------+----------------------+----------
     Total | 1,027,181    195,646 | 1,222,827 
           |    100.00     100.00 |    100.00 

. tab died_181_365_crossover case, co

+-------------------+
| Key               |
|-------------------|
|     frequency     |
| column percentage |
+-------------------+
```

```
           |     indicator: is
           |  `index_dt` the date
           | of the first positive
died_181_3 | covid-19 test result
65_crossov |          for
        er |         0          1 |     Total
-----------+----------------------+----------
         0 |   999,281    190,170 | 1,189,451 
           |     98.15      98.23 |     98.16 
-----------+----------------------+----------
         1 |    18,852      3,432 |    22,284 
           |      1.85       1.77 |      1.84 
-----------+----------------------+----------
     Total | 1,018,133    193,602 | 1,211,735 
           |    100.00     100.00 |    100.00 

. tab died_366_730_crossover case, co

+-------------------+
| Key               |
|-------------------|
|     frequency     |
| column percentage |
+-------------------+

           |     indicator: is
           |  `index_dt` the date
           | of the first positive
died_366_7 | covid-19 test result
30_crossov |          for
        er |         0          1 |     Total
-----------+----------------------+----------
         0 |   985,394    187,579 | 1,172,973 
           |     98.62      98.64 |     98.62 
-----------+----------------------+----------
         1 |    13,837      2,582 |    16,419 
           |      1.38       1.36 |      1.38 
-----------+----------------------+----------
     Total |   999,231    190,161 | 1,189,392 
           |    100.00     100.00 |    100.00 

. 
. *----------------------------------------------------------------------------
----------
. * for each period, identify whether the case and at least one 
comparator
. * in a matched group are alive on the first day of the period & were 
not
. * an infection crossover in the prior period
. *----------------------------------------------------------------------------
----------
. 
. * drop previous versions of variable
. 
```

```
. foreach i of numlist 91 181 366 {
  2.         drop alive_day`i'_casecontrol
  3. }

.
. foreach i of numlist 91 181 366 {
  2.
.        //cases
.        gen alive_day`i'_case = 0
  3.         replace alive_day`i'_case = 1 if case==1 &
alive_day`i'_crossover==1
  4.         replace alive_day`i'_case = 0 if case==1 & ttstudyend<`i'
  5.         tab alive_day`i'_case alive_day`i'_crossover if case==1
  6.         sum indextodeath if indextodeath<`i' & case==1
  7.
.        bysort matchgroupnumber: egen alive_day`i'_case_grp =
max(alive_day`i'_case)
  8.
.        //comparators
.        gen alive_day`i'_control = 0
  9.         replace alive_day`i'_control = 1 if case==0 &
alive_day`i'_crossover==1
 10.         replace alive_day`i'_control = 0 if case==0 &
ttstudyend<`i'
 11.
.        tab alive_day`i'_control alive_day`i'_crossover if case==0
 12.         sum indextodeath if indextodeath<`i' & case==0
 13.
.        bysort matchgroupnumber: egen alive_day`i'_control_num =
sum(alive_day`i'_control)
 14.
.        //case and at least one comparator alive on day`i'
.        gen alive_day`i'_crosscasecontrol = 0
 15.         replace alive_day`i'_crosscasecontrol = 1 if
alive_day`i'_case_grp==1 & alive_day`i'_control_num>=1
 16.
.        tab alive_day`i'_crosscasecontrol case
 17. }
(195,646 real changes made)
(0 real changes made)

alive_day9 | alive_day91_crossover
    1_case |         0          1 |     Total
-----------+----------------------+----------
         0 |    12,415          0 |    12,415
         1 |         0    195,646 |   195,646
-----------+----------------------+----------
     Total |    12,415    195,646 |   208,061

    Variable |        Obs        Mean    Std. dev.       Min        Max
-------------+---------------------------------------------------------
indextodea~x |     12,415    22.35747    19.79229          0         90
(1,010,268 real changes made)
(0 real changes made)
```

```
alive_day9 | alive_day91_crossover
 1_control |         0          1 |     Total
-----------+----------------------+----------
         0 |    27,155          0 |    27,155 
         1 |         0  1,010,268 | 1,010,268 
-----------+----------------------+----------
     Total |    27,155  1,010,268 | 1,037,423 

    Variable |        Obs        Mean    Std. dev.       Min        Max
-------------+---------------------------------------------------------
indextodea~x |     11,399      44.554    25.89809          0         90
(1,171,103 real changes made)

           |     indicator: is
           |  `index_dt` the date
           | of the first positive
alive_day9 | covid-19 test result
1_crosscas |          for
  econtrol |         0          1 |     Total
-----------+----------------------+----------
         0 |    61,943     12,438 |    74,381 
         1 |   975,480    195,623 | 1,171,103 
-----------+----------------------+----------
     Total | 1,037,423    208,061 | 1,245,484 
(193,602 real changes made)
(0 real changes made)

           | alive_day181_crossove
alive_day1 |           r
   81_case |         0          1 |     Total
-----------+----------------------+----------
         0 |    14,459          0 |    14,459 
         1 |         0    193,602 |   193,602 
-----------+----------------------+----------
     Total |    14,459    193,602 |   208,061 

    Variable |        Obs        Mean    Std. dev.       Min        Max
-------------+---------------------------------------------------------
indextodea~x |     14,459    38.05222    43.91964          0        180
(988,487 real changes made)
(0 real changes made)

           | alive_day181_crossove
alive_day1 |           r
81_control |         0          1 |     Total
-----------+----------------------+----------
         0 |    48,936          0 |    48,936 
         1 |         0    988,487 |   988,487 
-----------+----------------------+----------
     Total |    48,936    988,487 | 1,037,423 

    Variable |        Obs        Mean    Std. dev.       Min        Max
-------------+---------------------------------------------------------
```

```
indextodea~x |     21,714    87.84411    52.42273          0        180
(1,158,838 real changes made)

           |     indicator: is
           |  `index_dt` the date
           | of the first positive
alive_day1 | covid-19 test result
81_crossca |          for
 secontrol |         0          1 |     Total
-----------+----------------------+----------
         0 |    72,147     14,499 |    86,646
         1 |   965,276    193,562 | 1,158,838
-----------+----------------------+----------
     Total | 1,037,423    208,061 | 1,245,484
(181,657 real changes made)
(0 real changes made)

           | alive_day366_crossove
alive_day3 |          r
   66_case |         0          1 |     Total
-----------+----------------------+----------
         0 |    26,404          0 |    26,404
         1 |         0    181,657 |   181,657
-----------+----------------------+----------
     Total |    26,404    181,657 |   208,061

    Variable |        Obs        Mean    Std. dev.       Min        Max
-------------+---------------------------------------------------------
indextodea~x |     17,900    83.07486    103.0125          0        365
(887,451 real changes made)
(0 real changes made)

           | alive_day366_crossove
alive_day3 |          r
66_control |         0          1 |     Total
-----------+----------------------+----------
         0 |   149,972          0 |   149,972
         1 |         0    887,451 |   887,451
-----------+----------------------+----------
     Total |   149,972    887,451 | 1,037,423

    Variable |        Obs        Mean    Std. dev.       Min        Max
-------------+---------------------------------------------------------
indextodea~x |     44,403       182.9    106.9272          0        365
(1,087,393 real changes made)

           |     indicator: is
           |  `index_dt` the date
           | of the first positive
alive_day3 | covid-19 test result
66_crossca |          for
 secontrol |         0          1 |     Total
-----------+----------------------+----------
         0 |   131,633     26,458 |   158,091
```

```
         1 |   905,790    181,603 | 1,087,393
-----------+----------------------+----------
     Total | 1,037,423    208,061 | 1,245,484

.
. * drop variables we no longer need
.
. foreach i of numlist 91 181 366 {
  2.         drop alive_day`i'_case alive_day`i'_case_grp
alive_day`i'_control alive_day`i'_control_num
  3. }

.
. *---------------------------------------------------
. * Create categories for PP1 subgroup analyses
. *---------------------------------------------------
.
. * Note: all categories are in relation to the original infected case *
.
. ** age categories **
.
. * <65
. sum ageatindexdate

    Variable |        Obs        Mean    Std. dev.       Min        Max
-------------+---------------------------------------------------------
ageatinde~te |  1,245,484    60.47358    16.41887   18.88493   99.99726

.
. gen mkg_age_less65_case = 0

. replace mkg_age_less65=1 if case==1 & ageatindexdate<65
(115,041 real changes made)

.
. bysort matchgroupnumber (patienticn): egen age_less65_case =
max(mkg_age_less65_case)

. tab age_less65_case

age_less65_ |
       case |      Freq.     Percent        Cum.
------------+-----------------------------------
          0 |    557,143       44.73       44.73
          1 |    688,341       55.27      100.00
------------+-----------------------------------
      Total |  1,245,484      100.00

.
. drop mkg_age_less65_case

.
. * 65-85
. gen mkg_age_65to85_case = 0
```

```
. replace mkg_age_65to85_case=1 if case==1 & inrange(ageatindexdate, 65,
85)
(82,301 real changes made)

.
. bysort matchgroupnumber (patienticn): egen age_65to85_case =
max(mkg_age_65to85_case)

. tab age_65to85_case

age_65to85_ |
       case |      Freq.     Percent        Cum.
------------+-----------------------------------
          0 |    752,514       60.42       60.42
          1 |    492,970       39.58      100.00
------------+-----------------------------------
      Total |  1,245,484      100.00

.
. drop mkg_age_65to85_case

.
. * >85
. gen mkg_age_more85_case = 0

. replace mkg_age_more85_case=1 if case==1 & ageatindexdate>85
(10,719 real changes made)

.
. bysort matchgroupnumber (patienticn): egen age_more85_case =
max(mkg_age_more85_case)

. tab age_more85_case

age_more85_ |
       case |      Freq.     Percent        Cum.
------------+-----------------------------------
          0 |  1,181,311       94.85       94.85
          1 |     64,173        5.15      100.00
------------+-----------------------------------
      Total |  1,245,484      100.00

.
. drop mkg_age_more85_case

.
.
. ** Tertiles of Gagne scores **
. sum gagne if case==1, de

                gagne score (min=-2, max=24)
-------------------------------------------------------------
      Percentiles      Smallest
```

```
 1%           -1             -2
 5%           -1             -2
10%           -1             -2       Obs             208,061
25%            0             -2       Sum of wgt.     208,061

50%            1                      Mean           1.440803
                        Largest       Std. dev.      2.341555
75%            2             19
90%            4             20       Variance       5.482881
95%            6             20       Skewness       1.971733
99%           10             20       Kurtosis       8.011107

. xtile mkg_gagne_tertile = gagne if case==1, nq(3)

.
. bysort mkg_gagne_tertile: sum gagne

--------------------------------------------------------------------------
--------------------------------------------------------------------------
------------------------------------------------
-> mkg_gagne_tertile = 1

    Variable |        Obs        Mean    Std. dev.       Min        Max
-------------+---------------------------------------------------------
       gagne |     93,002   -.2447689    .4331417         -2          0

--------------------------------------------------------------------------
--------------------------------------------------------------------------
------------------------------------------------
-> mkg_gagne_tertile = 2

    Variable |        Obs        Mean    Std. dev.       Min        Max
-------------+---------------------------------------------------------
       gagne |     70,162    1.358656    .4796093          1          2

--------------------------------------------------------------------------
--------------------------------------------------------------------------
------------------------------------------------
-> mkg_gagne_tertile = 3

    Variable |        Obs        Mean    Std. dev.       Min        Max
-------------+---------------------------------------------------------
       gagne |     44,897    5.060761    2.379211          3         20

--------------------------------------------------------------------------
--------------------------------------------------------------------------
------------------------------------------------
-> mkg_gagne_tertile = .

    Variable |        Obs        Mean    Std. dev.       Min        Max
-------------+---------------------------------------------------------
       gagne |  1,037,423    1.402901    2.238274         -2         22
```

```
.
. bysort matchgroupnumber (patienticn): egen gagne_tertile_case =
max(mkg_gagne_tertile)

. sum gagne_tertile_case

    Variable |        Obs        Mean    Std. dev.       Min        Max
-------------+---------------------------------------------------------
gagne_tert~e |  1,245,484    1.769205    .7805706          1          3

.
. ** Probabilities of death in first 90 days **
.
. * fit logistic regression model for comparator deaths during first 90
days
. local covar ageatindexdate bmi i.sex3cat i.race7cat i.ethnicity3cat
///
>                          i.rurality2cat i.smoking4cat gagne numipadmits
util_numpcstops ///
>                          util_numscstops util_nummhstops i.immuno
i.clcatindexdate ///
>                          i.nosos11cat i.canscore7cat
neareststa3ndistance

.
. logit died_0_90 `covar' if case==0

Iteration 0:   log likelihood = -62756.685
Iteration 1:   log likelihood = -55368.077
Iteration 2:   log likelihood =  -52670.45
Iteration 3:   log likelihood = -52494.908
Iteration 4:   log likelihood = -52491.217
Iteration 5:   log likelihood = -52491.201
Iteration 6:   log likelihood = -52491.201

Logistic regression                                     Number of obs =
1,037,423
                                                        LR chi2(40)   =
20530.97
                                                        Prob > chi2   =
0.0000
Log likelihood = -52491.201                             Pseudo R2     =
0.1636

----------------------------------------------------------------------------
-----------------------
                    died_0_90 | Coefficient  Std. err.      z    P>|z|
[95% conf. interval]
------------------------------+---------------------------------------------
-----------------------
               ageatindexdate |   .0501644   .0010291    48.75   0.000
.0481474    .0521813
                          bmi |  -.0216606   .0016231   -13.35   0.000
-.0248418   -.0184793
```

sex3cat						
Male	.4578403	.0621326	7.37	0.000	.3360626	.579618
Unknown	.7157964	.1086869	6.59	0.000	.502774	.9288188
race7cat						
Asian	-.3956703	.1705535	-2.32	0.020	-.729949	-.0613915
Black/AfricanAmer	-.3923309	.0998636	-3.93	0.000	-.58806	-.1966019
NativeHawaiian/PacificIsland	-.1711533	.1416686	-1.21	0.227	-.4488187	.106512
White	-.2352478	.0975313	-2.41	0.016	-.4264057	-.0440899
MultipleRace	-.3396909	.1526475	-2.23	0.026	-.6388744	-.0405073
Missing	-.2915382	.1102476	-2.64	0.008	-.5076195	-.075457
ethnicity3cat						
NotHispanic	.0024398	.0417852	0.06	0.953	-.0794577	.0843372
Missing	.0287298	.0693445	0.41	0.679	-.107183	.1646426
rurality2cat						
Urban	-.014362	.0233265	-0.62	0.538	-.0600811	.0313572
smoking4cat						
Former	-.1434746	.0317828	-4.51	0.000	-.2057677	-.0811814
Never	-.2075364	.033906	-6.12	0.000	-.273991	-.1410818
Missing	.3945889	.0502605	7.85	0.000	.2960801	.4930977
gagne	.1579537	.0040056	39.43	0.000	.1501028	.1658046
numipadmits	-.0024634	.005221	-0.47	0.637	-.0126963	.0077696
util_numpcstops	.007242	.0009396	7.71	0.000	.0054003	.0090836
util_numscstops	-.0113454	.0008449	-13.43	0.000	-.0130013	-.0096896
util_nummhstops	-.0032296	.0005546	-5.82	0.000	-.0043166	-.0021426
1.immuno	.1424113	.0268694	5.30	0.000	.0897482	.1950744
1.clcatindexdate	.1860423	.0523672	3.55	0.000	.0834044	.2886801

```
                 nosos11cat |
                          2  |  -.3261046   .1031314    -3.16   0.002
-.5282385   -.1239708
                          3  |  -.0482072   .0932017    -0.52   0.605
-.2308791    .1344648
                          4  |  -.1992918   .0910699    -2.19   0.029
-.3777856   -.0207981
                          5  |  -.3142041   .0894185    -3.51   0.000
-.4894611   -.1389471
                          6  |  -.4216598   .0878902    -4.80   0.000
-.5939213   -.2493982
                          7  |  -.5351033    .086918    -6.16   0.000
-.7054595   -.3647472
                          8  |  -.5075741   .0854003    -5.94   0.000
-.6749556   -.3401927
                          9  |  -.5565842   .0848727    -6.56   0.000
-.7229317   -.3902367
                         10  |  -.3988552   .0850978    -4.69   0.000
-.5656438   -.2320665
                         99  |  -.0005753   .1173074    -0.00   0.996
-.2304936    .2293431
                             |
               canscore7cat |
                          2  |   .7516619   .0970557     7.74   0.000
.5614362    .9418876
                          3  |   1.249969   .0908691    13.76   0.000
1.071869    1.428069
                          4  |     1.6701   .0897059    18.62   0.000
1.49428    1.845921
                          5  |   2.056566   .0920936    22.33   0.000
1.876066    2.237067
                          6  |   2.388942   .0956076    24.99   0.000
2.201554    2.576329
                         99  |   1.596729   .1201958    13.28   0.000
1.36115    1.832309
                             |
       neareststa3ndistance |   .0024652   .0002929     8.42   0.000
.0018912    .0030393
                      _cons |   -9.01926   .1820659   -49.54   0.000
-9.376103   -8.662418
------------------------------------------------------------------------
-----------------------

. lroc, nograph //0.846

Logistic model for died_0_90

Number of observations =  1037423
Area under ROC curve   =   0.8459

. predict mkg_pr_died0_90
(option pr assumed; Pr(died_0_90))

. gen mkg_pr_died0_90_case = mkg_pr_died0_90 if case==1
```

```
(1,037,423 missing values generated)

.
. bysort matchgroupnumber (patienticn): egen pr_died0_90_case =
max(mkg_pr_died0_90_case)

.
. * create tertiles of predicted probabilities of death in first 90 days
. xtile pr_died0_90_tertile = pr_died0_90_case, nq(3)

. tab pr_died0_90_tertile

3 quantiles |
         of |
pr_died0_90 |
      _case |      Freq.     Percent        Cum.
------------+-----------------------------------
          1 |    415,167       33.33       33.33
          2 |    415,160       33.33       66.67
          3 |    415,157       33.33      100.00
------------+-----------------------------------
      Total |  1,245,484      100.00

.
. bysort pr_died0_90_tertile: sum pr_died0_90_case

-------------------------------------------------------------------------
-------------------------------------------------------------------------
-----------------------------------------------
-> pr_died0_90_tertile = 1

    Variable |        Obs        Mean    Std. dev.       Min        Max
-------------+---------------------------------------------------------
pr_died0_~se |    415,167    .0008415    .0004402   .0000576   .0017813

-------------------------------------------------------------------------
-------------------------------------------------------------------------
-----------------------------------------------
-> pr_died0_90_tertile = 2

    Variable |        Obs        Mean    Std. dev.       Min        Max
-------------+---------------------------------------------------------
pr_died0_~se |    415,160    .0042522    .0018158   .0017814   .0081787

-------------------------------------------------------------------------
-------------------------------------------------------------------------
-----------------------------------------------
-> pr_died0_90_tertile = 3

    Variable |        Obs        Mean    Std. dev.       Min        Max
-------------+---------------------------------------------------------
pr_died0_~se |    415,157    .0306809    .0323484   .0081789   .5654742
```

```
.
.
. ** Probabilities of death in days 181-730 **
.
. * died in days 181-730
. gen died_181_730 = !missing(best_death_date)

. replace died_181_730 = 0 if indextodeath_caseindex<181 |
indextodeath_caseindex>730
(37,561 real changes made)

.
. * fit logistic regression model for comparator deaths during 181-730
days
. local covar ageatindexdate bmi i.sex3cat i.race7cat i.ethnicity3cat
///
>                          i.rurality2cat i.smoking4cat gagne numipadmits
util_numpcstops ///
>                          util_numscstops util_nummhstops i.immuno
i.clcatindexdate ///
>                          i.nosos11cat i.canscore7cat
neareststa3ndistance

.
. logit died_181_730 `covar' if case==0

Iteration 0:   log likelihood = -204114.37
Iteration 1:   log likelihood = -191425.15
Iteration 2:   log likelihood = -166974.46
Iteration 3:   log likelihood = -166176.34
Iteration 4:   log likelihood =  -166166.2
Iteration 5:   log likelihood = -166166.17
Iteration 6:   log likelihood = -166166.17

Logistic regression                                     Number of obs =
1,037,423
                                                        LR chi2(40)   =
75896.39
                                                        Prob > chi2   =
0.0000
Log likelihood = -166166.17                             Pseudo R2     =
0.1859

----------------------------------------------------------------------------
-----------------------
                 died_181_730 | Coefficient  Std. err.      z    P>|z|
[95% conf. interval]
-----------------------------+----------------------------------------------
-----------------------
               ageatindexdate |   .0577912   .0005167   111.84   0.000
.0567784     .058804
                          bmi |  -.0113613   .0007813   -14.54   0.000
-.0128925     -.00983
                              |
```

sex3cat						
Male	.4820631	.0289214	16.67	0.000	.4253782	.538748
Unknown	.6188037	.0536677	11.53	0.000	.513617	.7239904
race7cat						
Asian	-.5767519	.0905526	-6.37	0.000	-.7542318	-.399272
Black/AfricanAmer	-.3593859	.0514899	-6.98	0.000	-.4603043	-.2584675
NativeHawaiian/PacificIsland	-.1970492	.0733454	-2.69	0.007	-.3408036	-.0532948
White	-.1911021	.0503895	-3.79	0.000	-.2898638	-.0923404
MultipleRace	-.1773534	.0748844	-2.37	0.018	-.3241243	-.0305826
Missing	-.1394429	.0562494	-2.48	0.013	-.2496898	-.0291961
ethnicity3cat						
NotHispanic	.1783973	.0216559	8.24	0.000	.1359525	.2208421
Missing	.1477052	.0350344	4.22	0.000	.079039	.2163714
rurality2cat						
Urban	.0058641	.0115908	0.51	0.613	-.0168534	.0285816
smoking4cat						
Former	-.2749115	.0153597	-17.90	0.000	-.305016	-.244807
Never	-.3506341	.0163387	-21.46	0.000	-.3826574	-.3186108
Missing	.2174937	.0260512	8.35	0.000	.1664343	.2685531
gagne	.1285267	.0021927	58.61	0.000	.1242291	.1328244
numipadmits	-.0054117	.002982	-1.81	0.070	-.0112564	.0004329
util_numpcstops	.0020919	.0005356	3.91	0.000	.0010422	.0031416
util_numscstops	-.0056286	.0004423	-12.72	0.000	-.0064955	-.0047616
util_nummhstops	-.0023689	.0002572	-9.21	0.000	-.002873	-.0018648
1.immuno	.0438204	.0142137	3.08	0.002	.0159621	.0716787
1.clcatindexdate	.1551883	.0306509	5.06	0.000	.0951136	.215263
nosos11cat						

```
                          2  |  -.1550357   .0493244    -3.14   0.002
-.2517097   -.0583618
                          3  |  -.1285367    .046825    -2.75   0.006
-.2203121   -.0367613
                          4  |  -.2072587   .0453812    -4.57   0.000
-.2962042   -.1183132
                          5  |  -.2603685   .0443569    -5.87   0.000
-.3473065   -.1734305
                          6  |   -.273505   .0434103    -6.30   0.000
-.3585877   -.1884224
                          7  |  -.3268706   .0429328    -7.61   0.000
-.4110172   -.2427239
                          8  |  -.3649313   .0425728    -8.57   0.000
-.4483724   -.2814902
                          9  |  -.3671297   .0424201    -8.65   0.000
-.4502715   -.2839878
                         10  |  -.1728259    .042793    -4.04   0.000
-.2566986   -.0889533
                         99  |   .0606899    .059871     1.01   0.311
-.056655    .1780349
                             |
               canscore7cat |
                          2  |   .6449698   .0398461    16.19   0.000
.5668728    .7230668
                          3  |   1.043083   .0376195    27.73   0.000
.9693501    1.116816
                          4  |   1.404931    .037375    37.59   0.000
1.331678    1.478185
                          5  |    1.73882   .0388632    44.74   0.000
1.662649     1.81499
                          6  |   1.947474    .041145    47.33   0.000
1.866831    2.028117
                         99  |   1.040115   .0581389    17.89   0.000
.9261645    1.154065
                             |
       neareststa3ndistance |   .0020697   .0001474    14.04   0.000
.0017808    .0023586
                      _cons |  -8.124368    .088594   -91.70   0.000
-8.298009   -7.950727
--------------------------------------------------------------------------
-----------------------

. lroc, nograph //0.830

Logistic model for died_181_730

Number of observations =  1037423
Area under ROC curve   =   0.8302

. predict mkg_pr_died181_730
(option pr assumed; Pr(died_181_730))

. gen mkg_pr_died181_730_case = mkg_pr_died181_730 if case==1
(1,037,423 missing values generated)
```

```
.
. bysort matchgroupnumber (patienticn): egen pr_died181_730_case =
max(mkg_pr_died181_730_case)

.
. * create tertiles of predicted probabilities of death in 181-730 days
. xtile pr_died181_730_tertile = pr_died181_730_case, nq(3)

. tab pr_died181_730_tertile

3 quantiles |
         of |
pr_died181_ |
   730_case |      Freq.     Percent        Cum.
------------+-----------------------------------
          1 |    415,164       33.33       33.33
          2 |    415,160       33.33       66.67
          3 |    415,160       33.33      100.00
------------+-----------------------------------
      Total |  1,245,484      100.00

.
. bysort pr_died181_730_tertile: sum pr_died181_730_case

-----------------------------------------------------------------------------
-----------------------------------------------------------------------------
-----------------------------------------------
-> pr_died181_730_tertile = 1

    Variable |        Obs        Mean    Std. dev.       Min        Max
-------------+---------------------------------------------------------
pr_died18~se |    415,164     .004442    .0024142   .0003538     .00979

-----------------------------------------------------------------------------
-----------------------------------------------------------------------------
-----------------------------------------------
-> pr_died181_730_tertile = 2

    Variable |        Obs        Mean    Std. dev.       Min        Max
-------------+---------------------------------------------------------
pr_died18~se |    415,160    .0237697    .0100769     .00979   .0450446

-----------------------------------------------------------------------------
-----------------------------------------------------------------------------
-----------------------------------------------
-> pr_died181_730_tertile = 3

    Variable |        Obs        Mean    Std. dev.       Min        Max
-------------+---------------------------------------------------------
pr_died18~se |    415,160    .1277293    .0909023   .0450463   .8045333


.
```

```
.
. drop mkg_pr*

.
. *-------------------------------------------------------
. * PP1 mortality for COVID+ Veterans and comparators
. *-------------------------------------------------------
.
.         /*
>
>         Criteria
>            1)   Case and at least 1 comparator in the strata must be
alive on the
>                         first day of the period
>
>            2)   Patients who were censored due to an infection
crossover
>                         in the previous period are excluded in the
current period
>
>            3)   Patients who die after the infection crossover are
alive
>                         at the time of the infection crossover
>         */
.
.
.
. * Overall *
.
. * 90-day mortality
. tab died_0_90_crossover infected , co

+-------------------+
| Key               |
|-------------------|
|     frequency     |
| column percentage |
+-------------------+

died_0_90_ |       infected
 crossover |         0          1 |     Total
-----------+----------------------+----------
         0 | 1,027,181    195,646 | 1,222,827
           |     99.01      94.03 |     98.18
-----------+----------------------+----------
         1 |    10,242     12,415 |    22,657
           |      0.99       5.97 |      1.82
-----------+----------------------+----------
     Total | 1,037,423    208,061 | 1,245,484
           |    100.00     100.00 |    100.00

. cs died_0_90_crossover infected

                 |        infected        |
```

```
                 |   Exposed   Unexposed  |      Total
-----------------+------------------------+------------
           Cases |     12415       10242  |      22657
        Noncases |    195646     1027181  |    1222827
-----------------+------------------------+------------
           Total |    208061     1037423  |    1245484
                 |                        |
            Risk |    .05967    .0098725  |   .0181913
                 |                        |
                 |      Point estimate    |    [95% conf. interval]
                 |------------------------+------------------------
 Risk difference |         .0497975       |     .048762    .0508329
      Risk ratio |         6.044037       |    5.890475    6.201604
 Attr. frac. ex. |         .8345477       |    .8302344    .8387514
 Attr. frac. pop |          .457294       |
                 +-------------------------------------------------
                               chi2(1) = 24062.00  Pr>chi2 = 0.0000

.
. * 180-day mortality
. tab died_91_180_crossover infected if alive_day91_crosscasecontrol==1
, co

+-------------------+
| Key               |
|-------------------|
|     frequency     |
| column percentage |
+-------------------+

died_91_18 |
0_crossove |       infected
         r |         0          1 |     Total
-----------+----------------------+----------
         0 |   957,712    193,579 | 1,151,291
           |     99.14      98.96 |     99.11
-----------+----------------------+----------
         1 |     8,335      2,044 |    10,379
           |      0.86       1.04 |      0.89
-----------+----------------------+----------
     Total |   966,047    195,623 | 1,161,670
           |    100.00     100.00 |    100.00

. cs died_91_180_crossover infected if alive_day91_crosscasecontrol==1

                 |         infected       |
                 |   Exposed   Unexposed  |      Total
-----------------+------------------------+------------
           Cases |      2044        8335  |      10379
        Noncases |    193579      957712  |    1151291
-----------------+------------------------+------------
           Total |    195623      966047  |    1161670
                 |                        |
            Risk |  .0104487    .0086279  |   .0089346
```

```
                 |                          |
                 |      Point estimate      |    [95% conf. interval]
                 |--------------------------+------------------------
 Risk difference |         .0018207         |    .0013338    .0023076
      Risk ratio |         1.211026         |    1.154118    1.270741
 Attr. frac. ex. |         .1742542         |    .1335377    .2130574
 Attr. frac. pop |         .0343169         |
                 +-------------------------------------------------
                               chi2(1) =    60.90  Pr>chi2 = 0.0000

.
. * 365-day mortality
. tab died_181_365_crossover infected if
alive_day181_crosscasecontrol==1 , co

+-------------------+
| Key               |
|-------------------|
|     frequency     |
| column percentage |
+-------------------+

died_181_3 |
65_crossov |       infected
        er |         0          1 |     Total
-----------+----------------------+----------
         0 |   930,617    190,130 | 1,120,747
           |     98.19      98.23 |     98.20
-----------+----------------------+----------
         1 |    17,158      3,432 |    20,590
           |      1.81       1.77 |      1.80
-----------+----------------------+----------
     Total |   947,775    193,562 | 1,141,337
           |    100.00     100.00 |    100.00

. cs died_181_365_crossover infected if alive_day181_crosscasecontrol==1

                 |        infected          |
                 |   Exposed   Unexposed    |      Total
-----------------+--------------------------+-----------
           Cases |      3432       17158    |      20590
        Noncases |    190130      930617    |    1120747
-----------------+--------------------------+-----------
           Total |    193562      947775    |    1141337
                 |                          |
            Risk |  .0177308    .0181035    |   .0180402
                 |                          |
                 |      Point estimate      |    [95% conf. interval]
                 |--------------------------+------------------------
 Risk difference |        -.0003727         |    -.001019    .0002736
      Risk ratio |         .9794128         |    .9444768    1.015641
 Prev. frac. ex. |         .0205872         |   -.0156411    .0555232
 Prev. frac. pop |         .0034914         |
                 +-------------------------------------------------
```

```
                                 chi2(1) =     1.26  Pr>chi2 = 0.2616

.
. * 730-day mortality
. tab died_366_730_crossover infected if
alive_day366_crosscasecontrol==1, co

+-------------------+
| Key               |
|-------------------|
|     frequency     |
| column percentage |
+-------------------+

died_366_7 |
30_crossov |       infected
        er |         0          1 |     Total
-----------+----------------------+----------
         0 |   861,248    179,086 | 1,040,334
           |     98.64      98.61 |     98.64
-----------+----------------------+----------
         1 |    11,877      2,517 |    14,394
           |      1.36       1.39 |      1.36
-----------+----------------------+----------
     Total |   873,125    181,603 | 1,054,728
           |    100.00     100.00 |    100.00

. cs died_366_730_crossover infected if alive_day366_crosscasecontrol==1

                 |        infected         |
                 |   Exposed   Unexposed   |      Total
-----------------+-------------------------+-----------
           Cases |      2517       11877   |      14394
        Noncases |    179086      861248   |    1040334
-----------------+-------------------------+-----------
           Total |    181603      873125   |    1054728
                 |                         |
            Risk |  .0138599    .0136029   |   .0136471
                 |                         |
                 |      Point estimate     |    [95% conf. interval]
                 |-------------------------+------------------------
 Risk difference |          .000257        |    -.000333    .0008471
      Risk ratio |         1.018896        |    .9762958    1.063355
 Attr. frac. ex. |         .0185456        |   -.0242797    .0595803
 Attr. frac. pop |          .003243        |
                 +--------------------------------------------------
                               chi2(1) =     0.74  Pr>chi2 = 0.3903

.
.
.
*************************************************************************
*
. * Per Protocol #1 - Cox Models: Censoring at Crossover Infections
```

```
(Unadjusted) *
.
************************************************************************
*
.
. *---------------------------
. * Prepping for Cox Models
. *---------------------------
.
. ** Censoring at 730 Days -Or- Among Comparators, at Infection
Crossover **
.
. * count number who were COVID+ prior to death or study end
. count if ttinfection730
(13,998 real changes made)

. replace tte_730_cross = indextodeath_caseindex if
indextodeath_caseindex730
(1,150,277 real changes made)

. replace died_730_cross = 0 if indextodeath_caseindex>ttinfection
(12,311 real changes made)

. replace died_730_cross = 0 if (indextodeath_caseindex>ttstudyend) &
(inrange(indextodeath_caseindex, 0, 730)) ///
>
& (inrange(ttstudyend, 0, 730))
(10,817 real changes made)

.
. tab died_730_cross

died_730_cr |
        oss |      Freq.     Percent        Cum.
------------+-----------------------------------
          0 |  1,173,405       94.21       94.21
          1 |     72,079        5.79      100.00
------------+-----------------------------------
      Total |  1,245,484      100.00

.
. *--------------------------------
. * Per Protocol #1: Cox Models
. *--------------------------------
.
. preserve

.
.          * stset data
.          sort uniq_patid

.          stset tte_730_cross, failure(died_730_cross) id(uniq_patid)

Survival-time data settings
```

```
           ID variable: uniq_patid
         Failure event: died_730_cross!=0 & died_730_cross<.
Observed time interval: (tte_730_cross[_n-1], tte_730_cross]
     Exit on or before: failure

------------------------------------------------------------------------
--
  1,245,484  total observations
        347  observations end on or before enter()
------------------------------------------------------------------------
--
  1,245,137  observations remaining, representing
  1,245,137  subjects
     71,738  failures in single-failure-per-subject data
  575765083  total analysis time at risk and under observation
                                                At risk from t =
0
                                     Earliest observed entry t =
0
                                          Last observed exit t =
730

.         stsplit period, at(0 90 180 365 730)
(3,457,111 observations (episodes) created)

.
.         * create new interaction variables after splitting data
.         gen timeinfected_0_90 = infected * (tte_730_cross<=90)

.         gen timeinfected_91_180 = infected * (tte_730_cross>90 &
tte_730_cross<=180)

.         gen timeinfected_181_365 = infected * (tte_730_cross>180 &
tte_730_cross<=365)

.         gen timeinfected_366_730 = infected * (tte_730_cross>365 &
tte_730_cross<=730)


.
.         * cox model
.         stcox   i.timeinfected_0_90 i.timeinfected_91_180
i.timeinfected_181_365  ///
>                         i.timeinfected_366_730
///
>                         , efron vce(cluster patienticn)
strata(matchgroupnumber)

        Failure _d: died_730_cross
  Analysis time _t: tte_730_cross
       ID variable: uniq_patid

Iteration 0:   log pseudolikelihood = -123060.13
Iteration 1:   log pseudolikelihood = -116684.28
```

```
Iteration 2:   log pseudolikelihood = -114516.47
Iteration 3:   log pseudolikelihood = -114388.85
Iteration 4:   log pseudolikelihood = -114388.84
Refining estimates:
Iteration 0:   log pseudolikelihood = -114388.84

Stratified Cox regression with Efron method for ties
Strata variable: matchgroupnumber

No. of subjects = 1,245,137                           Number of obs =
4,702,248
No. of failures =    71,738
Time at risk    = 575765083
                                                      Wald chi2(4)  =
22970.84
Log pseudolikelihood = -114388.84                     Prob > chi2   =
0.0000

                               (Std. err. adjusted for 1,091,935
clusters in patienticn)
------------------------------------------------------------------------
----------------
                       |               Robust
                    _t | Haz. ratio   std. err.      z    P>|z|     [95%
conf. interval]
-----------------------+------------------------------------------------
----------------
   1.timeinfected_0_90 |   6.357467   .0777116   151.32   0.000
6.206965    6.511618
 1.timeinfected_91_180 |   1.176034   .0264245     7.22   0.000
1.125366    1.228982
1.timeinfected_181_365 |    .920783   .0164475    -4.62   0.000
.8891043    .9535903
1.timeinfected_366_730 |   .8922076   .0181327    -5.61   0.000
.8573668    .9284643
------------------------------------------------------------------------
----------------


.
. restore

.
.
.
. *-------------------------------
. * Preparing dataset for IPCWs
. *-------------------------------
.
. *save Data\prepdata_pp1_ipcw, replace
.
. preserve

.
.         * open dataset
```

```
.         use Data\prepdata_pp1_ipcw, clear
(Created in file
'P:\ORD_Ioannou_202104007D\MethodsGroup\Programs\CORC_DataExplor)

.
.         * create a fractional tte_730_cross variable by dividing by 32
.         gen tte_month_cross = tte_730_cross/32

.
.         * keep only variables needed for analysis
.         keep    /* patient identifiers*/
///
>                         matchgroupnumber patienticn uniq_patid case
index_dt    ///
>                         index_dt_case
///
>                         /* covariates */
///
>                         ageatindexdate bmi ethnicity3cat smoking4cat
///
>                         gagne numipadmits util_numpcstops
util_numscstops             ///
>                         nosos11cat canscore7cat nearests ta3ndistance
///
>                         /*variables for subgroup analyses*/
///
>                         pr_died0_90_tertile pr_died181_730_tertile
///
>                         age_less65_case age_65to85_case
age_more85_case               ///
>                         gagne_tertile_case
///
>                         /*variables for cox models*/
///
>                         tte_month_cross died_730_cross tte_730_cross

.
.         * organize dataset
.         order uniq_patid, after(patienticn)

.         order index_dt_case, after(index_dt)

.
.         * set up dataset for analysis in SAS
.         expand tte_month_cross +1 //+1 so that we can include  month 0
(1,198,827 noninteger counts rounded to integer)
(18,019,710 observations created)

.         bysort matchgroupnumber patienticn: gen monthend=_n

.
.         gen month = monthend-1

.
```

```
.         * drop expanded months in which the tte month is less than or
equal to the
.         * start of the month; in the expand command, tte months that
were x.5
.         * added an additional month. we want to confirm that the tte
month
.         * always exceeds the start of the month
.         drop if tte_month_cross<=month & tte_month_cross!=0
(670,801 observations deleted)

.
.         * create mortality variable
.         bysort matchgroupnumber patienticn: gen N = _N

.         gen died = 0

.         replace died = 1 if died_730_cross==1 & monthend==N
(72,079 real changes made)

.
.         * identify the last observation in the panel. this will be
used for
.         * filling in the new tte variable created (below) with the
value in
.         * the tte_month_cross variable for the last month the subject
is in the study
.         gsort matchgroupnumber patienticn -month

.         by matchgroupnumber patienticn: gen lastnum = _n


.
.         * create tte
.         gen tte=monthend


.
.                 * replace the new tte with the tte_month_cross
variable in the month when
.                 * tte occurs
.                 replace tte = tte_month_cross if lastnum==1
(1,199,174 real changes made)


.
.         sort matchgroupnumber patienticn month


.
.         * censor each month unless it is a month when a patient dies
.         gen censor = 1

.         replace censor = 0 if died==1
(72,079 real changes made)

.
.         * merge in IPCWs
.         bysort matchgroupnumber patienticn (monthend): gen panel_n=_n
```

```
.         merge 1:1 matchgroupnumber patienticn panel_n using
Data\IPCW\ipcw_trunc

    Result                      Number of obs
    -----------------------------------------
    Not matched                     2,112,906
        from master                         0  (_merge==1)
        from using                  2,112,906  (_merge==2)

    Matched                        18,594,393  (_merge==3)
    -----------------------------------------

.         drop if _merge==2
(2,112,906 observations deleted)

.
.         * drop variables not needed for analysis
.         drop died_730_cross tte_730_cross tte_month_cross monthend N
smdate year _merge panel_n

.
.         * organize variables in dataset
.         order matchgroupnumber patienticn uniq_patid case index_dt
index_dt_case month tte died censor ipcw

.
.         * save dataset for inclusion of IPCWs
.         *save Data\mortality_pp1_ipcw_20221215, replace
.
. restore

.
.
. **********************************
. * PP2: With Censoring, Unweighted *
. **********************************
.
. use Data\prepdata_pp1_ipcw, clear
(Created in file
'P:\ORD_Ioannou_202104007D\MethodsGroup\Programs\CORC_DataExplor)

.
. * check ttinfection with negative values
. sum ttinfection

    Variable |        Obs        Mean    Std. dev.       Min        Max
-------------+---------------------------------------------------------
 ttinfection |    137,326    338.8583    179.3397          0        867

. count if ttinfection==0
  6

.
```

```
. * identify the earliest infection crossover for the strata
. gsort matchgroupnumber propensityscoreabsdiffrank -case

. by matchgroupnumber: egen ttinfection_strata = min(ttinfection)
(630,819 missing values generated)


.
. *---------------------------------------------------------------------
----------
. * identify those who die during each period;
. * those who die after infection crossover are coded as not dead at the
time of
. * crossover
. *---------------------------------------------------------------------
----------
.
. * Died Index Day-Day 90;
. gen died_0_90_stratacrossover = .
(1,245,484 missing values generated)

. replace died_0_90_stratacrossover = 1 if
inrange(indextodeath_caseindex, 0, 90)
(23,814 real changes made)

. replace died_0_90_stratacrossover = 0 if indextodeath_caseindex>90
(1,221,670 real changes made)

. replace died_0_90_stratacrossover = 0 if
indextodeath_caseindex>ttinfection_strata
(1,685 real changes made)

. tab died_0_90 died_0_90_stratacrossover, m

           | died_0_90_stratacross
           |         over
 died_0_90 |         0          1 |     Total
-----------+----------------------+----------
         0 | 1,221,670          0 | 1,221,670
         1 |     1,685     22,129 |    23,814
-----------+----------------------+----------
     Total | 1,223,355     22,129 | 1,245,484


.
. * Died Day 91-180;
. gen died_91_180_stratacrossover = .
(1,245,484 missing values generated)

. replace died_91_180_stratacrossover = 1 if
inrange(indextodeath_caseindex, 91, 180)
(12,359 real changes made)

. replace died_91_180_stratacrossover = 0 if indextodeath_caseindex>180
(1,209,311 real changes made)
```

```
. replace died_91_180_stratacrossover = 0 if
indextodeath_caseindex>ttinfection_strata
(4,058 real changes made)

. replace died_91_180_stratacrossover = 0 if
(indextodeath_caseindex>ttstudyend) & (inrange(indextodeath_caseindex,
91, 180)) ///
>
& (inrange(ttstudyend, 91, 180))
(0 real changes made)

. tab died_91_180 died_91_180_stratacrossover, m //missing represents
those who were censored in period 1

died_91_18 |   died_91_180_stratacrossover
         0 |         0          1          . |     Total
-----------+---------------------------------+----------
         0 | 1,209,311          0          0 | 1,209,311
         1 |     2,373      9,986          0 |    12,359
         . |     1,685          0     22,129 |    23,814
-----------+---------------------------------+----------
     Total | 1,213,369      9,986     22,129 | 1,245,484

.
. * Died Day 181-365;
. gen died_181_365_stratacrossover = .
(1,245,484 missing values generated)

. replace died_181_365_stratacrossover = 1 if
inrange(indextodeath_caseindex, 181, 365)
(26,130 real changes made)

. replace died_181_365_stratacrossover = 0 if indextodeath_caseindex>365
(1,183,181 real changes made)

. replace died_181_365_stratacrossover = 0 if
indextodeath_caseindex>ttinfection_strata
(12,102 real changes made)

. replace died_181_365_stratacrossover = 0 if
(indextodeath_caseindex>ttstudyend) & (inrange(indextodeath_caseindex,
181, 365)) ///
>
& (inrange(ttstudyend, 181, 365))
(37 real changes made)

. tab died_181_365 died_181_365_stratacrossover, m //missing represents
those who were censored in period 1&2

died_181_3 |   died_181_365_stratacrossover
        65 |         0          1          . |     Total
-----------+---------------------------------+----------
         0 | 1,183,244          0          0 | 1,183,244
         1 |     8,018     18,049          0 |    26,067
```

```
         . |     4,058          0     32,115 |    36,173
-----------+---------------------------------+----------
     Total | 1,195,320     18,049     32,115 | 1,245,484

.
. * Died Day 366-730;
. gen died_366_730_stratacrossover = .
(1,245,484 missing values generated)

. replace died_366_730_stratacrossover = 1 if
inrange(indextodeath_caseindex, 366, 730)
(32,904 real changes made)

. replace died_366_730_stratacrossover = 0 if indextodeath_caseindex>730
(1,150,277 real changes made)

. replace died_366_730_stratacrossover = 0 if
indextodeath_caseindex>ttinfection_strata
(28,416 real changes made)

. replace died_366_730_stratacrossover = 0 if
(indextodeath_caseindex>ttstudyend) & (inrange(indextodeath_caseindex,
366, 730)) ///
>
& (inrange(ttstudyend, 366, 730))
(5,987 real changes made)

.
. tab died_366_730 died_366_730_stratacrossover, m //missing represents
those who were censored in period 1-3

died_366_7 |   died_366_730_stratacrossover
        30 |         0          1          . |     Total
-----------+---------------------------------+----------
         0 | 1,162,593          0          0 | 1,162,593
         1 |     9,985     10,603          0 |    20,588
         . |    12,102          0     50,201 |    62,303
-----------+---------------------------------+----------
     Total | 1,184,680     10,603     50,201 | 1,245,484

.
. *-------------------------------------------------------------------
----------
. * for each period, identify whether the case and at least one
comparator
. * in a matched group are alive on the first day of the period, AND
. * their strata does not have an infection crossover in the prior
period(s)
. *-------------------------------------------------------------------
----------
.
. * Alive on day 91 & no strata crossover in prior period
. gen alive_day91_stratacrossover = .
(1,245,484 missing values generated)
```

```
. replace alive_day91_stratacrossover = 1 if indextodeath_caseindex>=91
(1,221,670 real changes made)

. replace alive_day91_stratacrossover = 0 if
inrange(indextodeath_caseindex, 0, 90)
(23,814 real changes made)

. replace alive_day91_stratacrossover = 0 if inrange(ttinfection_strata,
0, 90)
(93,866 real changes made)

. replace alive_day91_stratacrossover = 0 if ttstudyend<91
(0 real changes made)

.
. sum ttinfection_strata if inrange(ttinfection_strata, 0, 90)

    Variable |        Obs        Mean    Std. dev.       Min        Max
-------------+---------------------------------------------------------
ttinfectio~a |     97,014    45.60414    22.58688          0         90

. tab alive_day91 alive_day91_stratacrossover, m

           | alive_day91_stratacro
alive_day9 |        ssover
         1 |         0          1 |     Total
-----------+----------------------+----------
         0 |    23,814          0 |    23,814
         1 |    93,866  1,127,804 | 1,221,670
-----------+----------------------+----------
     Total |   117,680  1,127,804 | 1,245,484

.
. * Alive on day 181 & no strata crossover in prior periods
. gen alive_day181_stratacrossover = .
(1,245,484 missing values generated)

. replace alive_day181_stratacrossover = 1 if
indextodeath_caseindex>=181
(1,209,311 real changes made)

. replace alive_day181_stratacrossover = 0 if
inrange(indextodeath_caseindex, 0, 180)
(36,173 real changes made)

. replace alive_day181_stratacrossover = 0 if
inrange(ttinfection_strata, 0, 180)
(158,980 real changes made)

. replace alive_day181_stratacrossover = 0 if ttstudyend<181
(0 real changes made)

.
```

```
. sum ttinfection_strata if inrange(ttinfection_strata, 0, 180)

    Variable |        Obs        Mean    Std. dev.       Min        Max
-------------+---------------------------------------------------------
ttinfectio~a |    166,097    83.04211    50.51589          0        180

. tab alive_day181 alive_day181_stratacrossover

           | alive_day181_stratacr
alive_day1 |        ossover
        81 |         0          1 |     Total
-----------+----------------------+----------
         0 |    36,173          0 |    36,173
         1 |   158,980  1,050,331 | 1,209,311
-----------+----------------------+----------
     Total |   195,153  1,050,331 | 1,245,484

.
. * Alive on day 366 & no strata crossover in prior periods
. gen alive_day366_stratacrossover = .
(1,245,484 missing values generated)

. replace alive_day366_stratacrossover = 1 if
indextodeath_caseindex>=366
(1,183,181 real changes made)

. replace alive_day366_stratacrossover = 0 if
inrange(indextodeath_caseindex, 0, 365)
(62,303 real changes made)

. replace alive_day366_stratacrossover = 0 if
inrange(ttinfection_strata, 0, 365)
(348,881 real changes made)

. replace alive_day366_stratacrossover = 0 if ttstudyend<366
(33,944 real changes made)

.
. sum ttinfection_strata if inrange(ttinfection_strata, 0, 365)

    Variable |        Obs        Mean    Std. dev.       Min        Max
-------------+---------------------------------------------------------
ttinfectio~a |    371,401    192.4092    111.3391          0        365

. tab alive_day366 alive_day366_stratacrossover

           | alive_day366_stratacr
alive_day3 |        ossover
        66 |         0          1 |     Total
-----------+----------------------+----------
         0 |   114,374          0 |   114,374
         1 |   330,754    800,356 | 1,131,110
-----------+----------------------+----------
     Total |   445,128    800,356 | 1,245,484
```

```
.
. *--------------------------------------------------------------------------------
----------
. * for each period, identify whether the case and at least one
comparator
. * in a matched group are alive on the first day of the period & were
not
. * in an infection crossover strata in the prior period
. *--------------------------------------------------------------------------------
----------
.
. foreach i of numlist 91 181 366 {
  2.
.         //cases
.         gen alive_day`i'_stratacase = 0
  3.         replace alive_day`i'_stratacase = 1 if case==1 &
alive_day`i'_stratacrossover==1
  4.         replace alive_day`i'_stratacase = 0 if case==1 &
ttstudyend<`i'
  5.         tab alive_day`i'_stratacase alive_day`i'_stratacrossover if
case==1
  6.         sum indextodeath if indextodeath<`i' & case==1
  7.
.         bysort matchgroupnumber: egen alive_day`i'_stratacase_grp =
max(alive_day`i'_stratacase)
  8.
.         //comparators
.         gen alive_day`i'_stratacontrol = 0
  9.         replace alive_day`i'_stratacontrol = 1 if case==0 &
alive_day`i'_stratacrossover==1
 10.         replace alive_day`i'_stratacontrol = 0 if case==0 &
ttstudyend<`i'
 11.
.         tab alive_day`i'_stratacontrol alive_day`i'_stratacrossover if
case==0
 12.         sum indextodeath if indextodeath<`i' & case==0
 13.
.         bysort matchgroupnumber: egen alive_day`i'_stratacontrol_num =
sum(alive_day`i'_stratacontrol)
 14.
.         //case and at least one comparator alive on day`i'
.         gen alive_day`i'_stratacasecontrol = 0
 15.         replace alive_day`i'_stratacasecontrol = 1 if
alive_day`i'_stratacase_grp==1 & alive_day`i'_stratacontrol_num>=1
 16.
.         tab alive_day`i'_stratacasecontrol case
 17.
.         //drop variables we no longer need
.         drop alive_day`i'_stratacase alive_day`i'_stratacase_grp
alive_day`i'_stratacontrol alive_day`i'_stratacontrol_num
 18.
. }
(180,575 real changes made)
```

```
(0 real changes made)

alive_day9 | alive_day91_stratacro
1_strataca |        ssover
        se |         0          1 |     Total
-----------+----------------------+----------
         0 |    27,486          0 |    27,486 
         1 |         0    180,575 |   180,575 
-----------+----------------------+----------
     Total |    27,486    180,575 |   208,061 

    Variable |        Obs        Mean    Std. dev.       Min        Max
-------------+---------------------------------------------------------
indextodea~x |     12,415    22.35747    19.79229          0         90
(947,229 real changes made)
(0 real changes made)

alive_day9 | alive_day91_stratacro
1_strataco |        ssover
     ntrol |         0          1 |     Total
-----------+----------------------+----------
         0 |    90,194          0 |    90,194 
         1 |         0    947,229 |   947,229 
-----------+----------------------+----------
     Total |    90,194    947,229 | 1,037,423 

    Variable |        Obs        Mean    Std. dev.       Min        Max
-------------+---------------------------------------------------------
indextodea~x |     11,399      44.554    25.89809          0         90
(1,080,852 real changes made)

           |     indicator: is
           |  `index_dt` the date
           | of the first positive
alive_day9 | covid-19 test result
1_strataca |          for
 secontrol |         0          1 |     Total
-----------+----------------------+----------
         0 |   137,142     27,490 |   164,632 
         1 |   900,281    180,571 | 1,080,852 
-----------+----------------------+----------
     Total | 1,037,423    208,061 | 1,245,484 
(167,998 real changes made)
(0 real changes made)

alive_day1 | alive_day181_stratacr
81_stratac |        ossover
       ase |         0          1 |     Total
-----------+----------------------+----------
         0 |    40,063          0 |    40,063 
         1 |         0    167,998 |   167,998 
-----------+----------------------+----------
     Total |    40,063    167,998 |   208,061 
```

```
    Variable |        Obs        Mean    Std. dev.       Min        Max
-------------+---------------------------------------------------------
indextodea~x |     14,459    38.05222    43.91964          0        180
(882,333 real changes made)
(0 real changes made)

alive_day1 | alive_day181_stratacr
81_stratac |        ossover
   ontrol |         0          1 |     Total
-----------+----------------------+----------
         0 |   155,090          0 |   155,090
         1 |         0    882,333 |   882,333
-----------+----------------------+----------
     Total |   155,090    882,333 | 1,037,423

    Variable |        Obs        Mean    Std. dev.       Min        Max
-------------+---------------------------------------------------------
indextodea~x |     21,714    87.84411    52.42273          0        180
(1,005,700 real changes made)

           |     indicator: is
           |  `index_dt` the date
           | of the first positive
alive_day1 | covid-19 test result
81_stratac |          for
asecontrol |         0          1 |     Total
-----------+----------------------+----------
         0 |   199,715     40,069 |   239,784
         1 |   837,708    167,992 | 1,005,700
-----------+----------------------+----------
     Total | 1,037,423    208,061 | 1,245,484
(128,351 real changes made)
(0 real changes made)

alive_day3 | alive_day366_stratacr
66_stratac |        ossover
       ase |         0          1 |     Total
-----------+----------------------+----------
         0 |    79,710          0 |    79,710
         1 |         0    128,351 |   128,351
-----------+----------------------+----------
     Total |    79,710    128,351 |   208,061

    Variable |        Obs        Mean    Std. dev.       Min        Max
-------------+---------------------------------------------------------
indextodea~x |     17,900    83.07486    103.0125          0        365
(672,005 real changes made)
(0 real changes made)

alive_day3 | alive_day366_stratacr
66_stratac |        ossover
    ontrol |         0          1 |     Total
-----------+----------------------+----------
         0 |   365,418          0 |   365,418
```

```
         1 |         0    672,005 |   672,005 
-----------+----------------------+----------
     Total |   365,418    672,005 | 1,037,423 

    Variable |        Obs        Mean    Std. dev.       Min        Max
-------------+---------------------------------------------------------
indextodea~x |     44,403       182.9    106.9272          0        365
(768,170 real changes made)

           |     indicator: is
           |  `index_dt` the date
           | of the first positive
alive_day3 | covid-19 test result
66_stratac |          for
asecontrol |         0          1 |     Total
-----------+----------------------+----------
         0 |   397,595     79,719 |   477,314 
         1 |   639,828    128,342 |   768,170 
-----------+----------------------+----------
     Total | 1,037,423    208,061 | 1,245,484 


. 
. 
. *-----------------
. * PP2 Results
. *-----------------
. 
.         /*
> 
>         Criteria
>            1)   Case and at least 1 comparator in the strata must be 
alive on the 
>                         first day of the period
> 
>            2)   Everyone in the strata who was censored due to a 
strata infection
>                         crossover in the previous period is excluded 
in the current period
> 
>            3)   People who die after the infection crossover are 
considered alive 
>                         at the time of the infection crossover
>         */
. 
. 
. 
. * Overall *
. 
. * 90-day mortality
. tab died_0_90_stratacrossover infected , co

+-------------------+
| Key               |
|-------------------|
```

```
|     frequency     |
| column percentage |
+-------------------+

died_0_90_ |
stratacros |       infected
     sover |         0          1 |     Total
-----------+----------------------+----------
         0 | 1,027,496    195,859 | 1,223,355 
           |     99.04      94.14 |     98.22 
-----------+----------------------+----------
         1 |     9,927     12,202 |    22,129 
           |      0.96       5.86 |      1.78 
-----------+----------------------+----------
     Total | 1,037,423    208,061 | 1,245,484 
           |    100.00     100.00 |    100.00 

. cs died_0_90_stratacrossover infected

                 |        infected        |
                 |   Exposed   Unexposed  |      Total
-----------------+------------------------+-----------
           Cases |     12202        9927  |      22129
        Noncases |    195859     1027496  |    1223355
-----------------+------------------------+-----------
           Total |    208061     1037423  |    1245484
                 |                        |
            Risk |  .0586463    .0095689  |   .0177674
                 |                        |
                 |      Point estimate    |    [95% conf. interval]
                 |------------------------+------------------------
 Risk difference |         .0490774       |    .0480505    .0501042 
      Risk ratio |         6.128839       |    5.971127    6.290716 
 Attr. frac. ex. |          .836837       |    .8325274    .8410356 
 Attr. frac. pop |         .4614345       |
                 +-------------------------------------------------
                               chi2(1) = 23918.44  Pr>chi2 = 0.0000

. 
. * 180-day mortality
. tab died_91_180_stratacrossover infected if
alive_day91_stratacasecontrol==1 , co

+-------------------+
| Key               |
|-------------------|
|     frequency     |
| column percentage |
+-------------------+

died_91_18 |
0_stratacr |       infected
   ossover |         0          1 |     Total
-----------+----------------------+----------
```

```
         0 |   884,062    178,780 | 1,062,842
           |     99.15      99.01 |     99.13
-----------+----------------------+----------
         1 |     7,566      1,791 |     9,357
           |      0.85       0.99 |      0.87
-----------+----------------------+----------
     Total |   891,628    180,571 | 1,072,199
           |    100.00     100.00 |    100.00

. cs died_91_180_stratacrossover infected if
alive_day91_stratacasecontrol==1

                 |        infected       |
                 |   Exposed   Unexposed |      Total
-----------------+------------------------+-----------
           Cases |      1791        7566  |       9357
        Noncases |    178780      884062  |    1062842
-----------------+------------------------+-----------
           Total |    180571      891628  |    1072199
                 |                        |
            Risk |  .0099185    .0084856  |   .0087269
                 |                        |
                 |      Point estimate    |    [95% conf. interval]
                 |------------------------+------------------------
 Risk difference |         .0014329       |    .0009378    .0019281
      Risk ratio |         1.168867       |    1.110466    1.230338
 Attr. frac. ex. |         .1444704       |    .0994775    .1872152
 Attr. frac. pop |         .0276527       |
                 +-------------------------------------------------
                               chi2(1) =    35.64  Pr>chi2 = 0.0000

.
. * 365-day mortality
. tab died_181_365_stratacrossover infected if
alive_day181_stratacasecontrol==1 , co

+-------------------+
| Key               |
|-------------------|
|     frequency     |
| column percentage |
+-------------------+

died_181_3 |
65_stratac |       infected
  rossover |         0          1 |     Total
-----------+----------------------+----------
         0 |   808,442    165,330 |   973,772
           |     98.29      98.42 |     98.31
-----------+----------------------+----------
         1 |    14,035      2,662 |    16,697
           |      1.71       1.58 |      1.69
-----------+----------------------+----------
     Total |   822,477    167,992 |   990,469
```

```
          |    100.00     100.00 |    100.00

. cs died_181_365_stratacrossover infected if
alive_day181_stratacasecontrol==1

                 |        infected        |
                 |   Exposed   Unexposed  |      Total
-----------------+------------------------+-----------
           Cases |      2662       14035  |      16697
        Noncases |    165330      808442  |     973772
-----------------+------------------------+-----------
           Total |    167992      822477  |     990469
                 |                        |
            Risk |   .015846    .0170643  |   .0168577
                 |                        |
                 |      Point estimate    |    [95% conf. interval]
                 |------------------------+------------------------
 Risk difference |       -.0012183        |   -.0018778   -.0005588
      Risk ratio |        .9286045        |    .8912122    .9675657
 Prev. frac. ex. |        .0713955        |    .0324343    .1087878
 Prev. frac. pop |        .0121093        |
                 +-------------------------------------------------
                               chi2(1) =    12.49  Pr>chi2 = 0.0004

.
. * 730-day mortality
. tab died_366_730_stratacrossover infected if
alive_day366_stratacasecontrol==1 , co

+-------------------+
| Key               |
|-------------------|
|     frequency     |
| column percentage |
+-------------------+

died_366_7 |
30_stratac |       infected
 rossover |         0          1 |     Total
-----------+----------------------+----------
         0 |   608,168    126,869 |   735,037
           |     98.73      98.85 |     98.75
-----------+----------------------+----------
         1 |     7,833      1,473 |     9,306
           |      1.27       1.15 |      1.25
-----------+----------------------+----------
     Total |   616,001    128,342 |   744,343
           |    100.00     100.00 |    100.00

. cs died_366_730_stratacrossover infected if
alive_day366_stratacasecontrol==1

                 |        infected        |
                 |   Exposed   Unexposed  |      Total
```

```
-----------------+------------------------+------------
           Cases |      1473        7833  |       9306
        Noncases |    126869      608168  |     735037
-----------------+------------------------+------------
           Total |    128342      616001  |     744343
                 |                        |
            Risk |  .0114771    .0127159  |   .0125023
                 |                        |
                 |      Point estimate    |    [95% conf. interval]
                 |------------------------+------------------------
 Risk difference |        -.0012387       |   -.0018852   -.0005923
      Risk ratio |         .9025832       |    .8539939    .9539371
 Prev. frac. ex. |         .0974168       |    .0460629    .1460061
 Prev. frac. pop |         .0167969       |
                 +-------------------------------------------------
                               chi2(1) =    13.20  Pr>chi2 = 0.0003

.
. *-----------
. * Age <65
. *-----------
.
. * 90-day mortality
. tab died_0_90_stratacrossover infected if agecat==1, co

+-------------------+
| Key               |
|-------------------|
|     frequency     |
| column percentage |
+-------------------+

died_0_90_ |
stratacros |       infected
     sover |         0          1 |     Total
-----------+----------------------+----------
         0 |   553,690    113,678 |   667,368
           |     99.72      98.82 |     99.57
-----------+----------------------+----------
         1 |     1,527      1,363 |     2,890
           |      0.28       1.18 |      0.43
-----------+----------------------+----------
     Total |   555,217    115,041 |   670,258
           |    100.00     100.00 |    100.00

. cs died_0_90_stratacrossover infected if agecat==1

                 |        infected        |
                 |   Exposed   Unexposed  |      Total
-----------------+------------------------+------------
           Cases |      1363        1527  |       2890
        Noncases |    113678      553690  |     667368
-----------------+------------------------+------------
           Total |    115041      555217  |     670258
```

```
                 |                           |
            Risk |  .0118479    .0027503    |   .0043118
                 |                           |
                 |      Point estimate       |    [95% conf. interval]
                 |---------------------------+------------------------
 Risk difference |         .0090977          |    .0084574    .0097379
      Risk ratio |         4.307913          |    4.005608    4.633033
 Attr. frac. ex. |          .767869          |      .75035    .7841587
 Attr. frac. pop |         .3621472          |
                 +-------------------------------------------------
                               chi2(1) =  1837.19  Pr>chi2 = 0.0000

.
. * 180-day mortality
. tab died_91_180_stratacrossover infected if
alive_day91_stratacasecontrol==1 & agecat==1, co

+-------------------+
| Key               |
|-------------------|
|     frequency     |
| column percentage |
+-------------------+

died_91_18 |
0_stratacr |       infected
   ossover |         0          1 |     Total
-----------+----------------------+----------
         0 |   483,658    104,745 |   588,403
           |     99.76      99.75 |     99.76
-----------+----------------------+----------
         1 |     1,143        260 |     1,403
           |      0.24       0.25 |      0.24
-----------+----------------------+----------
     Total |   484,801    105,005 |   589,806
           |    100.00     100.00 |    100.00

. cs died_91_180_stratacrossover infected if
alive_day91_stratacasecontrol==1 & agecat==1

                 |         infected          |
                 |   Exposed   Unexposed     |      Total
-----------------+---------------------------+-----------
           Cases |       260        1143     |       1403
        Noncases |    104745      483658     |     588403
-----------------+---------------------------+-----------
           Total |    105005      484801     |     589806
                 |                           |
            Risk |  .0024761    .0023577    |   .0023787
                 |                           |
                 |      Point estimate       |    [95% conf. interval]
                 |---------------------------+------------------------
 Risk difference |         .0001184          |   -.0002117    .0004486
      Risk ratio |         1.050221          |    .9180504     1.20142
```

```
 Attr. frac. ex. |         .0478193       |   -.0892648    .1676514
 Attr. frac. pop |         .0088617       |
                 +------------------------------------------------
                               chi2(1) =     0.51  Pr>chi2 = 0.4752

.
. * 365-day mortality
. tab died_181_365_stratacrossover infected if
alive_day181_stratacasecontrol==1 & agecat==1, co

+-------------------+
| Key               |
|-------------------|
|     frequency     |
| column percentage |
+-------------------+

died_181_3 |
65_stratac |       infected
 rossover |         0          1 |     Total
-----------+----------------------+----------
         0 |   447,520     97,820 |   545,340
           |     99.52      99.57 |     99.53
-----------+----------------------+----------
         1 |     2,174        418 |     2,592
           |      0.48       0.43 |      0.47
-----------+----------------------+----------
     Total |   449,694     98,238 |   547,932
           |    100.00     100.00 |    100.00

. cs died_181_365_stratacrossover infected if
alive_day181_stratacasecontrol==1 & agecat==1

                 |        infected        |
                 |   Exposed   Unexposed  |      Total
-----------------+------------------------+-----------
           Cases |       418        2174  |       2592
        Noncases |     97820      447520  |     545340
-----------------+------------------------+-----------
           Total |     98238      449694  |     547932
                 |                        |
            Risk |   .004255    .0048344  |   .0047305
                 |                        |
                 |      Point estimate    |    [95% conf. interval]
                 |------------------------+------------------------
 Risk difference |        -.0005794       |   -.0010342   -.0001247
      Risk ratio |         .8801452       |    .7928536    .9770474
 Prev. frac. ex. |         .1198548       |    .0229526    .2071464
 Prev. frac. pop |         .0214886       |
                 +------------------------------------------------
                               chi2(1) =     5.75  Pr>chi2 = 0.0165

.
. * 730-day mortality
```

```
. tab died_366_730_stratacrossover infected if
alive_day366_stratacasecontrol==1 & agecat==1, co

+-------------------+
| Key               |
|-------------------|
|     frequency     |
| column percentage |
+-------------------+

died_366_7 |
30_stratac |       infected
  rossover |         0          1 |     Total
-----------+----------------------+----------
         0 |   338,682     75,282 |   413,964
           |     99.64      99.73 |     99.65
-----------+----------------------+----------
         1 |     1,239        205 |     1,444
           |      0.36       0.27 |      0.35
-----------+----------------------+----------
     Total |   339,921     75,487 |   415,408
           |    100.00     100.00 |    100.00

. cs died_366_730_stratacrossover infected if
alive_day366_stratacasecontrol==1 & agecat==1

                 |        infected        |
                 |   Exposed   Unexposed  |      Total
-----------------+------------------------+-----------
           Cases |       205        1239  |       1444
        Noncases |     75282      338682  |     413964
-----------------+------------------------+-----------
           Total |     75487      339921  |     415408
                 |                        |
            Risk |  .0027157     .003645  |   .0034761
                 |                        |
                 |      Point estimate    |    [95% conf. interval]
                 |------------------------+-----------------------
 Risk difference |       -.0009293        |   -.0013522   -.0005063
      Risk ratio |        .7450551        |    .6428347    .8635301
 Prev. frac. ex. |        .2549449        |    .1364699    .3571653
 Prev. frac. pop |         .046328        |
                 +-----------------------------------------------
                               chi2(1) =    15.40  Pr>chi2 = 0.0001

.
. *------------
. * Age 65-85
. *------------
.
. * 90-day mortality
. tab died_0_90_stratacrossover infected if agecat==2, co

+-------------------+
```

```
| Key               |
|-------------------|
|     frequency     |
| column percentage |
+-------------------+

died_0_90_ |
stratacros |       infected
     sover |         0          1 |     Total
-----------+----------------------+----------
         0 |   425,403     74,600 |   500,003
           |     98.63      90.64 |     97.35
-----------+----------------------+----------
         1 |     5,898      7,701 |    13,599
           |      1.37       9.36 |      2.65
-----------+----------------------+----------
     Total |   431,301     82,301 |   513,602
           |    100.00     100.00 |    100.00

. cs died_0_90_stratacrossover infected if agecat==2

                 |        infected        |
                 |   Exposed   Unexposed  |      Total
-----------------+------------------------+-----------
           Cases |      7701        5898  |      13599
        Noncases |     74600      425403  |     500003
-----------------+------------------------+-----------
           Total |     82301      431301  |     513602
                 |                        |
            Risk |  .0935712    .0136749  |   .0264777
                 |                        |
                 |      Point estimate    |    [95% conf. interval]
                 |------------------------+-------------------------
 Risk difference |         .0798963       |    .0778766    .0819159
      Risk ratio |         6.842546       |     6.61987    7.072712
 Attr. frac. ex. |         .8538556       |    .8489396    .8586115
 Attr. frac. pop |         .4835313       |
                 +-------------------------------------------------
                               chi2(1) = 17115.34  Pr>chi2 = 0.0000

.
. * 180-day mortality
. tab died_91_180_stratacrossover infected if
alive_day91_stratacasecontrol==1 & agecat==2, co

+-------------------+
| Key               |
|-------------------|
|     frequency     |
| column percentage |
+-------------------+

died_91_18 |
0_stratacr |       infected
```

```
   ossover |         0          1 |     Total
-----------+----------------------+----------
         0 |   361,087     67,472 |   428,559 
           |     98.75      98.40 |     98.70 
-----------+----------------------+----------
         1 |     4,561      1,098 |     5,659 
           |      1.25       1.60 |      1.30 
-----------+----------------------+----------
     Total |   365,648     68,570 |   434,218 
           |    100.00     100.00 |    100.00 

. cs died_91_180_stratacrossover infected if
alive_day91_stratacasecontrol==1 & agecat==2

                 |        infected        |
                 |   Exposed   Unexposed  |      Total
-----------------+------------------------+-----------
           Cases |      1098        4561  |       5659
        Noncases |     67472      361087  |     428559
-----------------+------------------------+-----------
           Total |     68570      365648  |     434218
                 |                        |
            Risk |  .0160128    .0124737  |   .0130326
                 |                        |
                 |      Point estimate    |    [95% conf. interval]
                 |------------------------+------------------------
 Risk difference |         .0035391       |     .002533    .0045451 
      Risk ratio |         1.283723       |     1.20248    1.370455 
 Attr. frac. ex. |         .2210157       |    .1683855    .2703152 
 Attr. frac. pop |         .0428831       |
                 +-------------------------------------------------
                               chi2(1) =    56.23  Pr>chi2 = 0.0000

.
. * 365-day mortality
. tab died_181_365_stratacrossover infected if
alive_day181_stratacasecontrol==1 & agecat==2, co

+-------------------+
| Key               |
|-------------------|
|     frequency     |
| column percentage |
+-------------------+

died_181_3 |
65_stratac |       infected
 rossover |         0          1 |     Total
-----------+----------------------+----------
         0 |   327,676     61,926 |   389,602 
           |     97.46      97.46 |     97.46 
-----------+----------------------+----------
         1 |     8,537      1,614 |    10,151 
           |      2.54       2.54 |      2.54 
```

```
-----------+----------------------+----------
     Total |   336,213     63,540 |   399,753 
           |    100.00     100.00 |    100.00 

. cs died_181_365_stratacrossover infected if
alive_day181_stratacasecontrol==1 & agecat==2

                 |       infected         |
                 |   Exposed   Unexposed  |      Total
-----------------+------------------------+-----------
           Cases |      1614        8537  |      10151
        Noncases |     61926      327676  |     389602
-----------------+------------------------+-----------
           Total |     63540      336213  |     399753
                 |                        |
            Risk |  .0254013    .0253916  |   .0253932
                 |                        |
                 |      Point estimate    |    [95% conf. interval]
                 |------------------------+------------------------
 Risk difference |         9.68e-06       |   -.0013243    .0013436 
      Risk ratio |         1.000381       |    .9491986    1.054324 
 Attr. frac. ex. |         .0003811       |   -.0535203    .0515247 
 Attr. frac. pop |         .0000606       |
                 +-------------------------------------------------
                               chi2(1) =     0.00  Pr>chi2 = 0.9887

. 
. * 730-day mortality
. tab died_366_730_stratacrossover infected if
alive_day366_stratacasecontrol==1 & agecat==2, co

+-------------------+
| Key               |
|-------------------|
|     frequency     |
| column percentage |
+-------------------+

died_366_7 |
30_stratac |       infected
 rossover |         0          1 |     Total
-----------+----------------------+----------
         0 |   245,990     47,500 |   293,490 
           |     98.12      98.07 |     98.12 
-----------+----------------------+----------
         1 |     4,703        933 |     5,636 
           |      1.88       1.93 |      1.88 
-----------+----------------------+----------
     Total |   250,693     48,433 |   299,126 
           |    100.00     100.00 |    100.00 

. cs died_366_730_stratacrossover infected if
alive_day366_stratacasecontrol==1 & agecat==2
```

```
                 |        infected        |
                 |   Exposed   Unexposed  |      Total
-----------------+------------------------+-----------
           Cases |       933        4703  |       5636
        Noncases |     47500      245990  |     293490
-----------------+------------------------+-----------
           Total |     48433      250693  |     299126
                 |                        |
            Risk |  .0192637      .01876  |   .0188416
                 |                        |
                 |      Point estimate    |    [95% conf. interval]
                 |------------------------+------------------------
 Risk difference |         .0005037       |   -.0008306    .0018381
      Risk ratio |         1.026851       |    .9578449    1.100829
 Attr. frac. ex. |          .026149       |   -.0440104    .0915936
 Attr. frac. pop |         .0043288       |
                 +-------------------------------------------------
                               chi2(1) =     0.56  Pr>chi2 = 0.4554

.
. *------------
. * Age 85+
. *------------
.
. * 90-day mortality
. tab died_0_90_stratacrossover infected if agecat==3, co

+-------------------+
| Key               |
|-------------------|
|     frequency     |
| column percentage |
+-------------------+

died_0_90_ |
stratacros |       infected
     sover |         0          1 |     Total
-----------+----------------------+----------
         0 |    48,403      7,581 |    55,984
           |     95.08      70.72 |     90.85
-----------+----------------------+----------
         1 |     2,502      3,138 |     5,640
           |      4.92      29.28 |      9.15
-----------+----------------------+----------
     Total |    50,905     10,719 |    61,624
           |    100.00     100.00 |    100.00

. cs died_0_90_stratacrossover infected if agecat==3

                 |        infected        |
                 |   Exposed   Unexposed  |      Total
-----------------+------------------------+-----------
           Cases |      3138        2502  |       5640
        Noncases |      7581       48403  |      55984
```

```
-----------------+------------------------+------------
           Total |     10719       50905  |      61624
                 |                        |
            Risk |  .2927512    .0491504  |   .0915228
                 |                        |
                 |      Point estimate    |    [95% conf. interval]
                 |------------------------+------------------------
 Risk difference |         .2436008       |    .2347844    .2524172
      Risk ratio |         5.956235       |    5.675809    6.250515
 Attr. frac. ex. |         .8321087       |    .8238137    .8400132
 Attr. frac. pop |         .4629711       |
                 +-------------------------------------------------
                               chi2(1) =  6319.45  Pr>chi2 = 0.0000

.
. * 180-day mortality
. tab died_91_180_stratacrossover infected if
alive_day91_stratacasecontrol==1 & agecat==3, co

+-------------------+
| Key               |
|-------------------|
|     frequency     |
| column percentage |
+-------------------+

died_91_18 |
0_stratacr |       infected
   ossover |         0          1 |     Total
-----------+----------------------+----------
         0 |    39,317      6,563 |    45,880
           |     95.48      93.81 |     95.24
-----------+----------------------+----------
         1 |     1,862        433 |     2,295
           |      4.52       6.19 |      4.76
-----------+----------------------+----------
     Total |    41,179      6,996 |    48,175
           |    100.00     100.00 |    100.00

. cs died_91_180_stratacrossover infected if
alive_day91_stratacasecontrol==1 & agecat==3

                 |        infected        |
                 |   Exposed   Unexposed  |      Total
-----------------+------------------------+------------
           Cases |       433        1862  |       2295
        Noncases |      6563       39317  |      45880
-----------------+------------------------+------------
           Total |      6996       41179  |      48175
                 |                        |
            Risk |  .0618925    .0452172  |   .0476388
                 |                        |
                 |      Point estimate    |    [95% conf. interval]
                 |------------------------+------------------------
```

```
 Risk difference |         .0166753       |    .0106829    .0226677
      Risk ratio |         1.368782       |    1.236728    1.514935
 Attr. frac. ex. |         .2694234       |     .191415    .3399058
 Attr. frac. pop |         .0508324       |
                 +-------------------------------------------------
                               chi2(1) =    36.65  Pr>chi2 = 0.0000

.
. * 365-day mortality
. tab died_181_365_stratacrossover infected if
alive_day181_stratacasecontrol==1 & agecat==3, co

+-------------------+
| Key               |
|-------------------|
|     frequency     |
| column percentage |
+-------------------+

died_181_3 |
65_stratac |       infected
  rossover |         0          1 |     Total
-----------+----------------------+----------
         0 |    33,246      5,584 |    38,830
           |     90.91      89.86 |     90.76
-----------+----------------------+----------
         1 |     3,324        630 |     3,954
           |      9.09      10.14 |      9.24
-----------+----------------------+----------
     Total |    36,570      6,214 |    42,784
           |    100.00     100.00 |    100.00

. cs died_181_365_stratacrossover infected if
alive_day181_stratacasecontrol==1 & agecat==3

                 |        infected        |
                 |   Exposed   Unexposed  |      Total
-----------------+------------------------+------------
           Cases |       630        3324  |       3954
        Noncases |      5584       33246  |      38830
-----------------+------------------------+------------
           Total |      6214       36570  |      42784
                 |                        |
            Risk |   .101384    .0908942  |   .0924177
                 |                        |
                 |      Point estimate    |    [95% conf. interval]
                 |------------------------+------------------------
 Risk difference |         .0104898       |    .0024275    .0185521
      Risk ratio |         1.115407       |    1.028818    1.209283
 Attr. frac. ex. |          .103466       |    .0280109    .1730636
 Attr. frac. pop |         .0164855       |
                 +-------------------------------------------------
                               chi2(1) =     6.97  Pr>chi2 = 0.0083
```

```
.
. * 730-day mortality
. tab died_366_730_stratacrossover infected if
alive_day366_stratacasecontrol==1 & agecat==3, co

+-------------------+
| Key               |
|-------------------|
|     frequency     |
| column percentage |
+-------------------+

died_366_7 |
30_stratac |       infected
 rossover |         0          1 |     Total
-----------+----------------------+----------
         0 |    23,496      4,087 |    27,583
           |     92.55      92.42 |     92.53
-----------+----------------------+----------
         1 |     1,891        335 |     2,226
           |      7.45       7.58 |      7.47
-----------+----------------------+----------
     Total |    25,387      4,422 |    29,809
           |    100.00     100.00 |    100.00

. cs died_366_730_stratacrossover infected if
alive_day366_stratacasecontrol==1 & agecat==3

                 |        infected        |
                 |   Exposed   Unexposed  |      Total
-----------------+------------------------+-----------
           Cases |       335        1891  |       2226
        Noncases |      4087       23496  |      27583
-----------------+------------------------+-----------
           Total |      4422       25387  |      29809
                 |                        |
            Risk |  .0757576    .0744869  |   .0746754
                 |                        |
                 |      Point estimate    |    [95% conf. interval]
                 |------------------------+------------------------
 Risk difference |         .0012706       |   -.0071708    .0097121
      Risk ratio |         1.017058       |    .9095618     1.13726
 Attr. frac. ex. |         .0167724       |   -.0994306    .1206934
 Attr. frac. pop |         .0025241       |
                 +-------------------------------------------------
                               chi2(1) =     0.09  Pr>chi2 = 0.7667

.
. *-----------
. * Wave 1
. *-----------
.
. * 90-day mortality
. tab died_0_90_stratacrossover infected if wave==1, co
```

```
+-------------------+
| Key               |
|-------------------|
|     frequency     |
| column percentage |
+-------------------+

died_0_90_ |
stratacros |       infected
     sover |         0          1 |     Total
-----------+----------------------+----------
         0 |    88,652     16,271 |   104,923 
           |     98.63      90.40 |     97.26 
-----------+----------------------+----------
         1 |     1,231      1,728 |     2,959 
           |      1.37       9.60 |      2.74 
-----------+----------------------+----------
     Total |    89,883     17,999 |   107,882 
           |    100.00     100.00 |    100.00 

. cs died_0_90_stratacrossover infected if wave==1

                 |        infected         |
                 |   Exposed   Unexposed   |      Total
-----------------+-------------------------+------------
           Cases |      1728        1231   |       2959
        Noncases |     16271       88652   |     104923
-----------------+-------------------------+------------
           Total |     17999       89883   |     107882
                 |                         |
            Risk |  .0960053    .0136956   |   .0274281
                 |                         |
                 |      Point estimate     |    [95% conf. interval]
                 |-------------------------+------------------------
 Risk difference |         .0823098        |    .0779394    .0866801 
      Risk ratio |         7.009949        |    6.527367    7.528209 
 Attr. frac. ex. |         .8573456        |    .8467989    .8671663 
 Attr. frac. pop |         .5006736        |
                 +--------------------------------------------------
                               chi2(1) =  3808.57  Pr>chi2 = 0.0000

.
. * 180-day mortality
. tab died_91_180_stratacrossover infected if
alive_day91_stratacasecontrol==1 & wave==1, co

+-------------------+
| Key               |
|-------------------|
|     frequency     |
| column percentage |
+-------------------+
```

```
died_91_18 |
0_stratacr |       infected
   ossover |         0          1 |     Total
-----------+----------------------+----------
         0 |    74,892     15,120 |    90,012
           |     98.82      98.32 |     98.73
-----------+----------------------+----------
         1 |       897        259 |     1,156
           |      1.18       1.68 |      1.27
-----------+----------------------+----------
     Total |    75,789     15,379 |    91,168
           |    100.00     100.00 |    100.00

. cs died_91_180_stratacrossover infected if
alive_day91_stratacasecontrol==1 & wave==1

                 |        infected        |
                 |   Exposed   Unexposed  |      Total
-----------------+------------------------+-----------
           Cases |       259         897  |       1156
        Noncases |     15120       74892  |      90012
-----------------+------------------------+-----------
           Total |     15379       75789  |      91168
                 |                        |
            Risk |  .0168411    .0118355  |   .0126799
                 |                        |
                 |      Point estimate    |    [95% conf. interval]
                 |------------------------+------------------------
 Risk difference |         .0050057       |    .0028311    .0071802
      Risk ratio |         1.422936       |    1.240554    1.632131
 Attr. frac. ex. |         .2972278       |    .1939088    .3873041
 Attr. frac. pop |         .0665934       |
                 +-------------------------------------------------
                               chi2(1) =    25.59  Pr>chi2 = 0.0000

.
. * 365-day mortality
. tab died_181_365_stratacrossover infected if
alive_day181_stratacasecontrol==1 & wave==1, co

+-------------------+
| Key               |
|-------------------|
|     frequency     |
| column percentage |
+-------------------+

died_181_3 |
65_stratac |       infected
  rossover |         0          1 |     Total
-----------+----------------------+----------
         0 |    66,998     13,740 |    80,738
           |     97.90      97.77 |     97.88
-----------+----------------------+----------
```

```
         1 |     1,437        314 |     1,751 
           |      2.10       2.23 |      2.12 
-----------+----------------------+----------
     Total |    68,435     14,054 |    82,489 
           |    100.00     100.00 |    100.00 

. cs died_181_365_stratacrossover infected if
alive_day181_stratacasecontrol==1 & wave==1

                 |        infected        |
                 |   Exposed   Unexposed  |      Total
-----------------+------------------------+-----------
           Cases |       314        1437  |       1751
        Noncases |     13740       66998  |      80738
-----------------+------------------------+-----------
           Total |     14054       68435  |      82489
                 |                        |
            Risk |  .0223424     .020998  |   .0212271
                 |                        |
                 |      Point estimate    |    [95% conf. interval]
                 |------------------------+------------------------
 Risk difference |         .0013444       |   -.0013248    .0040135
      Risk ratio |         1.064023       |    .9430077    1.200569
 Attr. frac. ex. |         .0601711       |   -.0604367    .1670617
 Attr. frac. pop |         .0107902       |
                 +-------------------------------------------------
                               chi2(1) =     1.01  Pr>chi2 = 0.3139


.
. * 730-day mortality
. tab died_366_730_stratacrossover infected if
alive_day366_stratacasecontrol==1 & wave==1, co

+-------------------+
| Key               |
|-------------------|
|     frequency     |
| column percentage |
+-------------------+

died_366_7 |
30_stratac |       infected
 rossover |          0          1 |     Total
-----------+----------------------+----------
         0 |    50,774     10,658 |    61,432 
           |     96.89      96.69 |     96.85 
-----------+----------------------+----------
         1 |     1,630        365 |     1,995 
           |      3.11       3.31 |      3.15 
-----------+----------------------+----------
     Total |    52,404     11,023 |    63,427 
           |    100.00     100.00 |    100.00 

. cs died_366_730_stratacrossover infected if
```

```
alive_day366_stratacasecontrol==1 & wave==1

                 |        infected         |
                 |   Exposed   Unexposed   |      Total
-----------------+-------------------------+-----------
           Cases |       365        1630   |       1995
        Noncases |     10658       50774   |      61432
-----------------+-------------------------+-----------
           Total |     11023       52404   |      63427
                 |                         |
            Risk |  .0331126    .0311045   |   .0314535
                 |                         |
                 |      Point estimate     |    [95% conf. interval]
                 |-------------------------+------------------------
 Risk difference |         .0020081        |    -.001648    .0056641
      Risk ratio |         1.064559        |     .952123    1.190273
 Attr. frac. ex. |         .0606442        |   -.0502844    .1598568
 Attr. frac. pop |         .0110953        |
                 +--------------------------------------------------
                               chi2(1) =     1.21  Pr>chi2 = 0.2722

.
. *-----------
. * Wave 2
. *-----------
.
. * 90-day mortality
. tab died_0_90_stratacrossover infected if wave==2, co

+-------------------+
| Key               |
|-------------------|
|     frequency     |
| column percentage |
+-------------------+

died_0_90_ |
stratacros |       infected
     sover |         0          1 |     Total
-----------+----------------------+----------
         0 |   208,921     39,805 |   248,726
           |     99.09      94.22 |     98.27
-----------+----------------------+----------
         1 |     1,925      2,441 |     4,366
           |      0.91       5.78 |      1.73
-----------+----------------------+----------
     Total |   210,846     42,246 |   253,092
           |    100.00     100.00 |    100.00

. cs died_0_90_stratacrossover infected if wave==2

                 |        infected         |
                 |   Exposed   Unexposed   |      Total
-----------------+-------------------------+-----------
```

```
           Cases |      2441        1925  |       4366
        Noncases |     39805      208921  |     248726
-----------------+------------------------+-----------
           Total |     42246      210846  |     253092
                 |                        |
            Risk |  .0577806    .0091299  |   .0172506
                 |                        |
                 |      Point estimate    |    [95% conf. interval]
                 |------------------------+------------------------
 Risk difference |         .0486507       |     .046389    .0509124
      Risk ratio |         6.328734       |    5.967197    6.712175
 Attr. frac. ex. |         .8419905       |    .8324171     .851017
 Attr. frac. pop |          .470751       |
                 +-------------------------------------------------
                               chi2(1) =  4913.64  Pr>chi2 = 0.0000

.
. * 180-day mortality
. tab died_91_180_stratacrossover infected if
alive_day91_stratacasecontrol==1 & wave==2, co

+-------------------+
| Key               |
|-------------------|
|     frequency     |
| column percentage |
+-------------------+

died_91_18 |
0_stratacr |       infected
   ossover |         0          1 |     Total
-----------+----------------------+----------
         0 |   177,688     35,869 |   213,557
           |     99.16      98.97 |     99.13
-----------+----------------------+----------
         1 |     1,506        373 |     1,879
           |      0.84       1.03 |      0.87
-----------+----------------------+----------
     Total |   179,194     36,242 |   215,436
           |    100.00     100.00 |    100.00

. cs died_91_180_stratacrossover infected if
alive_day91_stratacasecontrol==1 & wave==2

                 |        infected        |
                 |   Exposed   Unexposed  |      Total
-----------------+------------------------+-----------
           Cases |       373        1506  |       1879
        Noncases |     35869      177688  |     213557
-----------------+------------------------+-----------
           Total |     36242      179194  |     215436
                 |                        |
            Risk |  .0102919    .0084043  |   .0087218
                 |                        |
```

```
                  |      Point estimate    |    [95% conf. interval]
                  |------------------------+------------------------
 Risk difference |         .0018876       |    .0007659    .0030094
      Risk ratio |         1.224603       |    1.093982     1.37082
 Attr. frac. ex. |         .1834085       |    .0859078    .2705095
 Attr. frac. pop |         .0364084       |
                  +-------------------------------------------------
                               chi2(1) =    12.42  Pr>chi2 = 0.0004

.
. * 365-day mortality
. tab died_181_365_stratacrossover infected if
alive_day181_stratacasecontrol==1 & wave==2, co

+-------------------+
| Key               |
|-------------------|
|     frequency     |
| column percentage |
+-------------------+

died_181_3 |
65_stratac |       infected
 rossover |         0          1 |     Total
-----------+----------------------+----------
         0 |   151,753     30,995 |   182,748
           |     98.44      98.49 |     98.45
-----------+----------------------+----------
         1 |     2,407        474 |     2,881
           |      1.56       1.51 |      1.55
-----------+----------------------+----------
     Total |   154,160     31,469 |   185,629
           |    100.00     100.00 |    100.00

. cs died_181_365_stratacrossover infected if
alive_day181_stratacasecontrol==1 & wave==2

                  |        infected        |
                  |   Exposed   Unexposed  |      Total
-----------------+------------------------+-----------
            Cases |       474        2407  |       2881
        Noncases |     30995      151753  |     182748
-----------------+------------------------+-----------
            Total |     31469      154160  |     185629
                  |                        |
             Risk |  .0150624    .0156136  |   .0155202
                  |                        |
                  |      Point estimate    |    [95% conf. interval]
                  |------------------------+------------------------
 Risk difference |        -.0005512       |   -.0020324      .00093
      Risk ratio |         .9646972       |    .8748682     1.06375
 Prev. frac. ex. |         .0353028       |   -.0637496    .1251318
 Prev. frac. pop |         .0059848       |
                  +-------------------------------------------------
```

```
                                 chi2(1) =     0.52  Pr>chi2 = 0.4710

.
. * 730-day mortality
. tab died_366_730_stratacrossover infected if
alive_day366_stratacasecontrol==1 & wave==2, co

+-------------------+
| Key               |
|-------------------|
|     frequency     |
| column percentage |
+-------------------+

died_366_7 |
30_stratac |       infected
 rossover |         0          1 |     Total
-----------+----------------------+----------
         0 |   130,557     27,143 |   157,700
           |     98.21      98.33 |     98.23
-----------+----------------------+----------
         1 |     2,386        461 |     2,847
           |      1.79       1.67 |      1.77
-----------+----------------------+----------
     Total |   132,943     27,604 |   160,547
           |    100.00     100.00 |    100.00

. cs died_366_730_stratacrossover infected if
alive_day366_stratacasecontrol==1 & wave==2

                 |        infected        |
                 |   Exposed   Unexposed  |      Total
-----------------+------------------------+-----------
           Cases |       461        2386  |       2847
        Noncases |     27143      130557  |     157700
-----------------+------------------------+-----------
           Total |     27604      132943  |     160547
                 |                        |
            Risk |  .0167005    .0179475  |   .0177331
                 |                        |
                 |      Point estimate    |    [95% conf. interval]
                 |------------------------+------------------------
 Risk difference |        -.0012471       |   -.0029188    .0004246
      Risk ratio |         .9305162       |    .8429198    1.027216
 Prev. frac. ex. |         .0694838       |   -.0272157    .1570802
 Prev. frac. pop |         .0119468       |
                 +-------------------------------------------------
                                 chi2(1) =     2.04  Pr>chi2 = 0.1531

.
. *-----------
. * Wave 3
. *-----------
.
```

```
. * 90-day mortality
. tab died_0_90_stratacrossover infected if wave==3, co

+-------------------+
| Key               |
|-------------------|
|     frequency     |
| column percentage |
+-------------------+

died_0_90_ |
stratacros |       infected
     sover |         0          1 |     Total
-----------+----------------------+----------
         0 |   624,665    119,481 |   744,146 
           |     99.06      94.45 |     98.29 
-----------+----------------------+----------
         1 |     5,956      7,023 |    12,979 
           |      0.94       5.55 |      1.71 
-----------+----------------------+----------
     Total |   630,621    126,504 |   757,125 
           |    100.00     100.00 |    100.00 

. cs died_0_90_stratacrossover infected if wave==3

                 |        infected        |
                 |   Exposed   Unexposed  |      Total
-----------------+------------------------+-----------
           Cases |      7023        5956  |      12979
        Noncases |    119481      624665  |     744146
-----------------+------------------------+-----------
           Total |    126504      630621  |     757125
                 |                        |
            Risk |   .055516    .0094447  |   .0171425
                 |                        |
                 |      Point estimate    |    [95% conf. interval]
                 |------------------------+------------------------
 Risk difference |         .0460714       |    .0447872    .0473556 
      Risk ratio |         5.878035       |    5.681583    6.081279 
 Attr. frac. ex. |         .8298751       |    .8239927    .8355609 
 Attr. frac. pop |         .4490495       |
                 +-------------------------------------------------
                               chi2(1) = 13274.04  Pr>chi2 = 0.0000

.
. * 180-day mortality
. tab died_91_180_stratacrossover infected if
alive_day91_stratacasecontrol==1 & wave==3, co

+-------------------+
| Key               |
|-------------------|
|     frequency     |
| column percentage |
```

```
+-------------------+

died_91_18 |
0_stratacr |       infected
   ossover |         0          1 |     Total
-----------+----------------------+----------
         0 |   534,978    108,276 |   643,254 
           |     99.18      99.10 |     99.16 
-----------+----------------------+----------
         1 |     4,439        986 |     5,425 
           |      0.82       0.90 |      0.84 
-----------+----------------------+----------
     Total |   539,417    109,262 |   648,679 
           |    100.00     100.00 |    100.00 

. cs died_91_180_stratacrossover infected if
alive_day91_stratacasecontrol==1 & wave==3

                 |        infected        |
                 |   Exposed   Unexposed  |      Total
-----------------+------------------------+-----------
           Cases |       986        4439  |       5425
        Noncases |    108276      534978  |     643254
-----------------+------------------------+-----------
           Total |    109262      539417  |     648679
                 |                        |
            Risk |  .0090242    .0082293  |   .0083632
                 |                        |
                 |      Point estimate    |    [95% conf. interval]
                 |------------------------+-----------------------
 Risk difference |         .0007949       |    .0001846    .0014053 
      Risk ratio |         1.096598       |    1.023795    1.174577 
 Attr. frac. ex. |         .0880884       |    .0232421    .1486296 
 Attr. frac. pop |         .0160102       |
                 +------------------------------------------------
                               chi2(1) =     6.92  Pr>chi2 = 0.0085

.
. * 365-day mortality
. tab died_181_365_stratacrossover infected if
alive_day181_stratacasecontrol==1 & wave==3, co

+-------------------+
| Key               |
|-------------------|
|     frequency     |
| column percentage |
+-------------------+

died_181_3 |
65_stratac |       infected
  rossover |         0          1 |     Total
-----------+----------------------+----------
         0 |   502,902    102,864 |   605,766 
```

```
           |     98.24      98.41 |     98.27 
-----------+----------------------+----------
         1 |     9,011      1,657 |    10,668 
           |      1.76       1.59 |      1.73 
-----------+----------------------+----------
     Total |   511,913    104,521 |   616,434 
           |    100.00     100.00 |    100.00 

. cs died_181_365_stratacrossover infected if
alive_day181_stratacasecontrol==1 & wave==3

                 |        infected        |
                 |   Exposed   Unexposed  |      Total
-----------------+------------------------+-----------
           Cases |      1657        9011  |      10668
        Noncases |    102864      502902  |     605766
-----------------+------------------------+-----------
           Total |    104521      511913  |     616434
                 |                        |
            Risk |  .0158533    .0176026  |    .017306
                 |                        |
                 |      Point estimate    |    [95% conf. interval]
                 |------------------------+------------------------
 Risk difference |        -.0017493       |   -.0025879   -.0009108
      Risk ratio |         .9006211       |    .8550155    .9486593
 Prev. frac. ex. |         .0993789       |    .0513407    .1449845
 Prev. frac. pop |         .0168504       |
                 +-------------------------------------------------
                               chi2(1) =    15.62  Pr>chi2 = 0.0001

.
. * 730-day mortality
. tab died_366_730_stratacrossover infected if
alive_day366_stratacasecontrol==1 & wave==3, co

+-------------------+
| Key               |
|-------------------|
|     frequency     |
| column percentage |
+-------------------+

died_366_7 |
30_stratac |       infected
 rossover |         0          1 |     Total
-----------+----------------------+----------
         0 |   391,408     81,685 |   473,093 
           |     99.10      99.26 |     99.13 
-----------+----------------------+----------
         1 |     3,561        613 |     4,174 
           |      0.90       0.74 |      0.87 
-----------+----------------------+----------
     Total |   394,969     82,298 |   477,267 
           |    100.00     100.00 |    100.00 
```

```
. cs died_366_730_stratacrossover infected if
alive_day366_stratacasecontrol==1 & wave==3

                 |        infected         |
                 |   Exposed   Unexposed   |      Total
-----------------+------------------------+-----------
           Cases |       613        3561   |       4174
        Noncases |     81685      391408   |     473093
-----------------+------------------------+-----------
           Total |     82298      394969   |     477267
                 |                         |
            Risk |  .0074485    .0090159   |   .0087456
                 |                         |
                 |      Point estimate     |    [95% conf. interval]
                 |------------------------+------------------------
 Risk difference |        -.0015674        |   -.0022246   -.0009101
      Risk ratio |         .8261563        |    .7585497    .8997885
 Prev. frac. ex. |         .1738437        |    .1002115    .2414503
 Prev. frac. pop |         .0299769        |
                 +-------------------------------------------------
                               chi2(1) =    19.30  Pr>chi2 = 0.0000

.
. *------------------------------------------------------------------
. * PP2 Constructing TTE & Mortality Variables for Cox Model
. *------------------------------------------------------------------
.
. * censor matched strata at the time of an infection crossover;
. * patients in a crossover strata who die prior to the infection
crossover
. * will have their TTE recorded as their individual date of death
.
. * censor everyone at the earliest censoring time of anyone in the
matched
. * strata, using earliest time of death, study end, or crossover
. gsort matchgroupnumber propensityscoreabsdiffrank -case

.
. * create a new tte variable from the crossover tte variable, censoring
everyone
. * in the strata at the time of the earliest infection crossover
. by matchgroupnumber: egen mkg_tte_730_strata = min(tte_730_cross) if
isfutureinfectedcontrol==1
(1,108,158 missing values generated)

. by matchgroupnumber: egen tte_730_strata = min(mkg_tte_730_strata)
(630,819 missing values generated)

.
. * if someone dies prior to the time of the earliest censoring date for
the strata,
. * recode THAT individual with the date of their death
. replace tte_730_strata = tte_730_cross if indextodeath_caseindex
```

```
!missing(tte_730_strata) & ///
>
indextodeath_caseindex
indextodeath_caseindex<730
(23,020 real changes made)

.
. * any strata not censored at crossover has a tte as done previously
(at time
. * of study end) or at time of death
. replace tte_730_strata = tte_730_cross if tte_730_strata == .
(630,819 real changes made)

.
. * code the mortality variable from the crossover version above
. gen died_730_strata = died_730_cross

.
. * modify mortality variable to account for censoring at infection
crossover
. replace died_730_strata = 0 if indextodeath_caseindex>tte_730_strata &
!missing(indextodeath_caseindex)
(11,552 real changes made)

.
. * drop variables we no longer need
. drop mkg_tte_730_strata

.
. * PP2 - mortality and excess mortality among cases
. tab died_730_strata infected, co chi // (Abstract & Results)

+-------------------+
| Key               |
|-------------------|
|     frequency     |
| column percentage |
+-------------------+

died_730_s |       infected
     trata |         0          1 |     Total
-----------+----------------------+----------
         0 |   995,024    189,933 | 1,184,957
           |     95.91      91.29 |     95.14
-----------+----------------------+----------
         1 |    42,399     18,128 |    60,527
           |      4.09       8.71 |      4.86
-----------+----------------------+----------
     Total | 1,037,423    208,061 | 1,245,484
           |    100.00     100.00 |    100.00

          Pearson chi2(1) =  8.0e+03   Pr = 0.000

.
```

```
. di 42399/1037423 //4.09 mortality rate of comparators (Abstract &
Results)
.04086954

. di 208061*0.04086954 //mortality rate of comparators applied to cases
= 8503 deaths
8503.3574

. di 18128-8503.3574 //9625 excess deaths
9624.6426

.
. *----------------------------
. * PP2 Kaplan Meier Curves
. *----------------------------
.
. ** Creating Variables for KM **
.
. * alive on index day, censored at 90 days
. gen tte_0_90_strata = ttstudyend

. replace tte_0_90_strata = 90 if ttstudyend>90
(1,245,484 real changes made)

. replace tte_0_90_strata = indextodeath_caseindex  ///
>                                        if indextodeath_caseindex
indextodeath_caseindex<90
(23,659 real changes made)

. replace tte_0_90_strata = tte_730_strata if tte_730_strata<=90
(94,745 real changes made)

.
. * alive on Day 91, censored at 180 days
. gen tte_91_180_strata = ttstudyend

. replace tte_91_180_strata = 180 if ttstudyend>180
(1,245,484 real changes made)

. replace tte_91_180_strata = indextodeath_caseindex if   ///
>                                                indextodeath_caseindex
inrange(indextodeath_caseindex, 91, 180)
(12,195 real changes made)

. replace tte_91_180_strata = tte_730_strata if inrange(tte_730_strata,
91, 180)
(66,788 real changes made)

. replace tte_91_180_strata = . if alive_day91==0
(23,814 real changes made, 23,814 to missing)

.
. * alive on Day 181, censored at 365 days
. gen tte_181_365_strata = ttstudyend
```

```
. replace tte_181_365_strata = 365 if ttstudyend>365
(1,191,419 real changes made)

. replace tte_181_365_strata = indextodeath_caseindex if  ///
>                                                   indextodeath_caseindex
inrange(indextodeath_caseindex, 181, 365)
(25,921 real changes made)

. replace tte_181_365_strata = tte_730_strata if inrange(tte_730_strata,
181, 365)
(196,086 real changes made)

. replace tte_181_365_strata = . if alive_day181==0
(36,173 real changes made, 36,173 to missing)

.
. * alive on Day 366, censored at 730 days
. gen tte_366_730_strata = ttstudyend

. replace tte_366_730_strata = 730 if ttstudyend>730
(13,998 real changes made)

. replace tte_366_730_strata = indextodeath_caseindex if  ///
>                                                   indextodeath_caseindex
inrange(indextodeath_caseindex, 366, 730)
(20,049 real changes made)

. replace tte_366_730_strata = tte_730_strata if inrange(tte_730_strata,
366, 730)
(164,239 real changes made)

. replace tte_366_730_strata = . if alive_day366==0
(114,374 real changes made, 114,374 to missing)

.
.
. ** Figure 1: KM Curves **
.
. * 0-90 Days
. preserve

.         recode tte_0_90_strata (0=0.5)
(377 changes made to tte_0_90_strata)

.         stset tte_0_90_strata , failure(died_0_90_stratacrossover==1)

Survival-time data settings

         Failure event: died_0_90_stratacrossover==1
Observed time interval: (0, tte_0_90_strata]
     Exit on or before: failure

----------------------------------------------------------------------------
```

```
--
  1,245,484  total observations
          0  exclusions
------------------------------------------------------------------------
--
  1,245,484  observations remaining, representing
     22,129  failures in single-record/single-failure data
  106542617  total analysis time at risk and under observation
                                                At risk from t =
0
                                     Earliest observed entry t =
0
                                          Last observed exit t =
90

.         *sts test infected, logrank
.         sts graph, by(infected) name(survival090, replace)      ///
>                 legend(pos(5) ring(0) rows(2) region(lcolor(none)) ///
>                               size(small) label(1 "Comparators")
label(2 "COVID-19 Cases"))    ///
>                 graphregion(color(white)) bgcolor(white) title("Days
0-90", color(black) size(medium)) ///
>                 xtitle("") ///
>                 plot1opts(lpattern(solid) lcolor("0 114 178")) ///
>                 plot2opts(lpattern(dash) lcolor("230 159 0"))  ///
>                 tlabel(, labsize(small)) ylabel(, labsize(small))

        Failure _d: died_0_90_stratacrossover==1
  Analysis time _t: tte_0_90_strata

.         *graph save "survival090" "Figures\survival090.gph", replace
. restore

.
. * 91-180 Days
. preserve

.         stset tte_91_180_strata ,
failure(died_91_180_stratacrossover==1)

Survival-time data settings

         Failure event: died_91_180_stratacrossover==1
Observed time interval: (0, tte_91_180_strata]
     Exit on or before: failure

------------------------------------------------------------------------
--
  1,245,484  total observations
     23,814  event time missing (tte_91_180_strata>=.)      PROBABLE
ERROR
------------------------------------------------------------------------
--
  1,221,670  observations remaining, representing
```

```
      9,986  failures in single-record/single-failure data
  216380896  total analysis time at risk and under observation
                                                At risk from t =
0
                                     Earliest observed entry t =
0
                                          Last observed exit t =
180

.         *sts test infected, logrank
.         sts graph, by(infected) name(survival91180, replace)  ///
>                 legend(pos(5) ring(0) rows(2) region(lcolor(none)) ///
>                                 size(small) label(1 "Comparators")
label(2 "COVID-19 Cases"))    ///
>                 graphregion(color(white)) bgcolor(white) title("Days
91-180", color(black) size(medium)) ///
>                 xtitle("") ///
>                 plot1opts(lpattern(solid) lcolor("0 114 178")) ///
>                 plot2opts(lpattern(dash) lcolor("230 159 0"))   ///
>                 ylabel(0.9 (0.02) 1.0, labsize(small)) ///
>                 tmin(91) tlabel(90 (20) 190, labsize(small))

        Failure _d: died_91_180_stratacrossover==1
  Analysis time _t: tte_91_180_strata

.         *graph save "survival91180" "Figures\survival91180.gph",
replace
. restore

.
. * 181-365 Days
. preserve

.         stset tte_181_365_strata ,
failure(died_181_365_stratacrossover==1)

Survival-time data settings

         Failure event: died_181_365_stratacrossover==1
Observed time interval: (0, tte_181_365_strata]
     Exit on or before: failure

------------------------------------------------------------------------
--
  1,245,484  total observations
     36,173  event time missing (tte_181_365_strata>=.)     PROBABLE
ERROR
------------------------------------------------------------------------
--
  1,209,311  observations remaining, representing
     18,049  failures in single-record/single-failure data
  422019742  total analysis time at risk and under observation
                                                At risk from t =
0
```

```
                                    Earliest observed entry t =
0
                                         Last observed exit t =
365

.         *sts test infected, logrank
.         tab _d infected, co chi // (Results: 1.37% (1.4) vs 1.51%
(1.5))

+-------------------+
| Key               |
|-------------------|
|     frequency     |
| column percentage |
+-------------------+

      1 if |
failure; 0 |
        if |       infected
  censored |         0          1 |     Total
-----------+----------------------+----------
         0 | 1,000,322    190,940 | 1,191,262
           |     98.49      98.63 |     98.51
-----------+----------------------+----------
         1 |    15,387      2,662 |    18,049
           |      1.51       1.37 |      1.49
-----------+----------------------+----------
     Total | 1,015,709    193,602 | 1,209,311
           |    100.00     100.00 |    100.00

          Pearson chi2(1) =  21.6519   Pr = 0.000

.         sts graph, by(infected) name(survival181365, replace) ///
>                 legend(pos(5) ring(0) rows(2) region(lcolor(none)) ///
>                                 size(small) label(1 "Comparators")
label(2 "COVID-19 Cases"))    ///
>                 graphregion(color(white)) bgcolor(white) title("Days
181-365", color(black) size(medium)) ///
>                 xtitle("") ///
>                 plot1opts(lpattern(solid) lcolor("0 114 178")) ///
>                 plot2opts(lpattern(dash) lcolor("230 159 0"))   ///
>                 ylabel(0.9 (0.02) 1.0, labsize(small)) ///
>                 tmin(181) tlabel(180 (40) 380, labsize(small))

        Failure _d: died_181_365_stratacrossover==1
  Analysis time _t: tte_181_365_strata

.         *graph save "survival181365" "Figures\survival181365.gph",
replace
. restore

.
. * 366-730 Days
. preserve
```

```
.         stset tte_366_730_strata ,
failure(died_366_730_stratacrossover==1)

Survival-time data settings

         Failure event: died_366_730_stratacrossover==1
Observed time interval: (0, tte_366_730_strata]
     Exit on or before: failure

-------------------------------------------------------------------------
--
  1,245,484  total observations
    114,374  event time missing (tte_366_730_strata>=.)     PROBABLE
ERROR
-------------------------------------------------------------------------
--
  1,131,110  observations remaining, representing
     10,364  failures in single-record/single-failure data
  555436295  total analysis time at risk and under observation
                                                At risk from t =
0
                                     Earliest observed entry t =
0
                                          Last observed exit t =
730

.         *sts test infected, logrank
.         tab _d infected, co chi // (Results: 0.81% (0.8) vs 0.94%
(0.9))

+-------------------+
| Key               |
|-------------------|
|     frequency     |
| column percentage |
+-------------------+

      1 if |
failure; 0 |
        if |       infected
  censored |         0          1 |     Total
-----------+----------------------+----------
         0 |   940,563    180,183 | 1,120,746
           |     99.06      99.19 |     99.08
-----------+----------------------+----------
         1 |     8,890      1,474 |    10,364
           |      0.94       0.81 |      0.92
-----------+----------------------+----------
     Total |   949,453    181,657 | 1,131,110
           |    100.00     100.00 |    100.00

          Pearson chi2(1) =  26.2051   Pr = 0.000
```

```
.         sts graph, by(infected) name(survival366730, replace)  ///
>                 legend(pos(5) ring(0) rows(2) region(lcolor(none)) ///
>                                size(small) label(1 "Comparators")
label(2 "COVID-19 Cases"))    ///
>                 graphregion(color(white)) bgcolor(white) title("Days
366-730", color(black) size(medium)) ///
>                 xtitle("") ///
>                 plot1opts(lpattern(solid) lcolor("0 114 178")) ///
>                 plot2opts(lpattern(dash) lcolor("230 159 0"))   ///
>                 ylabel(0.9 (0.02) 1.0, labsize(small)) ///
>                 tmin(366) tlabel(365 450 550 650 730, labsize(small))

        Failure _d: died_366_730_stratacrossover==1
  Analysis time _t: tte_366_730_strata

.         *graph save "survival366730" "Figures\survival366730.gph",
replace
. restore


.
. * 0-730 Days
. preserve

.         recode tte_730_strata (0=0.5)
(377 changes made to tte_730_strata)

.         stset tte_730_strata , failure(died_730_strata)

Survival-time data settings

         Failure event: died_730_strata!=0 & died_730_strata<.
Observed time interval: (0, tte_730_strata]
     Exit on or before: failure

------------------------------------------------------------------------
--
  1,245,484  total observations
          0  exclusions
------------------------------------------------------------------------
--
  1,245,484  observations remaining, representing
     60,527  failures in single-record/single-failure data
  477586587  total analysis time at risk and under observation
                                                    At risk from t =
0
                                         Earliest observed entry t =
0
                                              Last observed exit t =
730

.         stdescribe

        Failure _d: died_730_strata
  Analysis time _t: tte_730_strata
```

```
                                 |-------------- Per subject ---------
-----|
Category                   Total        Mean         Min     Median
Max
----------------------------------------------------------------------
------
Number of subjects       1245484
Number of records        1245484           1           1          1
1

Entry time (first)                         0           0          0
0
Exit time (final)                   383.4546          .5        423
730

Subjects with gap              0
Time on gap                    0
Time at risk           4.776e+08    383.4546          .5        423
730

Failures                   60527    .0485972           0          0
1
----------------------------------------------------------------------
------

.         stdescribe if infected==1

        Failure _d: died_730_strata
  Analysis time _t: tte_730_strata

                                 |-------------- Per subject ---------
-----|
Category                   Total        Mean         Min     Median
Max
----------------------------------------------------------------------
------
Number of subjects        208061
Number of records         208061           1           1          1
1

Entry time (first)                         0           0          0
0
Exit time (final)                   368.0602          .5        417
730

Subjects with gap              0
Time on gap                    0
Time at risk            76578980    368.0602          .5        417
730

Failures                   18128    .0871283           0          0
1
----------------------------------------------------------------------
```

```
------

.         stdescribe if infected==0

        Failure _d: died_730_strata
  Analysis time _t: tte_730_strata

                                        |-------------- Per subject ---------
-----|
Category                   Total        Mean         Min     Median
Max
--------------------------------------------------------------------------
------
Number of subjects       1037423
Number of records        1037423           1           1          1
1

Entry time (first)                         0           0          0
0
Exit time (final)                    386.542          .5        424
730

Subjects with gap              0
Time on gap                    0
Time at risk           4.010e+08     386.542          .5        424
730

Failures                   42399    .0408695           0          0
1
--------------------------------------------------------------------------
------

.         *sts test infected, logrank
.         sts graph, by(infected) name(survival0730, replace) ///
>                 legend(pos(5) ring(0) rows(2) region(lcolor(none)) ///
>                                  size(small) label(1 "Comparators")
label(2 "COVID-19 Cases"))    ///
>                 graphregion(color(white)) bgcolor(white) title("Days
0-730", color(black) size(medium)) ///
>                 xtitle("") ///
>                 plot1opts(lpattern(solid) lcolor("0 114 178")) ///
>                 plot2opts(lpattern(dash) lcolor("230 159 0"))   ///
>                 tlabel(, labsize(small)) ylabel(, labsize(small))

        Failure _d: died_730_strata
  Analysis time _t: tte_730_strata

.         *graph save "survival0730" "Figures\survival0730.gph", replace
. restore

.
.
. *-----------------------------------
. * Table 2: PP-Unweighted Cox Models
```

```
. *-------------------------------------
.
. * Overall
.
. preserve

.
.         stset tte_730_strata, failure(died_730_strata) id(uniq_patid)

Survival-time data settings

           ID variable: uniq_patid
         Failure event: died_730_strata!=0 & died_730_strata<.
Observed time interval: (tte_730_strata[_n-1], tte_730_strata]
     Exit on or before: failure

-----------------------------------------------------------------------
--
  1,245,484  total observations
        377  observations end on or before enter()
-----------------------------------------------------------------------
--
  1,245,107  observations remaining, representing
  1,245,107  subjects
     60,186  failures in single-failure-per-subject data
  477586398  total analysis time at risk and under observation
                                                At risk from t =
0
                                     Earliest observed entry t =
0
                                          Last observed exit t =
730

.         stcox infected, efron vce(cluster patienticn)
strata(matchgroupnumber)

        Failure _d: died_730_strata
  Analysis time _t: tte_730_strata
       ID variable: uniq_patid

Iteration 0:   log pseudolikelihood = -105656.04
Iteration 1:   log pseudolikelihood = -102322.95
Iteration 2:   log pseudolikelihood = -102012.93
Iteration 3:   log pseudolikelihood = -102012.62
Refining estimates:
Iteration 0:   log pseudolikelihood = -102012.62

Stratified Cox regression with Efron method for ties
Strata variable: matchgroupnumber

No. of subjects = 1,245,107                          Number of obs =
1,245,107
No. of failures =    60,186
Time at risk    = 477586398
```

```
                                                    Wald chi2(1)  =
9220.08
Log pseudolikelihood = -102012.62                   Prob > chi2   =
0.0000

                     (Std. err. adjusted for 1,091,916 clusters in
patienticn)
------------------------------------------------------------------------
------
             |               Robust
          _t | Haz. ratio   std. err.      z    P>|z|     [95% conf.
interval]
-------------+----------------------------------------------------------
------
    infected |   2.270487   .0193893    96.02   0.000       2.2328
2.308809
------------------------------------------------------------------------
------


.
. restore

.
.
. * By period
.
. preserve

.
.          * stset dataset with new censoring and failure variables
.          stset tte_730_strata, failure(died_730_strata) id(uniq_patid)

Survival-time data settings

           ID variable: uniq_patid
         Failure event: died_730_strata!=0 & died_730_strata<.
Observed time interval: (tte_730_strata[_n-1], tte_730_strata]
     Exit on or before: failure

------------------------------------------------------------------------
--
  1,245,484  total observations
        377  observations end on or before enter()
------------------------------------------------------------------------
--
  1,245,107  observations remaining, representing
  1,245,107  subjects
     60,186  failures in single-failure-per-subject data
  477586398  total analysis time at risk and under observation
                                                At risk from t =
0
                                     Earliest observed entry t =
0
                                          Last observed exit t =
```

```
730

.         stsplit period, at(0 90 180 365 730)
(2,978,471 observations (episodes) created)

.
.         * create new interaction variables after splitting data
.         gen timeinfected_0_90 = infected * (tte_730_strata<=90)

.         gen timeinfected_91_180 = infected * (tte_730_strata>90 &
tte_730_strata<=180)

.         gen timeinfected_181_365 = infected * (tte_730_strata>180 &
tte_730_strata<=365)

.         gen timeinfected_366_730 = infected * (tte_730_strata>365 &
tte_730_strata<=730)

.
.         *------------------------
.         * Main Model - Unadj.
.         *------------------------
.
.         stcox   timeinfected_0_90 timeinfected_91_180
timeinfected_181_365  ///
>                         timeinfected_366_730
///
>                         , efron vce(cluster patienticn)
strata(matchgroupnumber)

        Failure _d: died_730_strata
  Analysis time _t: tte_730_strata
       ID variable: uniq_patid

Iteration 0:   log pseudolikelihood = -105656.04
Iteration 1:   log pseudolikelihood = -99350.316
Iteration 2:   log pseudolikelihood = -97168.116
Iteration 3:   log pseudolikelihood = -97023.527
Iteration 4:   log pseudolikelihood = -97023.522
Refining estimates:
Iteration 0:   log pseudolikelihood = -97023.522

Stratified Cox regression with Efron method for ties
Strata variable: matchgroupnumber

No. of subjects = 1,245,107                          Number of obs =
4,223,578
No. of failures =    60,186
Time at risk    = 477586398
                                                     Wald chi2(4)  =
22888.39
Log pseudolikelihood = -97023.522                    Prob > chi2   =
0.0000
```

```
                              (Std. err. adjusted for 1,091,916 clusters
in patienticn)
--------------------------------------------------------------------------
--------------
                     |               Robust
                  _t | Haz. ratio   std. err.      z    P>|z|     [95%
conf. interval]
---------------------+----------------------------------------------------
--------------
   timeinfected_0_90 |   6.472236   .0799848   151.12   0.000
6.317352    6.630917
  timeinfected_91_180 |   1.164641   .0279073     6.36   0.000
1.111209    1.220643
timeinfected_181_365 |   .9202852   .0187548    -4.08   0.000
.8842509    .9577879
timeinfected_366_730 |   .8847994   .0234548    -4.62   0.000
.8400027     .931985
--------------------------------------------------------------------------
--------------

.
. restore

.
.
. log close
      name:  
       log:  ...\LTO Mortality\Logs\analysis_lto_mortality_20230605.log
  log type:  text
 closed on:   5 Jun 2023, 16:24:17
--------------------------------------------------------------------------
--------------------------------------------------------------------------
------------------------------------------------
```
