# Supplementary material for: Late Mortality After COVID-19 Infection Among US Veterans vs Risk-Matched Comparators: A 2-Year Cohort Analysis
Source: JAMA Intern Med. 2023 Aug 21;183(10):1111–9. doi: 10.1001/jamainternmed.2023.3587 (PMC10442778; doi:10.1001/jamainternmed.2023.3587)
Supplement: Supplement 3. — Stata code .do file [file jamainternmed-e233587-s003.pdf]

```
clear all
cap more off
cap log close
version 17.0
cd ""

local day : display %tdCYND daily("$S_DATE", "DMY")
di "`day'"

log using "Logs\analysis_lto_mortality_`day'.log", replace

**************************************************************************
*
* Project:  LTO Mortality in COVID+ patients
*
* Author: Sarah Seelye
*
* Date Created: 2022 May 24
* Date Updated: 2023 June 5
**************************************************************************
*

* open death dataset using Portland mortality definitions
use Data\cohort_dod_allsrc, clear

* address duplicates
duplicates report patienticn
duplicates report patienticn birthdate
 //all of the duplicates have the same birthdate and patienticn.
duplicates report patienticn scrssn_numeric
 //patienticn and SCRSSN uniquely identify patients, but SCRSSN is not
 //included in the matchedcohort dataset
duplicates tag patienticn, gen(dup)
tab dup //266 duplicates; 133 unique Vets
bysort patienticn (scrssn): gen patientn = _n
bysort patienticn: gen dod_notsame = 1 if
best_death_date!=best_death_date[_n-1] & patientn==2
bysort patienticn: egen dod_notsame_pat = max(dod_notsame)
tab dod_notsame_pat patientn

* drop duplicates that have different birth dates
drop if dup==1 & dod_notsame_pat==1

* drop duplicate patienticns for second SCRSSN on record
drop if patientn==2

* keep variables for merge
keep patienticn best_death_date
destring patienticn, replace

format best_death_date %td

tempfile dod
```

```
save `dod'

* merge dod dataset with updated _datalong dataset(version 8/22/2022)
use Data\matchedcohort_25to1_datalong, clear
destring patienticn, replace
format patienticn %12.0g

merge m:1 patienticn using `dod'
drop _merge

*------------------------------
* Build 5:1 Matched Cohort
*------------------------------

* drop comparators who aren't matched to cases
tab ismatched_case
drop if ismatched_case==0
count //n=5,381,936
tab case //case=208,536

* create a new index_dt variable to use the case's index date for all
controls
gen index_dt_case = index_dt if case==1
format index_dt_case %td
gsort matchgroupnumber -case
by matchgroupnumber: replace index_dt_case = index_dt_case[_n-1] if
index_dt_case[_n-1]!=.

* create an indextodeath variable to count number of days between index
date
* and death
gen indextodeath_caseindex = best_death_date-index_dt_case
sum indextodeath_caseindex
sum indextodeath_caseindex if indextodeath_caseindex<0

* drop indextodeath_caseindex<0 to exclude observations that die before
the
* index date
drop if indextodeath_caseindex<0
tab case //case=208,063

* drop those with an infection date prior to index date
* create time-to-infection variable
gen ttinfection = futureinfectedcontrolindexdate - index_dt_case

sum ttinfection
sum ttinfection if ttinfection <0
drop if ttinfection<0 //n=1411
tab case //case=208,063

drop ttinfection

* keep the best 5 matches
gsort matchgroupnumber propensityscoreabsdiffrank -case
```

```
by matchgroupnumber: gen n = _n
tab n

keep if n<=6

tab n
tab n case

bysort matchgroupnumber (n): egen totalpairs = max(n)
tab totalpairs
tab totalpairs case // 3 patients - 2 cases - have no matched pair

* drop the 3 patients with no matched pair
drop if totalpairs==1
tab case //case=208,061

* confirm that all matched groups have a case
bysort matchgroupnumber: egen groupwithacase = max(case)
tab groupwithacase //2,829 comparators don't have a matched case

* drop comparator observations who don't have a matched case
drop if groupwithacase==0 //2,829 comparators dropped

* confirm that each case does not have more than 5 controls
gen control = case==0
by matchgroupnumber: egen totalcontrols = sum(control)
tab totalcontrols if case==1

* count
tab case //n=1,245,484, of which 208,061 are cases

* drop variables no longer needed
drop n totalpairs groupwithacase control totalcontrols

* merge with covid+ hospitalization dataset
merge 1:1 matchgroupnumber patienticn using
Data\Hospitalizations\lto_mortality_hospitalizations_20230516
drop if _merge==2
drop _merge
count //1,245,484

recode index_hospitalization (.=0)

* identify matchgroups in which the covid+ case was hospitalized/not
hospitalized
gen case_hospitalized=index_hospitalization==1 if case==1
bysort matchgroupnumber: egen case_hospitalized_matchgrp =
max(case_hospitalized)

drop case_hospitalized

*--------------------------
* Construct Covariates
*--------------------------
```

```
* create a new unique patient id variable that combines patienticn &
matchgroupnumber
tostring patienticn, gen(patienticn_str)
tostring matchgroupnumber, gen(matchgroup_str)

gen uniq_patid_str = patienticn_str + matchgroup_str
destring uniq_patid_str, gen(uniq_patid)
format uniq_patid %14.0g

drop patienticn_str matchgroup_str uniq_patid_str
duplicates report uniq_patid

* wave of infection
 * March-June 2020
 * July-Nov 2020
 * Dec 2020-Apr 2021

tab index_month

gen wave = .
replace wave = 1 if inrange(index_month, 0, 3)
replace wave = 2 if inrange(index_month, 4, 8)
replace wave = 3 if inrange(index_month, 9, 13)
tab index_month wave

* age categories
sum ageatindexdate

gen agecat = .
replace agecat=1 if ageatindexdate<65
replace agecat=2 if inrange(ageatindexdate, 65, 85)
replace agecat=3 if ageatindexdate>85

* create indicator for COVID+ cases
gen infected = .
replace infected = 1 if case==1
replace infected = 0 if case==0

* label values of categorical variables
tab sex3cat infected
recode sex3cat 0=99
lab def sex3cat 99 "Unknown" 1 "Female" 2 "Male", replace
lab val sex3cat sex3cat
tab sex3cat infected

tab race7cat infected
recode race7cat 0=99
lab def race7cat 1 "AmericanIndian/AlaskaNative" 2 "Asian" 3
"Black/AfricanAmer" ///
     4 "NativeHawaiian/PacificIsland" 5 "White" 6 "MultipleRace"     ///
     99 "Missing" , replace
lab val race7cat race7cat
tab race7cat infected
```

```
tab ethnicity3cat infected
recode ethnicity3cat 0=99
lab def ethnicity3cat 1 "Hispanic" 2 "NotHispanic" 99 "Missing", replace
lab val ethnicity3cat ethnicity3cat
tab ethnicity3cat infected, nol

tab rurality2cat infected
lab def rurality2cat 0 "NotUrban" 1 "Urban", replace
lab val rurality2cat rurality2cat
tab rurality2cat infected

tab smoking4cat infected
recode smoking4cat 0=99
lab def smoking4cat 1 "Current" 2 "Former" 3 "Never" 99 "Missing",
replace
lab val smoking4cat smoking4cat
tab smoking4cat infected

tab immuno infected
tab clcatindexdate infected

tab nosos11cat infected
recode nosos11cat 0=99
tab nosos11cat infected

tab canscore7cat infected
recode canscore7cat 0=99
tab canscore7cat infected

*---------------------------
* Time to Study End
*---------------------------

* identify study end as April 1, 2022
gen studyend = mdy(4, 1, 2022)
format studyend %td

* count days from index to studyend
gen ttstudyend = studyend-index_dt_case

*------------------------
* Mortality Variables
*------------------------

* Alive on day 91
gen alive_day91 = .
replace alive_day91 = 1 if indextodeath_caseindex>=91
replace alive_day91 = 0 if inrange(indextodeath_caseindex, 0, 90)
replace alive_day91 = 0 if ttstudyend<91
bysort alive_day91: sum indextodeath_caseindex

* Alive on day 181
gen alive_day181 = .
```

```
replace alive_day181 = 1 if indextodeath_caseindex>=181
replace alive_day181 = 0 if inrange(indextodeath_caseindex, 0, 180)
replace alive_day181 = 0 if ttstudyend<181
bysort alive_day181: sum indextodeath_caseindex

* Alive on day 366
gen alive_day366 = .
replace alive_day366 = 1 if indextodeath_caseindex>=366
replace alive_day366 = 0 if inrange(indextodeath_caseindex, 0, 365)
replace alive_day366 = 0 if ttstudyend<366
bysort alive_day366: sum indextodeath_caseindex

* Died Index Day-Day 90
gen died_0_90 = .
replace died_0_90 = 1 if inrange(indextodeath_caseindex, 0, 90)
replace died_0_90 = 0 if indextodeath_caseindex>90
tab died_0_90, m

* Died Day 91-Day 180
gen died_91_180 = .
replace died_91_180 = 1 if inrange(indextodeath_caseindex, 91, 180)
replace died_91_180 = 0 if indextodeath_caseindex>180
replace died_91_180 = 0 if (indextodeath_caseindex>ttstudyend) &
(inrange(indextodeath_caseindex, 91, 180)) ///
          & (inrange(ttstudyend, 91, 180))
tab died_91_180, m
sum indextodeath_caseindex if indextodeath_caseindex<91

* Died Day 181-Day 365
gen died_181_365 = .
replace died_181_365 = 1 if inrange(indextodeath_caseindex, 181, 365)
replace died_181_365 = 0 if indextodeath_caseindex>365
replace died_181_365 = 0 if (indextodeath_caseindex>ttstudyend) &
(inrange(indextodeath_caseindex, 181, 365)) ///
          & (inrange(ttstudyend, 181, 365))
tab died_181_365, m
sum indextodeath_caseindex if indextodeath_caseindex<181

* Died Day 366-730
gen died_366_730 = .
replace died_366_730 = 1 if inrange(indextodeath_caseindex, 366, 730)
replace died_366_730 = 0 if indextodeath_caseindex>730
replace died_366_730 = 0 if (indextodeath_caseindex>ttstudyend) &
(inrange(indextodeath_caseindex, 366, 730)) ///
          & (inrange(ttstudyend, 366, 730))
tab died_366_730, m
sum indextodeath_caseindex if indextodeath_caseindex<366

* Died Day 0-365
gen died_0_365 = .
replace died_0_365 = 1 if inrange(indextodeath_caseindex, 0, 365)
replace died_0_365 = 0 if indextodeath_caseindex>365
replace died_0_365 = 0 if (indextodeath_caseindex>ttstudyend) &
(inrange(indextodeath_caseindex, 0, 365)) ///
```

```
          & (inrange(ttstudyend, 0, 365))

* Died Day 0-730
gen died_0_730 = .
replace died_0_730 = 1 if inrange(indextodeath_caseindex, 0, 730)
replace died_0_730 = 0 if indextodeath_caseindex>730
replace died_0_730 = 0 if (indextodeath_caseindex>ttstudyend) &
(inrange(indextodeath_caseindex, 0, 730)) ///
          & (inrange(ttstudyend, 0, 730))

* Died Day 91-730
gen died_91_730 = .
replace died_91_730 = 1 if inrange(indextodeath_caseindex, 91, 730)
replace died_91_730 = 0 if indextodeath_caseindex>730
replace died_91_730 = 0 if (indextodeath_caseindex>ttstudyend) &
(inrange(indextodeath_caseindex, 91, 730)) ///
          & (inrange(ttstudyend, 91, 730))

*----------------------------
* Time to Event Variables
*----------------------------

* alive on index day, censored at 90 days
gen tte_0_90 = ttstudyend
replace tte_0_90 = 90 if ttstudyend>90
replace tte_0_90 = indextodeath_caseindex  ///
     if indextodeath_caseindex180
replace tte_91_180 = indextodeath_caseindex if  ///
      indextodeath_caseindex365
replace tte_181_365 = indextodeath_caseindex if  ///
      indextodeath_caseindex730
replace tte_366_730 = indextodeath_caseindex if  ///
      indextodeath_caseindex365
replace tte_0_365 = indextodeath_caseindex if ///
      indextodeath_caseindex730
replace tte_0_730 = indextodeath_caseindex if ///
      indextodeath_caseindex730
replace tte_91_730 = indextodeath_caseindex if  ///
      indextodeath_caseindex=1

 tab alive_day`i'_casecontrol case

 //drop variables we no longer need
 drop alive_day`i'_case alive_day`i'_case_grp alive_day`i'_control
alive_day`i'_control_num

}

* Save dataset for use in tables and Cox models
*save Data\mortality_varforcox_20230516, replace

*-----------------
* ITT Results
*-----------------
```

```
use Data\mortality_varforcox_20230516, clear

* excess deaths (Results)
tab died_0_730 infected, co

di 20409/208061 // 0.09809143 - mortality rate of cases
di 61988/1037423 // 0.0597519 - moratlity rate of comparators
di 208061*0.0597519 //12432.04 - number case deaths if comparator rate
di 20409-12432.04  //7976.96 excess deaths

* Overall *

* 90-day mortality for cases and comparators who are alive on index day
tab died_0_90 infected  , co
cs died_0_90 infected

* 180-day mortality for cases and comparators who are alive on day 91 &
* with case and at least one comparator from matchgroup alive
tab died_91_180 infected if alive_day91_casecontrol==1 , co
cs died_91_180 infected if alive_day91_casecontrol==1

* 365-day mortality for cases and comparators who are alive on day 181 &
not censored
tab died_181_365 infected if alive_day181_casecontrol==1 , co
cs died_181_365 infected if alive_day181_casecontrol==1

* 730-day mortality for cases and comparators who are alive on day 366 &
not censored
tab died_366_730 infected if alive_day366_casecontrol==1 , co
cs died_366_730 infected if alive_day366_casecontrol==1

*-----------
* Age <65
*-----------

* 90-day mortality for cases and comparators who are alive on day 1
tab died_0_90 infected if agecat==1 & ttstudyend>=0, co
cs died_0_90 infected if agecat==1 & ttstudyend>=0

* 180-day mortality for cases and comparators who are alive on day 91
tab died_91_180 infected if alive_day91_casecontrol==1 & agecat==1 &
ttstudyend>=91, co
cs died_91_180 infected if alive_day91_casecontrol==1 & agecat==1 &
ttstudyend>=91

* 365-day mortality for cases and comparators who are alive on day 181 &
not censored
tab died_181_365 infected if alive_day181_casecontrol==1 & agecat==1 &
ttstudyend>=181, co
cs died_181_365 infected if alive_day181_casecontrol==1 & agecat==1 &
ttstudyend>=181

* 730-day mortality for cases and comparators who are alive on day 366 &
```

```
not censored
tab died_366_730 infected if alive_day366_casecontrol==1 & agecat==1 &
ttstudyend>=366, co
cs died_366_730 infected if alive_day366_casecontrol==1 & agecat==1 &
ttstudyend>=366

*------------
* Age 65-85
*------------

* 90-day mortality for cases and comparators who are alive on day 1
tab died_0_90 infected if agecat==2 & ttstudyend>=0, co
cs died_0_90 infected if agecat==2 & ttstudyend>=0

* 180-day mortality for cases and comparators who are alive on day 91
tab died_91_180 infected if alive_day91_casecontrol==1 & agecat==2 &
ttstudyend>=91, co
cs died_91_180 infected if alive_day91_casecontrol==1 & agecat==2 &
ttstudyend>=91

* 365-day mortality for cases and comparators who are alive on day 181 &
not censored
tab died_181_365 infected if alive_day181_casecontrol==1 & agecat==2 &
ttstudyend>=181, co
cs died_181_365 infected if alive_day181_casecontrol==1 & agecat==2 &
ttstudyend>=181

* 730-day mortality for cases and comparators who are alive on day 366 &
not censored
tab died_366_730 infected if alive_day366_casecontrol==1 & agecat==2 &
ttstudyend>=366, co
cs died_366_730 infected if alive_day366_casecontrol==1 & agecat==2 &
ttstudyend>=366

*------------
* Age 85+
*------------

* 90-day mortality for cases and comparators who are alive on day 1
tab died_0_90 infected if agecat==3 & ttstudyend>=0, co
cs died_0_90 infected if agecat==3 & ttstudyend>=0

* 180-day mortality for cases and comparators who are alive on day 91
tab died_91_180 infected if alive_day91_casecontrol==1 & agecat==3 &
ttstudyend>=91, co
cs died_91_180 infected if alive_day91_casecontrol==1 & agecat==3 &
ttstudyend>=91

* 365-day mortality for cases and comparators who are alive on day 181 &
not censored
tab died_181_365 infected if alive_day181_casecontrol==1 & agecat==3 &
ttstudyend>=181, co
cs died_181_365 infected if alive_day181_casecontrol==1 & agecat==3 &
ttstudyend>=181
```

```
* 730-day mortality for cases and comparators who are alive on day 366 &
not censored
tab died_366_730 infected if alive_day366_casecontrol==1 & agecat==3 &
ttstudyend>=366, co
cs died_366_730 infected if alive_day366_casecontrol==1 & agecat==3 &
ttstudyend>=366

*-----------
* Wave 1
*-----------

* 90-day mortality for cases and comparators who are alive on day 1
tab died_0_90 infected if wave==1 & ttstudyend>=0, co
cs died_0_90 infected if wave==1 & ttstudyend>=0

* 180-day mortality for cases and comparators who are alive on day 91
tab died_91_180 infected if alive_day91_casecontrol==1 & wave==1 &
ttstudyend>=91, co
cs died_91_180 infected if alive_day91_casecontrol==1 & wave==1 &
ttstudyend>=91

* 365-day mortality for cases and comparators who are alive on day 181 &
not censored
tab died_181_365 infected if alive_day181_casecontrol==1 & wave==1 &
ttstudyend>=181, co
cs died_181_365 infected if alive_day181_casecontrol==1 & wave==1 &
ttstudyend>=181

* 730-day mortality for cases and comparators who are alive on day 366 &
not censored
tab died_366_730 infected if alive_day366_casecontrol==1 & wave==1 &
ttstudyend>=181, co
cs died_366_730 infected if alive_day366_casecontrol==1 & wave==1 &
ttstudyend>=181

*-----------
* Wave 2
*-----------

* 90-day mortality for cases and comparators who are alive on day 1
tab died_0_90 infected if wave==2 & ttstudyend>=0, co
cs died_0_90 infected if wave==2 & ttstudyend>=0

* 180-day mortality for cases and comparators who are alive on day 91
tab died_91_180 infected if alive_day91_casecontrol==1 & wave==2 &
ttstudyend>=91, co
cs died_91_180 infected if alive_day91_casecontrol==1 & wave==2 &
ttstudyend>=91

* 365-day mortality for cases and comparators who are alive on day 181 &
not censored
tab died_181_365 infected if alive_day181_casecontrol==1 & wave==2 &
ttstudyend>=181, co
```

```
cs died_181_365 infected if alive_day181_casecontrol==1 & wave==2 &
ttstudyend>=181

* 730-day mortality for cases and comparators who are alive on day 366 &
not censored
tab died_366_730 infected if alive_day366_casecontrol==1 & wave==2 &
ttstudyend>=366, co
cs died_366_730 infected if alive_day366_casecontrol==1 & wave==2 &
ttstudyend>=366

*-----------
* Wave 3
*-----------

* 90-day mortality for cases and comparators who are alive on day 1
tab died_0_90 infected if wave==3 & ttstudyend>=0, co
cs died_0_90 infected if wave==3 & ttstudyend>=0

* 180-day mortality for cases and comparators who are alive on day 91
tab died_91_180 infected if alive_day91_casecontrol==1 & wave==3 &
ttstudyend>=91, co
cs died_91_180 infected if alive_day91_casecontrol==1 & wave==3 &
ttstudyend>=91

* 365-day mortality for cases and comparators who are alive on day 181 &
not censored
tab died_181_365 infected if alive_day181_casecontrol==1 & wave==3 &
ttstudyend>=181, co
cs died_181_365 infected if alive_day181_casecontrol==1 & wave==3 &
ttstudyend>=181

* 730-day mortality for cases and comparators who are alive on day 366 &
not censored
tab died_366_730 infected if alive_day366_casecontrol==1 & wave==3 &
ttstudyend>=366, co
cs died_366_730 infected if alive_day366_casecontrol==1 & wave==3 &
ttstudyend>=366


*-------------------------------------------------------
* Overall for comparators by later infection status
*-------------------------------------------------------

tab isfutureinfectedcontrol case , co
tab isfutureinfectedcontrol case if alive_day91, co
tab isfutureinfectedcontrol case if alive_day181, co
tab isfutureinfectedcontrol case if alive_day366, co

* 90-day mortality for comparators by later infection status who are
alive on index date
tab died_0_90 isfutureinfectedcontrol if infected==0 & ttstudyend>=0, co
cs died_0_90 isfutureinfectedcontrol if infected==0 & ttstudyend>=0

* 180-day mortality for comparators by later infection status who are
```

```
alive on day 91
tab died_91_180 isfutureinfectedcontrol if infected==0 & alive_day91==1
& ttstudyend>=91, co
cs died_91_180 isfutureinfectedcontrol if infected==0 & alive_day91==1 &
ttstudyend>=91

* 365-day mortality for comparators by later infection status who are
alive on day 181 & not censored
tab died_181_365 isfutureinfectedcontrol if infected==0 &
alive_day181==1 & ttstudyend>=181, co
cs died_181_365 isfutureinfectedcontrol if infected==0 & alive_day181==1
& ttstudyend>=181

* 730-day mortality for comparators by later infection status who are
alive on day 366 & not censored
tab died_366_730 isfutureinfectedcontrol if infected==0 &
alive_day366==1 & ttstudyend>=366, co
cs died_366_730 isfutureinfectedcontrol if infected==0 & alive_day366==1
& ttstudyend>=366


*--------------------------------------------
* Appendix Figure 2 - Kaplan Meier Curves
*--------------------------------------------

* 0-90 Days
preserve
 stset tte_0_90 , failure(died_0_90==1)
 sts test infected, logrank
 sts graph, by(infected) name(survival090itt, replace) ///
  legend(pos(5) ring(0) rows(2) region(lcolor(none)) ///
    size(small) label(1 "Comparators") label(2 "COVID-19 Cases"))    ///
  graphregion(color(white)) bgcolor(white) title("Days 0-90",
color(black) size(medium)) ///
  xtitle("") ///
  plot1opts(lpattern(solid) lcolor("0 114 178")) ///
  plot2opts(lpattern(dash) lcolor("230 159 0"))  ///
  tlabel(, labsize(small)) ylabel(, labsize(small))
 *graph save "survival090itt" "Figures\survival090itt.gph", replace
 stset , clear
restore

* 91-180 Days
preserve
 keep if alive_day91_casecontrol==1
 stset tte_91_180 , failure(died_91_180==1)
 sts test infected, logrank
 sts graph, by(infected) name(survival91180itt, replace) ///
  legend(pos(5) ring(0) rows(2) region(lcolor(none)) ///
    size(small) label(1 "Comparators") label(2 "COVID-19 Cases"))    ///
  graphregion(color(white)) bgcolor(white) title("Days 91-180",
color(black) size(medium)) ///
  xtitle("") ///
  plot1opts(lpattern(solid) lcolor("0 114 178")) ///
```

```
  plot2opts(lpattern(dash) lcolor("230 159 0"))  ///
  ylabel(0.9 (0.02) 1.0, labsize(small)) ///
  tmin(91) tlabel(90 (20) 190, labsize(small))
 *graph save "survival91180itt" "Figures\survival91180itt.gph", replace
 stset , clear
restore

* 181-365 Days
preserve
 keep if alive_day181_casecontrol==1
 stset tte_181_365 , failure(died_181_365==1)
 sts test infected, logrank
 sts graph, by(infected) name(survival181365itt, replace) ///
  legend(pos(5) ring(0) rows(2) region(lcolor(none)) ///
    size(small) label(1 "Comparators") label(2 "COVID-19 Cases"))    ///
  graphregion(color(white)) bgcolor(white) title("Days 181-365",
color(black) size(medium)) ///
  xtitle("") ///
  plot1opts(lpattern(solid) lcolor("0 114 178")) ///
  plot2opts(lpattern(dash) lcolor("230 159 0"))  ///
  ylabel(0.9 (0.02) 1.0, labsize(small)) ///
  tmin(181) tlabel(180 (40) 380, labsize(small))
 *graph save "survival181365itt" "Figures\survival181365itt.gph",
replace
 stset , clear
restore

* 366-730 Days
preserve
 keep if alive_day366_casecontrol==1
 stset tte_366_730 , failure(died_366_730==1)
 sts test infected, logrank
 sts graph, by(infected) name(survival366730itt, replace)  ///
  legend(pos(5) ring(0) rows(2) region(lcolor(none)) ///
    size(small) label(1 "Comparators") label(2 "COVID-19 Cases"))    ///
  graphregion(color(white)) bgcolor(white) title("Days 366-730",
color(black) size(medium)) ///
  xtitle("") ///
  plot1opts(lpattern(solid) lcolor("0 114 178")) ///
  plot2opts(lpattern(dash) lcolor("230 159 0"))  ///
  ylabel(0.9 (0.02) 1.0, labsize(small)) ///
  tmin(366) tlabel(365 450 550 650 730, labsize(small))
 *graph save "survival366730itt" "Figures\survival366730itt.gph",
replace
 stset , clear
restore

* 0-365 Days
preserve
 stset tte_0_365 , failure(died_0_365)
 *sts test infected, logrank
 sts graph, by(infected) name(survival0365, replace)
 stset , clear
restore
```

```
* 0-730 Days
preserve
 stset tte_0_730 , failure(died_0_730)
 stdescribe if infected==1
 stdescribe if infected==0
 *sts test infected, logrank
 sts graph, by(infected) name(survival0730itt, replace) ///
  legend(pos(5) ring(0) rows(2) region(lcolor(none)) ///
    size(small) label(1 "Comparators") label(2 "COVID-19 Cases"))    ///
  graphregion(color(white)) bgcolor(white) title("Days 0-730",
color(black) size(medium)) ///
  xtitle("") ///
  plot1opts(lpattern(solid) lcolor("0 114 178")) ///
  plot2opts(lpattern(dash) lcolor("230 159 0"))  ///
  tlabel(, labsize(small)) ylabel(, labsize(small))
 *graph save "survival0730itt" "Figures\survival0730itt.gph", replace
 stset , clear
restore

*------------------------
* Table 1: SMDs
*------------------------

* Baseline covariate balance for 5-to-1 match cohort

tab infected

stddiff ageatindexdate, by(infected)
stddiff bmi, by(infected)
stddiff i.sex3cat, by(infected)
stddiff i.race7cat, by(infected)
stddiff i.ethnicity3cat, by(infected)
stddiff i.rurality2cat, by(infected)
stddiff i.smoking4cat, by(infected)
stddiff gagne, by(infected)
stddiff numipadmits, by(infected)
stddiff util_numpcstops, by(infected)
stddiff util_numscstops, by(infected)
stddiff util_nummhstops, by(infected)
stddiff i.immuno, by(infected)
stddiff i.clcatindexdate, by(infected)
stddiff i.nosos11cat, by(infected)
stddiff i.canscore7cat, by(infected)
stddiff neareststa3ndistance, by(infected)


*******
* ITT *
*******

use Data\mortality_varforcox_20230516, clear

*------------------------------------
```

```
* Table 2: ITT Unadjusted
*-------------------------------------

* Overall
preserve
 sort uniq_patid
 stset tte_0_730, failure(died_0_730) id(uniq_patid)
 stcox infected, efron vce(cluster patienticn) strata(matchgroupnumber)
restore

* By Period

* stset data
sort uniq_patid
recode tte_0_730 (0=0.5)
stset tte_0_730, failure(died_0_730) id(uniq_patid)
stsplit period, at(0 90 180 365 730)

stdescribe if infected==1
stdescribe if infected==0

* create new interaction variables after splitting data
gen timeinfected_0_90 = infected * (tte_0_730<=90)
gen timeinfected_91_180 = infected * (tte_0_730>90 & tte_0_730<=180)
gen timeinfected_181_365 = infected * (tte_0_730>180 & tte_0_730<=365)
gen timeinfected_366_730 = infected * (tte_0_730>365 & tte_0_730<=730)

* Cox Model
stcox  timeinfected_0_90 timeinfected_91_180 timeinfected_181_365  ///
  timeinfected_366_730     ///
  , efron vce(cluster patienticn) strata(matchgroupnumber)

*-----------------------------------------------
* Subgroup Analyses - Hospitalization, Adj.
*-----------------------------------------------

* Subgroup analysis for hospitalized/not hospitalized & adjust for
* imbalanced hospitalization covariates (SMDs>0.1 for hospitalization
subgroups)


* Hospitalized Patients
local imbalanced ageatindexdate bmi i.ethnicity3cat i.smoking4cat ///
     gagne numipadmits util_numpcstops util_numscstops ///
     i.nosos11cat i.canscore7cat neareststa3ndistance

stcox  timeinfected_0_90 timeinfected_91_180 timeinfected_181_365  ///
  timeinfected_366_730 `imbalanced'  ///
  if index_hospitalization==1 ///
  , efron vce(cluster patienticn) strata(matchgroupnumber)

* Non-Hospitalized
stcox  timeinfected_0_90 timeinfected_91_180 timeinfected_181_365  ///
  timeinfected_366_730 `imbalanced'  ///
```

```
  if index_hospitalization==0 ///
  , efron vce(cluster patienticn) strata(matchgroupnumber)

*-----------------------------------
* Subgroup Analysis - Age, Adj.
*-----------------------------------

local ageimbal   bmi i.ethnicity3cat i.smoking4cat ///
     gagne numipadmits util_numpcstops util_numscstops ///
     i.nosos11cat i.canscore7cat nearest sta3ndistance

stcox  timeinfected_0_90 timeinfected_91_180 timeinfected_181_365  ///
  timeinfected_366_730 `ageimbal'  ///
  if agecat==1 ///
  , efron vce(cluster patienticn) strata(matchgroupnumber)

stcox  timeinfected_0_90 timeinfected_91_180 timeinfected_181_365  ///
  timeinfected_366_730 `ageimbal'  ///
  if agecat==2 ///
  , efron vce(cluster patienticn) strata(matchgroupnumber)

stcox  timeinfected_0_90 timeinfected_91_180 timeinfected_181_365  ///
  timeinfected_366_730 `ageimbal'  ///
  if agecat==3 ///
  , efron vce(cluster patienticn) strata(matchgroupnumber)


*-----------------------------------------------
* Subgroup Analyses - Comorbidities, Adj.
*-----------------------------------------------

* Subgroup analysis by tertile of comorbidity & adjust for
* covariates

* create tertiles of Gagne scores
sum gagne, de
histogram gagne
xtile gagne_tertile = gagne, nq(3)
bysort gagne_tertile: sum gagne, de

* Tertiles of Gagne scores
local imbalanced ageatindexdate bmi i.ethnicity3cat i.smoking4cat ///
     numipadmits util_numpcstops util_numscstops ///
     i.nosos11cat i.canscore7cat neareststa3ndistance

forval i=1/3 {

 stcox  timeinfected_0_90 timeinfected_91_180 timeinfected_181_365  ///
   timeinfected_366_730 `imbalanced'  ///
   if gagne_tertile==`i'  ///
   , efron vce(cluster patienticn) strata(matchgroupnumber)
}
```

```
*******************************************************************
* PP1: Per Protocol for IPCWs - Censoring at Crossover Infections *
*******************************************************************

*---------------------------------------
* Prepping Data for IPCWs and Table 1
*---------------------------------------

use Data\mortality_varforcox_20230516, clear

* create ttinfection variable
gen ttinfection = futureinfectedcontrolindexdate - index_dt_case
sum ttinfection
count if ttinfection==0

*-----------------------------------------------------------------------
--------
* identify those who die during each period; those who die after
infection
* crossover are coded as not dead at the time of crossover
*-----------------------------------------------------------------------
--------

* Died Index Day-Day 90;
gen died_0_90_crossover = .
replace died_0_90_crossover = 1 if inrange(indextodeath_caseindex, 0,
90)
replace died_0_90_crossover = 0 if indextodeath_caseindex>90
replace died_0_90_crossover = 0 if indextodeath_caseindex>ttinfection

tab died_0_90 died_0_90_crossover, m
tab died_0_90_crossover case, m

* Died Day 91-180;
gen died_91_180_crossover = .
replace died_91_180_crossover = 1 if inrange(indextodeath_caseindex, 91,
180)
replace died_91_180_crossover = 0 if indextodeath_caseindex>180
replace died_91_180_crossover = 0 if indextodeath_caseindex>ttinfection
replace died_91_180_crossover = 0 if (indextodeath_caseindex>ttstudyend)
& (inrange(indextodeath_caseindex, 91, 180)) ///
          & (inrange(ttstudyend, 91, 180))
tab died_91_180 died_91_180_crossover, m //missing represents those who
were censored in period 1
tab died_91_180_crossover case, m

* Died Day 181-365;
gen died_181_365_crossover = .
replace died_181_365_crossover = 1 if inrange(indextodeath_caseindex,
181, 365)
replace died_181_365_crossover = 0 if indextodeath_caseindex>365
replace died_181_365_crossover = 0 if indextodeath_caseindex>ttinfection
replace died_181_365_crossover = 0 if
```

```
(indextodeath_caseindex>ttstudyend) & (inrange(indextodeath_caseindex,
181, 365)) ///
          & (inrange(ttstudyend, 181, 365))

tab died_181_365 died_181_365_crossover, m //missing represents those
who were censored in period 1&2
tab died_181_365_crossover case, m

* Died Day 366-730;
gen died_366_730_crossover = .
replace died_366_730_crossover = 1 if inrange(indextodeath_caseindex,
366, 730)
replace died_366_730_crossover = 0 if indextodeath_caseindex>730
replace died_366_730_crossover = 0 if indextodeath_caseindex>ttinfection
replace died_366_730_crossover = 0 if
(indextodeath_caseindex>ttstudyend) & (inrange(indextodeath_caseindex,
366, 730)) ///
          & (inrange(ttstudyend, 366, 730))

tab died_366_730 died_366_730_crossover, m //missing represents those
who were censored in period 1-3
tab died_366_730_crossover case, m

tab died_366_730 died_366_730_crossover if case==1
tab died_366_730 died_366_730_crossover if case==0

*------------------------------------------------------------------------
--------
* for each period, identify whether patient is alive on the first day of
the
* period AND was not an infection crossover in the prior period(s)
*------------------------------------------------------------------------
--------

* Alive on day 91 & no crossover in prior period
gen alive_day91_crossover = .
replace alive_day91_crossover = 1 if indextodeath_caseindex>=91
replace alive_day91_crossover = 0 if inrange(indextodeath_caseindex, 0,
90)
replace alive_day91_crossover = 0 if inrange(ttinfection, 0, 90)
replace alive_day91_crossover = 0 if ttstudyend<91

sum ttinfection if inrange(ttinfection, 0, 90)
tab alive_day91 alive_day91_crossover, m
tab alive_day91_crossover died_91_180_crossover
tab alive_day91 alive_day91_crossover if case==1
tab alive_day91 alive_day91_crossover if case==0
tab alive_day91_crossover died_0_90_crossover, m
sum ttinfection if ttinfection<91 //17044 crossover infections prior to
day 91

* Alive on day 181 & no crossover in prior periods
gen alive_day181_crossover = .
replace alive_day181_crossover = 1 if indextodeath_caseindex>=181
```

```
replace alive_day181_crossover = 0 if inrange(indextodeath_caseindex, 0,
180)
replace alive_day181_crossover = 0 if inrange(ttinfection, 0, 180)
replace alive_day181_crossover = 0 if ttstudyend<181

sum ttinfection if inrange(ttinfection, 0, 180)
tab alive_day181 alive_day181_crossover, m
tab alive_day181 alive_day181_crossover if case==1
tab alive_day181 alive_day181_crossover if case==0
tab alive_day181_crossover died_91_180_crossover
sum ttinfection if ttinfection<181 //29,870 crossover infections prior
to day 181
tab died_181_365_crossover case if alive_day181_crossover

* Alive on day 366 & no crossover in prior periods
gen alive_day366_crossover = .
replace alive_day366_crossover = 1 if indextodeath_caseindex>=366
replace alive_day366_crossover = 0 if inrange(indextodeath_caseindex, 0,
365)
replace alive_day366_crossover = 0 if inrange(ttinfection, 0, 365)
replace alive_day366_crossover = 0 if ttstudyend<366

sum ttinfection if inrange(ttinfection, 0, 365)
tab alive_day366 alive_day366_crossover
tab alive_day366 alive_day366_crossover if case==1
tab alive_day366 alive_day366_crossover if case==0
tab alive_day366_crossover died_181_365_crossover
sum ttinfection if ttinfection<366  //72253 crossover infections prior
to day 181
tab died_366_730_crossover case if alive_day366_crossover

* check mortality by period prior to applying below criteria
tab died_0_90_crossover case, co
tab died_91_180_crossover case, co
tab died_181_365_crossover case, co
tab died_366_730_crossover case, co

*----------------------------------------------------------------------
--------
* for each period, identify whether the case and at least one comparator
* in a matched group are alive on the first day of the period & were not
* an infection crossover in the prior period
*----------------------------------------------------------------------
--------

* drop previous versions of variable

foreach i of numlist 91 181 366 {
 drop alive_day`i'_casecontrol
}

foreach i of numlist 91 181 366 {

 //cases
```

```
 gen alive_day`i'_case = 0
 replace alive_day`i'_case = 1 if case==1 & alive_day`i'_crossover==1
 replace alive_day`i'_case = 0 if case==1 & ttstudyend<`i'
 tab alive_day`i'_case alive_day`i'_crossover if case==1
 sum indextodeath if indextodeath<`i' & case==1

 bysort matchgroupnumber: egen alive_day`i'_case_grp =
max(alive_day`i'_case)

 //comparators
 gen alive_day`i'_control = 0
 replace alive_day`i'_control = 1 if case==0 & alive_day`i'_crossover==1
 replace alive_day`i'_control = 0 if case==0 & ttstudyend<`i'

 tab alive_day`i'_control alive_day`i'_crossover if case==0
 sum indextodeath if indextodeath<`i' & case==0

 bysort matchgroupnumber: egen alive_day`i'_control_num =
sum(alive_day`i'_control)

 //case and at least one comparator alive on day`i'
 gen alive_day`i'_crosscasecontrol = 0
 replace alive_day`i'_crosscasecontrol = 1 if alive_day`i'_case_grp==1 &
alive_day`i'_control_num>=1

 tab alive_day`i'_crosscasecontrol case
}

* drop variables we no longer need

foreach i of numlist 91 181 366 {
 drop alive_day`i'_case alive_day`i'_case_grp alive_day`i'_control
alive_day`i'_control_num
}

*-----------------------------------------------
* Create categories for PP1 subgroup analyses
*-----------------------------------------------

* Note: all categories are in relation to the original infected case *

** age categories **

* <65
sum ageatindexdate

gen mkg_age_less65_case = 0
replace mkg_age_less65=1 if case==1 & ageatindexdate<65

bysort matchgroupnumber (patienticn): egen age_less65_case =
max(mkg_age_less65_case)
tab age_less65_case

drop mkg_age_less65_case
```

```
* 65-85
gen mkg_age_65to85_case = 0
replace mkg_age_65to85_case=1 if case==1 & inrange(ageatindexdate, 65, 
85)

bysort matchgroupnumber (patienticn): egen age_65to85_case = 
max(mkg_age_65to85_case)
tab age_65to85_case

drop mkg_age_65to85_case

* >85
gen mkg_age_more85_case = 0
replace mkg_age_more85_case=1 if case==1 & ageatindexdate>85

bysort matchgroupnumber (patienticn): egen age_more85_case = 
max(mkg_age_more85_case)
tab age_more85_case

drop mkg_age_more85_case


** Tertiles of Gagne scores **
sum gagne if case==1, de
xtile mkg_gagne_tertile = gagne if case==1, nq(3)

bysort mkg_gagne_tertile: sum gagne

bysort matchgroupnumber (patienticn): egen gagne_tertile_case = 
max(mkg_gagne_tertile)
sum gagne_tertile_case

** Probabilities of death in first 90 days **

* fit logistic regression model for comparator deaths during first 90 
days
local covar ageatindexdate bmi i.sex3cat i.race7cat i.ethnicity3cat ///
   i.rurality2cat i.smoking4cat gagne numipadmits util_numpcstops ///
   util_numscstops util_nummhstops i.immuno i.clcatindexdate ///
   i.nosos11cat i.canscore7cat neareststa3ndistance

logit died_0_90 `covar' if case==0
lroc, nograph //0.846
predict mkg_pr_died0_90
gen mkg_pr_died0_90_case = mkg_pr_died0_90 if case==1

bysort matchgroupnumber (patienticn): egen pr_died0_90_case = 
max(mkg_pr_died0_90_case)

* create tertiles of predicted probabilities of death in first 90 days
xtile pr_died0_90_tertile = pr_died0_90_case, nq(3)
tab pr_died0_90_tertile
```

```
bysort pr_died0_90_tertile: sum pr_died0_90_case


** Probabilities of death in days 181-730 **

* died in days 181-730
gen died_181_730 = !missing(best_death_date)
replace died_181_730 = 0 if indextodeath_caseindex<181 |
indextodeath_caseindex>730

* fit logistic regression model for comparator deaths during 181-730
days
local covar ageatindexdate bmi i.sex3cat i.race7cat i.ethnicity3cat ///
   i.rurality2cat i.smoking4cat gagne numipadmits util_numpcstops ///
   util_numscstops util_nummhstops i.immuno i.clcatindexdate ///
   i.nosos11cat i.canscore7cat nearest sta3ndistance

logit died_181_730 `covar' if case==0
lroc, nograph //0.830
predict mkg_pr_died181_730
gen mkg_pr_died181_730_case = mkg_pr_died181_730 if case==1

bysort matchgroupnumber (patienticn): egen pr_died181_730_case =
max(mkg_pr_died181_730_case)

* create tertiles of predicted probabilities of death in 181-730 days
xtile pr_died181_730_tertile = pr_died181_730_case, nq(3)
tab pr_died181_730_tertile

bysort pr_died181_730_tertile: sum pr_died181_730_case


drop mkg_pr*

*---------------------------------------------------------
* PP1 mortality for COVID+ Veterans and comparators
*---------------------------------------------------------

 /*

 Criteria
    1)  Case and at least 1 comparator in the strata must be alive on
the
   first day of the period

    2)  Patients who were censored due to an infection crossover
   in the previous period are excluded in the current period

    3)  Patients who die after the infection crossover are alive
   at the time of the infection crossover
 */
```

```
* Overall *

* 90-day mortality
tab died_0_90_crossover infected , co
cs died_0_90_crossover infected

* 180-day mortality
tab died_91_180_crossover infected if alive_day91_crosscasecontrol==1 ,
co
cs died_91_180_crossover infected if alive_day91_crosscasecontrol==1

* 365-day mortality
tab died_181_365_crossover infected if alive_day181_crosscasecontrol==1
, co
cs died_181_365_crossover infected if alive_day181_crosscasecontrol==1

* 730-day mortality
tab died_366_730_crossover infected if alive_day366_crosscasecontrol==1,
co
cs died_366_730_crossover infected if alive_day366_crosscasecontrol==1


************************************************************************
*
* Per Protocol #1 - Cox Models: Censoring at Crossover Infections
(Unadjusted) *
************************************************************************
*

*---------------------------
* Prepping for Cox Models
*---------------------------

** Censoring at 730 Days -Or- Among Comparators, at Infection Crossover
**

* count number who were COVID+ prior to death or study end
count if ttinfection730
replace tte_730_cross = indextodeath_caseindex if
indextodeath_caseindex730
replace died_730_cross = 0 if indextodeath_caseindex>ttinfection
replace died_730_cross = 0 if (indextodeath_caseindex>ttstudyend) &
(inrange(indextodeath_caseindex, 0, 730)) ///
          & (inrange(ttstudyend, 0, 730))

tab died_730_cross

*---------------------------------
* Per Protocol #1: Cox Models
*---------------------------------

preserve

 * stset data
```

```
 sort uniq_patid
 stset tte_730_cross, failure(died_730_cross) id(uniq_patid)
 stsplit period, at(0 90 180 365 730)

 * create new interaction variables after splitting data
 gen timeinfected_0_90 = infected * (tte_730_cross<=90)
 gen timeinfected_91_180 = infected * (tte_730_cross>90 &
tte_730_cross<=180)
 gen timeinfected_181_365 = infected * (tte_730_cross>180 &
tte_730_cross<=365)
 gen timeinfected_366_730 = infected * (tte_730_cross>365 &
tte_730_cross<=730)

 * cox model
 stcox  i.timeinfected_0_90 i.timeinfected_91_180 i.timeinfected_181_365
///
   i.timeinfected_366_730     ///
   , efron vce(cluster patienticn) strata(matchgroupnumber)

restore



*------------------------------
* Preparing dataset for IPCWs
*------------------------------

*save Data\prepdata_pp1_ipcw, replace

preserve

 * open dataset
 use Data\prepdata_pp1_ipcw, clear

 * create a fractional tte_730_cross variable by dividing by 32
 gen tte_month_cross = tte_730_cross/32

 * keep only variables needed for analysis
 keep  /* patient identifiers*/        ///
   matchgroupnumber patienticn uniq_patid case index_dt    ///
   index_dt_case            ///
   /* covariates */          ///
   ageatindexdate bmi ethnicity3cat smoking4cat    ///
   gagne numipadmits util_numpcstops util_numscstops   ///
   nosos11cat canscore7cat nearest sta3ndistance   ///
   /*variables for subgroup analyses*/      ///
   pr_died0_90_tertile pr_died181_730_tertile    ///
   age_less65_case age_65to85_case age_more85_case   ///
   gagne_tertile_case          ///
   /*variables for cox models*/       ///
   tte_month_cross died_730_cross tte_730_cross

 * organize dataset
 order uniq_patid, after(patienticn)
```

```
 order index_dt_case, after(index_dt)

 * set up dataset for analysis in SAS
 expand tte_month_cross +1 //+1 so that we can include  month 0
 bysort matchgroupnumber patienticn: gen monthend=_n

 gen month = monthend-1

 * drop expanded months in which the tte month is less than or equal to
the
 * start of the month; in the expand command, tte months that were x.5
 * added an additional month. we want to confirm that the tte month
 * always exceeds the start of the month
 drop if tte_month_cross<=month & tte_month_cross!=0

 * create mortality variable
 bysort matchgroupnumber patienticn: gen N = _N
 gen died = 0
 replace died = 1 if died_730_cross==1 & monthend==N

 * identify the last observation in the panel. this will be used for
 * filling in the new tte variable created (below) with the value in
 * the tte_month_cross variable for the last month the subject is in the
study
 gsort matchgroupnumber patienticn -month
 by matchgroupnumber patienticn: gen lastnum = _n

 * create tte
 gen tte=monthend

  * replace the new tte with the tte_month_cross variable in the month
when
  * tte occurs
  replace tte = tte_month_cross if lastnum==1

 sort matchgroupnumber patienticn month

 * censor each month unless it is a month when a patient dies
 gen censor = 1
 replace censor = 0 if died==1

 * merge in IPCWs
 bysort matchgroupnumber patienticn (monthend): gen panel_n=_n
 merge 1:1 matchgroupnumber patienticn panel_n using
Data\IPCW\ipcw_trunc
 drop if _merge==2

 * drop variables not needed for analysis
 drop died_730_cross tte_730_cross tte_month_cross monthend N smdate
year _merge panel_n

 * organize variables in dataset
 order matchgroupnumber patienticn uniq_patid case index_dt
index_dt_case month tte died censor ipcw
```

```
 * save dataset for inclusion of IPCWs
 *save Data\mortality_pp1_ipcw_20221215, replace

restore


***********************************
* PP2: With Censoring, Unweighted *
***********************************

use Data\prepdata_pp1_ipcw, clear

* check ttinfection with negative values
sum ttinfection
count if ttinfection==0

* identify the earliest infection crossover for the strata
gsort matchgroupnumber propensityscoreabsdiffrank -case
by matchgroupnumber: egen ttinfection_strata = min(ttinfection)

*----------------------------------------------------------------------------
--------
* identify those who die during each period;
* those who die after infection crossover are coded as not dead at the 
time of
* crossover
*----------------------------------------------------------------------------
--------

* Died Index Day-Day 90;
gen died_0_90_stratacrossover = .
replace died_0_90_stratacrossover = 1 if inrange(indextodeath_caseindex, 
0, 90)
replace died_0_90_stratacrossover = 0 if indextodeath_caseindex>90
replace died_0_90_stratacrossover = 0 if 
indextodeath_caseindex>ttinfection_strata
tab died_0_90 died_0_90_stratacrossover, m

* Died Day 91-180;
gen died_91_180_stratacrossover = .
replace died_91_180_stratacrossover = 1 if 
inrange(indextodeath_caseindex, 91, 180)
replace died_91_180_stratacrossover = 0 if indextodeath_caseindex>180
replace died_91_180_stratacrossover = 0 if 
indextodeath_caseindex>ttinfection_strata
replace died_91_180_stratacrossover = 0 if 
(indextodeath_caseindex>ttstudyend) & (inrange(indextodeath_caseindex, 
91, 180)) ///
          & (inrange(ttstudyend, 91, 180))
tab died_91_180 died_91_180_stratacrossover, m //missing represents 
those who were censored in period 1

* Died Day 181-365;
```

```
gen died_181_365_stratacrossover = .
replace died_181_365_stratacrossover = 1 if
inrange(indextodeath_caseindex, 181, 365)
replace died_181_365_stratacrossover = 0 if indextodeath_caseindex>365
replace died_181_365_stratacrossover = 0 if
indextodeath_caseindex>ttinfection_strata
replace died_181_365_stratacrossover = 0 if
(indextodeath_caseindex>ttstudyend) & (inrange(indextodeath_caseindex,
181, 365)) ///
          & (inrange(ttstudyend, 181, 365))
tab died_181_365 died_181_365_stratacrossover, m //missing represents
those who were censored in period 1&2

* Died Day 366-730;
gen died_366_730_stratacrossover = .
replace died_366_730_stratacrossover = 1 if
inrange(indextodeath_caseindex, 366, 730)
replace died_366_730_stratacrossover = 0 if indextodeath_caseindex>730
replace died_366_730_stratacrossover = 0 if
indextodeath_caseindex>ttinfection_strata
replace died_366_730_stratacrossover = 0 if
(indextodeath_caseindex>ttstudyend) & (inrange(indextodeath_caseindex,
366, 730)) ///
          & (inrange(ttstudyend, 366, 730))

tab died_366_730 died_366_730_stratacrossover, m //missing represents
those who were censored in period 1-3

*-------------------------------------------------------------------------
--------
* for each period, identify whether the case and at least one comparator
* in a matched group are alive on the first day of the period, AND
* their strata does not have an infection crossover in the prior
period(s)
*-------------------------------------------------------------------------
--------

* Alive on day 91 & no strata crossover in prior period
gen alive_day91_stratacrossover = .
replace alive_day91_stratacrossover = 1 if indextodeath_caseindex>=91
replace alive_day91_stratacrossover = 0 if
inrange(indextodeath_caseindex, 0, 90)
replace alive_day91_stratacrossover = 0 if inrange(ttinfection_strata,
0, 90)
replace alive_day91_stratacrossover = 0 if ttstudyend<91

sum ttinfection_strata if inrange(ttinfection_strata, 0, 90)
tab alive_day91 alive_day91_stratacrossover, m

* Alive on day 181 & no strata crossover in prior periods
gen alive_day181_stratacrossover = .
replace alive_day181_stratacrossover = 1 if indextodeath_caseindex>=181
replace alive_day181_stratacrossover = 0 if
inrange(indextodeath_caseindex, 0, 180)
```

```
replace alive_day181_stratacrossover = 0 if inrange(ttinfection_strata,
0, 180)
replace alive_day181_stratacrossover = 0 if ttstudyend<181

sum ttinfection_strata if inrange(ttinfection_strata, 0, 180)
tab alive_day181 alive_day181_stratacrossover

* Alive on day 366 & no strata crossover in prior periods
gen alive_day366_stratacrossover = .
replace alive_day366_stratacrossover = 1 if indextodeath_caseindex>=366
replace alive_day366_stratacrossover = 0 if
inrange(indextodeath_caseindex, 0, 365)
replace alive_day366_stratacrossover = 0 if inrange(ttinfection_strata,
0, 365)
replace alive_day366_stratacrossover = 0 if ttstudyend<366

sum ttinfection_strata if inrange(ttinfection_strata, 0, 365)
tab alive_day366 alive_day366_stratacrossover

*-----------------------------------------------------------------------
--------
* for each period, identify whether the case and at least one comparator
* in a matched group are alive on the first day of the period & were not
* in an infection crossover strata in the prior period
*-----------------------------------------------------------------------
--------

foreach i of numlist 91 181 366 {

 //cases
 gen alive_day`i'_stratacase = 0
 replace alive_day`i'_stratacase = 1 if case==1 &
alive_day`i'_stratacrossover==1
 replace alive_day`i'_stratacase = 0 if case==1 & ttstudyend<`i'
 tab alive_day`i'_stratacase alive_day`i'_stratacrossover if case==1
 sum indextodeath if indextodeath<`i' & case==1

 bysort matchgroupnumber: egen alive_day`i'_stratacase_grp =
max(alive_day`i'_stratacase)

 //comparators
 gen alive_day`i'_stratacontrol = 0
 replace alive_day`i'_stratacontrol = 1 if case==0 &
alive_day`i'_stratacrossover==1
 replace alive_day`i'_stratacontrol = 0 if case==0 & ttstudyend<`i'

 tab alive_day`i'_stratacontrol alive_day`i'_stratacrossover if case==0
 sum indextodeath if indextodeath<`i' & case==0

 bysort matchgroupnumber: egen alive_day`i'_stratacontrol_num =
sum(alive_day`i'_stratacontrol)

 //case and at least one comparator alive on day`i'
 gen alive_day`i'_stratacasecontrol = 0
```

```
 replace alive_day`i'_stratacasecontrol = 1 if
alive_day`i'_stratacase_grp==1 & alive_day`i'_stratacontrol_num>=1

 tab alive_day`i'_stratacasecontrol case

 //drop variables we no longer need
 drop alive_day`i'_stratacase alive_day`i'_stratacase_grp
alive_day`i'_stratacontrol alive_day`i'_stratacontrol_num

}


*-----------------
* PP2 Results
*-----------------

 /*

 Criteria
    1)  Case and at least 1 comparator in the strata must be alive on
the
   first day of the period

    2)  Everyone in the strata who was censored due to a strata
infection
   crossover in the previous period is excluded in the current period

    3)  People who die after the infection crossover are considered
alive
   at the time of the infection crossover
 */



* Overall *

* 90-day mortality
tab died_0_90_stratacrossover infected , co
cs died_0_90_stratacrossover infected

* 180-day mortality
tab died_91_180_stratacrossover infected if
alive_day91_stratacasecontrol==1 , co
cs died_91_180_stratacrossover infected if
alive_day91_stratacasecontrol==1

* 365-day mortality
tab died_181_365_stratacrossover infected if
alive_day181_stratacasecontrol==1 , co
cs died_181_365_stratacrossover infected if
alive_day181_stratacasecontrol==1

* 730-day mortality
tab died_366_730_stratacrossover infected if
```

```
alive_day366_stratacasecontrol==1 , co
cs died_366_730_stratacrossover infected if
alive_day366_stratacasecontrol==1

*-----------
* Age <65
*-----------

* 90-day mortality
tab died_0_90_stratacrossover infected if agecat==1, co
cs died_0_90_stratacrossover infected if agecat==1

* 180-day mortality
tab died_91_180_stratacrossover infected if
alive_day91_stratacasecontrol==1 & agecat==1, co
cs died_91_180_stratacrossover infected if
alive_day91_stratacasecontrol==1 & agecat==1

* 365-day mortality
tab died_181_365_stratacrossover infected if
alive_day181_stratacasecontrol==1 & agecat==1, co
cs died_181_365_stratacrossover infected if
alive_day181_stratacasecontrol==1 & agecat==1

* 730-day mortality
tab died_366_730_stratacrossover infected if
alive_day366_stratacasecontrol==1 & agecat==1, co
cs died_366_730_stratacrossover infected if
alive_day366_stratacasecontrol==1 & agecat==1

*------------
* Age 65-85
*------------

* 90-day mortality
tab died_0_90_stratacrossover infected if agecat==2, co
cs died_0_90_stratacrossover infected if agecat==2

* 180-day mortality
tab died_91_180_stratacrossover infected if
alive_day91_stratacasecontrol==1 & agecat==2, co
cs died_91_180_stratacrossover infected if
alive_day91_stratacasecontrol==1 & agecat==2

* 365-day mortality
tab died_181_365_stratacrossover infected if
alive_day181_stratacasecontrol==1 & agecat==2, co
cs died_181_365_stratacrossover infected if
alive_day181_stratacasecontrol==1 & agecat==2

* 730-day mortality
tab died_366_730_stratacrossover infected if
alive_day366_stratacasecontrol==1 & agecat==2, co
cs died_366_730_stratacrossover infected if
```

```
alive_day366_stratacasecontrol==1 & agecat==2

*------------
* Age 85+
*------------

* 90-day mortality
tab died_0_90_stratacrossover infected if agecat==3, co
cs died_0_90_stratacrossover infected if agecat==3

* 180-day mortality
tab died_91_180_stratacrossover infected if
alive_day91_stratacasecontrol==1 & agecat==3, co
cs died_91_180_stratacrossover infected if
alive_day91_stratacasecontrol==1 & agecat==3

* 365-day mortality
tab died_181_365_stratacrossover infected if
alive_day181_stratacasecontrol==1 & agecat==3, co
cs died_181_365_stratacrossover infected if
alive_day181_stratacasecontrol==1 & agecat==3

* 730-day mortality
tab died_366_730_stratacrossover infected if
alive_day366_stratacasecontrol==1 & agecat==3, co
cs died_366_730_stratacrossover infected if
alive_day366_stratacasecontrol==1 & agecat==3

*-----------
* Wave 1
*-----------

* 90-day mortality
tab died_0_90_stratacrossover infected if wave==1, co
cs died_0_90_stratacrossover infected if wave==1

* 180-day mortality
tab died_91_180_stratacrossover infected if
alive_day91_stratacasecontrol==1 & wave==1, co
cs died_91_180_stratacrossover infected if
alive_day91_stratacasecontrol==1 & wave==1

* 365-day mortality
tab died_181_365_stratacrossover infected if
alive_day181_stratacasecontrol==1 & wave==1, co
cs died_181_365_stratacrossover infected if
alive_day181_stratacasecontrol==1 & wave==1

* 730-day mortality
tab died_366_730_stratacrossover infected if
alive_day366_stratacasecontrol==1 & wave==1, co
cs died_366_730_stratacrossover infected if
alive_day366_stratacasecontrol==1 & wave==1
```

```
*-----------
* Wave 2
*-----------

* 90-day mortality
tab died_0_90_stratacrossover infected if wave==2, co
cs died_0_90_stratacrossover infected if wave==2

* 180-day mortality
tab died_91_180_stratacrossover infected if
alive_day91_stratacasecontrol==1 & wave==2, co
cs died_91_180_stratacrossover infected if
alive_day91_stratacasecontrol==1 & wave==2

* 365-day mortality
tab died_181_365_stratacrossover infected if
alive_day181_stratacasecontrol==1 & wave==2, co
cs died_181_365_stratacrossover infected if
alive_day181_stratacasecontrol==1 & wave==2

* 730-day mortality
tab died_366_730_stratacrossover infected if
alive_day366_stratacasecontrol==1 & wave==2, co
cs died_366_730_stratacrossover infected if
alive_day366_stratacasecontrol==1 & wave==2

*-----------
* Wave 3
*-----------

* 90-day mortality
tab died_0_90_stratacrossover infected if wave==3, co
cs died_0_90_stratacrossover infected if wave==3

* 180-day mortality
tab died_91_180_stratacrossover infected if
alive_day91_stratacasecontrol==1 & wave==3, co
cs died_91_180_stratacrossover infected if
alive_day91_stratacasecontrol==1 & wave==3

* 365-day mortality
tab died_181_365_stratacrossover infected if
alive_day181_stratacasecontrol==1 & wave==3, co
cs died_181_365_stratacrossover infected if
alive_day181_stratacasecontrol==1 & wave==3

* 730-day mortality
tab died_366_730_stratacrossover infected if
alive_day366_stratacasecontrol==1 & wave==3, co
cs died_366_730_stratacrossover infected if
alive_day366_stratacasecontrol==1 & wave==3

*-----------------------------------------------------------------
* PP2 Constructing TTE & Mortality Variables for Cox Model
```

```
*---------------------------------------------------------------

* censor matched strata at the time of an infection crossover;
* patients in a crossover strata who die prior to the infection
crossover
* will have their TTE recorded as their individual date of death

* censor everyone at the earliest censoring time of anyone in the
matched
* strata, using earliest time of death, study end, or crossover
gsort matchgroupnumber propensityscoreabsdiffrank -case

* create a new tte variable from the crossover tte variable, censoring
everyone
* in the strata at the time of the earliest infection crossover
by matchgroupnumber: egen mkg_tte_730_strata = min(tte_730_cross) if
isfutureinfectedcontrol==1
by matchgroupnumber: egen tte_730_strata = min(mkg_tte_730_strata)

* if someone dies prior to the time of the earliest censoring date for
the strata,
* recode THAT individual with the date of their death
replace tte_730_strata = tte_730_cross if
indextodeath_caseindextte_730_strata & !missing(indextodeath_caseindex)

* drop variables we no longer need
drop mkg_tte_730_strata

* PP2 - mortality and excess mortality among cases
tab died_730_strata infected, co chi // (Abstract & Results)

di 42399/1037423 //4.09 mortality rate of comparators (Abstract &
Results)
di 208061*0.04086954 //mortality rate of comparators applied to cases =
8503 deaths
di 18128-8503.3574 //9625 excess deaths

*----------------------------
* PP2 Kaplan Meier Curves
*----------------------------

** Creating Variables for KM **

* alive on index day, censored at 90 days
gen tte_0_90_strata = ttstudyend
replace tte_0_90_strata = 90 if ttstudyend>90
replace tte_0_90_strata = indextodeath_caseindex  ///
     if indextodeath_caseindex180
replace tte_91_180_strata = indextodeath_caseindex if  ///
      indextodeath_caseindex365
replace tte_181_365_strata = indextodeath_caseindex if  ///
      indextodeath_caseindex730
replace tte_366_730_strata = indextodeath_caseindex if  ///
      indextodeath_caseindex90 & tte_730_strata<=180)
```

```
 gen timeinfected_181_365 = infected * (tte_730_strata>180 &
tte_730_strata<=365)
 gen timeinfected_366_730 = infected * (tte_730_strata>365 &
tte_730_strata<=730)

 *-----------------------
 * Main Model - Unadj.
 *-----------------------

 stcox  timeinfected_0_90 timeinfected_91_180 timeinfected_181_365  ///
   timeinfected_366_730     ///
   , efron vce(cluster patienticn) strata(matchgroupnumber)

restore


log close
```
