# Supplementary material for: Late Mortality After COVID-19 Infection Among US Veterans vs Risk-Matched Comparators: A 2-Year Cohort Analysis
Source: JAMA Intern Med. 2023 Aug 21;183(10):1111–9. doi: 10.1001/jamainternmed.2023.3587 (PMC10442778; doi:10.1001/jamainternmed.2023.3587)
Supplement: Supplement 4. — Nonauthor Collaborators. The VA HSD&R Collaboratory nonauthor collaborators [file jamainternmed-e233587-s004.pdf]

Supplementary Online Material: Nonauthor Collaborators
*First name, last name, and suffix (if applicable) are required and will appear in PubMed.

*Group Name(s): VA HSR&D COVID-19 Observational Research Collaboratory (CORC)							
***First Name and Middle Initial(s)**	***Last Name**	***Suffix (eg, Jr, III)**	Academic Degrees	Institution	Location (city, state/province, country)	Role or Contribution, eg, chair, principal investigator	Group (if more than 1 Group listed in the byline) and/or Subgroup (eg, Steering Committee)
Andrew	Admon		MD	VA Ann Arbor Healthcare System	Ann Arbor, MI	Site Collaborator	**CORC Participating Collaborators**
Kathleen	Akgun		MD	VA Connecticut Healthcare System	West Haven, CT	Site Collaborator	**CORC Participating Collaborators**
Stacy	Anderson		BA	VA Ann Arbor Healthcare System	Ann Arbor, MI	Research Assistant	**CORC Participating Collaborators**
Mihaela	Aslan		PhD	VA Connecticut Healthcare System	West Haven, CT	Site Collaborator	**CORC Participating Collaborators**
David	Au		MD	VA Puget Sound Healthcare System	Seattle, WA	Physician Consultant	**CORC Participating Collaborators**
Lisa	Backus		MD	VA Palo Alto Healthcare System	Palo Alto, CA	Site Collaborator	**CORC Participating Collaborators**
Kristina	Bajema		MD	VA Portland Healthcare System	Portland, OR	Site Collaborator	**CORC Participating Collaborators**
Aaron	Baraff		MS	VA Puget Sound Healthcare System	Seattle, WA	Data Analyst	**CORC Participating Collaborators**
Lisa	Batten		MD	VA Puget Sound Healthcare System	Seattle, WA	Project Manager	**CORC Participating Collaborators**
Theodore	Berkowitz		MS	VA Durham Healthcare System	Durham, NC	Data Manager	**CORC Participating Collaborators**
Taylor	Bernstein		MPH	VA Ann Arbor Healthcare System	Ann Arbor, MI	Research Assistant	**CORC Participating Collaborators**
Kristin	Berry Wyatt		PhD	VA Puget Sound Healthcare System	Seattle, WA	Data Manager	**CORC Participating Collaborators**
Joseph	Bogdan		BA	VA Durham Healthcare System	Durham, NC	Project Manager	**CORC Participating Collaborators**
Hayden	Bosworth		PhD	VA Durham Healthcare System	Durham, NC	Site Collaborator	**CORC Participating Collaborators**

Supplementary Online Material: Nonauthor Collaborators
*First name, last name, and suffix (if applicable) are required and will appear in PubMed.

***First Name and Middle Initial(s)**	***Last Name**	***Suffix (eg, Jr, III)**	Academic Degrees	Institution	Location (city, state/province, country)	Role or Contribution, eg, chair, principal investigator	Group (if more than 1 Group listed in the byline) and/or Subgroup (eg, Steering Committee)
Nathan	Boucher		PhD	VA Durham Healthcare System	Durham, NC	Site Collaborator	**CORC Participating Collaborators**
Nicholas	Burwick		MD	VA Puget Sound Healthcare System	Seattle, WA	Physician Consultant	**CORC Participating Collaborators**
Aissa	Cabrales		BA	VA Ann Arbor Healthcare System	Ann Arbor, MI	Research Assistant	**CORC Participating Collaborators**
Jennifer	Cano		MPH	VA Ann Arbor Healthcare System	Ann Arbor, MI	Data Analyst	**CORC Participating Collaborators**
Wen	Chai		BS	VA Durham	Durham, NC	Data Analyst	**CORC Participating Collaborators**
Jason	Chen		MD	VA Portland Healthcare System	Portland, OR	Site Collaborator	**CORC Participating Collaborators**
Kei-Hoi	Cheung		PhD	VA Connecticut Healthcare System	West Haven, CT	Site Collaborator	**CORC Participating Collaborators**
Kristina	Crothers		MD	VA Puget Sound Healthcare System	Seattle, WA	Site Collaborator	**CORC Participating Collaborators**
Jeffrey	Curtis		MD	VA Ann Arbor Healthcare System	Ann Arbor, MI	Physician Consultant	**CORC Participating Collaborators**
Marie	Davis		MD	VA Puget Sound Healthcare System	Seattle, WA	Physician Consultant	**CORC Participating Collaborators**
Emily	Del Monico		MPH	VA Ann Arbor Healthcare System	Ann Arbor, MI	Research Assistant	**CORC Participating Collaborators**
Aram	Dobalian		PhD, JD	VA Greater Los Angeles Healthcare System	Los Angeles, CA	Physician Consultant	**CORC Participating Collaborators**
Jacob	Doll		MD	VA Puget Sound Healthcare System	Seattle, WA	Physician Consultant	**CORC Participating Collaborators**
Jason	Dominitz		MD	VA Puget Sound Healthcare System	Seattle, WA	Physician Consultant	**CORC Participating Collaborators**
McKenna	Eastment		MD	VA Puget Sound Healthcare System	Seattle, WA	Physician Consultant	**CORC Participating Collaborators**

Supplementary Online Material: Nonauthor Collaborators
*First name, last name, and suffix (if applicable) are required and will appear in PubMed.

***First Name and Middle Initial(s)**	***Last Name**	***Suffix (eg, Jr, III)**	Academic Degrees	Institution	Location (city, state/province, country)	Role or Contribution, eg, chair, principal investigator	Group (if more than 1 Group listed in the byline) and/or Subgroup (eg, Steering Committee)
Vincent	Fan		MD	VA Puget Sound Healthcare System	Seattle, WA	Physician Consultant	**CORC Participating Collaborators**
Jacqueline	Ferguson		PhD	VA Palo Alto Healthcare System	Palo Alto, CA	Site Collaborator	**CORC Participating Collaborators**
Breanna	Floyd		MPH	VA Durham Healthcare System	Durham, NC	Site Collaborator	**CORC Participating Collaborators**
Alexandra	Fox		MS	VA Puget Sound Healthcare System	Seattle, WA	Data Analyst	**CORC Participating Collaborators**
Matthew	Goetz		MD	VA Greater Los Angeles Healthcare System	Los Angeles, CA	Physician Consultant	**CORC Participating Collaborators**
Diana	Govier		PhD	VA Portland Healthcare System	Portland, OR	Data Analyst	**CORC Participating Collaborators**
Pamela	Green		PhD	VA Puget Sound Healthcare System	Seattle, WA	Data Manager	**CORC Participating Collaborators**
Susan Nicole	Hastings		MD	VA Durham Healthcare System	Durham, NC	Physician Consultant	**CORC Participating Collaborators**
Katie	Hauschildt		PhD	VA Ann Arbor Healthcare System	Ann Arbor, MI	Site Collaborator	**CORC Participating Collaborators**
Eric	Hawkins		PhD	VA Puget Sound Healthcare System	Seattle, WA	Site Collaborator	**CORC Participating Collaborators**
Paul	Hebert		PhD	VA Puget Sound Healthcare System	Seattle, WA	Site Collaborator	**CORC Participating Collaborators**
Mark	Helfand		PhD	VA Portland Healthcare System	Portland, OR	Site Collaborator	**CORC Participating Collaborators**
Alex	Hickok		MS	VA Portland Healthcare System	Portland, OR	Data Analyst	**CORC Participating Collaborators**
Dana	Horowitz		MSW	VA Ann Arbor Healthcare System	Ann Arbor, MI	Research Assistant	**CORC Participating Collaborators**
Catherine	Hough		MD	VA Portland Healthcare System	Portland, OR	Physician Consultant	**CORC Participating Collaborators**

Supplementary Online Material: Nonauthor Collaborators
*First name, last name, and suffix (if applicable) are required and will appear in PubMed.

***First Name and Middle Initial(s)**	***Last Name**	***Suffix (eg, Jr, III)**	Academic Degrees	Institution	Location (city, state/province, country)	Role or Contribution, eg, chair, principal investigator	Group (if more than 1 Group listed in the byline) and/or Subgroup (eg, Steering Committee)
Elaine	Hu		MS	VA Puget Sound Healthcare System	Seattle, WA	Data Analyst	**CORC Participating Collaborators**
Kevin	Ikuta		MD	VA Greater Los Angeles Healthcare System	Los Angeles, CA	Physician Consultant	**CORC Participating Collaborators**
Barbara	Jones		MD	VA Salt Lake City Healthcare System	Salt Lake City, UT	Site Collaborator	**CORC Participating Collaborators**
Makoto	Jones		MD	VA SLC Healthcare System	Salt Lake City, UT	Physician Consultant	**CORC Participating Collaborators**
Lee	Kamphuis		MPH	VA Ann Arbor Healthcare System	Ann Arbor, MI	Project Manager	**CORC Participating Collaborators**
Brystana	Kaufman		PhD	VA Durham Healthcare System	Durham, NC	Site Collaborator	**CORC Participating Collaborators**
Sara	Knight		PhD	VA Salt Lake City Healthcare System	Salt Lake City, UT	Site Collaborator	**CORC Participating Collaborators**
Anna	Korpak		PhD	VA Puget Sound Healthcare System	Seattle, WA	Data Analyst	**CORC Participating Collaborators**
Peggy	Korpela		MPH	VA Ann Arbor Healthcare System	Ann Arbor, MI	Research Assistant	**CORC Participating Collaborators**
Kyle	Kumbier		MS	VA Ann Arbor Healthcare System	Ann Arbor, MI	Data Analyst	**CORC Participating Collaborators**
Kenneth	Langa		MD	VA Ann Arbor Healthcare System	Ann Arbor, MI	Physician Consultant	**CORC Participating Collaborators**
Ryan	Laundry		BS	VA Puget Sound Healthcare System	Seattle, WA	Data Analyst	**CORC Participating Collaborators**
Stacy	Lavin		PhD	VA Durham Healthcare System	Durham, NC	Site Collaborator	**CORC Participating Collaborators**
Yuli	Li		MS	VA Connecticut Healthcare System	West Haven, CT	Site Collaborator	**CORC Participating Collaborators**
Jennifer	Linquist		PhD	VA Durham Healthcare System	Durham, NC	Data Analyst	**CORC Participating Collaborators**

Supplementary Online Material: Nonauthor Collaborators
*First name, last name, and suffix (if applicable) are required and will appear in PubMed.

***First Name and Middle Initial(s)**	***Last Name**	***Suffix (eg, Jr, III)**	Academic Degrees	Institution	Location (city, state/province, country)	Role or Contribution, eg, chair, principal investigator	Group (if more than 1 Group listed in the byline) and/or Subgroup (eg, Steering Committee)
Holly	McCready		BS	VA Portland Healthcare System	Portland, OR	Project Manager	**CORC Participating Collaborators**
Martha	Michel		PhD	VA Puget Sound Healthcare System	Seattle, WA	Data Analyst	**CORC Participating Collaborators**
Amy	Miles		MPH	VA Durham Healthcare System	Durham, NC	Site Collaborator	**CORC Participating Collaborators**
Jessie	Milne		MPH	VA Ann Arbor Healthcare System	Ann Arbor, MI	Research Assistant	**CORC Participating Collaborators**
Max	Monahan		MPH	VA Ann Arbor Healthcare System	Ann Arbor, MI	Project Manager	**CORC Participating Collaborators**
Daniel	Morelli		BA	VA Puget Sound Healthcare System	Seattle, WA	Data Analyst	**CORC Participating Collaborators**
Pradeep	Mutalik		MD	VA Connecticut Healthcare System	West Haven, CT	Site Collaborator	**CORC Participating Collaborators**
Jennifer	Naylor		MD	VA Durham Healthcare System	Durham, NC	Site Collaborator	**CORC Participating Collaborators**
Meike	Neiderhausen		PhD	VA Portland Healthcare System	Portland, OR	Data Analyst	**CORC Participating Collaborators**
Summer	Newell		PhD	VA Portland Healthcare System	Portland, OR	Site Collaborator	**CORC Participating Collaborators**
Shannon	Nugent		PhD	VA Portland Healthcare System	Portland, OR	Site Collaborator	**CORC Participating Collaborators**
Michael	Ong		MD, PhD	VA Greater Los Angeles Healthcare System	Los Angeles, CA	Physician Consultant	**CORC Participating Collaborators**
Thomas	Osborne		MD	VA Palo Alto Healthcare System	Palo Alto, CA	Site Collaborator	**CORC Participating Collaborators**
Matthew	Peterson		MS	VA Portland Healthcare System	Portland, OR	Data Manager	**CORC Participating Collaborators**
Alexander	Peterson		MS	VA Puget Sound Healthcare System	Seattle, WA	Data Analyst	**CORC Participating Collaborators**

***First Name and Middle Initial(s)**	***Last Name**	***Suffix (eg, Jr, III)**	Academic Degrees	Institution	Location (city, state/province, country)	Role or Contribution, eg, chair, principal investigator	Group (if more than 1 Group listed in the byline) and/or Subgroup (eg, Steering Committee)
Nallakkandi	Rajeevan		PhD	VA Connecticut Healthcare System	West Haven, CT	Site Collaborator	**CORC Participating Collaborators**
Ashok	Reddy		MD	VA Puget Sound Healthcare System	Seattle, WA	Physician Consultant	**CORC Participating Collaborators**
Marylena	Rouse		BS	VA Ann Arbor Healthcare System	Ann Arbor, MI	Research Assistant	**CORC Participating Collaborators**
Mazhgan	Rowneki		MPH	VA Portland Healthcare System	Portland, OR	Data Manager	**CORC Participating Collaborators**
Som	Saha		MD	VA Portland Healthcare System	Portland, OR	Physician Consultant	**CORC Participating Collaborators**
Sameer	Saini		MD	VA Ann Arbor Healthcare System	Ann Arbor, MI	Physician Consultant	**CORC Participating Collaborators**
Javeed	Shah		MD	VA Puget Sound Healthcare System	Seattle, WA	Physician Consultant	**CORC Participating Collaborators**
Troy	Shahoumian		PhD	VA Palo Alto Healthcare System	Palo Alto, CA	Data Analyst	**CORC Participating Collaborators**
Aasma	Shaukat		MD	VA Minneapolis Healthcare System	Minneapolis, MN	Physician Consultant	**CORC Participating Collaborators**
Megan	Shepherd-Banigan		PhD	VA Durham Healthcare System	Durham, NC	Site Collaborator	**CORC Participating Collaborators**
Whitney	Showalter		PhD	VA Puget Sound Healthcare System	Seattle, WA	Project Manager	**CORC Participating Collaborators**
Christopher	Slatore		MD	VA Portland Healthcare System	Portland, OR	Physician Consultant	**CORC Participating Collaborators**
Nicholas	Smith		PhD	VA Puget Sound Healthcare System	Seattle, WA	Site Collaborator	**CORC Participating Collaborators**
Battista	Smith		MPH	VA Durham Healthcare System	Durham, NC	Project Manager	**CORC Participating Collaborators**
Pardeep	Suri		MD	VA Puget Sound Healthcare System	Seattle, WA	Physician Consultant	**CORC Participating Collaborators**

Supplementary Online Material: Nonauthor Collaborators
*First name, last name, and suffix (if applicable) are required and will appear in PubMed.

***First Name and Middle Initial(s)**	***Last Name**	***Suffix (eg, Jr, III)**	Academic Degrees	Institution	Location (city, state/province, country)	Role or Contribution, eg, chair, principal investigator	Group (if more than 1 Group listed in the byline) and/or Subgroup (eg, Steering Committee)
Jeremy	Sussman		MD	VA Ann Arbor Healthcare System	Ann Arbor, MI	Physician Consultant	**CORC Participating Collaborators**
Yumie	Takata		PhD	VA Portland Healthcare System	Portland, OR	Site Collaborator	**CORC Participating Collaborators**
Alan	Teo		MD	VA Portland Healthcare System	Portland, OR	Site Collaborator	**CORC Participating Collaborators**
Eva	Thomas		MPH	VA Puget Sound Healthcare System	Seattle, WA	Data Analyst	**CORC Participating Collaborators**
Laura	Thomas		MPH/MSW	VA Ann Arbor Healthcare System	Ann Arbor, MI	Project Manager	**CORC Participating Collaborators**
Anais	Tuepker		PhD	VA Portland Healthcare System	Portland, OR	Site Collaborator	**CORC Participating Collaborators**
Zachary	Veigulis		MS	VA Palo Alto Healthcare System	Palo Alto, CA	Data Analyst	**CORC Participating Collaborators**
Elizabeth	Vig		MD	VA Puget Sound Healthcare System	Seattle, WA	Site Collaborator	**CORC Participating Collaborators**
Kelly	Vranas		MD	VA Portland Healthcare System	Portland, OR	Site Collaborator	**CORC Participating Collaborators**
Xiao Qing	Wang		MPH	VA Ann Arbor Healthcare System	Ann Arbor, MI	Data Manager	**CORC Participating Collaborators**
Katrina	Wicks		MPH	VA Puget Sound Healthcare System	Seattle, WA	Data Analyst	**CORC Participating Collaborators**
Kara	Winchell		MA	VA Portland Healthcare System	Portland, OR	Project Manager	**CORC Participating Collaborators**
Edwin	Wong		PhD	VA Puget Sound Healthcare System	Seattle, WA	Site Collaborator	**CORC Participating Collaborators**
Chris	Woods		MD	VA Durham Healthcare System	Durham, NC	Physician Consultant	**CORC Participating Collaborators**
Katherine	Wysham		MD	VA Puget Sound Healthcare System	Seattle, WA	Physician Consultant	**CORC Participating Collaborators**

Supplementary Online Material: Nonauthor Collaborators
*First name, last name, and suffix (if applicable) are required and will appear in PubMed.

***First Name and Middle Initial(s)**	***Last Name**	***Suffix (eg, Jr, III)**	Academic Degrees	Institution	Location (city, state/province, country)	Role or Contribution, eg, chair, principal investigator	Group (if more than 1 Group listed in the byline) and/or Subgroup (eg, Steering Committee)
Lei	Yan		PhD	VA Connecticut Healthcare System	West Haven, CT	Site Collaborator	**CORC Participating Collaborators**
Donna	Zulman		MD	VA Palo Alto Healthcare System	Palo Alto, CA	Physician Consultant	**CORC Participating Collaborators**
