# Supplementary material for: Late Mortality After COVID-19 Infection Among US Veterans vs Risk-Matched Comparators: A 2-Year Cohort Analysis
Source: JAMA Intern Med. 2023 Aug 21;183(10):1111–9. doi: 10.1001/jamainternmed.2023.3587 (PMC10442778; doi:10.1001/jamainternmed.2023.3587)
Supplement: Supplement 5. — Data Sharing Statement [file jamainternmed-e233587-s005.pdf]

Data Sharing Statement

Iwashyna. Late Mortality After COVID-19 Infection Among US Veterans vs Risk-Matched Comparators. *JAMA Intern Med*. Published August 21, 2023.
doi:10.1001/jamainternmed.2023.3587

Data
Data available: No

Additional Information
Explanation for why data not available: Individual patient data will be made available with an approved Dept of Veterans Affairs data sharing plan
